# Mathematical modeling of the molecular switch of TNFR1-mediated signaling pathways applying Petri net formalism and *in silico* knockout analysis

**DOI:** 10.1371/journal.pcbi.1010383

**Published:** 2022-08-22

**Authors:** Leonie K. Amstein, Jörg Ackermann, Jennifer Hannig, Ivan Đikić, Simone Fulda, Ina Koch

**Affiliations:** 1 Goethe University Frankfurt, Institute of Computer Science, Department of Molecular Bioinformatics, Frankfurt am Main, Germany; 2 Cognitive Information Systems, Kompetenzzentrum für Informationstechnologie, Technische Hochschule Mittelhessen, Friedberg, Germany; 3 Goethe University Frankfurt, Institute of Biochemistry II, Medical Faculty, Frankfurt am Main, Germany; University of Pittsburgh, UNITED STATES

## Abstract

The paper describes a mathematical model of the molecular switches of cell survival, apoptosis, and necroptosis in cellular signaling pathways initiated by tumor necrosis factor 1. Based on experimental findings in the literature, we constructed a Petri net model based on detailed molecular reactions of the molecular players, protein complexes, post-translational modifications, and cross talk. The model comprises 118 biochemical entities, 130 reactions, and 299 edges. We verified the model by evaluating invariant properties of the system at steady state and by *in silico* knockout analysis. Applying Petri net analysis techniques, we found 279 pathways, which describe signal flows from receptor activation to cellular response, representing the combinatorial diversity of functional pathways.120 pathways steered the cell to survival, whereas 58 and 35 pathways led to apoptosis and necroptosis, respectively. For 65 pathways, the triggered response was not deterministic and led to multiple possible outcomes. We investigated the *in silico* knockout behavior and identified important checkpoints of the TNFR1 signaling pathway in terms of ubiquitination within complex I and the gene expression dependent on NF-κB, which controls the caspase activity in complex II and apoptosis induction. Despite not knowing enough kinetic data of sufficient quality, we estimated system’s dynamics using a discrete, semi-quantitative Petri net model.

## Author summary

It is still a challenge to develop mechanistic models for big molecular systems without the knowledge of enough kinetic parameters of sufficient quality. At the same time, more qualitative and semi-quantitative data have been produced in increasing numbers, e.g., by new high-throughput technologies. This has generated demands for new concepts at appropriate abstraction levels. The Petri net formalism enables the integration of qualitative as well as quantitative data and provides algorithms and methods for model verification and model simulation. Moreover, Petri nets exhibit a clear and coherent visualization. Here, we modeled the molecular switches between cell survival, apoptosis, and necroptosis induced by tumor necrosis factor 1. We were interested not only in an exhaustive exploration of all possible signaling pathways, but also in finding the system’s checkpoints. Our Petri net model comprises 118 biochemical entities, 130 reactions, and 299 edges. We found 279 pathways that describe signal flows from receptor activation to cellular response.120 pathways steered the cell to survival, whereas 58 and 35 pathways led to apoptosis and necroptosis, respectively. For 65 pathways, the triggered response was not deterministic, leading to multiple possible outcomes. We applied *in silico* knockout analyses to the Petri net model and could identify important checkpoints of the tumor necrosis factor 1 signaling pathway.

## Introduction

The tumor necrosis factor receptor 1 (TNFR1) controls pivotal cellular processes involved in immunity and developmental processes [1]. TNFR1 mediates signaling pathways, which induce opposing cellular responses from initiation of gene expression to two forms of cell death, apoptosis and necroptosis [2,3]. Apoptosis has long been viewed as the only form of cell death, which is initiated by the cell itself. Apoptosis is regulated by a specific family of death effector enzymes—the caspases [4]. A cascade of caspase activation leads to the cleavage of substrates that initiate further processes of the cell death machinery [5]. Two major pathways of apoptosis induction exist, the extrinsic pathway via the direct activation of effector caspase 3 (CASP3) by caspase 8 (CASP8) and the intrinsic pathway via the mitochondrion, inducing mitochondrial outer membrane permeabilization (MOMP) and eventually leading to CASP3 activation [6]. At several stages of apoptosis induction, inhibitory events can prevent apoptosis, the ratio between anti-apoptotic and pro-apoptotic proteins tips the balance. For a more detailed description of the extrinsic and intrinsic pathways, see **S1 Text**.

Whereas apoptosis is a well-known and well-studied pathway, the regulation and function of the necroptosis pathway has just recently been discovered and is still under study [7,8]. Necroptosis describes a cell death mode that exhibits the phenotype of necrosis, although it is ordered and controlled like apoptosis [9]. Alike necrosis, necroptosis features a form of cellular explosion, releasing the cellular content into the cell surrounding and initiating inflammation in the tissue [9]. On the contrary, cells that undergo apoptosis recycle most of the cellular molecules to reserve the energy and slowly digest themselves without inducing an inflammatory response in the surrounding cells [4]. Necroptosis seems to play a crucial role in nonalcoholic fatty liver disease, nonalcoholic steatohepatitis, and liver cancer [10].

Alternatively to cell death, the activation of nuclear factor κ-light-chain-enhancer of activated B cells (NF-κB) initiates the gene expression of mainly pro-inflammatory and anti-apoptotic operating genes [3]. Therefore, the NF-κB pathway is often referred to as the survival pathway triggered by TNFR1 stimulation [1]. A permanent activation of NF-κB can result in chronical inflammation and promote the formation of tumors [11]. In cancer cells, the gene expression is often permanently active, for example, by a disruption of the TNFR1 signaling pathway, such that the cells exhibit a resistance against cell death induction. Anticancer therapy aims to induce cell death in cancer cells often by triggering apoptosis pathways [12–16] and therapeutic exploitation of necroptosis [17].

**Fig 1:**
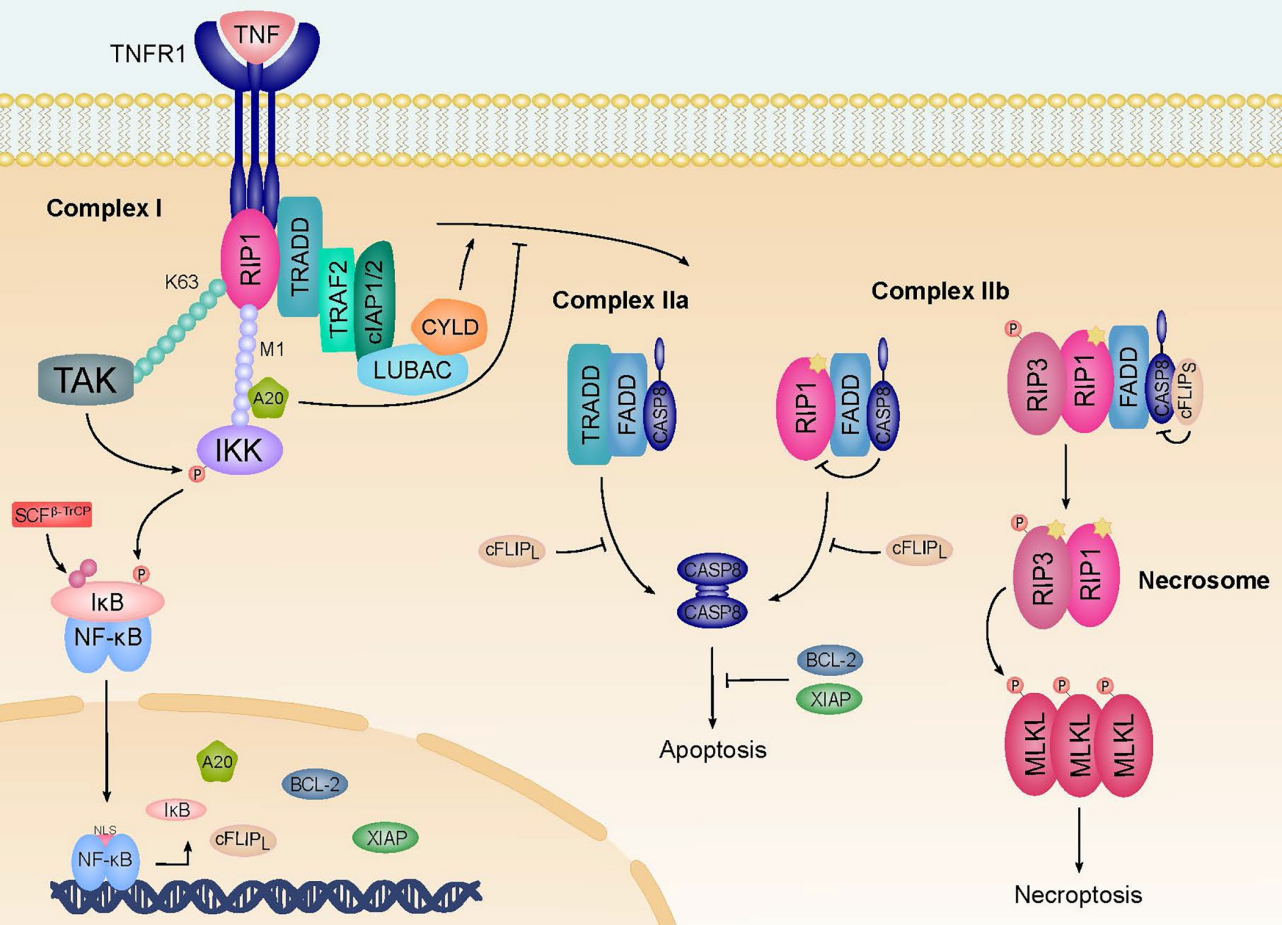
The TNFR1 signal transduction pathway. Upon engagement of TNFR1, complex I is rapidly formed and mediates the signaling to NF-κB activation. The ubiquitination mediated by E3 ligases, like cellular inhibitor of apoptosis protein 1 (cIAP1) or cellular inhibitor of apoptosis protein 2 (cIAP2) and linear ubiquitin chain assembly complex (LUBAC), promotes the association of complex I. The Ub modification is required for full activation of the inhibitor of NF-κB (IκB) and subsequent NF-κB activation. Activated NF-κB in the nucleus initiates the expression of target genes like IκB, A20, cellular FLICE-inhibitory protein (cFLIP_L_), B-cell lymphoma 2 (BCL-2), and X-linked inhibitor of apoptosis protein (XIAP). A20 is a deubiquitinating enzyme (DUB), which is reported to cleave lysine 63 (K63) chains while protecting methionine 1 (M1) chains from cleavage. The deubiquitination by CYLD (cylindromatosis) destabilizes the complex and promotes the formation of complex II in the cytosol. Complex IIa associates caspase 8 (CASP8), while complex IIb additionally binds RIP1. cFLIP_L_ reduces, but does not fully inhibit, caspase activity, which leads to RIP1 and RIP3 cleavage and inhibits apoptosis and necroptosis. cFLIP_S_ fully inhibits caspase activity and promotes the formation of the necrosome. Autophosphorylation of RIP3 allows the recruitment and phosphorylation of MLKL, which subsequently forms active oligomers and translocates to the plasma membrane to induce necroptosis.

The regulation of the opposing signaling cascades has been often considered as a molecular switch. Receptor-interacting protein 1 (RIP1) seems to have a pivotal function in modulating the controversial outcomes since it is an essential signaling node in all pathways, see **Fig 1**. The activity and function of RIP1 is sensitively controlled [18], for example, by post-translational modifications, such as phosphorylation and ubiquitination. During ubiquitination, ubiquitin (Ub) covalently attaches Ub molecules to substrate proteins, forming chains of different linkage types [19] and assigning specific functions to the respective proteins [20]. Linear Ub chains influence the modulation and control of activity in signal transduction [21–23]. The Ub system may have a promising therapeutic potential similar to the post-translational modification of phosphorylation mediated by kinases [14,24].

Although high-throughput technologies have provided many experimental data, there is a lack regarding the quality, quantity, and completeness of the data. Computational models are powerful approaches to represent and understand the complexity of biological systems. Computational systems biology can provide information on the system-wide behavior without knowing kinetic parameters. Systematic analyses can gain new insights of regulation, reveal correlations in diseases and pathologies, and can suggest potential targets for therapeutic treatment [25,26]. Newly emerging experimental procedures, in combination with improved computational methods, are promising approaches to analyze signaling pathways also with regard to therapeutic intervention and drug treatment [27]. The available data and the questions to be addressed determine the modeling approach. These approaches cover kinetic or stochastic, quantitative models, for example, systems of ordinary differential equations (ODEs) [28], qualitative models as Boolean models [29,30], or semi-quantitative models, as, for example, Petri nets (PNs) [31,32]. PNs allow for qualitative discrete modeling as well as for quantitative, continuous modeling. PNs have been widely applied to model biological pathways at different scales of abstraction, including metabolic systems, signal transduction pathways, gene regulatory systems [33–39]. Additionally, PNs provide a simplified and clear user-friendly visualization of the model graph [40,41].

The TNFR1 signaling pathway has often been a subject of mathematical modeling [42]. The models aimed to describe dynamics, regulations, and crosstalk of the NF-κB pathway [43,44]. On the one hand, the NF-κB regulation is well- characterized and has often been analyzed by quantitative modeling approaches, such as, e.g., an ODE-based model of the NF-κB signaling module [45]. According to new measured values and estimated parameters, there exist various adaptations and further developments of this model [46–51]. Other ODE-based applications consider, for example, oscillation dynamics of the functional switching of NF-κB for B-cell activation [52] or therapeutic questions on NF-κB synthetic decoy oligodeoxynucleotides [53]. On the other hand, new insights have often supersede older views of the regulation and have initiated the development of, for example, a hybrid PN of NF-κB activation and regulation of gene expression [54]. A Boolean model have described the interplay between NF-κB activation, apoptosis, and necroptosis, following the stimulation of TNFR1 and FAS receptor [55]. Schlatter *et al*. have proposed a Boolean model of the processes of apoptosis, which considers several stimuli [56]. Schliemann *et al*. have merged two existing models to an ODE model with pro- and anti-apoptotic responses of TNFR1 signaling [57]. Melas *et al*. have introduced a hybrid model, covering the stimulation of seven receptors and 22 cytokine stimuli in immunological pathways [58]. Very recently, Mothes *et al*. have investigated effects of different A20 feedback implementations for the NF-κB signaling dynamics, applying ODE modeling techniques [59]. All these models have aimed do describe molecular systems in detail. Their focus have been specific processes or stimuli. No previous model has considered the entire molecular switch between cell survival, apoptosis, and necroptosis.

The optimal modeling approach should be guided by the current knowledge and the amount and quality of the available data of the biological system under study and by the questions that should be addressed. For signaling pathways, many qualitative information is available, although exact parameters and kinetic data are scarce. The reason is that signaling systems exhibit kinetics, which is hard to elucidate in experimental investigations. Therefore, biological systems often lack of sufficient data. Only small-sized systems have been modeled and analyzed using equation-based approaches, for example, the IκB-NF-κB signaling module [45]. Qualitative information is extensively available and have been derived from, e.g., knockout experiments or pulldown assays to identify the components and causal relations of a pathway. The huge amount of qualitative data encourages the development and application of topology-based models to molecular systems. To integrate as much as possible of knowledge in a mathematical model, we abstracted from a complete set of kinetic parameters, as, e.g., concentrations and reaction rates. Instead, we have been applying a theoretical concept that allows semi-quantitative modeling if sufficient data is available and otherwise enables an appropriate abstraction level: the Petri net formalism.

### Petri nets

We apply a mathematical formalism called Petri nets (PNs) that enables to explore the dynamics in a non-deterministic way. For model construction, we need the knowledge on the chemical reactions, such as complex formation, phosphorylation, ubiquitination, or metabolic reactions. This active part of the system is modeled as *transitions* visualized as squares or rectangles. The chemical or biological entities, like proteins, DNA, RNA, or metabolites, are modeled as *places* drawn as circles. Places and transitions are connected via directed edges. To model the dynamics of a system, we used movable objects—the *tokens*. Places can carry tokens, which represent a number of an entity, e.g., one mole of a compound. Tokens can move through the system according to specific *firing* rules. Starting with an initial distribution of the tokens on the places that could correspond to a physiological state, we interpret a flow of tokens as a flow of substances or a flow of signals. An important feature of PNs is the availability of mathematically proven techniques for verification of consistency and of correctness of a model [60,61]. In particular for biological systems, the analysis of system’s invariants provide valuable insights. Invariants remain true for each possible state of the system. Biologically, invariants correspond to general principles that are valid under steady-state conditions. We apply *place invariants and transition invariants*. Place invariants are sets of places (entities) that describe the conservation of substances. A transition invariant is a set of transitions that represents a specific fundamental pathway. Transition invariants decompose the PN model into modules. The module of a transition invariant is the subnetwork defined by its transitions (reactions), the places connected to the transitions, and edges between the transitions and places. For biological models, transition invariants represent functional modules. Each reaction should be part of a transition invariant, otherwise the reaction could be removed from the model or the model contains an error. Thus, transition invariants can be applied to check the model for consistency and partly also for its correctness. For more detailed information, we refer to section “*Materials and Methods*”.

In this paper, we were interested in an exhaustive modeling of the molecular switch behavior of the TFNR1-induced signaling pathway, covering the NF-κB pathway, apoptosis, and necroptotic processes. Our model primarily focuses on the TNFR1-mediated pathways because TNFR1 is a ubiquitous membrane receptor, which has been studied intensively by numerous experimental groups. We abstained from incorporating further receptors. An extension of the model to include other relevant cell death-inducing receptors, as, e.g., the FAS receptor, would significantly increase the complexity of the model but is a worthwhile task for future work. Here, we developed a semi-quantitative PN model and applied invariant-based methods and *in silico* knockout analysis to investigate and discuss the system’s behavior of the PN. This includes a detailed discussion of the molecular switch behavior in the TNFR1-induced signaling pathway.

## Results

### Compilation of literature-based knowledge

It is crucial to find the suitable level of abstraction and scope of model with regard to the available knowledge of the signaling pathway and the questions to be addressed. Significant aspects are the availability, amount, quality, and reliability of experimental findings. For the TNFR1 signaling system, kinetic data are scarce and do not cover the entire scope of processes. We cannot build a fully quantitative model because the required knowledge of stoichiometry, concentration, and other kinetic parameters is lacking for many processes. Note that, experimental investigation of detailed kinetics under relevant *in vivo* conditions is difficult. The lack of kinetic parameters motivated us to work at a higher level of abstraction. We chose the Petri net formalism [31,32] as the appropriate modeling framework because PNs provide a hierarchy of levels of abstraction, a broad variety of rigorous methods for analysis and simulation, and a convenient visualization of the model and of the analysis results. The initial challenge of the modeling process was the construction of the model, which demanded for an unbiased manual curation of available information in the literature. We did not apply text-mining approaches but fully manually researched the literature, starting with review papers. Whereas for some processes of the TNFR1 pathway, information and experimental findings were uniquely reported, for other processes, the interpretations of experiments were contradictorily discussed. In these cases, we discussed the literature with our experimentally working coauthors and chose an appropriate abstraction. In the initial step, we compiled all processes into a graphical representation using Inkscape [62], see Fig 1. This graphic was the starting point for the construction of the PN. For every reaction (transition) of the model, S1 and S2 Tables give the name, the biological process, the corresponding literature reference, and, if available, the organism and/or cell line. The majority of reactions was measured in mammals (human and mice).

Information on the stoichiometry was lacking for the majority of reactions. We abstained to speculate about reaction kinetics, as, e.g., the Michalis-Menten kinetics, that might be reasonable for some of the reactions. Instead, we abstracted processes by a single transition if not more detailed information was available. The PN model might ignore the detailed kinetics of many reactions. Note that, a detailed kinetic model of the entire TFNR1 pathway is currently out of the scope of any theoretical approach. Moreover, detailed kinetics is mostly available for specific cell lines and specific organisms. The specificity for cell line and organism reduces the relevant experimental data that can be integrated into a kinetic model. The PN approach enables the further development of a model of high abstraction level to a detailed kinetic model if the relevant information becomes available.

**Fig 1** schematically illustrates the molecular processes of TNFR1 signal transduction. This pathway is initiated by the stimulation of the TNF receptor followed by the formation of complex I and a diversity of consecutive and concurrent molecular processes. An example is the translocation of NF-κB into the nucleus, which facilitates gene expression activity and transcription of proteins like IκB, A20, cellular FLICE-inhibitory protein (cFLIPL), B-cell lymphoma 2 (BCL-2), and X-linked inhibitor of apoptosis protein (XIAP). The transcription of these proteins affects, e.g., the regulation of the TNFR1 signaling pathway. The formation of complex IIa, complex IIb, and the necrosome may induce either apoptosis or necroptosis.

### The Petri net model of signaling processes of cell survival, apoptosis, and necroptosis

**Fig 2:**
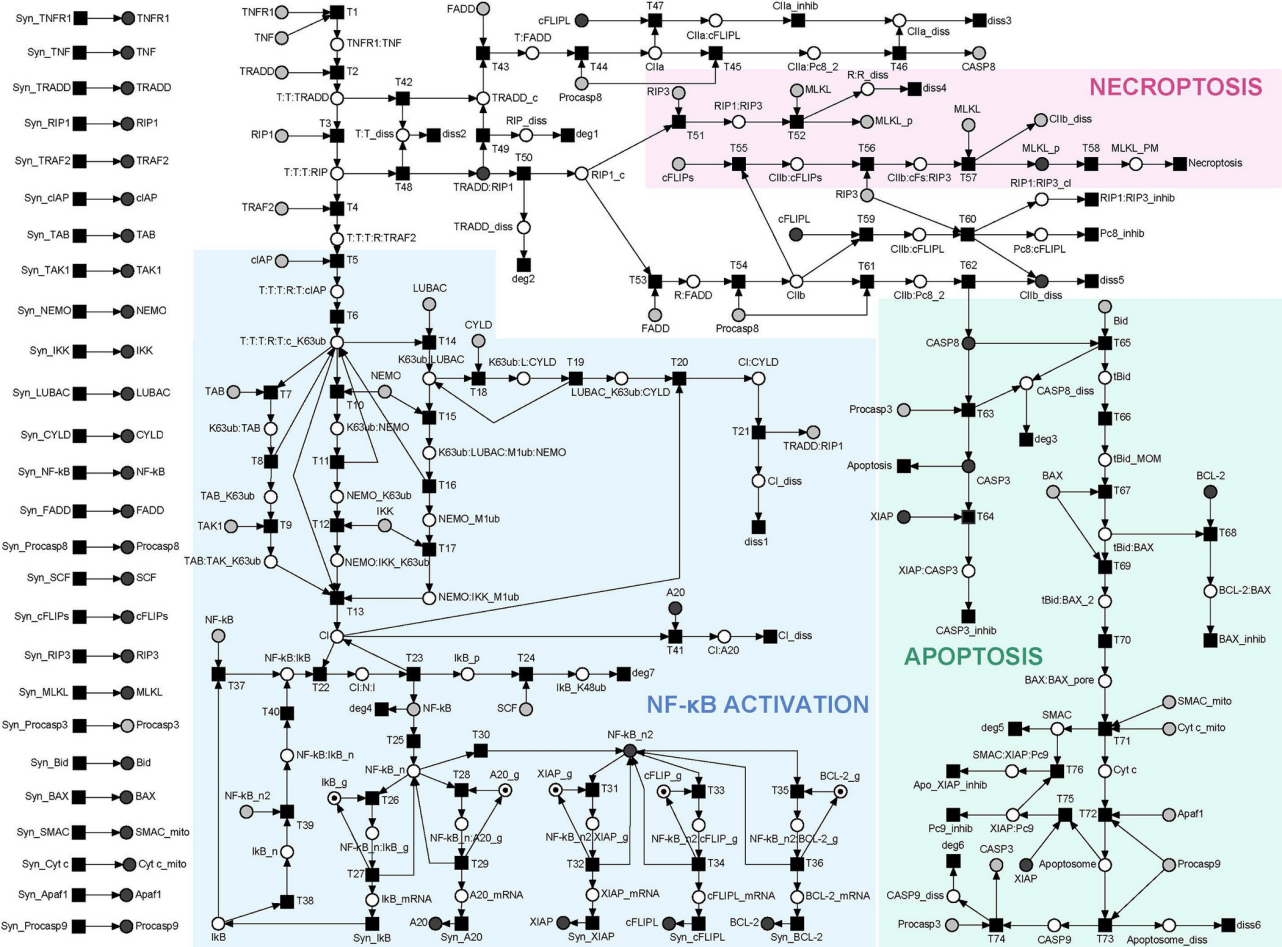
The PN model of TNFR1 signal transduction. The PN consists of 118 places drawn as white or gray circles, 130 transitions drawn as black squares, and 299 directed edges. Logical places were colored gray, describing vertices with equal names that represent one vertex in the underlying data structure of the PN. The essential processes of NF-κB activation, apoptosis, and necroptosis were shaded blue, green, and red, respectively. The initial marking was represented by one token (black dot) assigned to the places *IκB_g*, *A20_g*, *XIAP_g*, *cFLIP_g*, and *BCL-2_g* (*g* stands for gene) for each place invariant (PI).

In the following, we refer to the PN terminology, which we explain in detail in section “*Materials and Methods*”. Based on the processes illustrated in **Fig 1**, we constructed a PN model to analyze the broad combinatorial spectra of signaling pathways. The model comprises stoichiometry relations for well-studied processes in combination with the abstraction of a simple transition for processes with unknown stoichiometry or controversial experimental findings. **Fig 2** represents the PN model with 118 places, 130 transitions, and 299 edges. For a SBML version of the Petri net, we refer to S1 Data. For the list of transitions, places, and label abbreviations, we refer to **S1–S4 Tables**, respectively. In **Fig 2**, signal cascades towards NF-κB activation, apoptosis, and necroptosis are highlighted blue, green, and red, respectively. A dot in a circle indicates a place with one token in the initial marking. Gray circles represent *logical places*, which appear at several locations of the network layout and represent one unique place of the same name in the PN. On the left side, the layout separately shows the synthesis of 26 housekeeping proteins that are required for maintaining the basic cellular function. The input and output transitions were labeled according to their biological meaning. All other transitions were consecutively numbered. Input transitions (squares without outgoing edges) represent syntheses of proteins. Output transitions (squares without incoming edges) model the diverse cellular outcomes, like apoptosis, necroptosis, or survival as well as degradation and dissociation processes for proteins and protein complexes, respectively. The places were labeled according to the biological meaning, e.g., by the names of a protein, a modified protein, or a protein complex. To ensure correctness and completeness of the model to the greatest possible extent, we applied the invariant analysis.

### Place invariants reflect substance conservation

The five place invariants (PIs) of the PN, all containing two places, represented the conservation of the proteins IκB, A20, XIAP, cFLIPL, and BCL-2, see **Table 1**.

**Table 1:** The place invariants with their places and the biological meaning.

PI	Places	Biological meaning
1	*IkB_g*, *NF-kB*:*IkB_g*	Gene expression of IκB
2	*A20_g*, *NF-kB*:*A20_g*	Gene expression of A20
3	*XIAP_g*, *NF-kB*:*XIAP_g*	Gene expression of XIAP
4	*cFLIPL_g*, *NF-kB*:*cFLIPL_g*	Gene expression of cFLIPL
5	*BCL-2_g*, *NF-kB*:*BCL-2_g*	Gene expression of BCL-2

**Fig 3:**
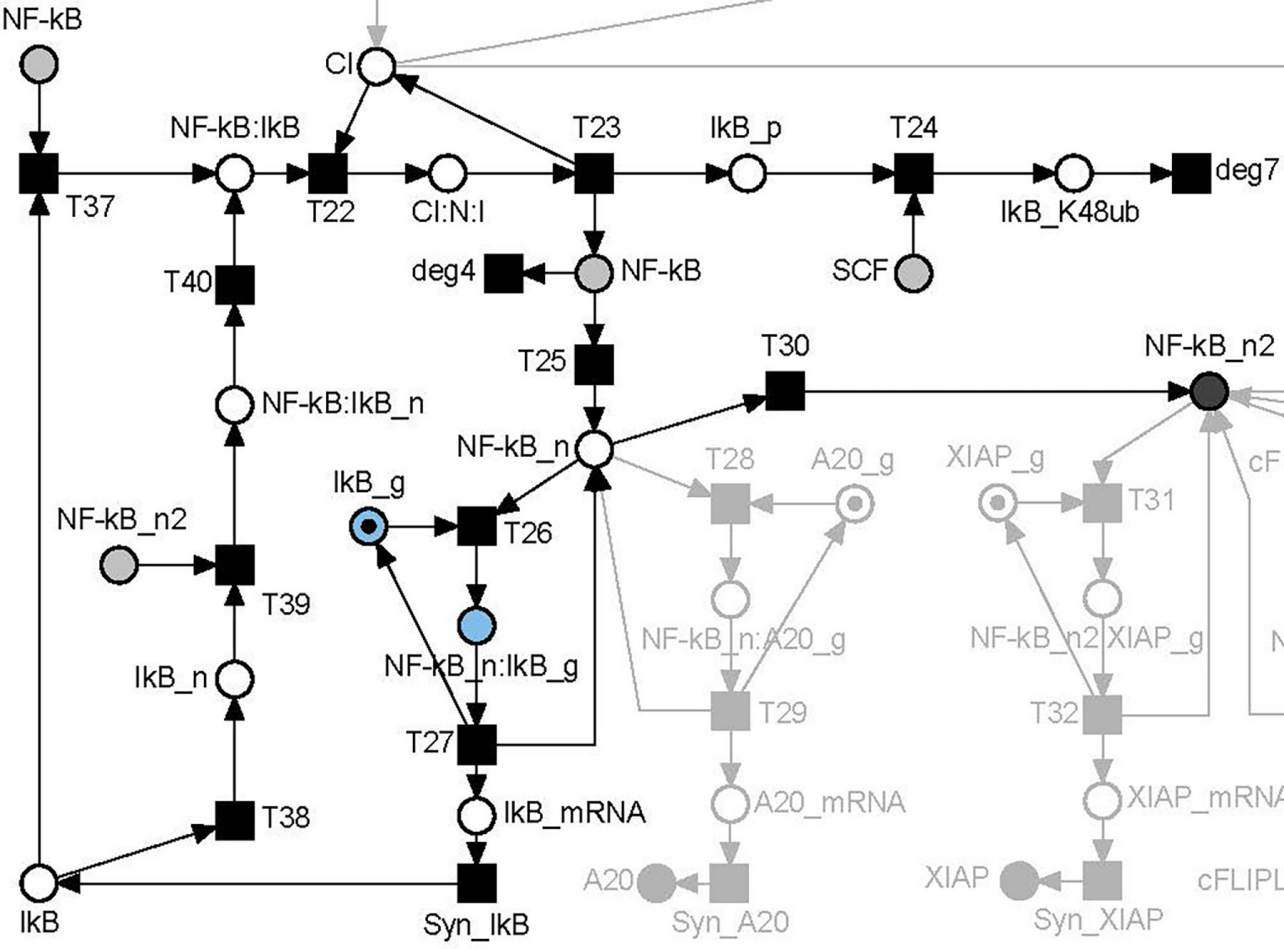
Model of the regulation of NF-κB activity. A part of the PN model in **Fig 2** was colored black and blue. Places of the place invariant PI1, *IkB_g* and *NF-kB*:*IkB_g*, were colored blue. White circles represent places and black squares transitions. A dot on a place illustrates a token, here on place *IkB_g*. The token may move to place *NF-kB_n*:*IkB_g* and back but will never vanish. The invariant PI1 reflects the conversation of the gene for the transcription of IkB.

**Fig 3** depicts the regulation of NF-B activity highlighting the place invariant PI1. PI1 describes the conservation of the gene of IκB, which was neither produced nor degraded. The total token load never changes for the two places and with regard to the initial marking, the token is either allocated to place *IkB_g* or place *NF-kB*:*IkB_g*. In the initial marking, a token was assigned to place *IkB_g*, which represents the gene of IκB. The activated transcription factor can bind to the gene (transition *T26*) and induces the transcription of the mRNA. Following the transcription, the gene as well as the transcription factor dissociated, and both were regained (transition *T27*). The other PIs of the PN, PI2, PI3, PI4, PI5, featured a similar regulatory motif.

### Transition invariants reflect basic dynamic patterns

The examination of transition invariants (TIs) is an important analysis for the verification of the PN since they reflect basic dynamic patterns. The TNFR1 PN was covered by 48 TIs, wherein each transition was part of a TI. All 48 TIs represented reasonable signal flows, see **S5 Table**. All TIs contained input and output transitions. Only TI_18_ was a trivial TI, which described the synthesis and degradation of NF-κB. 33 of the 48 TIs represented *incomplete* signaling pathways, so-called *dissected pathway*s. Dissected pathways do not cover a pathway from receptor activation to cell response. **S5 Table** indicated dissected pathways by TI numbers in bold face. **S1** (TI_15_) and **S2** (TI_9_) **Figs** show examples of dissected pathways. A dissected pathway ignores interrelation with other pathways, see, e.g., the missing activation of NF-κB that is necessary to induce the A20 feedback regulation of TI_9_. For the list of TIs and their biological interpretations, we refer to **S5 Table**. The remaining 15 TIs were also Manatee invariants (MIs) that describe complete signaling pathways, i.e., from the receptor activation to the cell response [63], see section “*Materials and Methods*”. For the verification of a biological PN, we postulated as important criterion that every TI should be biologically meaningful.

**Fig 4:**
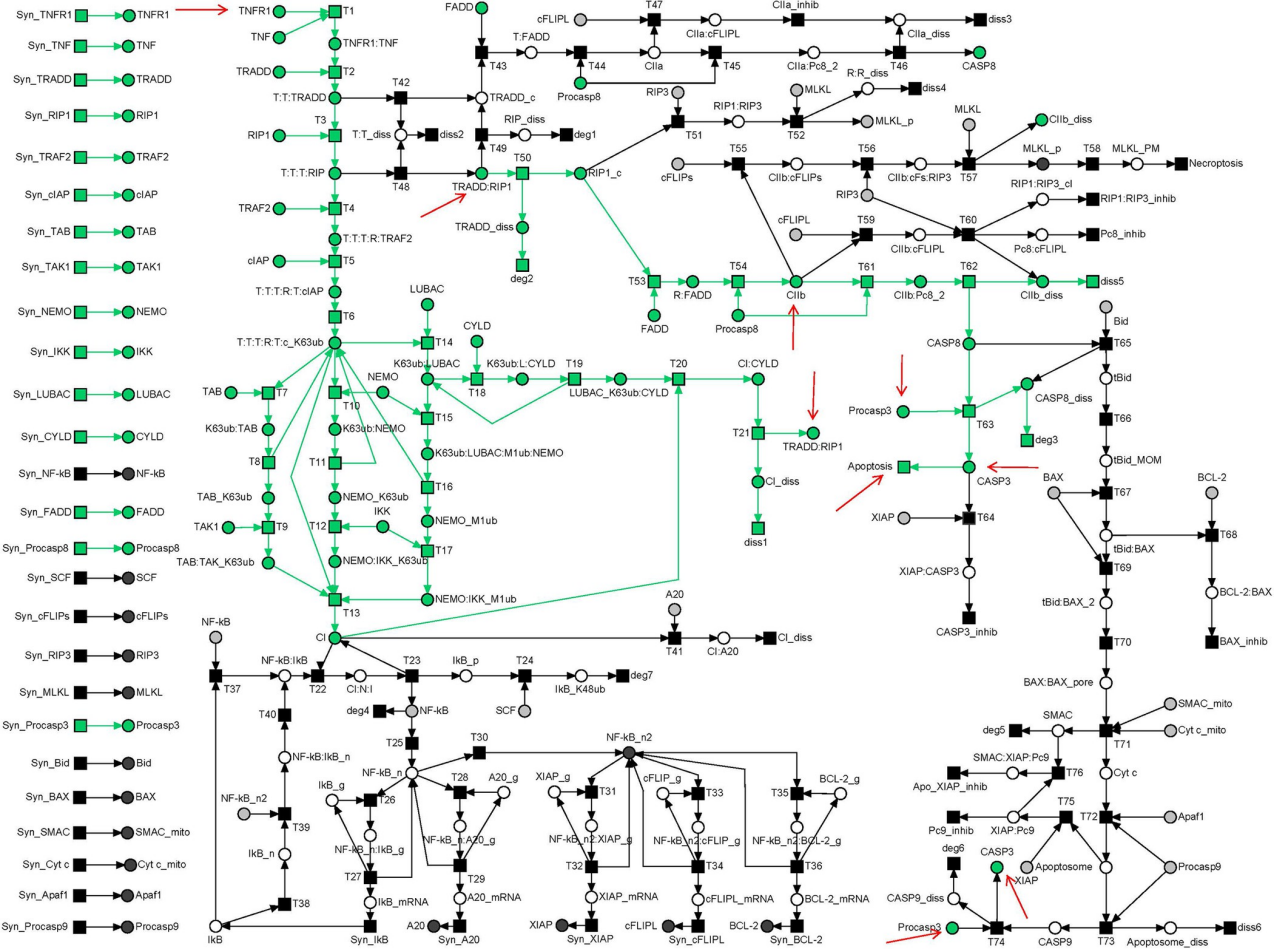
The TI_2_-induced subnetwork. The TI_2_-induced subnetwork was highlighted green in the PN model of **Fig 2**. The subnetwork covers the formation of complex IIb (place *CIIb* indicated by a red arrow) and induction of apoptosis (transition *Apoptosis* indicated by a red arrow) via the activation of CASP3 (places *CASP3* as logical places indicated by a red arrows) in the extrinsic pathway. Additional logical places that connected graphical subnetworks were *Procaspase3* and *TRADD*:*RIP1*, each marked by a red arrow.

Exemplarily, TI_2_ colored green in **Fig 4** comprised the formation of complex I, along with its ubiquitination with K63 and M1 Ub chains, the dissociation of complex I by ubiquitination via CYLD recruited to LUBAC, the formation of complex IIb, and extrinsic activation of caspase 3, which induces apoptosis. TI_2_ described a reasonable signal flow in the PN and hence corresponded to a possible pathway mediated by TNFR1 signal transduction.

### Manatee invariants describe complete signaling pathways from receptor activation to cell response

**Fig 5:**
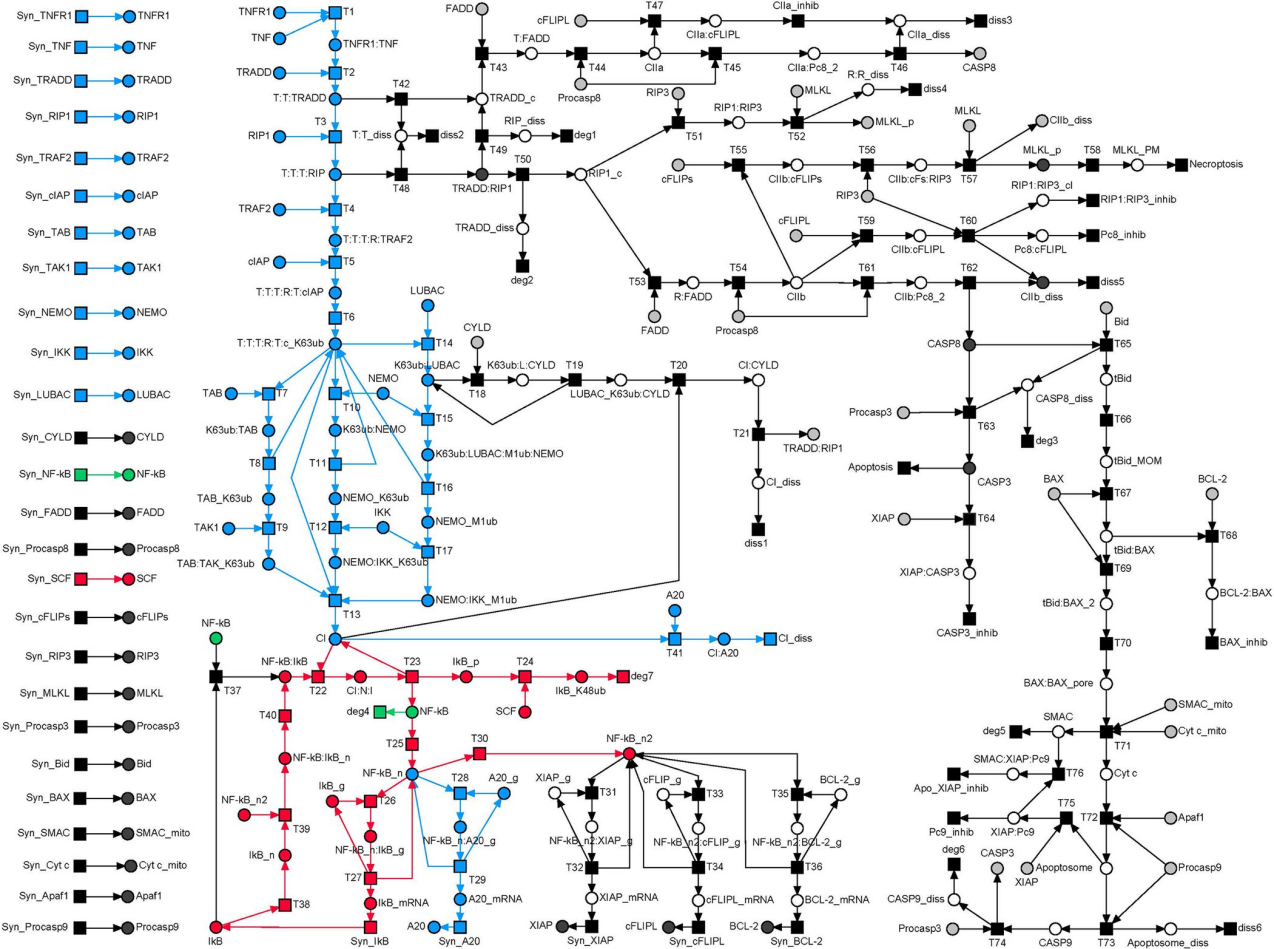
The MI_7_-induced subnetwork of the PN model in Fig 2. The MI_7_-induced subnetwork consists of TI_9_ in blue, TI_15_ in red, and TI_18_ in green.

Overall, we found 279 MIs, see **S6 Table**. Each of the 279 MIs represented a unique pathway of the molecular switch between cell survival, apoptosis, and necroptosis. Exemplarily, **Fig 5** highlights MI_7_ that combined three TIs, TI_9_ colored blue, TI_15_ colored red, and TI_18_ colored green. MI_7_ exemplified typical mutual dependencies of TIs that make isolated TIs nonfunctional. The red signal flow described by TI_15_ required NF-κB, i.e., a token on place *NF-κB*, as well as a token on place *CI* (complex I). NF-κB was provided by transition *Syn_NF-κB* of the green TI_18_. Complex I was provided by transition *T13* of the blue TI_9_. Vice versa, the signal flow described by the blue TI_9_ cannot work without NF-κB in the nucleus, i.e., a token on place *NF-κB_n*. Translocation of NF-κB into the nucleus required an active transition T25 of the red invariant TI_15_.

TI_9_ **S2 Fig** describes the dissected pathway of the A20 feedback regulation in complex I. MI_7,_ see **Fig 5**, which includes TI_9_ colored blue, represents a complete signal flow. MI_7_ includes the A20 feedback loop and covers the signal flow of complex I formation and activation of NF-κB with its translocation into the nucleus and gene expression of IκB and A20. The inhibitor, IκB, terminates gene expression and restores the inhibitory complex of NF-κB and IκB in the cytosol. A20 binds to complex I, leads to the dissociation of the complex, and prevents the formation of complex II. For other MIs that contain TI_9_, see S3 and S4 **Figs**.

### Classification of MI-defined signaling pathways

Each of the 279 MIs denoted a complete and unique signaling pathway, see **S6 Table**. For space reasons, we abstained from a discussion of each individual pathway. We classified the MIs according to their biological outcome. We assigned 166 MI-induced subnetworks to unique cell response, either survival, apoptosis, or necroptosis. We assigned 65 MIs to multiple cell responses and called them ambiguous pathways. An ambiguous pathway covers, e.g., the inhibition of MOMP induction, which would result in cell survival and apoptosis induction via the extrinsic pathway. In this special case, MOMP induction is part of the intrinsic pathway, but extrinsic apoptosis induction is still possible. Thus, the MOMP induction would be classified as an apoptosis pathway. This was true for 48 MIs of the 113 MIs, so they were all considered for the classification, overall 214 (48 + 166) MIs.

**Fig 6:**
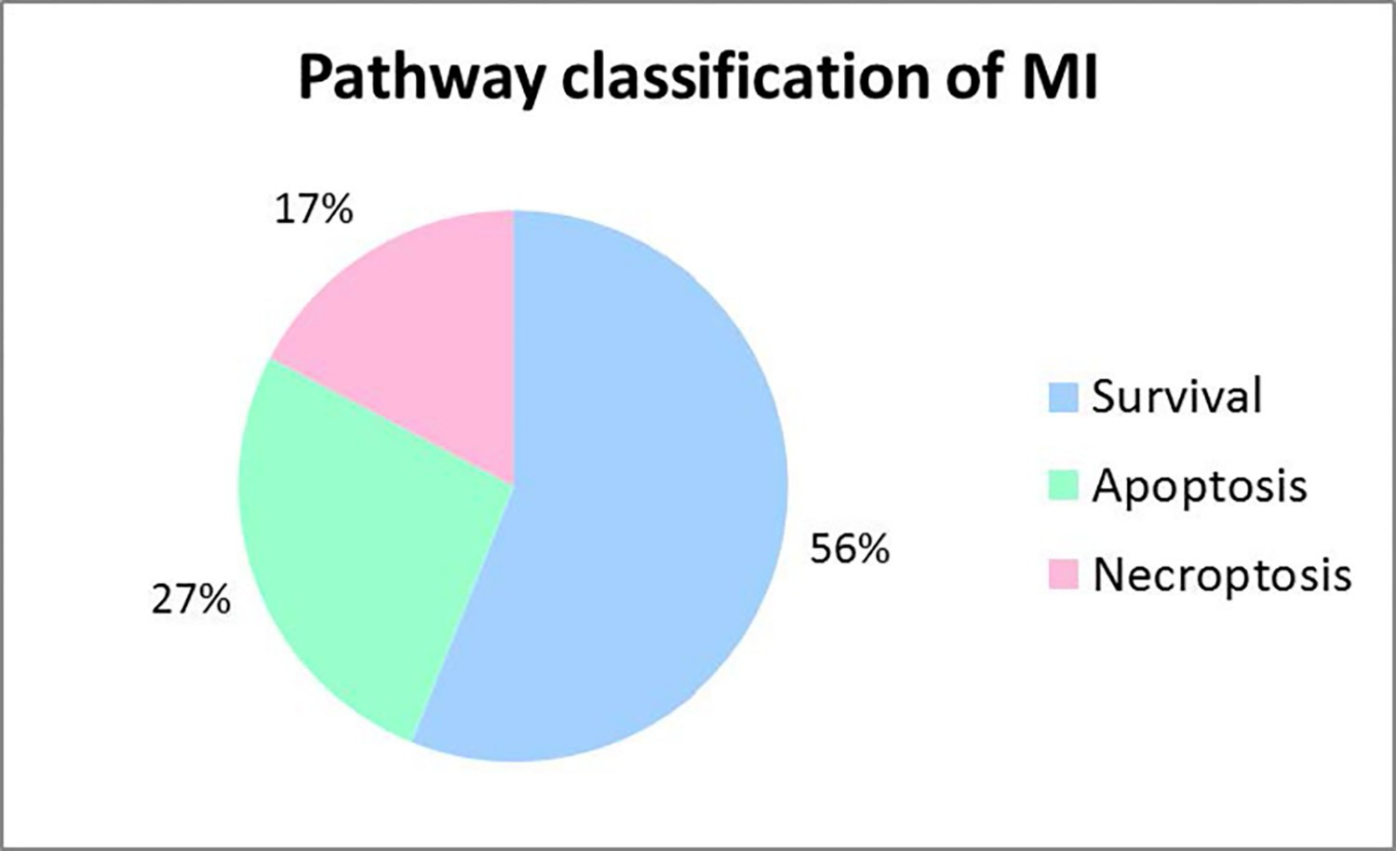
Pathway classification of MIs. Pie chart of the classification of MIs according to survival, apoptosis, and necroptosis pathways. Ambiguous MIs were neglected in the chart. In total, 214 MIs were taken into account.

The largest fraction of 56% of the pathways steered the cell to survival, whereas 27% and 17% of the pathways led to apoptosis and necroptosis, respectively, see **Fig 6**. We neglected the 65 ambiguous pathways because they either could trigger both types of cell death, apoptosis and necroptosis, or represent housekeeping pathways without induction of a specific cellular response. A simple example of a housekeeping pathway is the synthesis and degradation of NF-κB described by TI_18_ colored green in **Fig 5**. Note that, TI_18_ also corresponds to MI_18_. For pathways that can trigger both types of cell death, accurate quantitative simulations would be required to determine the stochastic chance of the cell to end up either in apoptosis or necroptosis.

### *In silico* knockouts

**Fig 7:**
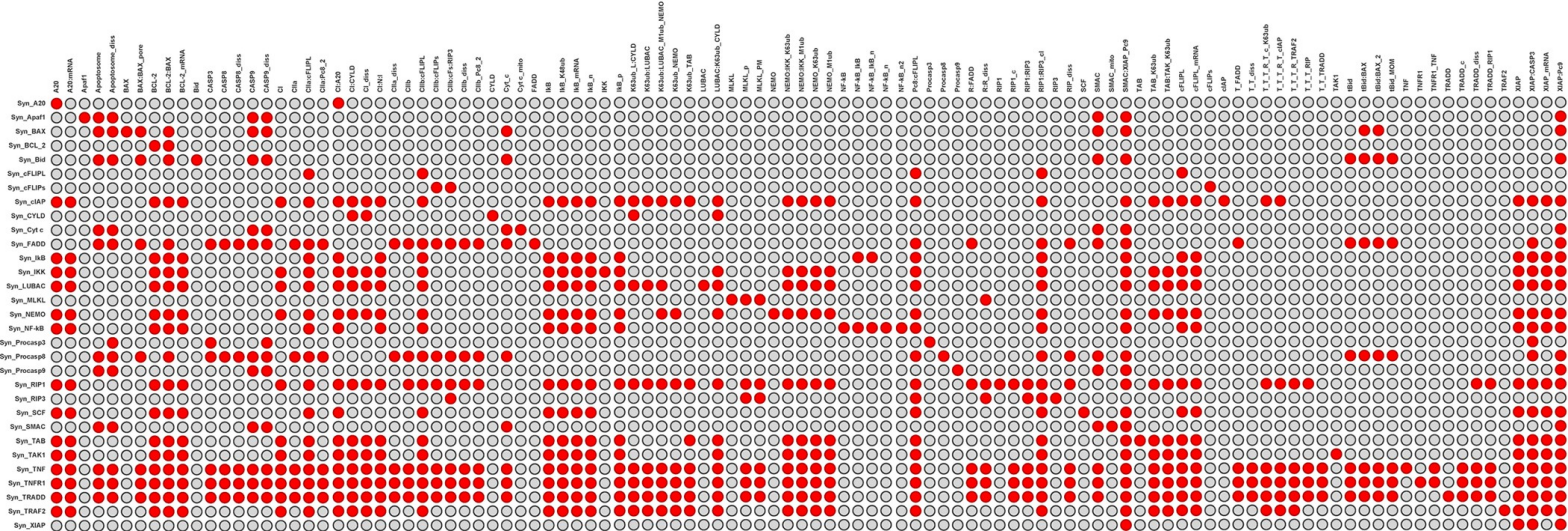
Complete knockout matrix the PN of TNFR1 signal transduction in Fig 2. The color-coded effects of the knockout of all syntheses of proteins (31 transitions) are displayed for all places of the PN except for the places belonging to a PI (108 places). Red denotes affected and gray non-affected places.

The knowledge of the combinatorial diversity of pathways enabled us to estimate the vulnerability of the system to perturbations, caused, for example, by knockouts of proteins. We examined the PN for its robustness properties and vulnerability, applying knockout studies on MIs. In the *in silico* knockout analysis we wanted to get the number of blocked molecular species downstream of the pathways [64]. We performed the *in silico* knockout analysis for all proteins and the complete set of proteins and protein complexes. We selected all transitions, which represent protein syntheses, and all places of the PN model except for the places of a PI. In total, we considered 31 transitions and 108 places in the knockout matrix illustrated in **Fig 7**. The knockout was performed applying the software tool isiKnock [64] based on MIs (fast search).

**Fig 8:**
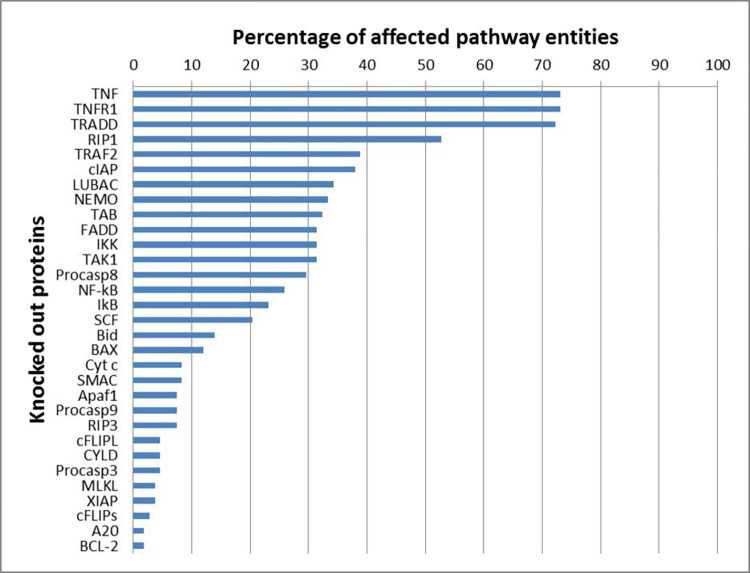
Ranking of proteins of the TNFR1 signaling pathway. The bar chart displays the percentage of affected MIs for the knocked out proteins, see **Fig 7**.

All protein knockouts affected at least one pathway component, which emphasized that all proteins have a specific function in the network. **Fig 8** shows a bar plot of the percentage of the network that becomes inoperable, if we would knockout the synthesis of a specific protein. We ranked the proteins according to the percentage of affected pathway entities. The dissection of the hierarchy of a pathway is important for potential application in therapeutic interventions. A protein that is a player more upstream in the pathway may have also an impact on other downstream branches in an undesired form of crosstalk. Therefore, a later intervention of the pathway is often more favorable because it acts more specifically [9]. Some proteins or complexes, which can be activated in various ways, were more robust to errors because alternative signal flows might enabled their activation. The components of the pathway that were involved in the processes of receptor stimulation and complex I formation were obviously more sensitive to perturbations than downstream branching pathways. TNF-α (called TNF in **Fig 8**) and TNFR were top-ranked as they are essential upstream in each pathway. RIP1 was an important node, as it plays key roles in NF-κB activation, apoptosis, and necroptosis. However, not all branches of the network were RIP1-dependent, like apoptosis mediated via complex IIa. Only housekeeping pathways remained unaffected. Proteins of the intrinsic apoptotic branch, necroptosis, and proteins upregulated by NF-κB had more specific functions in the molecular switch and got a lower ranking.

### The hierarchical cluster tree

**Fig 9:**
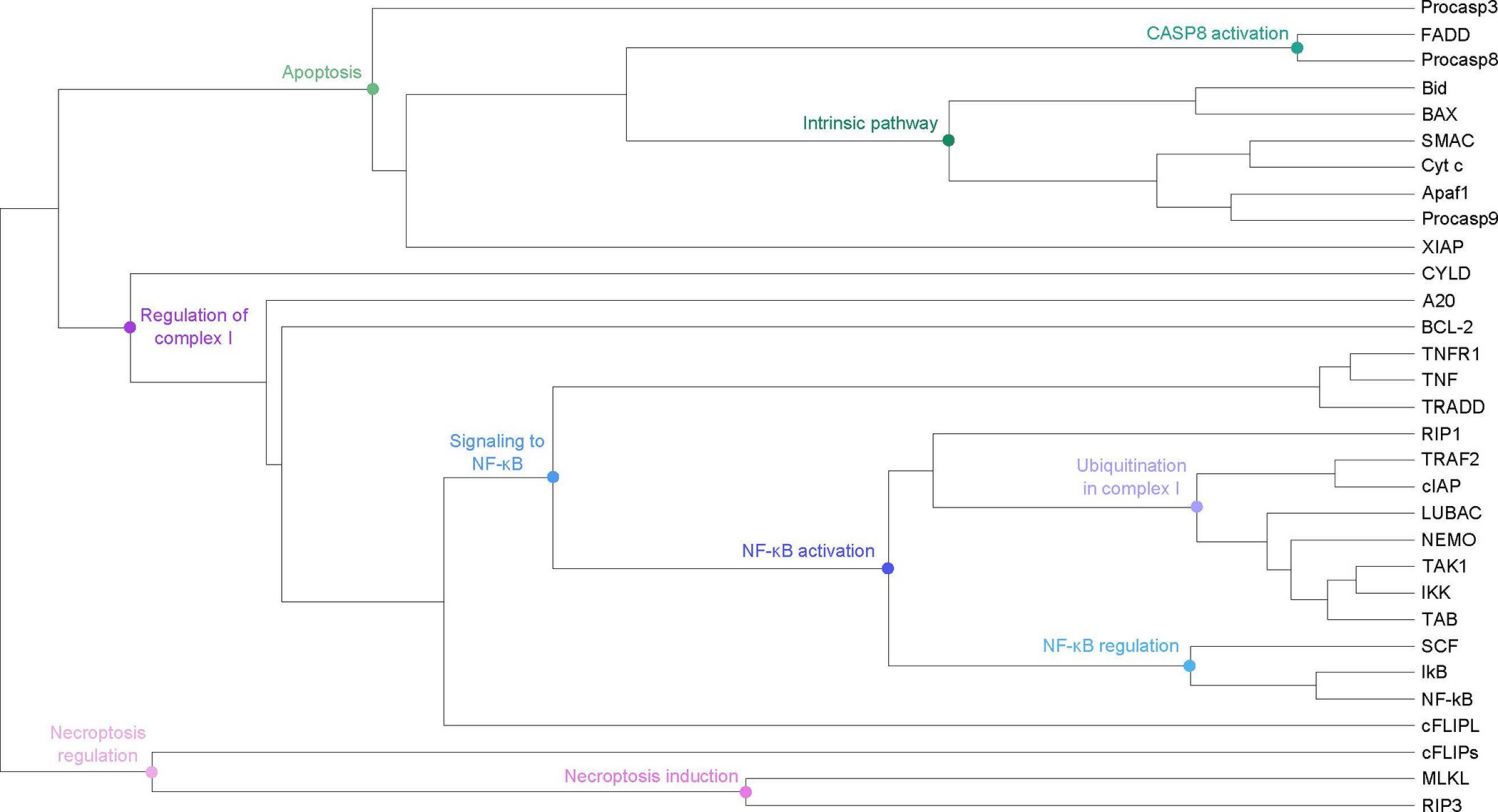
Hierarchical cluster tree. The places in the PN were clustered based on the matrix in **Fig 7**. The hierarchical clustering was performed, using the software tool NOVA [65] with UPGMA (Unweighted Pair Group Method with Arithmetic mean) [66] with Pearson correlation distance [67]. Each leaf of the tree is a protein. Some nodes in the cluster tree were marked blue, green, and red, referring to processes of NF-κB activation, apoptosis induction, and necroptosis induction, respectively.

We performed a clustering of the knockout data of the complete in silico knockout matrix in Fig 7. For the hierarchical clustering of the matrix entries, we applied the software NOVA [65] with the settings UPGMA [66] with Pearson correlation distance [67]. Fig 9 depicts the resulting hierarchical cluster tree. The cluster tree simplifies the interpretation of the knockout results for the complete in silico knockout matrix in Fig 7. Each leaf of the tree is a protein. We labeled some nodes of the cluster tree according to a relevant biological process. To cluster the proteins, we represented a protein by the downstream effect of its knockout, i.e., the set of blocked species. We labeled specific branch points by the characteristic, regulative function of the group of proteins, e.g., ubiquitination in complex I, activation of CASP8, and activation of NF-κB.

The three major branches of the TNFR1 signaling pathway are illustrated in Fig 9. The green color marks the nodes associated to processes of apoptosis induction, the blue and purple colors label the nodes associated to processes of the signaling to NF-κB, and the red color marks the nodes associated to necroptosis initiation. Proteins involved in similar processes were correctly clustered together, as the regulation via ubiquitination in complex I, the activation of CASP8, and the regulation of NF-κB activity. Due to crosstalk and feedback, the regulation of complex I was more strongly coupled to apoptosis than to necrosis, leading to a merging of the two branches Regulation of complex I and Apoptosis. The necroptosis branch was separately clustered because the regulation of complex I has a stronger link to the apoptosis pathway via crosstalk and feedback mechanisms derived from NF-κB-mediated gene expression. The three proteins of the necroptosis branch, RIP3, MLKL, and cFLIPs, were grouped together very late. Large clusters for the activation of NF-κB or the intrinsic pathway of apoptosis were already merged before RIP3 and MLKL clustered together. The necroptosis branch remained separated from all other functions until the very last clustering step.

### Knockout analysis of a selected submatrix

**Fig 10:**
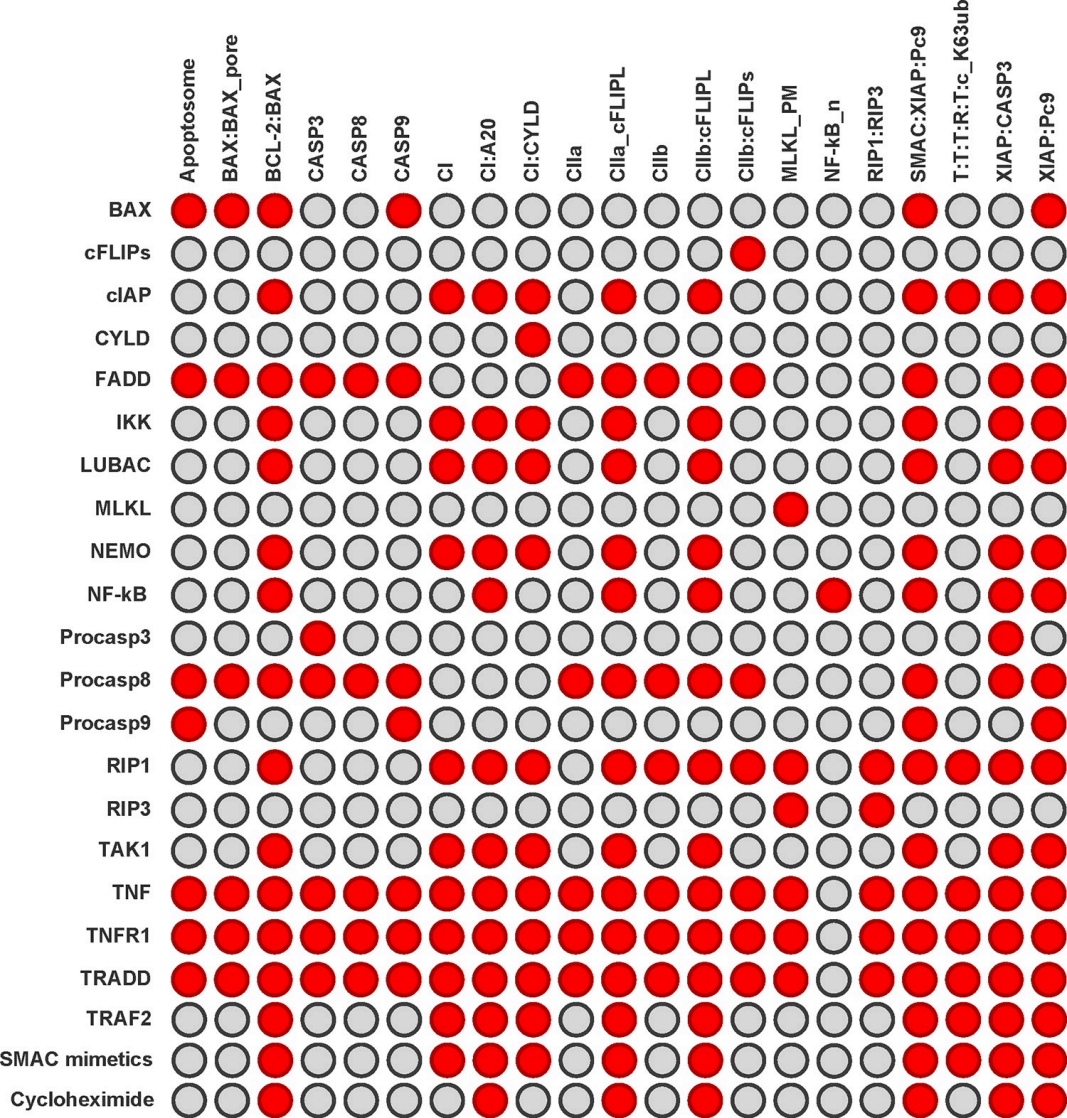
*In silico* knockout submatrix of the PN in Fig 2. The rows list the proteins, which were knocked out, and the columns give the protein complexes in the network, which could be affected by the knockout. A red (gray) entry indicates that the respective complex was (was not) affected. We performed a single knockout analysis for twenty proteins and displayed the effect for a part of 21 pathway entities. The last two rows represent multiple knockouts and display the effect of SMAC mimetic, i.e., the knockout of XIAP and cIAP, and the impairment of the translation of upregulated genes by cycloheximide, i.e., the knockout of IκB, A20, XIAP, cFLIP_L_, and BCL-2.

We employed the *in silico* knockout for an additional verification of the PN model and for a discussion of the molecular switch behavior. Whereas some knockout effects were obvious, others can only be derived from network analysis. In the following, we discuss exemplary knockouts in detail. **Fig 10** shows a subsection of the *in silico* knockout matrix in **Fig 7**. We selected 20 proteins for single knockout and determined the effects for 21 pathway entities**, see S7 Table**. The additional two rows in **Fig 10** represent the effect of the multiple knockouts for SMAC mimetic and the impairment of translation by cycloheximide. The selection should allow to deduce the impact of the proteins on the molecular switching behavior. Therefore, we examined the effect of the *in silico* knockouts on selected pathway entities, which represent important signaling nodes and cover all pathways, including complex I (CI), complex IIa (CIIa), complex IIb (CIIb), apoptosome, and necrosome (RIP1:RIP3). To perform a detailed analysis for the knockout of all proteins in all network components is out of the scope of the paper.

In the following, we explicitly describe the results of *in silico* single and multiple knockouts of the submatrix in **Fig 10** summarized in **S7 Table**.

**Knockout of BAX (row 1)**: There were six red entries. Obviously, the knockout of BAX had a negative effect on the complex formation of BCL-2 and BAX (*BCL-2*:*BAX*), but an impact on the activation of CASP9 in the apoptosome (*CASP9*, *Apoptosome*). The activation of CASP3 and CASP8 were affected *(CASP3*, *CASP8*).

**Knockout of cFLIPS (row 2):** We observed only one red entry for the complex of cFLIPS bound to complex IIb (*CIIb*:*cFLIPs*). cFLIPS can promote necroptosis induction in complex IIb. Since other pathways exist that can induce necroptosis, the knockout of cFLIPS had no direct effect on necroptosis induction.

**Knockout of cIAP1/2, TRAF2 (rows 3, 20)**: Both rows had the same ten red entries affecting the formation of complex I and the NF-κB-dependent gene expression as well as the feedback and crosstalk regulation of the target genes. This emphasized the direct regulation of both proteins since cIAP1/2 requires TRAF2 for recruitment.

**Knockout of CYLD (row 4):** There was one red entry for the complex of CYLD bound to complex I (*CI*:*CYLD*). CYLD promotes the dissociation of complex I and the formation of complex II. Since several pathways also cover downstream processes, no additional effects were observed.

**Knockout of FADD, procaspase 8 (rows 5, 12):** Both rows exhibited the same 14 red entries all associated to apoptosis processes. Only the survival pathways and necroptosis induction were still functional. This emphasized the direct regulation of both proteins since procaspase 8 requires FADD for recruitment.

**Knockout of IKK, LUBAC, NEMO, TAK1 (rows 6, 7, 9, 16):** All four rows had the same nine red entries, which indicated the strong relation of the proteins in the Ub-dependent regulation in complex I. All red entries affected the downstream activation of NF-κB and the regulation of the target genes, while complex II formation and cell death induction remained functional.

**Knockout of MLKL (row 8):** We got only one red entry for activated MLKL located at the plasma membrane prior necroptosis induction *(MLKL_PM*). Since this refers to the last step in the necroptosis pathway, necroptosis induction was hampered.

**Knockout of NF-κB (row 10):** We had eight red entries, all referring to NF-κB regulation via IκB and the regulation of NF-κB-dependent genes.

**Knockout of procaspase 3 (row 11):** We observed two red entries. The knockout affected CASP3 activation and CASP3 inhibition by XIAP (*CASP3*, *XIAP*:*CASP3*).

**Knockout of procaspase 9 (row 13):** We had four red entries, all referring to the processes of the regulation of procaspase 9 in the apoptosome via XIAP and SMAC.

**Knockout of RIP1 (row 14):** We got 14 red entries, affecting the formation of complex I and the induction of necroptosis. Only apoptosis processes were still functioning since RIP1 is a major player in the TNFR1 signal transduction pathway.

**Knockout of RIP3 (row 15):** We had two red entries. The formation of the necrosome and the activation of MLKL were affected by the knockout of RIP3 (*RIP1*:*RIP3*, *MLKL_PM*).

**Knockout of TNF, TNFR1, TRADD (rows 17, 18, 19):** All three rows showed the same 20 red entries, affecting all places except the nuclear NF-κB (*NF-kB_n*), due to the turnover of NF-κB, which remains unaffected by the knockout. Since the three proteins initialized the pathway, all downstream pathway components were affected by the knockouts.

**Effect of SMAC mimetic by multiple knockout of cIAP1/2 and XIAP (row 21):** We had ten red entries, all referring to the formation of complex I, NF-κB-dependent gene expression and XIAP regulation. Only apoptosis and necroptosis induction remained functional.

**Effect of cycloheximide by multiple knockout of IκB, A20, XIAP, cFLIPL, and BCL-2 (row 22):** We got seven red entries, mimicking the effect of cycloheximide, which impaired the translation of upregulated genes. This predicted that only the cell death pathways remain unaffected. Upon TNF stimulation, most cells did not exert cell death because of rapid gene expression of cFLIPL, cIAP2, XIAP, and BCL-2, which may inhibit cell death signaling [68]. The treatment with cycloheximide, an inhibitor of translation, or actinomycin D, an inhibitor of transcription, resulted in enhanced cell death [69].

## Discussion

### The Petri net model

The study of TNFR1 signal transduction has a long history and revealed many theories. Each theory has its own the individual focus and may reflect alternative viewpoints [10,70]. Contradictory results in literature and variations in signal transduction, occurring, for example, in different cell types, require a disentangled view of the involved interplay of complex molecular processes [71].

Incompleteness and diversity of the data, limited knowledge, and uncertainties in the literature are general challenges for all modeling techniques. Therefore, we used experimentally determined data published in peer-reviewed and high-ranking journals for model creation. We started with review papers. Because of the expertise of physicians in the group, we were mainly interested in mammalian processes. We did not perform any wet-lab experiments, but discussed contradicting findings in the literature with experimentalists, exhibiting expertise in the relevant topic. The application of techniques to verify theoretical consistency of the model and to check the biological interpretation for correctness was mandatory to get confidence into the model.

The PN covers signaling processes of cell survival, apoptosis, and necroptosis. The PN model compiled the current view of the TNFR1 signaling pathway. During the development of the model, well-established views of molecular regulations had been superseded by other proposed regulatory mechanisms. An example is the regulation of A20, which operates as a deubiquitinating enzyme in the feedback regulation of NF-κB signaling. Originally, its suppressive role in NF-κB signaling has been assigned to the proteasomal degradation of RIP1 by a K48-linked Ub tag [72]. Recently, this assignment has been questioned even though the functional role of A20 in a feedback mechanism has long been viewed as important to terminate signal transduction.

On the contrary, less-understood processes could not be integrated in the PN model since the exact mechanism of regulation was not entirely characterized. Important aspects that need further investigation are, for example, the effect of RIP1 phosphorylation and the regulation by ubiquitination within complex II. Further, the exact mechanism of necroptosis execution and the mode of action of MLKL remains to be identified. Further experimental efforts are necessary to get a clearer picture of the TNFR1-mediated signaling pathways and to provide information to support a sound theoretical investigation.

### Model analysis

To investigate networks of pathways in systems biology profoundly, the determination of all possible signal flows was obligatory. The mathematical approach of place invariants (PIs) and transition invariants (TIs) explained substance conservation and the basic system’s behavior, respectively. TI-induced subnetworks represented functional modules. Manatee invariants (MIs) constructed by linear combination of TIs described complete functional signal flows in a network that operates at steady state. The complexity of the computation of MIs was related to the number of TIs and the possible linear combinations. For the PN of TNFR1 signal transduction, the number of MIs highly increased with regard to the number of TIs, from 48 TIs to 279 MIs. The analysis revealed substance conservation, basic dynamics of the system, and all complete signaling pathways.

**Knockout analysis:** Knockout analysis was applied for classification of pathways, ranking of pathway’s entities, and clustering of processes. The deduction of the regulation of signal transduction via knockout experiments was not an easy task since the pathway components were involved in several processes. Further, the variation of results between cell types, type of experiment, and working group, had an essential influence on the diversity of the data. The *in silico* knockout analysis could reveal obvious relations, expected dependencies, and predictions of effects that were not yet experimentally proven. Not in every case, the results of the knockout prediction did match the experimental knockouts. On the one hand, the TI or MI analysis may not capture all relevant pathway dependencies due to an insufficiently detailed modeling of the processes, or the knockout behavior is dependent on other signal flows, too, which were not explicitly included in the PN model. On the other hand, the experiments may be obtained for a specific cell type and may not be applicable to all cells. For such cases, we suggest to adapt the PN model of TNFR1 signal transduction to a specific cell type.

**Pathway classification:** The result was in accordance with the expected biological behavior because most cells exhibited a robust survival response and suppressed the cell death induction [68]. The dissection of the hierarchy of a pathway was important for later use in therapeutic implication. A protein that was a player upstream in the pathway may had also an impact on other downstream branches in an undesired form of crosstalk. Therefore, a later intervention of the pathway was often more favorable because it acts more specifically [9]. Some proteins or complexes, which could be activated in different ways, were more robust to errors since alternative signal flows could still lead to their activation. The components of the pathway that were involved in the processes of receptor stimulation and complex I formation, were obviously more sensitive to perturbations since many downstream-branching pathways were dependent on the initialization. Therefore, TNFR1, TNF-α, and TRADD were the proteins with the highest influence on other network components. Hereafter, the proteins of complex I with RIP1 were leading the way. RIP1 was an important protein since it played key roles in NF-κB activation, apoptosis, and necroptosis. However, not all branches of the network were RIP1-dependent, like apoptosis mediated via complex IIa. The proteins of complex I had a higher impact, too, because they had an influence on the formation of complex II, the activation of NF-κB, and subsequent gene expression. The resulting crosstalk to the cell death pathways enhanced the influence of the proteins of complex I. The proteins of the intrinsic apoptotic branch and necroptosis induction as well as the proteins, which were upregulated by NF-κB, were less essential and acted more specifically.

**Robustness** describes an inherent quality of systems and aims to maintain and ensure the correct function of a system [26]. Alternative signal flows, which target the same cellular response, enhance the robustness. The more redundant signal flows activate one cellular outcome, the more robust is the system to potential failing modes. The various signal flows to the different outcomes determined by MIs revealed the robustness of the TNFR1 signaling system. We concluded that the system is robust to perturbations and that the survival response is most likely to occur followed by apoptosis and then necroptosis, with regard to the amount of assigned pathways.

**Pharmaceutical therapies:** The TNFR1 signaling pathway will always be a target of cytoprotective or cytotoxic therapies since it controls opposing responses and has a major function in immunity and development [13,17]. The intertwined regulatory network makes it difficult to directly intervene cell death pathways in the desired way [73]. For cancer treatment, it is an important strategy to overcome the resistance to cell death by manipulation of signaling pathways. Such a strategy is based on SMAC mimetic, which inhibits IAP proteins [74]. SMAC mimetic mocks the function of SMAC and inhibits cIAPs, thus, preventing RIP1 ubiquitination and phosphorylation [68]. It intervenes the early checkpoint and leads to a decrease of Ub chains in complex I and promotes the formation of complex II, inducing RIP1 kinase-dependent cell death [15,75]. The prediction of the *in silico* knockout was in accordance with the experimental findings of SMAC-mimetic treatment. Upon TNF-α stimulation, most cells do not exert cell death because of rapid gene expression of cFLIP_L_, cIAP2, XIAP, and BCL-2, which inhibit cell death signaling [68]. The treatment with cycloheximide, an inhibitor of translation, or actinomycin D, an inhibitor of transcription, results in enhanced cell death [69].

**The molecular switch:** The determination of specific checkpoints of the system was important to intervene the signaling cascade in a desired manner. The survival response was very robust to perturbations. Therefore, we needed to determine the factors that overcome this robust response and promote cell death pathways. We determined the important checkpoints in complex I in terms of the ubiquitination within complex I and the activation of NF-κB-dependent gene expression. The impairment of ubiquitination, e.g., by SMAC mimetic or the *in silico* knockout of TRAF2 and cIAP, favored the induction of apoptosis and necroptosis. The upregulated genes by NF-κB negatively controlled cell death signaling. We showed that the impairment of NF-κB activation, e.g., by knockout of proteins of complex I like LUBAC, and the translation of upregulated genes, e.g., by simulating a cycloheximide treatment, promoted cell death induction.

Ubiquitinated RIP1 has been reported to have a scaffold function for the required kinases, TAK1 and IKK, in complex I and to promote cell survival [18]. Deubiquitinated RIP1 can form complex II and positively regulates cell death [76,77]. cIAP is important for TNFR1 signaling since the depletion abolishes the Ub decoration within the complex I [78,79]. The PN model supported this view since in absence of RIP1 only apoptosis induction can occur, and the impairment of RIP1 ubiquitination by cIAP and TRAF2 led to the formation of complex II.

Phosphorylated RIP1 has been reported to inhibit kinase-dependent induction of cell death, following TNFR1 ligation [76]. Several studies have reported either IKK or MAPKAP kinase 2 (MK2), which are activated within and downstream of the complex I, to be kinases that may phosphorylate RIP1 [76,80–82]. It has been suggested that the phosphorylation of RIP1 has affected the interaction of RIP1 with FADD and CASP8 [81,76]. For the association of the necrosome and the activation of RIP3, RIP1 kinase activity is required. The phosphorylation of RIP1 may function as a repressor of necroptosis besides of apoptosis [68]. To integrate the exact mechanism of RIP1 phosphorylation into the PN model, further experimental studies would be required.

Another checkpoint to enhance the resistance to cell death induction is the NF-κB-dependent gene expression. Only full activation of IKK leads to NF-κB activation [18,71]. It has been shown that the depletion or inhibition of IKK and NEMO affects the induction of apoptosis [81,83]. LUBAC and TAK1 inhibition also promote complex II formation [81,84,85]. This is in accordance with the *in silico* knockout predictions for IKK, NEMO, TAK1, and LUBAC because only apoptosis induction and necroptosis induction remained functional after a single knockout of either IKK, NEMO, TAK1, or LUBAC.

The level of cFLIP_L_ is regulated by NF-κB activation. cFLIP_L_ is a homolog of CASP8 and competes with CASP8 to form a heterodimer and prevents full activation of CASP8. If NF-κB activation is blocked, the level of cFLIP_L_ decreases, leading to the induction of apoptosis [86]. Other target genes of NF-κB, BCL-2 and XIAP inhibit the intrinsic apoptosis pathway and apoptosis induction by caspase inhibition [87]. Cycloheximide treatment impairs the translation of the upregulated genes. The results of the *in silico* knockout of cFLIPL, BCL-2, and XIAP was in accordance with the expected effects of the drug cycloheximide.

The checkpoints that mediate signal transduction in complex I and from complex I to complex II are well-characterized. But the exact regulation within complex II has not been entirely clarified. In complex II, the checkpoints mainly control caspase activity. TRADD needs to dissociate from complex I and binds to FADD to provide a platform for CASP8 recruitment and apoptosis induction [88]. cFLIP_L_ is usually upregulated at the time point at which complex II can has been assembled in the cytosol and inhibits caspase activation. The two isoforms of cFLIP differentially regulate the activity of complex II [79]. While cFLIP_L_, binding to CASP8 and FADD, has a survival function, blocking apoptosis and necroptosis, cFLIPS, binding to CASP8, inhibits full activation of caspase activity [89,90]. There are evidences that the formation of complex IIa and complex IIb has also several checkpoints, involving post-translational modifications. The influence of ubiquitination in complex II needs to be further studied [91]. CYLD is a substrate of CASP8, which may be involved in the regulation of the switch of complex IIa to complex IIb [92]. Also A20 has been reported to inhibit RIP3 activation by ubiquitination and to prevent necroptosis induction, which would result in another crosstalk from the target gene of NF-κB [91].

## Materials and Methods

### Petri nets

Petri nets (PNs) represent a graph theory-based mathematical formalism to model systems of concurrent processes [31,32]. PNs are widely-used in computer science for technical applications [93] and systems biology [33,35,36,38,94–96]. PNs are directed, bipartite, labeled graphs. They exhibit two type of vertices, one type for the passive elements of the system called *places* and one for the active elements called *transitions*. For biochemical systems, the places model biological entities, for example, proteins, ligands, protein complexes, genes, transcripts, metabolites, and other chemical compounds. Transitions stand for the reactions transforming one place into another, for example, chemical reactions, phosphorylation, ubiquitination, complex formation, and other. The directed edges connect only vertices of different type. Places with outgoing edges are called pre-places and places with ingoing edges post-places, with respect to the transition the edges connect with the considered place. Edges are labeled by integers.

Formally, we define a PN as a quintuple N = (*P*, *T*, *F*, *W*, *m*_0_) with:

*P* = {*p*_1_, *p*_2_, …, *p*_*m*_} is the finite set of places.

*T* = {*t*_1_, *t*_2_, …, *t*_*n*_} is the finite set of transitions.

*F* ⊆ (*P* × *T*) ∪ (*T* × *P*) is the set of flow relations or edges, resp.

*W*: *F* → N defines the edge weights.

*m*_0_: *P* → N_0_ is the initial marking.

For classical PNs called P/T nets (Place/Transition nets), the dynamics is performed by movable objects named *tokens* that are located on the places. A token represents a discrete unit of an entity, for example, one mole of a chemical compound or one molecule. A token distribution defines a system state and is given by the marking, *m*, which is a vector of the size of the set of places, |*P*|. The initial marking, *m*_0_, describes the initial state of the system before starting a simulation. The marking is illustrated by points on the corresponding places or by integer numbers.

**Fig 11:**
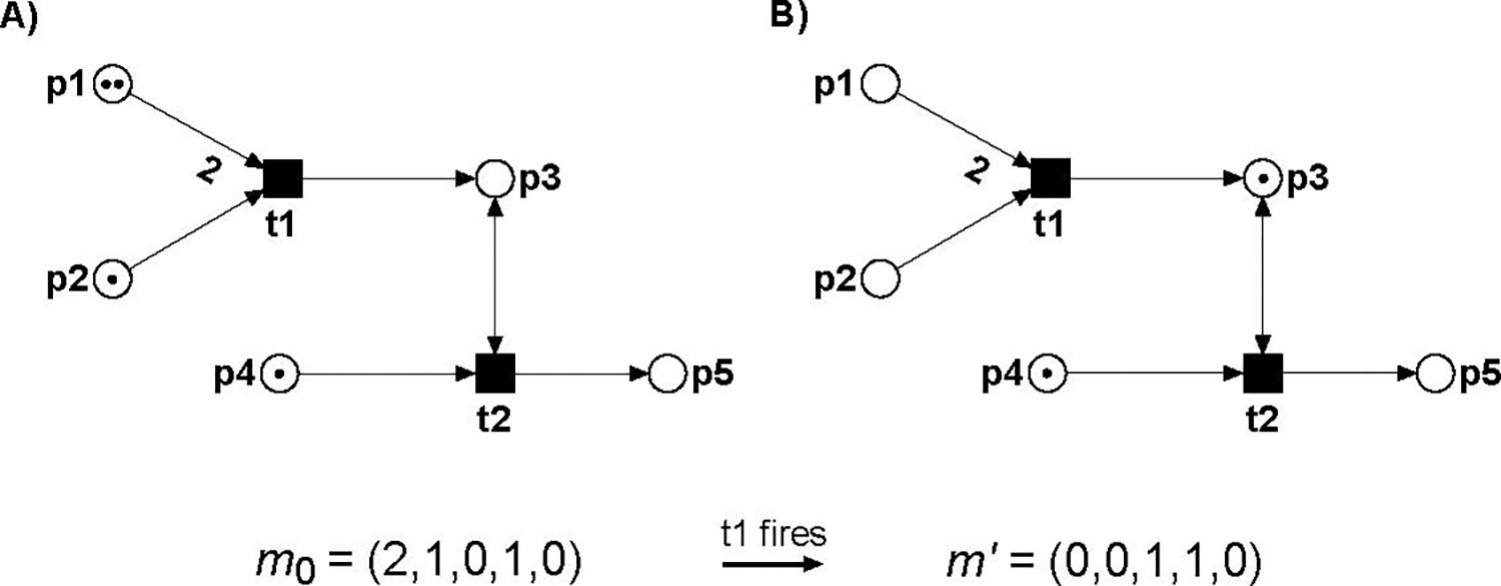
PN example for firing of a P/T net. A) The PN consists of five places depicted by circles, two transitions depicted by squares, and six directed edges. The edge from p1 to t1 has a weight of two. For all other edges with a weight equals one, no label is drawn. p3 and t2 are connected via a read edge (read arc), which is bidirectional. Tokens are depicted as dots on the places p1, p2, and p4, defining the initial marking *m*_0_ = (2, 1, 0, 1, 0). In this marking, transition t1 is *activated*. B) The PN after the firing of t1. The marking has changed by removing tokens from the pre-places, p1 and p2, and producing a token on the post-place, p3. Then, the new marking is *m*‘ = (0, 0, 1, 1, 0).

Tokens move through a PN following specific *firing rules*. In P/T nets, firing rules are timeless, meaning that the tokens on the pre-places are removed at the same time as the tokens are produced on the post-places, see **Fig 11**.

In the following, we adopt the notations for a vector *x* and a scalar *a*:

- *x* = *a* means that all components of *x* are identical to *a*,
- *x*≥*a* means that no component of *x* is less than *a* but *x* = *a* is excluded,
- and *x*>*a* means that all components of *x* are larger than *a*.

Even if not explicitly specified, all implicit relations are assumed to be satisfied. For example, for a matrix *C* and a vector *x*, a product *C x* implies that the number of columns of the matrix *C* is the same as the number of components of the vector, *x*.

We modeled the PN as an open system, meaning that all proteins of the pathway are synthesized and degraded. There are transitions without pre-places for synthesis and transitions without post-places for degradation. The only exceptions were genes that induce the synthesis of proteins in a controlled manner and, therefore, formed specific patterns in the PN model.

### Invariants

Among other properties, invariants characterize a PN. This property always holds at steady state independent of the system state and of the initial marking [97]. Invariants can relate the structure of the net to the behavior of the system and allow for predictions of the system’s dynamics.

We define *transition invariants* (TIs) and *place invariants* (PIs). Both are based on the incidence matrix of the PN, *C*: *P*×*T*, with *P* as the set of places and *T* as the set of transitions, see also the definition of PNs in the previous subsection “Petri nets”. An entry in *C* indicates the change in the number of tokens on the considered place (row), when the considered transition (column) fires. The incidence matrix describes the topology of a pure PN in a unique way. A pure PN does not contain loops, meaning bidirectional edges, between a transition and a place.

### Transition invariants

A TI is a superset of transitions, whose firing sequence reestablishes an arbitrary initial marking, Δ*m*_0_ = 0, i.e., keeps the system state invariant. A TI is defined as a Parikh vector, *x*: *T*→ℕ, of a firing sequence that fulfills the equation Δ*m* = *C x* = 0. The number of firings per transition is given by the elements of *x*. An integer solution *x* is a true invariant if it has no negative components, i.e., *x*≥0. The set of transitions, whose components in *x* are positive, i.e., *x* > 0, defines the support of the TI, supp(*x*). A TI, *x*, is minimal if no other solution, *x*‘, exists with supp(*x*) ⊇ supp(*x*’), and the largest common divisor of all elements of *x* equals one. We consider minimal, non-negative, integer solutions, *x*, as a TI. A PN is *covered by TIs* (*CTI*) if every transition is a member of at least one TI. For metabolic systems, a TI, then called an elementary mode, describes a biological pathway at steady state [98]. The concept of elementary modes has been developed via a convex cone analysis and has been mainly applied to metabolic systems. Meanwhile, also applications to signaling and gene regulatory systems have been published [99]. The analysis of TIs gives a rigorous way to verify the model for its correctness [100]. The CTI property describes the consistency in a theoretical sense [32].

### Place invariants

Analogously to TIs, we define a PI as an integer solution, *x*: *T*→ℕ, of the equation *y C*^*T*^ = 0, where *C*^*T*^ denotes the transposed incidence matrix. The definition of a minimal and true PI is analogous to the definition of a minimal and true TI. A PN is *covered by PIs* (CPI), if every place is member of at least one PI.

### Manatee invariants

The criterion for network verification demanded for a plausible biological interpretation for every TI. For signal transduction networks, the determination of all possible signal flows from the signal reception to cellular response is elementary to derive insights of the behavior of the biological system. This is a necessary prerequisite for the biological verification of the PN model as well as follow-up studies. The PN model described the molecular processes of TNFR1 signal transduction on a high level of detail. To allow for network analysis, we could have modeled the processes in a more abstract way but this would have simplified the processes, and the model might have lost important regulations. Therefore, we decided to adapt the TI analysis to obtain biologically meaningful results in terms of complete biological pathways, while keeping the high level of detail of the PN model. We introduced so-called Manatee invariants as linear combinations of TIs to ensure that the TI covers a signaling pathway from receptor activation to cell response. For the detailed definition and derivation of MIs, we refer to [63] Amstein *et al*, 2017.

### Invariant-induced subnetworks

Each invariant induces a subnetwork. A TI-induced or MI-induced subnetwork is formed by the transitions of the TI or MI, respectively, and the places and edges in between. Analogously, a PI-induced subnetwork is defined by the places of the PI and transitions and edges in between. Invariants decompose the PN into subnetworks that can overlap. These subnetworks should be biologically relevant and should represent biologically functional modules.

### *In silico* knockout analysis

Knockout studies or perturbation studies are suitable methods to reveal the vulnerable parts of a system. The *in silico* knockout analysis supports a profound investigation of a comprehensive PN model of a signaling pathway [101]. We define a knockout matrix, where each row represents the knockout of a protein, i.e., the deletion of an input transition. Each column denotes a protein or a protein complex of the PN, whose formation could be affected by the considered knockouts. We visualize the knockout results by coloring the matrix entries either gray (red) if the place is part (not part) of at least one MI-induced subnet. Biologically, a gray entry indicates that the formation of the respective protein or protein complex remains unaffected by the knockout, while a red entry stands for an effect on protein or protein complex formation.

### Software

For model reconstruction and analysis, we used the open-source software MonaLisa [40,41], available under https://github.com/MolBIFFM/MonaLisa/tree/master/store. The knockout was performed applying the open-source software *isiKnock* [64] based on MIs using additional output transitions. For the hierarchical clustering of the matrix entries, we applied the open-source software NOVA [65] with the settings UPGMA (Unweighted Pair Group Method with Arithmetic mean) [66] and the Pearson correlation [67] as distance measure.

## Supporting information

S1 Text The extrinsic and intrinsic pathways of apoptosis induction. (DOCX)

S1 Table List of 130 transitions. For each transition, we list its name, describe the biological meaning, and give references to relevant literature. For degradation and synthesis of proteins, we give no reference to literature.
(DOCX)

S2 Table Cell line specifications of transitions and references to experiments. For transitions, references and specifications of cell lines that have been used in relevant *in vivo* experiments are given.
(DOCX)

S3 Table List of 118 places. For each place, its name and biological meaning are given. For abbreviations applied to name places, we refer to S4 Table.
(DOCX)

S4 Table List of abbreviations applied to name places, see S3 Table and the Petri net of TNFR1 signal transduction in Fig 2. (DOCX)

S5 Table List of 48 transition invariants (TIs). For each TI, the number, the names of the transitions, and the biological meaning are given. The number is highlighted by bold face if the TI covers a dissected pathway. 33 TIs represent dissected pathways. An example of a dissected pathway is TI_9_. TI_9_ induces A20 feedback regulation but ignores the interrelation of this process with a necessary activation of NF-κB, see text.
(DOCX)

S6 Table List of 279 Manatee invariants (MIs). For each MI, its number, the number(s) of the transition invariants (TIs), and the names of transitions are given. For descriptions of transitions, we refer to S1 Table. For descriptions of TIs, see S5 Table. An MI combines several TIs to cover a complete pathway. We indicate whether an MI is pure. A pure MI induces a network that is free of place invariants, see text.
(DOCX)

S7 Table Effects of *in silico* knockouts of specific proteins. The list interprets the *in silico* knockout matrix of Fig 10. For each row of the matrix, the table lists the knocked out protein(s), the number of affected proteins or complexes, and descriptions of affected processes and of related processes. Knockouts with identical patterns of red circles are merged.
(DOCX)

S1 Fig Exemplary transition invariant (TI), TI_15_. TI_15_ highlighted in red describes the activation of NF-κB, the degradation of IκB, the gene expression of IκB, and the interaction of complex I with the inhibitory complex of NF-κB and I κB. The pathway relies on a former production of complex I, place CI. The assembly process of complex I is not part of TI_15_ and hence, TI_15_ represents an incomplete pathway.
(DOCX)

S2 Fig Exemplary transition invariant (TI), TI_9_. TI_9_ highlighted in blue describes the assembly of complex I and the dissociation of complex I via A20. The transcription of A20 relies on a former translocation of NF-κB into the nucleus. The translocation of NF-κB into the nucleus is not part of TI_9_ and hence, TI_9_ represents an incomplete pathway.
(DOCX)

S3 Fig Exemplary Manatee invariant (MI), MI_131_. MI_131_ is the linear combination of four transitions invariants (TIs), TI_4_ highlighted in dark green, TI_9_ highlighted in blue, TI_15_ highlighted in red, and TI_18_ highlighted in green. Note that, the TIs, i.e., their color code in the figure, may overlap. For detailed information on the TIs, we refer to **S5 Table**. MI_131_ represents a possible signal flow that is induced by the binding of TNF to the receptor TNFR, see TI_9_ and TI_4_, highlighted in blue and in dark green, respectively. The assembly of complex I, place CI, is part of TI_9_. MI_131_ combines the assembly of complex I with the degradation of complex I, see TI_15_, highlighted in red, and the synthesis of NF-κB, see TI_18_, highlighted in green. MI_131_ includes the inhibition of apoptosis by cFLIP, see TI_4_, highlighted in dark green. MI_131_ represents a complete pathway. MI_131_ resolves all relevant preconditions and interrelation of processes, as, e.g., the A20 feedback loop is accompanied by a preceding activation of NF-κB.
(DOCX)

S4 Fig Exemplary Manatee invariant (MI), MI_209_. MI_209_ is the linear combination of four transition invariants (TI), TI_9_, highlighted in blue, TI_15_, highlighted in red, TI_16_, highlighted in purple, and TI_18_, highlighted in green. Note that, the transition invariants, i.e., their color code in the figure, may overlap. For detailed information on the transition invariants, we refer to S5 Table. MI_209_ represents a possible signal flow that is induced by the binding of TNF to the receptor TNFR, see TI_9_, highlighted in blue. The assembly of complex I, place CI, is part of TI_9_. MI_209_ combines the assembly of complex I with the degradation of complex I, see TI_16_, highlighted in purple, the synthesis of NF-κB, see TI_18_, highlighted in green, and the translocation of NF-κB into the nucleus followed by the induction of transcription of IκB, see TI_15_, highlighted in red. MI_209_ represents a complete pathway. MI_209_ resolves all relevant preconditions and interrelation of processes, as, e.g., the A20 feedback loop is accompanied by a preceding activation of NF-κB.
(DOCX)

S1 Data Petri net in format of Systems Biology Markup Language (SBML). (XML)

## References

[1] Walczak H, Kantari C. Death Domain-Containing Receptors–Decision between Suicide and Death. In: Reed JC, Green DR, editors. Apoptosis: Physiology and Pathology. Cambridge University Press; 2011. pp. 23–36.

[2] Walczak H. TNF and ubiquitin at the crossroads of gene activation, cell death, inflammation, and cancer. Immunol Rev. 2011;244:9–28. doi: 10.1111/j.1600-065X.2011.01066.x 22017428

[3] Pasparakis M, Vandenabeele P. Necroptosis and its role in inflammation. Nature. 2015;517:311–320. doi: 10.1038/nature14191 25592536

[4] Reed JC, Green DR (editors). Apoptosis: Physiology and Pathology. Cambridge, UK: Cambridge University Press; 2011.

[5] Taylor RC, Cullen SP, Martin SJ. Apoptosis: controlled demolition at the cellular level. Nat Rev Mol Cell Biol. 2008;9(3):231–241. doi: 10.1038/nrm2312 18073771

[6] Galluzzi L, Vitale I, Aaronson SA, Abrams JM, Adam D, Agostinis P, et al. Molecular mechanisms of cell death: Recommendations of the nomenclature committee on cell death. Cell Death Differ. 2008;25(3):1–56. 10.1038/s41418-017-0012-4 PMC5864239 29362479

[7] Dhuriya YK, Sharma D. Necroptosis: a regulated inflammatory mode of cell death. J Neuroinflamm. 2018;15:199. Available from: doi: 10.1186/s12974-018-1235-0 29980212 PMC6035417

[8] Degterev A, Huang Z, Boyce M, Li Y, Jagtap P, Mizushima N, et al. Chemical inhibitor of nonapoptotic cell death with therapeutic potential for ischemic brain injury. Nat Chem Biol. 2005;1:112–119. doi: 10.1038/nchembio711 16408008

[9] Vandenabeele P, Galluzzi L, Berghe TV, Kroemer G Molecular mechanisms of necroptosis: an ordered cellular explosion. Nat Rev Mol Cell Biol. 2010;11:700–714. 20823910 10.1038/nrm2970

[10] Schwabe RF, Luedde T. Apoptosis and necroptosis in the liver: a matter of life and death. Nat Rev Gastroenterol Hepatol. 2018;15(12):738–752. doi: 10.1038/s41575-018-0065-y 30250076 PMC6490680

[11] DiDonato JA, Mercurio F, Karin M. NF-κB and the link between inflammation and cancer. Immun Rev. 2012;246:379–400. doi: 10.1111/j.1600-065X.2012.01099.x 22435567

[12] Fulda S, Galluzzi L, Kroemer G. Targeting mitochondria for cancer therapy. Nat Rev Drug Discovery. 2010;9(6):447–464. doi: 10.1038/nrd3137 20467424

[13] Fulda S. Targeting Apoptosis Signaling in Pancreatic Cancer. Cancers. 2011;3: 241–251. doi: 10.3390/cancers3010241 24212616 PMC3756359

[14] Fulda S, Rajalingam K, Dikic I. Ubiquitylation in immune disorders and cancer: from molecular mechanisms to therapeutic implications. EMBO Mol Med. 2012;4: 545–556. doi: 10.1002/emmm.201100707 22730341 PMC3407942

[15] Fulda S, Vukic D. Targeting IAP proteins for therapeutic interventions in cancer. Nat Rev Drug Discovery. 2012;11:109–124. 22293567 10.1038/nrd3627

[16] Fulda S. Alternative Cell Death Pathways and Cell Metabolism. Internat J Cell Biol. 2013;2013: 463637. Available from: doi: 10.1155/2013/463637 23401689 PMC3564271

[17] Fulda S. Therapeutic exploitation of necroptosis for cancer therapy. Seminars Cell & Dev Biol. 2014;35: 51–56. doi: 10.1016/j.semcdb.2014.07.002 25065969

[18] Peltzer N, Darding M, Walczak H. Holding RIPK1 on the Ubiquitin Leash in TNFR1 Signaling. Trends Cell Biol. 2016;26:445–461. doi: 10.1016/j.tcb.2016.01.006 26877205

[19] Ikeda F & Dikic I. Atypical ubiquitin chains: new molecular signals. EMBO. 2008;9:536–542. 10.1038/embor.2008.93 PMC2427391 18516089

[20] Grabbe C, Husnjak K, Dikic I. The spatial and temporal organization of ubiquitin networks. Nat Rev Mol Cell Biol. 2011;12: 295–307. doi: 10.1038/nrm3099 21448225 PMC3654194

[21] Walczak H, Iwai K, Dikic I. Generation and physiological roles of linear ubiquitin chains. BMC Biol. 2012;10: 23. Available from: doi: 10.1186/1741-7007-10-23 22420778 PMC3305636

[22] Kensche T, Tokunaga F, Ikeda F, Goto E, Iwai K, Dikic I. Analysis of Nuclear Factor- κB (NF-κB) essential modulator (NEMO) binding to linear and lysine-linked ubiquitin chains and its role in the activation of NF-κB. J Biol Chem. 2012;287:23626–23634. doi: 10.1074/jbc.M112.347195 22605335 PMC3390637

[23] Declercq W, Berghe TV, Vandenabeele P. RIP Kinases at the Crossroads of Cell Death and Survival. Cell. 2009;138:229–232. doi: 10.1016/j.cell.2009.07.006 19632174

[24] Hoeller D, Dikic I. Targeting the ubiquitin system in cancer therapy. Nature. 2009;458:438–444. doi: 10.1038/nature07960 19325623

[25] Kitano H. Systems Biology: A Brief Overview. Science. 2002;295:1662–1664. doi: 10.1126/science.1069492 11872829

[26] Kitano H. Biological Robustness. Nature. 2004;5:826–837. doi: 10.1038/nrg1471 15520792

[27] Saez-Rodriguez J, MacNamara A, Cook S. Modeling Signaling Networks to advance New Cancer Therapies. Ann Rev Biomed Engin. 2015;17: 143–163. doi: 10.1146/annurev-bioeng-071813-104927 26274601

[28] Heinrich R, Rapoport TA. A Linear Steady-State Treatment of Enzymatic Chains General Properties, Control and Effector Strength. Eur J Biochem. 1974;42:89–95. 4830198 10.1111/j.1432-1033.1974.tb03318.x

[29] Aldridge BB, Burke JM, Luffenburger DA, Sorger PK. Physicochemical modelling of cell signaling pathways. Nat Cell Biol. 2006;8:1195–1203. 17060902 10.1038/ncb1497

[30] Wang R-S, Saadatpour A, Albert R. Boolean modeling in systems biology: an overview of methodology and applications. Phys Biol. 2012; 9: 055001. doi: 10.1088/1478-3975/9/5/055001 23011283

[31] Reisig W. Petri Nets: An Introduction. In: Brauer W, Rozenberg G, Salomaa A, editors. EATCS, Monographs on Theoretical Computer Science. Berlin: Springer Verlag; 1985.

[32] Murata T (1989) Petri nets: Properties, analysis and applications. In: Proc IEEE 1989;77(4). pp. 541–580.

[33] Koch I, Junker BH, Heine M. Application of Petri net theory for modelling and validation of the sucrose breakdown pathway in the potato tuber. Bioinformatics. 2005;21:1219–1226. doi: 10.1093/bioinformatics/bti145 15546934

[34] Formanowicz D, Sackmann A, Formanowiczb P, Błażewicz J. Petri net based model of the body iron homeostasis. J Biomed Inform. 2006;40:476–485. doi: 10.1016/j.jbi.2006.12.001 17258508

[35] Sackmann A, Formanowicz D, Formanowicz P, Koch I, Blazewicz J. An analysis of the Petri net based model of the human body iron homeostasis process. Comput Biol Chem. 2007;31: 1–10. doi: 10.1016/j.compbiolchem.2006.09.005 17097351

[36] Koch I, Reisig W, Schreiber F. (editors) Modeling in Systems Biology. Springer Berlin/Heidelberg, Berlin Heidelberg New York. 2011.

[37] Minervini G, Panizzoni E, Giollo M, Masiero A, Ferrari C, Tosatto SCE. Design and Analysis of a Petri Net Model of the Von Hippel-Lindau (VHL) Tumor Suppressor Interaction Network. PLoS ONE. 2014;9(6):e96986. Available from: doi: 10.1371/journal.pone.0096986 24886840 PMC4041725

[38] Koch I, Nöthen J, Schleiff E. Modeling the metabolism of Arabidopsis thaliana: application of network decomposition and network reduction in the context of Petri nets. Front Genetics. 2017;8:85–107. 10.3389/fgene.2017.00085 PMC5491931 28713420

[39] Jacobsen A, Ivanova O, Amini S, Heringa J, Kemmeren P, Feenstra KA. A framework for exhaustive modelling of genetic interaction patterns using Petri nets. Bioinformatics. 2020;36:2142–2149. doi: 10.1093/bioinformatics/btz917 31845959

[40] Einloft J, Ackermann J, Nöthen J, Koch I. MonaLisa—visualization and analysis of functional modules in biochemical networks. Bioinformatics. 2013;29:1469–1470. doi: 10.1093/bioinformatics/btt165 23564846

[41] Balazki P, Lindauer K, Einloft J, Ackermann J, Koch I. MONALISA for stochastic simulations of Petri net models of biochemical systems. BMC Bioinformatics. 2015;16:215. Available from: doi: 10.1186/s12859-015-0596-y 26156221 PMC4496887

[42] Mitchell S, Vargas J, Hoffmann. A Signaling via the NF-κB system. WIREs Systems Biol Med. 2016;8:227–241. 10.1002/wsbm.1331 PMC8363188 26990581

[43] Basak S, Behar M, Hoffmann A. Lessons from mathematically modeling the NF-κB pathway. Immun Rev. 2012;246:221–238. doi: 10.1111/j.1600-065X.2011.01092.x 22435558 PMC3343698

[44] Cheng TMK, Gulati S, Agius R, Bates PA. Understanding cancer mechanisms through network dynamics. Brief Funct Genomics. 2012;11:543–560. doi: 10.1093/bfgp/els025 22811516

[45] Hoffmann A, Levchenko A, Scott M, Baltimore D. The IκB-NF-κB Signaling Module: Temporal Control and Selective Gene Activation. Science. 2002;298:1241–1245. 12424381 10.1126/science.1071914

[46] Lipniacki T, Paszek P, Brasier AR, Luxon B, Kimmel M. Mathematical model of NF-κB regulatory module. J Theor Biol. 2004;228:195–215. 15094015 10.1016/j.jtbi.2004.01.001

[47] Kearns JD, Basak S, Werner SL, Huang CS, Hoffmann A. IκBɛ provides negative feedback to control NF-κB oscillations, signaling dynamics, and inflammatory gene expression. J Cell Biol. 2006;173:659–664. 16735576 10.1083/jcb.200510155 PMC2063883

[48] Rangamani P, Sirovich L. Survival and apoptotic pathways initiated by TNF-α: Modeling and predictions. Biotech & Bioengin. 2007;97: 1216–1229. 10.1002/bit.21307 17171720

[49] Tay S, Hughey JJ, Lee TK, Lipniacki T, Quake SR. Covert MW Single-cell NF-κB dynamics reveal digital activation and analogue information processing. Nature. 2010;466:267–271. 20581820 10.1038/nature09145 PMC3105528

[50] Sheppard PW, Sun X, Emery JF, Giffard RG, Khammash M. Quantitative characterization and analysis of the dynamic NF-κB response in microglia. BMC Bioinformatics. 2011;12: 276. Available from: doi: 10.1186/1471-2105-12-276 21729324 PMC3158563

[51] Mothes J, Busse D, Kofahl B, Wolf J. Sources of dynamic variability in NF-κB signal transduction: A mechanistic model. BioEssays. 2015;37:452–462. doi: 10.1002/bies.201400113 25640005 PMC4409097

[52] Inoue K, Shinohara H, Behar M, Yumoto N, Tanaka G, Hoffmann A, Aihara K, Okada-Hatakeyama M. Oscillation dynamics underlie functional switching of NF-κB for B-cell activation. npj Syst Biol and Appl. 2016; 2:16024. Available from: doi: 10.1038/npjsba.2016.24 28725478 PMC5516862

[53] Wang Z, Potoyan DA, Wolynes PG. Modeling the therapeutic efficacy of NFκB synthetic decoy oligodeoxynucleotides (ODNs). BMC Syst Biol. 2018;12: 4. Available from: doi: 10.1186/s12918-018-0525-6 29382384 PMC5791368

[54] Peng SC, Wong DSH, Tung KC, Chen YY, Chao CC, Peng CH, et al. Computational modeling with forward and reverse engineering links signaling network and genomic regulatory responses: NF-κB signaling-induced gene expression responses in inflammation. BMC Bioinformatics. 2010;11: 308. Available from: 10.1186/1471-2105-11-308 20529327 PMC2889938

[55] Calzone L, Tournier L, Fourquet S, Thieffry D, Zhivotovsky B, Barillot E, Zinovyev A. Mathematical Modelling of Cell-Fate Decision in Response to Death Receptor Engagement. PLoS Comput Biol. 2010;6:e1000702. Available from: doi: 10.1371/journal.pcbi.1000702 20221256 PMC2832675

[56] Schlatter R, Schmich K, Vizcarra IA, Scheurich P, Sauter T, Borner C, et al. ON/OFF and Beyond–A Boolean Model of Apoptosis. PLoS Comput Biol. 2009;5: e1000595.Available from: doi: 10.1371/journal.pcbi.1000595 20011108 PMC2781112

[57] Schliemann M, Bullinger E, Borchers S, Allgöwer F, Findeisen R, Scheurich P Heterogeneity reduces sensitivity of cell death for TNF-stimuli. BMC Syst Biol. 2011;5: 204. Available from: 10.1186/1752-0509-5-204 22204418 PMC3313907

[58] Melas I, Mitsos A, Messinis DE, Weiss TS, Alexopoulos LG. Combined logical and data-driven models for linking signalling pathways to cellular response. BMC Syst Biol. 2011;5:107. Available from: doi: 10.1186/1752-0509-5-107 21729292 PMC3145575

[59] Mothes J, Ipenberg I, Arslan SÇ, Benary U, Scheidereit C, Wolf J. A Quantitative Modular Modeling Approach Reveals the Effects of Different A20 Feedback Implementations for the NF-kB Signaling Dynamics. Front Physiol. 2020;11:896. Available from: doi: 10.3389/fphys.2020.00896 32848849 PMC7402004

[60] Yao S, Shatz SM. Consistency Checking of UML Dynamic Models Based on Petri Net Techniques, 15th Int Conf Comp. 2006; pp. 289–297. Available from: 10.1109/CIC.2006.32.

[61] Heiner M, Koch I. Petri Net Based Model Validation in Systems Biology. Proc 25th Int Conf Appl Theory of Petri Nets, LCNS. 2004;3099:216–237. Available from: 10.1007/978-3-540-27793-4_13.

[62] Inkscape Project. Inkscape. 2020. Available from: https://inkscape.org

[63] Amstein L, Ackermann J, Scheidel J, Fulda S, Dikic I, Koch I. Manatee invariants reveal functional pathways in signaling networks. BMC Syst Biol. 2017;11:72. Available from doi: 10.1186/s12918-017-0448-7 28754124 PMC5534052

[64] Hannig J, Giese H, Schweizer B, Amstein L, Ackermann J, Koch I. isiKnock: in silico knockouts in biochemical pathways. Bioinformatics. 2019;5:892–894. 10.1093/bioinformatics/bty700 30102342

[65] Giese H, Ackermann J, Heide H, Bleier L, Dröse S, Wittig I, et al. NOVA: a software to analyze complexome profiling data. Bioinformatics. 2015;31:440–441. doi: 10.1093/bioinformatics/btu623 25301849

[66] Sokal R, Michener C. A statistical method for evaluating systematic relationships. Univ Kansas Sci Bull. 1958.

[67] Pearson K. Notes on regression and inheritance in the case of two parents. Proc R Soc Lond. 1895;58:240–242.

[68] Ting AT, Bertrand MJM. More to Life than NF-κB in TNFR1 Signaling. Trends Immunol. 2016;37(8):535–545. 27424290 10.1016/j.it.2016.06.002 PMC5076853

[69] Karin M, Lin A. NF-κB at the crossroads of life and death. Nat Immunol. 2002;3:221–227. 11875461 10.1038/ni0302-221

[70] Wallach D. The cybernetics of TNF: Old views and newer ones. Seminars in Cell & Developmental Biol. 2016;50: 105–114. doi: 10.1016/j.semcdb.2015.10.014 26474540

[71] Wajant H, Scheurich P. TNFR1-induced activation of the classical NF-κB pathway. FEBS J. 2011;278(6):862–876. doi: 10.1111/j.1742-4658.2011.08015.x 21232017

[72] Wertz I, Dixit VM. Ubiquitin-mediated regulation of TNFR1 signaling. Cytokine and Growth Factor Rev. 2008;19(3–4): 313–324. doi: 10.1016/j.cytogfr.2008.04.014 18515172

[73] Lockshin RA, Zekeri Z. Cell death in health and disease. J Cellular & Mol Med. 2007;11:1214–1224. doi: 10.1111/j.1582-4934.2007.00150.x 18031301 PMC4401285

[74] Schmidt N, Haydn T, Schneider I, Busch H, Boerries M, Fulda S. Smac mimetic induces an early wave of gene expression via NF-κB and AP-1 and a second wave via TNFR1 signaling. Cancer Letters. 2018;421(1): 170–185. doi: 10.1016/j.canlet.2018.01.082 29421152

[75] Bertrand MJM, Milutinovic S, Dickson KM, Ho WC, Boudreault A, Durkin J, et al. cIAP1 and cIAP2 falicitate cancer cell survival by functioning as E3 ligases that promote RIP1 ubiquitination. Mol Cell. 2008;30(6):689–700. 18570872 10.1016/j.molcel.2008.05.014

[76] Jaco I, Annibaldi A, Lalaoui N, Wilson R, Tenev T, Laurien L, et al. MK2 Phosphorylates RIPK1 to Prevent TNF-induced Cell Death. Mol Cell. 2017;66(5):698–710. doi: 10.1016/j.molcel.2017.05.003 28506461 PMC5459754

[77] Oberst A. MK2 balances inflammation and cell death. Nat Cell Biol. 2017;9(10):1150–1152. doi: 10.1038/ncb3619 28960200

[78] Tenev T, Bianchi K, Darding M, Broemer M, Langlais C, Wallberg F, et al. The Ripoptosome, a Signaling Platform that Assembles in Response to Genotoxic Stress and Loss of IAPs. Mol Cell. 2011;43(3):432–448. doi: 10.1016/j.molcel.2011.06.006 21737329

[79] Feokistova M, Geserick P, Kellert B, Dimitrova DP, Langlais C, Hupe M, et al. cIAPs Block Ripoptosome Formation, a RIP1/Caspase-8 Containing Intracellular Cell Death Complex Differentially Regulated by cFLIP Isoforms. Mol Cell. 2011;43(3):449–463. doi: 10.1016/j.molcel.2011.06.011 21737330 PMC3163271

[80] Dondelinger Y, Aguileta MA, Goossens V, Dubuisson C, Grootjans S, Dejardin E, et al. RIPK3 contributes to TNFR1-mediated RIPK1 kinase-dependent apoptosis in condition of cIAP1/2 depletion or TAK1 kinase inhibition. Cell Death & Differ. 2013;20(10):1381–1392. 10.1038/cdd.2013.94 PMC3770330 23892367

[81] Dondelinger Y, Jouan-Lanhouet S, Divert T, Theatre E, Bertin J, Gough PJ, et al. NF-κB-Independent Role of IKKα/IKKβ in Preventing RIPK1 Kinase-Dependent Apoptotic and Necroptotic Cell Death during TNF Signaling. Mol Cell. 2015;60(1) 63–76. doi: 10.1016/j.molcel.2015.07.032 26344099

[82] Dondelinger Y, Delanghe T, Rojas-Rivera D, Priem D, Delvaeye T, Bruggeman I, et al. MK2 phosphorylation of RIPK1 regulates TNF-mediated cell death. Nat Cell Biol. 2017;19(10):1237–1247. doi: 10.1038/ncb3608 28920952

[83] Linkermann A, Green DR. Necroptosis. New England J Med. 2014;370(5):455–465. 24476434 10.1056/NEJMra1310050 PMC4035222

[84] Gerlach B, Cordier SM, Schmukle AC, Emmerich CH, Rieser E, Haas TL, et al. Linear ubiquitination prevents inflammation and regulates immune signaling. Nature. 2011;471:591–596. 21455173 10.1038/nature09816

[85] Weinlich R, Oberst A, Beere HM, Green DR. Necroptosis in development, inflammation and disease. Mol Cell Biol. 2016;18(2): 127–136. doi: 10.1038/nrm.2016.149 27999438

[86] Tsuchiya Y, Nakabayashi O, Nakano H. FLIP the Switch: Regulation of Apoptosis and Necroptosis by cFLIP. Int J Mol Sci. 2015;16(12):30321–30341. doi: 10.3390/ijms161226232 26694384 PMC4691174

[87] Shore GC, Nguyen M. Bcl-2 proteins and apoptosis: Choose your partner. Cell. 2008;135(6):1004–1006. doi: 10.1016/j.cell.2008.11.029 19070569

[88] Micheau O, Tschopp J. Induction of TNF Receptor I-Mediated Apoptosis via Two Sequential Signaling Complexes. Cell. 2003;114(2):181–190. doi: 10.1016/s0092-8674(03)00521-x 12887920

[89] Oberst A, Dillon CP, Weinlich R, McCormick LL, Fitzgerald P, Pop C, et al. Catalytic activity of the caspase-8-FLIPL complex inhibits RIPK3-dependent necrosis. Nature. 2011;471(7338):363–367. doi: 10.1038/nature09852 21368763 PMC3077893

[90] Dillon CP, Oberst A, Weinlich R, Janke LJ, Kang T-B, Ben-Moshe T, et al. Survival Function of the FADD-CASPASE-8-cFLIPL Complex. Cell Reports. 2012;1(5):401–407. doi: 10.1016/j.celrep.2012.03.010 22675671 PMC3366463

[91] Onizawa M, Oshima S, Schulze-Topphoff U, Oses-Prieto JA, Lu T, Tavares R, et al. The ubiquitin-modifying enzyme A20 restricts ubiquitination of the kinase RIPK3 and protects cells from necroptosis. Nat Immunol. 2015;16(6): 618–627. doi: 10.1038/ni.3172 25939025 PMC4439357

[92] O’Donnell MA, Perez-Jimenez E, Oberst A, Massouri R, Xavier R, Green DR, et al. Caspase 8 inhibits programmed necrosis by processing CYLD. Nat Cell Biol. 2011;13(12):1437–1442. doi: 10.1038/ncb2362 22037414 PMC3229661

[93] Zhou K.-Q., Azlan Z. Fuzzy Petri nets and industrial applications: a review. Artificial Intelligence Rev. 2016;45: 1–42.

[94] Reddy VN, Liebmann MN, Mavrovouniotis ML. Petri Net Representations in Metabolic Pathways. In: Proc Int Conf Intell Syst Mol Biol. 1993;94(1). pp. 328–336. 7584354

[95] Grunwald S, Speer A, Ackermann J, Koch I Petri net modelling of gene regulation of the Duchenne muscular dystrophy BioSystems. 2008;92:189–205. 18372101 10.1016/j.biosystems.2008.02.005

[96] Kielbassa J, Bortfeldt R, Schuster S, Koch I. Modeling of the U1 snRNP assembly pathway in alternative splicing in human cells using Petri nets. Comp Biol Chem. 2009;33:46–61. doi: 10.1016/j.compbiolchem.2008.07.022 18775676

[97] Lautenbach K, GMD Report. 1973; No. 82.

[98] Schuster S, Hilgetag C. On elementary flux modes in biochemical reaction systems at steady state. J Biol Sys. 1994;2(02):597–617.

[99] Trares K, Ackermann J, Koch I.The canonical and non-canonical NF-κB pathways and their crosstalk: A comparative study based on Petri nets. BioSystems. 2021;11:104564. Available from: doi: 10.1016/j.biosystems.2021.104564 34688841

[100] Schuster S, Pfeiffer T, Moldenbauer F, Koch I, Dandekar T. Exploring the pathway structure of metabolism: decomposition into subnetworks and application to Mycoplasma pneumoniae. Bioinformatics. 2002;18(2):351–361. doi: 10.1093/bioinformatics/18.2.351 11847093

[101] Scheidel J, Amstein L, Ackermann J, Dikic I, Koch I. In silico knockout studies of xenophagic capturing of Salmonella. PLoS Comput Biol, (2016)12(12): e1005200. Available from doi: 10.1371/journal.pcbi.1005200 27906974 PMC5131900

