# Correction: Mathematical modeling of the molecular switch of TNFR1-mediated signaling pathways applying Petri net formalism and *in silico* knockout analysis

**DOI:** 10.1371/journal.pcbi.1011065

**Published:** 2023-04-14

**Authors:** The PLOS Computational Biology Staff

## Notice of Republication

This article was republished on April 3, 2023, to correct the fourth author’s name in the article PDF. The publisher apologizes for the errors. Please download this article again to view the correct version.
