# Supplementary material for: Mathematical modeling of the molecular switch of TNFR1-mediated signaling pathways applying Petri net formalism and in silico knockout analysis
Source: PLoS Comput Biol. 2022 Aug 22;18(8):e1010383. doi: 10.1371/journal.pcbi.1010383 (PMC9467317; doi:10.1371/journal.pcbi.1010383)
Supplement: S1 Text — (DOCX) [file pcbi.1010383.s001.docx]

**The extrinsic and intrinsic pathways of apoptosis induction**

In the extrinsic pathway, the induction of apoptosis can be triggered by death receptors like TNFR1, which exhibits a death domain DD to bind adaptor proteins that can initiate cascades to cell death [1]. The disassociation of complex I and the internalization of TNFR1 leads to a modulation of the assembled proteins [2]. Among the cytosolic proteins are TRADD, Fas-associated protein with a death domain (FADD), RIP1, cellular FLICE-inhibitory protein (cFLIP) isoforms, and procaspase 8 or procaspase 10, forming the death-inducing signaling complex (DISC) or complex II [3]. FADD is an important adaptor protein because its death effector domain (DED) can recruit the effector caspases and facilitate their homodimerization and activation [4]. Caspases are present in their inactive proenzyme form as procaspases. The full activation requires a proteolytic cleavage of the procaspases. The proteolytic self-activation is achieved by formation of a dimer of procaspases [5]. cFLIP is highly homologous to procaspase 8 and, therefore, competes with procaspase 8 for binding to FADD. Since cFLIP lacks enzymatic capability, it prevents full activation. Two isoforms of cFLIP exist, the short form cFLIPS and the long form cFLIPL [6]. Both isoforms have a different effect for caspase activity within complex II. While cFLIPS fully inhibits caspase activity, cFLIPL only restricts the proteolytic activity to the local substrates associated to complex II like RIP1 [7,8]. The associated proteins can vary depending on different cell types and cellular conditions. The formation of the complex II can directly promote the dimerization and activation of CASP8, which in turn cleaves and activates CASP3 inducing the relevant cell death processes via the extrinsic pathway [9].

A second pathway can be triggered by the intrinsic branch via the mitochondrion. CASP8 cleaves BH3-interacting domain death agonist (BID) to truncated BID (tBID) [10]. tBID induces the permeabilization of the outer mitochondrial membrane by activation of BCL-2 antagonist/killer (BAK) and BCL-2-associated X protein (BAX) [11]. B-cell lymphoma 2 (BCL-2) can negatively regulate BAX activation by preventing its oligomerization [12,13]. Oligomerization of BAX or BAK forms pores in the outer mitochondrial membrane inducing MOMP and the release of proteins, which usually reside inside the mitochondrial intermembrane space, like cytochrome c (cyt c) and second mitochondria derived activator of caspase (SMAC) [14]. MOMP is generally assumed as a point of no return for apoptosis as cytotoxic molecules are released and the integrity of the mitochondrion is destroyed [15]. The BCL-2 family of proteins comprises pro- and anti-apoptotic proteins and balance the induction or prevention of MOMP [11]. Cyt c forms the apoptosome associating apoptotic protease-activating factor 1 (Apaf1) and deoxyadenosine triphosphate (dATP), a platform to activate caspase 9 (CASP9) and eventually CASP3 [16]. CASP3 and CASP9 are executioner caspases that cleave various substrates to initiate apoptosis. X-linked inhibitor of apoptosis protein (XIAP) inhibits CASP9, CASP3, and caspase 7 (CASP7) activation by binding to the catalytic pocket [17,18,19]. The IAP family of proteins regulates cell death by blocking of caspase activity, either via direct binding or ubiquitination [20]. SMAC is a mitochondrial protein, which inhibits IAP proteins, especially XIAP to promote caspase-dependent apoptosis [21,17].

## References

1. Walczak H, Kantari C. Death Domain-Containing Receptors – Decision between Suicide and Death. In: Reed JC, Green DR, editors. Apoptosis: Physiology and Pathology. Cambridge University Press; 2011. pp. 23-36.

2. Micheau O, Tschopp J. Induction of TNF Receptor I-Mediated Apoptosis via Two Sequential Signaling Complexes. Cell. 2003;114(2):181-190.

3. Galluzzi L, Vitale I, Abrams JM, Alnemri ES, Baehrecke EH, Blagosklonny MV, Dawson TM, Dawson VL, El-Deiry WS, Fulda S, et al. Molecular definitions of cell death subroutines: recommendations of the Nomenclature Committee on Cell Death 2012. Cell Death Differ. 2012;19(1):107–120.

4. Dickens L, Powley I, Hughes M, MacFarlane M. The ‘complexities‘ of life and death: Death receptor signalling platforms. Exp Cell Res. 2012;318(11):1269–1277.

5. Oberst A, Pop C, Tremblay AG, Blais V, Denault J-B, Salvesen GS, Green DR. Inducible Dimerization and Inducible Cleavage Reveal a Requirement for Both Processes in Caspase-8 Activation. J Biol Chem. 2010;285(22):16632–16642.

6. Tsuchiya Y, Nakabayashi O, Nakano H. FLIP the Switch: Regulation of Apoptosis and Necroptosis by cFLIP. Int J Mol Sci. 2015;16(12):30321-30341.

7. Oberst A, Dillon CP, Weinlich R, McCormick LL, Fitzgerald P, Pop C, et al. Catalytic activity of the caspase-8-FLIPL complex inhibits RIPK3-dependent necrosis. Nature. 2011;471(7338):363-367.

8. Dillon CP, Oberst A, Weinlich R, Janke LJ, Kang T-B, Ben-Moshe T, et al. Survival Function of the FADD-CASPASE-8-cFLIPL Complex. Cell Reports. 2012;1(5):401-407.

9. Green DR. Means to an End: Apoptosis and Other Cell Death Mechanisms. Cold Spring Harbor Laboratory Press, 2011.

10. Shamas-Din A, Brahmbhatt H, Leber B, Andrews DW. BH3-only proteins: Orchestrators of apoptosis. Biochimica et Biophysica Acta (BBA) - Mol Cell Res. 2011;1813(4):508–520.

11. Chipuk JE, Green DR. How do BCL-2 proteins induce mitochondrial outer membrane permeabilization? Trends Cell Biol. 2008;18(4):157–164

12. Shore GC, Nguyen M. Bcl-2 proteins and apoptosis: Choose your partner. Cell. 2008;135(6):1004-1006.

13. Ola MS, M. Nawaz M, Ahsan H. Role of Bcl-2 family proteins and caspases in the regulation of apoptosis. Mol Cell Biochem. 2011;351(1-2):41–58.

14. Czabotar PE, Lessene G, Strasser A, Adams JM. Control of apoptosis by the BCL-2 protein family: implications for physiology and therapy. Nat Rev Mol Cell Biol. 2014;159(1):46-63.

15. Reed JC, Green DR (editors). Apoptosis: Physiology and Pathology. Cambridge, UK: Cambridge University Press; 2011.

16. Würstle ML, Laussmann MA, Rehm M. The central role of initiator caspase-9 in apoptosis signal transduction and the regulation of its activation and activity on the apoptosome. Exp Cell Res. 2012;318(11):1213–1220.

17. Galban S & Duckett CS. XIAP as a ubiquitin ligase in cellular signaling. Cell Death Differ. 2010;7(1):54–60.

18. Gyrd-Hansen M, Meier P. IAPs: from caspase inhibitors to modulators of NF-κB, inflammation and cancer. Nat Rev Cancer. 2010;10(8):561–574.

19. Schile AJ, García-Fernández M, Steller H. Regulation of apoptosis by XIAP ubiquitin-ligase activity. Genes Dev. 2008;22(16):2256–2266.

20. Fulda S, Vukic D. Targeting IAP proteins for therapeutic interventions in cancer. Nat Rev Drug Discovery. 2012;11:109-124.

21. Verhagen AM, Ekert PG, Pakusch M, Silke J, Connolly LM, Reid GE, Moritz RL, Simpson RJ, Vaux DL. Identification of DIABOLO, a Mammalian Protein that Promotes Apoptosis by Binding to and Antagonizing IAP Proteins. Cell. 2000;102(1):43–53.
