# Supplementary material for: Mathematical modeling of the molecular switch of TNFR1-mediated signaling pathways applying Petri net formalism and in silico knockout analysis
Source: PLoS Comput Biol. 2022 Aug 22;18(8):e1010383. doi: 10.1371/journal.pcbi.1010383 (PMC9467317; doi:10.1371/journal.pcbi.1010383)
Supplement: S1 Table — For each transition, we list its name, describe the biological meaning, and give references to relevant literature. For degradation and synthesis of proteins, we give no reference to literature. (DOCX) [file pcbi.1010383.s002.docx]

**S1 Table:** List of 130 transitions. For each transition, we list its name, describe the biological meaning, and give references to relevant literature. For degradation and synthesis of proteins, we give no reference to literature.

| **Transition** | **Biochemical meaning** | **References** |
| --- | --- | --- |
| Apoptosis | Induction of apoptosis | Reed & Green, 2011 [20]  Taylor *et al*, 2008 [24] |
| Apo_XIAP_inhib | Inhibition of XIAP by SMAC and apoptosis induction | Galban & Duckett, 2010 [8]  Verhagen *et al*, 2000 [29] |
| BAX_inhib | Inhibition of BAX and inhibition of apoptosis | Shore & Nguyen, 2008 [22]  Ola *et al*, 2011 [17] |
| CASP3_inhib | Inhibition of caspase 3 by XIAP and inhibition of apoptosis | Gyrd-Hansen & Meier, 2010 [9]  Schile *et al*, 2008 [21] |
| CI_diss | Dissociation of complex I by A20 regulation and termination of signal transduction | Draber *et al*, 2015 [6] |
| CIIa_inhib | cFLIPL bound to CIIa promotes survival | Dillon *et al*, 2012 [5]  Tsuchiya *et al*, 2015 [26] |
| deg1 | Degradation of RIP1 |  |
| deg2 | Degradation of TRADD |  |
| deg3 | Degradation of CASP8 |  |
| deg4 | Degradation of NF-κB |  |
| deg5 | Degradation of SMAC |  |
| deg6 | Degradation of CASP9 |  |
| deg7 | Degradation of IκB |  |
| diss1 | Dissociation of complex I |  |
| diss2 | Dissociation of TNFR1 and TNF-α |  |
| diss3 | Dissociation of complex IIa |  |
| diss4 | Dissociation of the necrosome |  |
| diss5 | Dissociation of complex IIb |  |
| diss6 | Dissociation of the apoptosome |  |
| Necroptosis | Induction of necroptosis | Wang *et al*, 2014 [30]  Cai *et al*, 2014 [2] |
| Pc8_inhib | Inhibition of procaspase 8 by cFLIPL and survival of the cell | Dillon *et al*., 2012 [5]  Tsuchiya *et al*, 2015 [26] |
| Pc9_inhib | Inhibition of procaspase 9 by XIAP and survival of the cell | Gyrd-Hansen & Meier, 2010 [9] |
| RIP1:RIP3_inhib | Cleavage and inhibition of RIP1 and RIP3 by cFLIPL | Oberst *et al*, 2011 [15]  Tsuchiya *et al*, 2015 [26] |
| Syn_Apaf1 | Synthesis of Apaf1 |  |
| Syn_A20 | Synthesis of A20 |  |
| Syn_BAX | Synthesis of BAX |  |
| Syn_BCL-2 | Synthesis of BCL-2 |  |
| Syn_Bid | Synthesis of BID |  |
| Syn_cFLIPL | Synthesis of cFLIPL |  |
| Syn_cFLIPS | Synthesis of cFLIPS |  |
| Syn_cIAP | Synthesis of cIAP proteins |  |
| Syn_CLYD | Synthesis of CYLD |  |
| Syn_Cyt c | Synthesis of cytochrome c |  |
| Syn_FADD | Synthesis of FADD |  |
| Syn_IkB | Synthesis of IκB |  |
| Syn_IKK | Synthesis of IKK |  |
| Syn_LUBAC | Synthesis of LUBAC |  |
| Syn_MLKL | Synthesis of MLKL |  |
| Syn_NEMO | Synthesis of NEMO |  |
| Syn_NF-kB | Synthesis of NF-κB |  |
| Syn_Procasp3 | Synthesis of procaspase 3 |  |
| Syn_Procasp8 | Synthesis of procaspase 8 |  |
| Syn_Procasp9 | Synthesis of procaspase 9 |  |
| Syn_RIP1 | Synthesis of RIP1 |  |
| Syn_RIP3 | Synthesis of RIP3 |  |
| Syn_SCF | Synthesis of SCFβ-TrCP |  |
| Syn_SMAC | Synthesis of SMAC |  |
| Syn_TAB | Synthesis of TAB2/3 |  |
| Syn_TAK1 | Synthesis of TAK1 |  |
| Syn_TNF | Synthesis of TNF-α |  |
| Syn_TNFR1 | Synthesis of TNFR1 |  |
| Syn_TRADD | Synthesis of TRADD |  |
| Syn_TRAF2 | Synthesis of TRAF2 |  |
| Syn_XIAP | Synthesis of XIAP |  |
| T1 | TNF-α binding to TNFR1 | Ting & Bertrand, 2016 [25]  Varfolomeev & Vucic, 2018 [26] |
| T2 | TRADD binding | Ting & Bertrand, 2016 [25]  Varfolomeev & Vucic, 2018 [26] |
| T3 | RIP1 binding | Ting & Bertrand, 2016 [25]  Varfolomeev & Vucic, 2018 [26] |
| T4 | TRAF2 binding | Ting & Bertrand, 2016 [25]  Varfolomeev & Vucic, 2018 [26] |
| T5 | cIAP1/2 binding | Ting & Bertrand, 2016 [25]  Varfolomeev & Vucic, 2018 [26] |
| T6 | K63-linked ubiquitination by cIAP1/2 | Ting & Bertrand, 2016 [25]  Varfolomeev & Vucic, 2018 [26] |
| T7 | TAB2/3 binding to K63 Ub chains | Kensche *et al*, 2012 [11] |
| T8 | Regaining of K63 Ub protein complex | Kensche *et al,* 2012 [11] |
| T9 | TAK1 binding to TAB2/3 | Kensche *et al*, 2012 [11] |
| T10 | NEMO binding to K63 Ub chains | Kensche *et al*, 2012 [11] |
| T11 | Regaining of K63 ubiquitinated complex I | Kensche *et al*, 2012 [11] |
| T12 | Binding of IKK to K63 recruited NEMO | Kensche *et al*, 2012 [11] |
| T13 | Formation of complex I and full activation of IKK | Zhang *et al*, 2014 [32]  Peltzer *et al*, 2016 [19] |
| T14 | Recruitment of LUBAC | Haas *et al*, 2009 [10]  Peltzer *et al*, 2016 [19] |
| T15 | Binding of NEMO to M1 Ub chains | Kensche *et al*, 2012 [11] |
| T16 | Regaining of K63 ubiquitinated complex I | Kensche *et al*, 2012 [11] |
| T17 | Binding of IKK to M1 recruited NEMO | Kensche *et al*, 2012 [11] |
| T18 | Binding of CYLD to LUBAC | Draber *et al*, 2015 [6] |
| T19 | Recruitment of CYLD | Draber *et al*, 2015 [6] |
| T20 | CYLD interacting with Ub chains in complex I | Draber *et al,* 2015 [6]  Kovalenko *et al*, 2003 [12] |
| T21 | CYLD deubiquitinates complex I | Draber *et al*, 2015 [6]  Kovalenko *et al*, 2003 [12] |
| T22 | Interaction of complex I and the inhibitory complex of NF-κB and I κB | Varfolomeev & Vucic, 2018 |
| T23 | Release of NF-κB and phosphorylated IκB | Brown *et al*., 1993 [1]  Varfolomeev & Vucic, 2018 [28] |
| T24 | Ubiquitination of IκB by SCF -βTrCP | Oeckinghaus & Ghosh, 2009 [16]  Varfolomeev & Vucic, 2018 [26] |
| T25 | Translocation of NF-κB into the nucleus | Varfolomeev & Vucic, 2018 [26] |
| T26 | NF-κB initiates gene expression of IκB | Pahl, 1999 [18]  Brown *et al*, 1993 [1] |
| T27 | Transcription of IκB mRNA | Pahl, 1999 [18]  Brown *et al*, 1993 [1] |
| T28 | NF-κB initiates gene expression of A20 | Pahl, 1999 [18] |
| T29 | Transcription of A20 mRNA | Pahl, 1999 [18] |
| T30 | NF-κB initiates gene expression of survival genes | Pahl, 1999 [18] |
| T31 | NF-κB initiates gene expression of XIAP | Pahl, 1999 [18] |
| T32 | Transcription of XIAP mRNA | Pahl, 1999 [18] |
| T33 | NF-κB initiates gene expression of cFLIP | Micheau *et al*, 2001 [13] |
| T34 | Transcription of cFLIPL mRNA | Pahl, 1999 [18] |
| T35 | NF-κB initiates gene expression of BCL-2 | Pahl, 1999 [18] |
| T36 | Transcription of BCL-2 mRNA | Pahl, 1999 [18] |
| T37 | IκB binding to NF-κB | Brown *et al*, 1993 [1] |
| T38 | Translocation of IκB into the nucleus | Brown *et al*, 1993 [1] |
| T39 | IκB binds NF-κB in the nucleus | Brown *et al*, 1993 [1] |
| T40 | Translocation of the complex of NF-κB and IκB into the cytosol | Brown *et al*, 1993 [1] |
| T41 | Destabilization of complex I by A20 | Draber *et al*, 2015 [6] |
| T42 | TRADD getting cytosolic | Dickens *et al,* 2012 [4] |
| T43 | FADD binding to TRADD | Dickens *et al,* 2012 [4] |
| T44 | Procaspase8bindingtoFADDtoformcomplex IIa | Dickens *et al,* 2012 [4] |
| T45 | Dimerization of procaspase 8 in complex IIa | Dickens *et al,* 2012 [4] |
| T46 | Caspase 8 activation by complex IIa | Reed & Green, 2011 [20] |
| T47 | cFLIPL binding to complex IIa | Dillon *et al*, 2012 [5]  Tsuchiya *et al*, 2015 [26] |
| T48 | TRADD and RIP1 getting cytosolic | Dickens *et al,* 2012 [4] |
| T49 | Dissociation of RIP1 |  |
| T50 | Dissociation of TRADD |  |
| T51 | Binding of RIP3 to RIP1 | Sun *et al*, 2002 [23] |
| T52 | Phosphoryation of MLKL by RIP3 in the necrosome | Murphy *et al*, 2013 [14]  Vanden Berghe *et al*, 2014 [27] |
| T53 | FADD binding to RIP1 | Dickens *et al,* 2012 [4] |
| T54 | Recruitment of procapase 8 to form complex IIb | Dickens *et al.,* 2012 [4] |
| T55 | cFLIPS bound to complex IIb | Feokistova *et al*, 2011 [7] |
| T56 | BindingofRIP3tocomplexIIbwithcFLIPS | Feokistova *et al*, 2011 [7] |
| T57 | Phosporylation of MLKL by complex IIb with cFLIPS and RIP3 | Feokistova *et al*, 2011 [7] |
| T58 | Translocation of phosporylated MLKL to the plasma membrane | Feokistova *et al*, 2011 [7] |
| T59 | cFLIPL binding to complex IIb | Oberst *et al*, 2011 [15] |
| T60 | Local cleavage of RIP1 and RIP3 by CASP8 bound to cFLIPL | Oberst *et al*, 2011 [15] |
| T61 | Dimerization of procaspase 8 in complex IIb | Reed & Green, 2011 [20] |
| T62 | Activation of CASP8 and dissociation of complex IIb | Reed & Green, 2011 [20] |
| T63 | Direct cleavage of procaspase 3 by CASP8 | Reed & Green, 2011 [20] |
| T64 | XIAP binds and inhibits caspase 3 | Gyrd-Hansen & Meier, 2010 [9] |
| T65 | Cleavage of BID to tBID by CASP8 | Chipuk & Green, 2008 [3]  Ola *et al*, 2011 [17] |
| T66 | Translocation of tBID to the mitochondrial outer membrane | Chipuk & Green, 2008 [3]  Ola *et al*, 2011 [17] |
| T67 | Binding of BAX to tBID | Chipuk & Green, 2008 [3]  Ola *et al*, 2011 [17] |
| T68 | Inhibition of BAX by BCL-2 and inhibition of apoptosis | Shore & Nguyen, 2008 [22]  Chipuk & Green, 2008 [3]  Ola *et al*, 2011 [17] |
| T69 | BAX oligomerization in the mitochondrial outer membrane | Chipuk & Green, 2008 [3]  Ola *et al*, 2011 [17] |
| T70 | BAX pore formation | Chipuk & Green, 2008 [3]  Ola *et a*, 2011 [17] |
| T71 | Release of mitochondrial proteins cyt c and SMAC | Reed & Green, 2011 [20]  Ola *et al*, 2011 [17] |
| T72 | Apaf1 and procaspase 9 form the apoptosome with cyt c | Würstle *et al*, 2012 [31] |
| T73 | Dimerization of procaspase 9 in the apoptosome | Würstle *et al*, 2012 [31] |
| T74 | Cleavage of procaspase 3 by CASP9 | Würstle *et al*, 2012 [31] |
| T75 | XIAP interaction with procaspase 9 in the apoptosome | Gyrd-Hansen & Meier, 2010 [9] |
| T76 | SMAC binds and inhibits XIAP | Verhagen *et al*, 2000 [29]  Ola *et al*, 2011 [17] |

## References

1. Brown K, Park S, Kanno T, Franzoso G, Siebenlist U. Mutual regulation of the transcriptional activator NF-kappa B and its inhibitor, I kappa B-alpha. Proceedings of the National Academy of Sciences.1993;90(6):2532–2536.
2. Cai Z, Jitkaew S, Zhao J, Chiang H-C, Choksi S, Liu J, Ward Y, et al. Plasma membrane translocation of trimerized MLKL protein is required for TNF-induced necroptosis. Nat Cell Biol. 2014;16(3):55–65.
3. Chipuk JE, Green DR. How do BCL-2 proteins induce mitochondrial outer membrane permeabilization? Trends Cell Biol. 2008;18(4):157–164.
4. Dickens L, Powley I, Hughes M, MacFarlane M. The ‘complexities‘ of life and death: Death receptor signalling platforms. Exp Cell Res. 2012;318(11):1269–1277.
5. Dillon CP, Oberst A, Weinlich R, Janke LJ, Kang T-B, Ben-Moshe T, et al. Survival Function of the FADD-CASPASE-8-cFLIPL Complex. Cell Reports. 2012;1(5):401-407.
6. Draber P, Kupka S, Reichert M, Draberova H, Lafont E, de Miguel D et al. LUBAC-recruited CYLD and A20 regulate gene activation and cell death by exerting opposing effects on linear ubiquitin in signaling complexes. Cell Rep. 2015;13(10):2258–2272.
7. Feokistova M, Geserick P, Kellert B, Dimitrova DP, Langlais C, Hupe M, et al. cIAPs Block Ripoptosome Formation, a RIP1/Caspase-8 Containing Intracellular Cell Death Complex Differentially Regulated by cFLIP Isoforms. Mol Cell. 2011;43(3):449-463.
8. Galban S & Duckett CS. XIAP as a ubiquitin ligase in cellular signaling. Cell Death Differ. 2010;7(1):54–60.
9. Gyrd-Hansen M, Meier P. IAPs: from caspase inhibitors to modulators of NF-κB, inflammation and cancer. Nat Rev Cancer. 2010;10(8):561–574.
10. Haas TL, Emmerich CH, Gerlach B, Schmukle AC, Cordier SM, Rieser E, et al. Recruitment of the Linear Ubiquitin Chain Assembly Complex Stabilizes the TNFR1 Signaling Complex and Is Required for TNF-Mediated Gene Induction. Mol Cell. 2009;36(5):831–844.
11. Kensche T, Tokunaga F, Ikeda F, Goto E, Iwai K, Dikic I. Analysis of Nuclear Factor- κB (NF-κB) essential modulator (NEMO) binding to linear and lysine-linked ubiquitin chains and its role in the activation of NF-κB. J Biol Chem. 2012;287:23626-23634.
12. Kovalenko A, Chable-Bessia C, Cantarella G, Israël A, Wallach D, Courtois G. The tumor suppressor CYLD negativaly regulates NF-κB signaling by deubiquitination. Nature. 2003;424(6950):801–805.
13. Micheau O., Lens S, Gaide O, Alevizopoulos K, Tschopp J. NF-κB signals induce the expression of c-FLIP. Mol Cell Biol. 2001;21(16):5299–5305.
14. Murphy JM, Czabotar PE, Hildebrand JM, Lucet IS, Zhang J-G, Alvarez-Diaz S et al. The pseudokinase MLKL mediates necroptosis via a molecular switch mechanism. Immunity. 2013;39(3):443–453.
15. Oberst A, Dillon CP, Weinlich R, McCormick LL, Fitzgerald P, Pop C, et al. Catalytic activity of the caspase-8-FLIPL complex inhibits RIPK3-dependent necrosis. Nature. 2011;471(7338):363-367.
16. Oeckinghaus A, Ghosh S. The NF-κB Family of Transcription Factors and Its Regulation. Cold Spring Harbor Perspect Biol. 2009;1(4):a000034. Available from: https://doi.org/10.1101/cshperspect.a000034
17. Ola MS, M. Nawaz M, Ahsan H. Role of Bcl-2 family proteins and caspases in the regulation of apoptosis. Mol Cell Biochem. 2011;351(1-2):41–58.
18. Pahl H. Activators and target genes of Rel/NF-κB transcription factors. Nat Oncogene. 1999;18(49):6853–6866.
19. Peltzer N, Darding M, Walczak H. Holding RIPK1 on the Ubiquitin Leash in TNFR1 Signaling. Trends Cell Biol. 2016;26:445-461.
20. Reed JC, Green DR (editors). Apoptosis: Physiology and Pathology. Cambridge, UK: Cambridge University Press; 2011.
21. Schile AJ, García-Fernández M, Steller H. Regulation of apoptosis by XIAP ubiquitin-ligase activity. Genes Dev. 2008;22(16):2256–2266.
22. Shore GC, Nguyen M. Bcl-2 proteins and apoptosis: Choose your partner. Cell. 2008;135(6):1004-1006.
23. Sun X, Yin J, Starovasnik MA, Fairbrother WJ, Dixit VM. Identification of a novel homotypic interaction motif required for the phosphorylation of RIP (receptor-interacting protein) by RIP3. J Biol Chem. 2002;277(11):9505–9511.
24. Taylor RC, Cullen SP, Martin SJ. Apoptosis: controlled demolition at the cellular level. Nat Rev Mol Cell Biol. 2008;9(3):231–241.
25. Ting AT, Bertrand MJM. More to Life than NF-κB in TNFR1 Signaling. Trends Immunol. 2016;37(8):535-545.
26. Tsuchiya Y, Nakabayashi O, Nakano H. FLIP the Switch: Regulation of Apoptosis and Necroptosis by cFLIP. Int J Mol Sci. 2015;16(12):30321-30341.
27. Vanden Berghe T, Linkermann A, Jouan-Lanhouet S, Walczak H, Vandenabeele P. Regulated necrosis: the expanding network of nonapoptotic cell death pathways. Nat Rev Mol Cell Biol. 2014;15(2):135–147.
28. Varfolomeev E, Vucic D. Intracellular regulation of TNF activity in health and disease. Cytokine. 2018;101:26–32, 2018.
29. Verhagen AM, Ekert PG, Pakusch M, Silke J, Connolly LM, Reid GE, et al. Identification of DIABOLO, a Mammalian Protein that Promotes Apoptosis by Binding to and Antagonizing IAP Proteins. Cell. 2000;102(1):43–53.
30. Wang H, Sun L, Su L, Rizo J, Liu L, Wang L-F, et al. Mixed lineage kinase domain-like protein MLKL causes necrotic membrane disruption upon phosphorylation by RIP3. Mol Cell. 2014;54(1):133–146.
31. Würstle ML, Laussmann MA, Rehm M. The central role of initiator caspase-9 in apoptosis signal transduction and the regulation of its activation and activity on the apoptosome. Exp Cell Res. 2012;318(11):1213–1220.
32. Zhang J, Clark K, Lawrence T, Peggie MW, Cohen P. An unexpected twist to the activation of IKKβ: TAK1 primes IKKβ for activation by autophosphorylation. Biochem J. 2014;461(3):531–537.
