# Supplementary material for: Mathematical modeling of the molecular switch of TNFR1-mediated signaling pathways applying Petri net formalism and in silico knockout analysis
Source: PLoS Comput Biol. 2022 Aug 22;18(8):e1010383. doi: 10.1371/journal.pcbi.1010383 (PMC9467317; doi:10.1371/journal.pcbi.1010383)
Supplement: S2 Table — For transitions, references and specifications of cell lines that have been used in relevant in vivo experiments are given. (DOCX) [file pcbi.1010383.s003.docx]

**S2 Table:** Cell line specificities of transitions and references to experiments. For transitions, references and specifications of cell lines that have been used in relevant *in vivo* experiments are given.

| **Transition name** | **References** |  |
| --- | --- | --- |
| Apoptosis | Reed & Green, 2011 [20]  Taylor *et al*, 2008 [24] | Mammalian (mainly), review  Human, HeLa cells, review |
| Apo_XIAP_inhib | Galban & Duckett, 2010 [8]  Verhagen *et al*, 2000 [29] | Human, mice, mammals  Human, mouse, mammalian |
| BAX_inhib | Shore & Nguyen, 2008 [22]  Ola *et al*, 2011 [17] | Mammalian  Mammalian, review |
| CASP3_inhib | Gyrd-Hansen & Meier, 2010 [9]  Schile *et al*, 2008 [21] | Mammalian  Human, mice, mammalian |
| CI_diss, T18, T19, T41 | Draber *et al*, 2015 [6] | Human, mice, K562 cells, A549 cells HeLa cells |
| CIIa_inhib, Pc8_inhib, T47 | Dillon *et al*, 2012 [5]  Tsuchiya *et al*, 2015 [26] | Human, mice  Human, mice, mammalian, review |
| Necroptosis | Wang *et al*, 2014 [30]  Cai *et al*, 2014 [2] | Human liver cells, mice  Human |
| Pc9_inhib | Gyrd-Hansen & Meier, 2010 [9] | Mammalian |
| RIP1:RIP3_inhib | Oberst *et al*, 2011 [15]  Tsuchiya *et al*, 2015 [26] | Mice  Human, mice, mammalian, review |
| T1, T2, T3, T4, T5, T6 | Ting & Bertrand, 2016 [25]  Varfolomeev & Vucic, 2018 [28] | Human, mice  Mammalian |
| T7, T8, T9, T10, T11, T12, T15, T16, T17 | Kensche *et al*, 2012 [11] | Human |
| T13 | Zhang *et al*, 2014 [32]  Peltzer *et al*, 2016 [19] | Human, mice  Mammalian, review |
| T14 | Haas *et al*, 2009 [10]  Peltzer *et al*, 2016 [19] | Human, HeLa cells  Mammalian, review |
| T20, T21 | Draber *et al,* 2015 [6]  Kovalenko *et al*, 2003 [12] | Human, K562 and HeLa cells  Human |
| T22, T25 | Varfolomeev & Vucic, 2018 [28] | Mammalian |
| T23 | Brown *et al*., 1993 [1]  Varfolomeev & Vucic, 2018 [28] | Human T lymphocytes and monocytes,  mammalian |
| T24 | Oeckinghaus & Ghosh, 2009 [16]  Varfolomeev & Vucic, 2018 [28] | Human, mice, mammalian  Human |
| T26, T27 | Pahl, 1999 [18]  Brown *et al*, 1993 [1] | Human  Human |
| T33 | Micheau *et al*, 2001 [13] | Human, HeLa and HT1080 (human fibrosarcoma) cells |
| T37, T38, T39, T40 | Brown *et al*, 1993 [1] | Human T lymphocytes and monocytes |
| T42, T43, T44,T45, T48, T53, T54 | Dickens *et al,* 2012 [4] | Human, mammalian |
| T46 | Reed & Green, 2011 [20] | Mammalian (mainly), review |
| T51 | Sun *et al*, 2002 [23] | Human, mice |
| T52 | Murphy *et al*, 2013 [14]  Vanden Berghe *et al*, 2014 [27] | Mice  Human, mammalian, review |
| T55, T56, T57, T58 | Feokistova *et al*, 2011 [7] | Human, mammalian |
| T59, T60 | Oberst *et al*, 2011 [15] | Human, mice |
| T61, T62, T63 | Reed & Green, 2011 [20] | Mammalian (mainly), review |
| T64 | Gyrd-Hansen & Meier, 2010 [9] | Mammalian |
| T65, T66, T67, T69, T70 | Chipuk & Green, 2008 [3]  Ola *et al*, 2011 [17] | Human, mammalian  Mammalian, review |
| T68 | Shore & Nguyen, 2008 [22]  Chipuk & Green, 2008 [3]  Ola *et al*, 2011 [17] | Mammalian  Human, mammalian  Mammalian, review |
| T71 | Reed & Green, 2011 [20]  Ola *et al*, 2011 [17] | Mammalian (mainly), review  Mammalian, review |
| T72, T73, T74 | Würstle *et al*, 2012 [31] | Mammalian |
| T75 | Gyrd-Hansen & Meier, 2010 [9] | Mammalian |
| T76 | Verhagen *et al*, 2000 [29]  Ola *et al*, 2011 [17] | Human, mouse, mammalian  Mammalian, review |

## References

1. Brown K, Park S, Kanno T, Franzoso G, Siebenlist U. Mutual regulation of the transcriptional activator NF-kappa B and its inhibitor, I kappa B-alpha. Proceedings of the National Academy of Sciences.1993;90(6):2532–2536.
2. Cai Z, Jitkaew S, Zhao J, Chiang H-C, Choksi S, Liu J, Ward Y, et al. Plasma membrane translocation of trimerized MLKL protein is required for TNF-induced necroptosis. Nat Cell Biol. 2014;16(3):55–65.
3. Chipuk JE, Green DR. How do BCL-2 proteins induce mitochondrial outer membrane permeabilization? Trends Cell Biol. 2008;18(4):157–164.
4. Dickens L, Powley I, Hughes M, MacFarlane M. The ‘complexities‘ of life and death: Death receptor signalling platforms. Exp Cell Res. 2012;318(11):1269–1277.
5. Dillon CP, Oberst A, Weinlich R, Janke LJ, Kang T-B, Ben-Moshe T, et al. Survival Function of the FADD-CASPASE-8-cFLIPL Complex. Cell Reports. 2012;1(5):401-407.
6. Draber P, Kupka S, Reichert M, Draberova H, Lafont E, de Miguel D et al. LUBAC-recruited CYLD and A20 regulate gene activation and cell death by exerting opposing effects on linear ubiquitin in signaling complexes. Cell Rep. 2015;13(10):2258–2272.
7. Feokistova M, Geserick P, Kellert B, Dimitrova DP, Langlais C, Hupe M, et al. cIAPs Block Ripoptosome Formation, a RIP1/Caspase-8 Containing Intracellular Cell Death Complex Differentially Regulated by cFLIP Isoforms. Mol Cell. 2011;43(3):449-463.
8. Galban S & Duckett CS. XIAP as a ubiquitin ligase in cellular signaling. Cell Death Differ. 2010;7(1):54–60.
9. Gyrd-Hansen M, Meier P. IAPs: from caspase inhibitors to modulators of NF-κB, inflammation and cancer. Nat Rev Cancer. 2010;10(8):561–574.
10. Haas TL, Emmerich CH, Gerlach B, Schmukle AC, Cordier SM, Rieser E, et al. Recruitment of the Linear Ubiquitin Chain Assembly Complex Stabilizes the TNFR1 Signaling Complex and Is Required for TNF-Mediated Gene Induction. Mol Cell. 2009;36(5):831–844.
11. Kensche T, Tokunaga F, Ikeda F, Goto E, Iwai K, Dikic I. Analysis of Nuclear Factor- κB (NF-κB) essential modulator (NEMO) binding to linear and lysine-linked ubiquitin chains and its role in the activation of NF-κB. J Biol Chem. 2012;287:23626-23634.
12. Kovalenko A, Chable-Bessia C, Cantarella G, Israël A, Wallach D, Courtois G. The tumor suppressor CYLD negativaly regulates NF-κB signaling by deubiquitination. Nature. 2003;424(6950):801–805.
13. Micheau O., Lens S, Gaide O, Alevizopoulos K, Tschopp J. NF-κB signals induce the expression of c-FLIP. Mol Cell Biol. 2001;21(16):5299–5305.
14. Murphy JM, Czabotar PE, Hildebrand JM, Lucet IS, Zhang J-G, Alvarez-Diaz S et al. The pseudokinase MLKL mediates necroptosis via a molecular switch mechanism. Immunity. 2013;39(3):443–453.
15. Oberst A, Dillon CP, Weinlich R, McCormick LL, Fitzgerald P, Pop C, et al. Catalytic activity of the caspase-8-FLIPL complex inhibits RIPK3-dependent necrosis. Nature. 2011;471(7338):363-367.
16. Oeckinghaus A, Ghosh S. The NF-κB Family of Transcription Factors and Its Regulation. Cold Spring Harbor Perspect Biol. 2009;1(4):a000034. Available from: https://doi.org/10.1101/cshperspect.a000034
17. Ola MS, M. Nawaz M, Ahsan H. Role of Bcl-2 family proteins and caspases in the regulation of apoptosis. Mol Cell Biochem. 2011;351(1-2):41–58.
18. Pahl H. Activators and target genes of Rel/NF-κB transcription factors. Nat Oncogene. 1999;18(49):6853–6866.
19. Peltzer N, Darding M, Walczak H. Holding RIPK1 on the Ubiquitin Leash in TNFR1 Signaling. Trends Cell Biol. 2016;26:445-461.
20. Reed JC, Green DR (editors). Apoptosis: Physiology and Pathology. Cambridge, UK: Cambridge University Press; 2011.
21. Schile AJ, García-Fernández M, Steller H. Regulation of apoptosis by XIAP ubiquitin-ligase activity. Genes Dev. 2008;22(16):2256–2266.
22. Shore GC, Nguyen M. Bcl-2 proteins and apoptosis: Choose your partner. Cell. 2008;135(6):1004-1006.
23. Sun X, Yin J, Starovasnik MA, Fairbrother WJ, Dixit VM. Identification of a novel homotypic interaction motif required for the phosphorylation of RIP (receptor-interacting protein) by RIP3. J Biol Chem. 2002;277(11):9505–9511.
24. Taylor RC, Cullen SP, Martin SJ. Apoptosis: controlled demolition at the cellular level. Nat Rev Mol Cell Biol. 2008;9(3):231–241.
25. Ting AT, Bertrand MJM. More to Life than NF-κB in TNFR1 Signaling. Trends Immunol. 2016;37(8):535-545.
26. Tsuchiya Y, Nakabayashi O, Nakano H. FLIP the Switch: Regulation of Apoptosis and Necroptosis by cFLIP. Int J Mol Sci. 2015;16(12):30321-30341.
27. Vanden Berghe T, Linkermann A, Jouan-Lanhouet S, Walczak H, Vandenabeele P. Regulated necrosis: the expanding network of nonapoptotic cell death pathways. Nat Rev Mol Cell Biol. 2014;15(2):135–147.
28. Varfolomeev E, Vucic D. Intracellular regulation of TNF activity in health and disease. Cytokine. 2018;101:26–32, 2018.
29. Verhagen AM, Ekert PG, Pakusch M, Silke J, Connolly LM, Reid GE, et al. Identification of DIABOLO, a Mammalian Protein that Promotes Apoptosis by Binding to and Antagonizing IAP Proteins. Cell. 2000;102(1):43–53.
30. Wang H, Sun L, Su L, Rizo J, Liu L, Wang L-F, et al. Mixed lineage kinase domain-like protein MLKL causes necrotic membrane disruption upon phosphorylation by RIP3. Mol Cell. 2014;54(1):133–146.
31. Würstle ML, Laussmann MA, Rehm M. The central role of initiator caspase-9 in apoptosis signal transduction and the regulation of its activation and activity on the apoptosome. Exp Cell Res. 2012;318(11):1213–1220.
32. Zhang J, Clark K, Lawrence T, Peggie MW, Cohen P. An unexpected twist to the activation of IKKβ: TAK1 primes IKKβ for activation by autophosphorylation. Biochem J. 2014;461(3):531–537.
