# Supplementary material for: Mathematical modeling of the molecular switch of TNFR1-mediated signaling pathways applying Petri net formalism and in silico knockout analysis
Source: PLoS Comput Biol. 2022 Aug 22;18(8):e1010383. doi: 10.1371/journal.pcbi.1010383 (PMC9467317; doi:10.1371/journal.pcbi.1010383)
Supplement: S3 Table — For each place, its name and biological meaning are given. For abbreviations applied to name places, we refer to S4 Table. (DOCX) [file pcbi.1010383.s004.docx]

**S3 Table:** List of 118 places. For each place, its name and biological meaning are given. For abbreviations applied to name places, we refer to S4 Table.

| **Name of place** | **Biological meaning** |
| --- | --- |
| Apaf1 | Apaf1 |
| Apoptosome | Apoptosome |
| Apoptosome_diss | Dissociated apoptosome |
| A20 | A20 |
| A20_g | Gene of A20 |
| A20_mRNA | mRNA of A20 |
| BAX | BAX |
| BAX:BAX_pore | Pore of BAX oligomers in the outer mitochondrial membrane |
| BCL-2 | BCL-2 |
| BCL-2:BAX | Inhibitory complex of BCL-2 and BAX |
| BCL-2_g | Gene of BCL-2 |
| BCL-2_mRNA | mRNA of BCL-2 |
| Bid | BID |
| CASP3 | Activated CASP3 |
| CASP8 | Activated CASP8 |
| CASP8_diss | Dissociated CASP8 |
| CASP9 | Activated CASP9 |
| CASP9_diss | Dissociated CASP9 |
| cFLIP_g | Gene of cFLIP |
| cFLIPL | cFLIPL |
| cFLIPL_mRNA | mRNA of cFLIPL |
| cFLIPs | cFLIPS |
| CI | Complex I |
| cIAP | cIAP proteins 1 and 2 |
| CI:A20 | A20 bound to complex I |
| CI:CYLD | CYLD bound to complex I |
| CI_diss | Dissociation of complex I |
| CI:N:I | Complex I interaction with the inhibitory complex of NF-κB and I κB |
| CIIa | Complex IIa |
| CIIa:cFLIPL | cFLIPL bound to complex IIa |
| CIIa_diss | Dissociation of complex IIa |
| CIIa:Pc8_2 | Dimer of procaspase 8 bound to complex IIa |
| CIIb | Complex IIb |
| CIIb:cFLIPL | cFLIPL bound to complex IIb |
| CIIb:cFLIPs | cFLIPS bound to complex IIb |
| CIIb:cFs:RIP3 | RIP3 bound to the complex IIb with cFLIPS |
| CIIb_diss | Dissociation of complex IIb |
| CIIb:Pc8_2 | Dimer of procaspase 8 bound to complex IIb |
| CYLD | CYLD |
| Cyt c | Cyt c |
| Cyt c_mito | Cyt c in the mitochondrial intermembrane space |
| FADD | FADD |
| IkB | IκB |
| IkB_g | Gene of IκB |
| IkB_K48ub | IκB tagged with a K48 Ub chain |
| IkB_mRNA | mRNA of IκB |
| IkB_n | Nuclear IκB |
| IkB_p | Phosphorylated IκB |
| IKK | IKKα/ IKKβ |
| K63ub:L:CYLD | CYLD binding to LUBAC |
| K63ub:LUBAC | LUBAC binding to K63 Ub chains |
| K63ub:LUBAC:M1ub:NEMO | NEMO binding to M1 Ub chains |
| K63ub:NEMO | NEMO binding to K63 Ub chains |
| K63ub:TAB | TAB2/3 binding to K63 Ub chains |
| LUBAC | LUBAC |
| LUBAC_K63ub:CYLD | CYLD recruitment to LUBAC in complex I |
| MLKL | MLKL |
| MLKL_p | Phosphorylated MLKL |
| MLKL_PM | MLKL at the plasma membrane |
| NEMO | NEMO |
| NEMO:IKK_K63ub | IKKα/β recruitment by K63 Ub chains to complex I |
| NEMO:IKK_M1ub | IKKα/β recruitment by M1 Ub chains to complex I |
| NEMO_K63ub | NEMO recruitment by K63 Ub chains to complex I |
| NEMO_M1ub | NEMO recruitment by M1 Ub chains to complex I |
| NF-kB | NF-κB |
| NF-kB:IkB | Inhibitory complex of NF-κB and I κB |
| NF-kB:IkB_n | Inhibitory complex of NF-κB and I κB in the nucleus |
| NF-kB_n | Nuclear NF-κB |
| NF-kB_n2 | Nuclear NF-κB late |
| NF-kB_n:A20_g | Nuclear NF-κB bound to the gene of A20 |
| NF-kB_n:IkB_g | Nuclear NF-κB bound to the gene of IκB |
| NF-kB_n2:BCL-2_g | Nuclear NF-κB bound to the gene of BCL-2 |
| NF-kB_n2:cFLIP_g | Nuclear NF-κB bound to the gene of cFLIP |
| NF-kB_n2:XIAP_g | Nuclear NF-κB bound to the gene of XIAP |
| Pc8:cFLIPL | Inhibitory complex of procaspase 8 and cFLIPL |
| Procasp3 | Procaspase 3 |
| Procasp8 | Procaspase 8 |
| Procasp9 | Procaspase 9 |
| RIP1 | RIP1 |
| RIP1_c | Cytosolic RIP1 |
| RIP1:RIP3 | RIP1 and RIP3 form the necrosome |
| RIP1:RIP3_cl | Cleaved RIP1 and RIP3 |
| RIP3 | RIP3 |
| RIP_diss | Dissociated RIP1 |
| R:FADD | FADD bound to RIP1 |
| R:R_diss | Dissociation of RIP1 and RIP3 |
| SCF | SCF-^βTrCP^ |
| SMAC | SMAC |
| SMAC_mito | SMAC in the mitochondrial intermembrane space |
| SMAC:XIAP:Pc9 | SMAC bound to XIAP with procaspase 9 |
| TAB | TAB2/3 |
| TAB_K63ub | TAB2/3 recruitment by K63 Ub chains to complex I |
| TAK1 | TAK1 |
| TAB:TAK_K63ub | TAK recruitment by K63 Ub chains to complex I |
| tBid | tBID |
| tBid:BAX | BAX bound to tBID |
| tBid:BAX_2 | Dimer of BAX bound to tBID |
| tBid_MOM | tBID in the mitochondrial outer membrane |
| TNF | TNF-α |
| TNFR1 | TNFR1 |
| TNFR1:TNF | TNF-α bound to TNFR1 |
| TRADD | TRADD |
| TRADD_c | Cytosolic TRADD |
| TRADD_diss | Dissociation of TRADD |
| TRADD:RIP1 | RIP1 bound to TRADD |
| TRAF2 | TRAF2 |
| T:FADD | FADD bound to TRADD |
| T:T_diss | Dissociation of TNFR1 and TNF-α |
| T:T:TRADD | TRADD bound to TNFR1 |
| T:T:T:RIP | RIP1 bound to TRADD in complex I |
| T:T:T:R:TRAF2 | TRAF2 bound to complex I |
| T:T:T:R:T:cIAP | cIAP1/2 bound to TRAF2 in complex I |
| T:T:T:R:T:c_K63ub | K63 ubiquitinated complex I |
| XIAP | XIAP |
| XIAP:CASP3 | XIAP bound to CASP3 |
| XIAP_g | Gene of XIAP |
| XIAP_mRNA | mRNA of XIAP |
| XIAP:Pc9 | XIAP bound to procaspase 9 |
|  |  |
