# Supplementary material for: Mathematical modeling of the molecular switch of TNFR1-mediated signaling pathways applying Petri net formalism and in silico knockout analysis
Source: PLoS Comput Biol. 2022 Aug 22;18(8):e1010383. doi: 10.1371/journal.pcbi.1010383 (PMC9467317; doi:10.1371/journal.pcbi.1010383)
Supplement: S4 Table — (DOCX) [file pcbi.1010383.s005.docx]

**S4 Table:** List of abbreviations applied to name places, see S3 Table and the Petri net of TNFR1 signal transduction in Fig 2.

| **Abbreviations** | **Biological meaning** |
| --- | --- |
| c | cytosolic |
| cl | cleavage |
| deg | degradation |
| diss | dissociation |
| g | gene |
| inhib | inhibition |
| K48ub | ubiquitinated with K48-linked Ub chains |
| K63ub | ubiquitinated with K63-linked Ub chains |
| mito | mitochondrial |
| MOM | Mitochondrial outer membrane |
