# Supplementary material for: Mathematical modeling of the molecular switch of TNFR1-mediated signaling pathways applying Petri net formalism and in silico knockout analysis
Source: PLoS Comput Biol. 2022 Aug 22;18(8):e1010383. doi: 10.1371/journal.pcbi.1010383 (PMC9467317; doi:10.1371/journal.pcbi.1010383)
Supplement: S5 Table — For each TI, the number, the names of the transitions, and the biological meaning are given. The number is highlighted by bold face if the TI covers a dissected pathway. 33 TIs represent dissected pathways. An example of a dissected pathway is TI9. TI9 induces A20 feedback regulation but ignores the interrelation of this process with a necessary activation of NF-κB, see text. (DOCX) [file pcbi.1010383.s006.docx]

**S5 Table:** List of 48 transition invariants (TIs). For each TI, the number, the names of the transitions, and the biological meaning are given. The number is highlighted by bold face if the TI covers a dissected pathway. 33 TIs represent dissected pathways. An example of a dissected pathway is TI_9_. TI_9_ induces A20 feedback regulation but ignores the interrelation of this process with a necessary activation of NF-κB, see text.

| **TI** | **Transitions** | **Biological meaning** |
| --- | --- | --- |
| 1 | Apoptosis, deg3, Syn_TNFR1, Syn_TNF, Syn_TRADD, Syn_RIP1, Syn_TRAF2, Syn_cIAP, Syn_TAB, Syn_TAK1, 2 Syn_NEMO, 2 Syn_IKK, Syn_LUBAC, Syn_FADD, 2 Syn_Procasp8, T1, T2, T3, T4, T5, T14, T7, T15, T8, T16, T9, T17, T13, T63, T10, T11, T12, T18, T19, Syn_CYLD, diss1, T21, T20, T43, T44, T45, T46, T49, deg1, diss3, T6, Syn_Procasp3 | Formation of complex I and dissociation via CYLD, induction of apoptosis via complex IIa and extrinsic activation of caspase 3 |
| 2 | Apoptosis, deg3, Syn_TNFR1, Syn_TNF, Syn_TRADD, Syn_RIP1, Syn_TRAF2, Syn_cIAP, Syn_TAB, Syn_TAK1, 2 Syn_NEMO, 2 Syn_IKK, Syn_LUBAC, Syn_FADD, 2 Syn_Procasp8, T1, T2, T3, T4, T5, T14, T7, T15, T8, T16, T9, T17, T13, T63, T10, T11, T12, T18, T19, Syn_CYLD, diss1, T21, T20, T53, T54, T61, T62, T50, deg2, diss5, T6, Syn_Procasp3 | Formation of complex I and dissociation via CYLD, induction of apoptosis via complex IIb and extrinsic activation of caspase 3 |
| 3 | Apoptosis, deg3, Syn_TNFR1, Syn_TNF, Syn_TRADD, Syn_RIP1, Syn_FADD, 2 Syn_Procasp8, T1, T2, T3, T63, T48, diss2, T43, T44, T45, T46, T49, deg1, diss3, Syn_Procasp3 | Dissociation of TRADD and RIP1 and extrinsic induction of apoptosis via complex IIa |
| **4** | Syn_TNFR1, Syn_TNF, Syn_TRADD, Syn_FADD, Syn_Procasp8, T1, T2, T33, T34, diss2, T42, T43, T44, T47, CIIa_inhib, Syn_cFLIPL, diss3 | Dissociation of TRADD, formation of complex IIa and inhibition of apoptosis by cFLIP_L_ |
| 5 | Apoptosis, deg3, Syn_TNFR1, Syn_TNF, Syn_TRADD, Syn_FADD, 2 Syn_Procasp8, T1, T2, T63, diss2, T42, T43, T44, T45, T46, diss3, Syn_Procasp3 | Dissociation of TRADD and induction of apoptosis via complex IIa |
| 6 | Apoptosis, deg3, Syn_TNFR1, Syn_TNF, Syn_TRADD, Syn_RIP1, Syn_FADD, 2 Syn_Procasp8, T1, T2, T3, T63, T48, diss2, T53, T54, T61, T62, T50, deg2, diss5, Syn_Procasp3 | Dissociation of RIP1 and TRADD and and induction of apoptosis via complex IIb and the extrinsic pathway |
| **7** | deg3, Syn_TNFR1, Syn_TNF, Syn_TRADD, Syn_RIP1, Syn_TRAF2, Syn_cIAP, Syn_TAB, Syn_TAK1, 2 Syn_NEMO, 2 Syn_IKK, Syn_LUBAC, Syn_FADD, 2 Syn_Procasp8, T1, T2, T3, T4, T5, T14, T64, CASP3_inhib, T7, T15, T8, T16, T9, T17, T13, T31, T32, T63, T10, T11, T12, T18, T19, Syn_CYLD, diss1, T21, T20, T43, T44, T45, T46, T49, deg1, Syn_XIAP, diss3, T6, Syn_Procasp3 | Formation of complex I and dissociation via CYLD, formation of complex IIa and inhibition of caspase 3 extrinsic activation by XIAP |
| **8** | deg3, Syn_TNFR1, Syn_TNF, Syn_TRADD, Syn_RIP1, Syn_TRAF2, Syn_cIAP, Syn_TAB, Syn_TAK1, 2 Syn_NEMO, 2 Syn_IKK, Syn_LUBAC, Syn_FADD, 2 Syn_Procasp8, T1, T2, T3, T4, T5, T14, T64, CASP3_inhib, T7, T15, T8, T16, T9, T17, T13, T31, T32, T63, T10, T11, T12, T18, T19, Syn_CYLD, diss1, T21, T20, T53, T54, T61, T62, T50, deg2, Syn_XIAP, diss5, T6, Syn_Procasp3 | Formation of complex I and dissociation via CYLD, formation of complex IIb and inhibition of caspase 3 extrinsic activation by XIAP |
| **9** | Syn_TNFR1, Syn_TNF, Syn_TRADD, Syn_RIP1, Syn_TRAF2, Syn_cIAP, Syn_TAB, Syn_TAK1, 2 Syn_NEMO, 2 Syn_IKK, Syn_LUBAC, T1, T2, T3, T4, T5, T14, T7, T15, T8, T16, T9, T17, T13, T28, T29, T41, CI_diss, T10, T11, T12, Syn_A20, T6 | Formation of complex I and dissociation via A20 |
| 10 | Syn_TNFR1, Syn_TNF, Syn_TRADD, Syn_RIP1, Syn_FADD, Syn_Procasp8, Syn_cFLIPs, Syn_RIP3, Syn_MLKL, T1, T2, T3, T58, T48, diss2, T53, T54, T55, T56, T57, Necroptosis, T50, deg2, diss5 | Dissociation of TRADD and RIP1, formation of complex IIbandnecroptosisinduction via inhibition by cFLIP_S_ |
| **11** | deg3, Syn_TNFR1, Syn_TNF, Syn_TRADD, Syn_RIP1, Syn_FADD, 2∗Syn_Procasp8, T1, T2, T3, T64, CASP3_inhib, T31, T32, T63, T48, diss2, T43, T44, T45, T46, T49, deg1, Syn_XIAP, diss3, Syn_Procasp3 | Dissociation of TRADD and RIP1, formation of complex IIa and inhibition of extrinsic apoptosis induction via XIAP |
| **12** | deg3, Syn_TNFR1, Syn_TNF, Syn_TRADD, Syn_FADD, 2 Syn_Procasp8, T1, T2, T64, CASP3_inhib, T31, T32, T63, diss2, T42, T43, T44, T45, T46, Syn_XIAP, diss3, Syn_Procasp3 | Dissociation of TRADD and formation of complex IIa, inhibition of extrinsic apoptosis induction by XIAP |
| 13 | Syn_TNFR1, Syn_TNF, Syn_TRADD, Syn_RIP1, Syn_RIP3, Syn_MLKL, T1, T2, T3, T58, T51, T52, diss4, T48, diss2, Necroptosis, T50, deg2 | Dissociation of TRADD and RIP1 and formation of the necrosome leading to necroptosis |
| **14** | deg3, Syn_TNFR1, Syn_TNF, Syn_TRADD, Syn_RIP1, Syn_FADD, 2∗Syn_Procasp8, T1, T2, T3, T64, CASP3_inhib, T31, T32, T63, T48, diss2, T53, T54, T61, T62, T50, deg2, Syn_XIAP, diss5, Syn_Procasp3 | Dissociation of TRADD and RIP1 and formation of complex IIb, inhibition of extrinsic apoptosis induction by XIAP |
| **15** | T22, T23, T24, deg7, Syn_SCF, T25, T26, T27, T30, T39, T40, T38, Syn_IkB | Activation of NF-κB, degradation of IκB and gene expression of IκB, formation of the inhibitory complex |
| **16** | T22, T37, T23, T24, deg7, Syn_SCF, T26, T27, Syn_IkB | NF-κB-dependent gene expression of IκB and formation of the inhibitory complex in the cytosol, activation of NF-κB and degradation of the inhibitor |
| **17** | Syn_TNFR1, Syn_TNF, Syn_TRADD, Syn_RIP1, Syn_FADD, Syn_Procasp8, T1, T2, T3, T33, T34, T48, diss2, T43, T44, T47, CIIa_inhib, T49, deg1, Syn_cFLIPL, diss3 | Dissociation of TRADD and RIP1 and formation of complex IIa, inhibition of apoptosis induction by cFLIP_L_ |
| 18 | Syn_NF-kB, deg4 | Turnover of NF-κB |
| **19** | Syn_TNFR1, Syn_TNF, Syn_TRADD, Syn_RIP1, Syn_FADD, Syn_Procasp8, Syn_RIP3, T1, T2, T3, T60, RIP1_RIP3_inhib, T33, T34, T48, diss2, T53, T54, T59, Pc8_inhib, T50, deg2, Syn_cFLIPL, diss5 | Dissociation of TRADD and RIP1 and formation of complex IIb, inhibition of apoptosis by cFLIP_L_ |
| 20 | Syn_TNFR1, Syn_TNF, Syn_TRADD, Syn_RIP1, Syn_TRAF2, Syn_cIAP, Syn_TAB, Syn_TAK1, 2 Syn_NEMO, 2 Syn_IKK, Syn_LUBAC, Syn_FADD, Syn_Procasp8, Syn_cFLIPs, Syn_RIP3, Syn_MLKL, T1, T2, T3, T4, T5, T14, T58, T7, T15, T8, T16, T9, T17, T13, T10, T11, T12, T18, T19, Syn_CYLD, diss1, T21, T20, T53, T54, T55, T56, T57, Necroptosis, T50, deg2, diss5, T6 | Formation of complex I and dissociation via CYLD, formation of complex IIb and necroptosis induction following cFLIP_S_ inhibition |
| 21 | 2 Syn_BAX, Syn_SMAC, Syn_Cyt c, Syn_Apaf1, 2 Syn_Procasp9, Apoptosis, T65, deg3, T66, T67, Syn_TNFR1, Syn_TNF, Syn_TRADD, Syn_RIP1, T69, Syn_TRAF2, Syn_cIAP, T71, Syn_TAB, Syn_TAK1, 2 Syn_NEMO,T72, 2 Syn_IKK,Syn_LUBAC, T73, Syn_FADD, 2 Syn_Procasp8, T1, T2, T3, T4, T5, diss6, T14, T70, T7, T15, T8, T16, T9, T17, T13, deg5, deg6, T74, T10, T11, T12, T18, T19, Syn_CYLD, diss1, T21, T20, T43, T44, T45, T46, T49, deg1, diss3, T6, Syn_Procasp3, Syn_Bid | Formation of complex I and dissociation via CYLD, formation of complex IIa and intrinsic induction of apoptosis |
| **22** | Syn_TNFR1, Syn_TNF, Syn_TRADD, Syn_RIP1, Syn_TRAF2, Syn_cIAP, Syn_TAB, Syn_TAK1, 2 Syn_NEMO, 2 Syn_IKK, Syn_LUBAC, Syn_FADD, Syn_Procasp8, Syn_RIP3, T1, T2, T3, T4, T5, T14, T60, RIP1_RIP3_inhib, T7, T15, T8, T16, T9, T17, T13, T33, T34, T10, T11, T12, T18, T19, Syn_CYLD, diss1, T21, T20, T53, T54, T59, Pc8_inhib, T50, deg2, Syn_cFLIPL, diss5, T6 | Formation of complex I and dissociation via CYLD, formation of complex IIb and inhibition of apoptosis by cFLIP_L_ |
| **23** | Syn_TNFR1, Syn_TNF, Syn_TRADD, Syn_RIP1, Syn_TRAF2, Syn_cIAP, Syn_TAB, Syn_TAK1, 2 Syn_NEMO, 2 Syn_IKK, Syn_LUBAC, Syn_FADD, Syn_Procasp8, T1, T2, T3, T4, T5, T14, T7, T15, T8, T16, T9, T17, T13, T33, T34, T10, T11, T12, T18, T19, Syn_CYLD, diss1, T21, T20, T43, T44, T47, CIIa_inhib, T49, deg1, Syn_cFLIPL, diss3, T6 | Formation of complex I and dissociation via CYLD, formation of complex IIa and inhibition of apoptosis induction by cFLIP_L_ |
| 24 | 2 Syn_BAX, Syn_SMAC, Syn_Cyt c, Syn_Apaf1, 2 Syn_Procasp9, Apoptosis, T65, deg3, T66, T67, Syn_TNFR1, Syn_TNF, Syn_TRADD, Syn_RIP1, T69, Syn_TRAF2, Syn_cIAP, T71, Syn_TAB, Syn_TAK1, 2 Syn_NEMO,T72, 2 Syn_IKK,Syn_LUBAC, T73 , Syn_FADD, 2 Syn_Procasp8, T1, T2, T3, T4, T5, diss6, T14, T70, T7, T15, T8, T16, T9, T17, T13, deg5, deg6, T74, T10, T11, T12, T18, T19, Syn_CYLD, diss1, T21, T20, T53, T54, T61, T62, T50, deg2, diss5, T6, Syn_Procasp3, Syn_Bid | Formation of complex I and dissociation via CYLD, formation of complex IIb and intrinsic induction of apoptosis |
| 25 | Syn_TNFR1, Syn_TNF, Syn_TRADD, Syn_RIP1, Syn_TRAF2, Syn_cIAP, Syn_TAB, Syn_TAK1, 2 Syn_NEMO, 2 Syn_IKK, Syn_LUBAC, Syn_RIP3, Syn_MLKL, T1, T2, T3, T4, T5, T14, T58, T51, T7, T15, T52, T8, T16, T9, T17, T13, diss4, T10, T11, T12, T18, T19, Syn_CYLD, diss1, T21, T20, Necroptosis, T50, deg2, T6 | Formation of complex I and dissociation via CYLD, formation of the necrosome and induction of necroptosis |
| **26** | 2 Syn_BAX, Syn_SMAC, Syn_Cyt_c, Syn_Apaf1, 2 Syn_Procasp9, T65, deg3, T66, T67, Syn_TNFR1, Syn_TNF, Syn_TRADD, Syn_RIP1, T69, T71, T72, T73, Syn_FADD, 2 Syn_Procasp8, T1, T2, T3, diss6, T64, CASP3_inhib, T70, deg5, T31, T32, deg6, T74, T48, diss2, T53, T54, T61, T62, T50, deg2, Syn_XIAP, diss5, Syn_Procasp3 , Syn_Bid | Dissociation of TRADD and RIP1 and formation of complex IIb, inhibition of caspase 3 by XIAP via the intrinsic apoptosis pathway |
| **27** | 2 Syn_BAX, Syn_SMAC, Syn_Cyt c, Syn_Apaf1, 2 Syn_Procasp9, T65, deg3, T66, T67, Syn_TNFR1, Syn_TNF, Syn_TRADD, T69, T71, T72, T73, Syn_FADD, 2 Syn_Procasp8, T1, T2, diss6, T64, CASP3_inhib, T70, deg5, T31, T32, deg6, T74, diss2, T42, T43, T44, T45, T46, Syn_XIAP, diss3, Syn_Procasp3, Syn_Bid | Dissociation of TRADD and formation of complex IIa, inhibition of caspase 3 by XIAP via the intrinsic apoptosis pathway |
| **28** | 2 Syn_BAX, Syn_SMAC, Syn_Cyt c, Syn_Apaf1, 2 Syn_Procasp9, T65, deg3, T66, T67, Syn_TNFR1, Syn_TNF, Syn_TRADD, Syn_RIP1, T69, T71, T72, T73, Syn_FADD, 2 Syn_Procasp8, T1, T2, T3, diss6, T64, CASP3_inhib, T70, deg5, T31, T32, deg6, T74, T48, diss2, T43, T44, T45, T46, T49, deg1, Syn_XIAP, diss3, Syn_Procasp3, Syn_Bid | Dissociation of TRADD and RIP1 and formation of complex IIa, inhibition of caspase 3 by XIAP via the intrinsic apoptosis pathway |
| **29** | 2 Syn_BAX, Syn_SMAC, Syn_Cyt c, Syn_Apaf1, 2 Syn_Procasp9, T65, deg3, T66, T67, Syn_TNFR1, Syn_TNF, Syn_TRADD, Syn_RIP1, T69, Syn_TRAF2, Syn_cIAP,T71, Syn_TAB, Syn_TAK1, 2 Syn_NEMO, T72, 2 Syn_IKK, Syn_LUBAC, T73, Syn_FADD, 2 Syn_Procasp8, T1, T2, T3, T4, T5, diss6, T14, T64, CASP3_inhib, T70, T7, T15, T8, T16, T9, T17, T13, deg5, T31, T32, deg6, T74, T10, T11, T12, T18, T19, Syn_CYLD, diss1, T21, T20, T53, T54, T61, T62, T50, deg2, Syn_XIAP, diss5, T6, Syn_Procasp3, Syn_Bid | Formation of complex I and dissociation via CYLD, formation of complex IIb and inhibition of caspase 3 by XIAP via the intrinsic apoptosis pathway |
| **30** | 2 Syn_BAX, Syn_SMAC, Syn_Cyt c, Syn_Apaf1, 2∗Syn_Procasp9, T65, deg3, T66, T67, Syn_TNFR1, Syn_TNF, Syn_TRADD, Syn_RIP1, T69, Syn_TRAF2, Syn_cIAP,T71, Syn_TAB, Syn_TAK1, 2 Syn_NEMO, T72, 2 Syn_IKK, Syn_LUBAC, T73, Syn_FADD, 2 Syn_Procasp8, T1, T2, T3, T4, T5, diss6, T14, T64, CASP3_inhib, T70, T7, T15, T8, T16, T9, T17, T13, deg5, T31, T32, deg6, T74, T10, T11, T12, T18, T19, Syn_CYLD, diss1, T21, T20, T43, T44, T45, T46, T49, deg1, Syn_XIAP, diss3, T6, Syn_Procasp3, Syn_Bid | Formation of complex I and dissociation via CYLD, formation of complex IIa and inhibition of caspase 3 by XIAP via the intrinsic apoptosis pathway |
| **31** | 2 Syn_BAX, Syn_SMAC, Syn_Cyt c, Syn_Apaf1, Syn_Procasp9, T65, deg3, T66, T67, Syn_TNFR1, Syn_TNF, Syn_TRADD, Syn_RIP1, T69, T71, T72, Syn_FADD, 2 Syn_Procasp8, T75, Pc9_inhib, T1, T2, T3, T70, deg5, T31, T32, T48, diss2, T53, T54, T61, T62, T50, deg2, Syn_XIAP, diss5, Syn_Bid | Dissociation of TRADD and RIP1 and formation of complex IIb, inhibition of procaspase 9 by XIAP in the intrinsic pathway |
| **32** | 2 Syn_BAX, Syn_SMAC, Syn_Cyt c, Syn_Apaf1, Syn_Procasp9, T65, deg3, T66, T67, Syn_TNFR1, Syn_TNF, Syn_TRADD, T69, T71, T72, Syn_FADD, 2 Syn_Procasp8 , T75, Pc9_inhib, T1, T2, T70, deg5, T31, T32, diss2, T42, T43, T44, T45, T46, Syn_XIAP, diss3, Syn_Bid | Dissociation of TRADD and formation of complex IIa, inhibition of procaspase 9 by XIAP in the intrinsic pathway |
| **33** | 2 Syn_BAX, Syn_SMAC, Syn_Cyt c, Syn_Apaf1, Syn_Procasp9, T65, deg3, T66, T67, Syn_TNFR1, Syn_TNF, Syn_TRADD, Syn_RIP1, T69, T71, T72, Syn_FADD, 2 Syn_Procasp8, T75, Pc9_inhib, T1, T2, T3, T70, deg5, T31, T32, T48, diss2, T43, T44, T45, T46, T49, deg1, Syn_XIAP, diss3, Syn_Bid | Dissociation of TRADD and RIP1 and formation of complex IIa, inhibition of procaspase 9 by XIAP in the intrinsic pathway |
| **34** | 2 Syn_BAX, Syn_SMAC, Syn_Cyt c, Syn_Apaf1, Syn_Procasp9, T65, deg3, T66, T67, Syn_TNFR1, Syn_TNF, Syn_TRADD, Syn_RIP1, T69, Syn_TRAF2, Syn_cIAP,T71, Syn_TAB, Syn_TAK1, 2 Syn_NEMO, T72, 2 Syn_IKK, Syn_LUBAC, Syn_FADD, 2 Syn_Procasp8, T75, Pc9_inhib, T1, T2, T3, T4, T5, T14, T70, T7, T15, T8, T16, T9, T17, T13, deg5, T31, T32, T10, T11, T12, T18, T19, Syn_CYLD, diss1, T21, T20, T53, T54, T61, T62, T50, deg2, Syn_XIAP, diss5, T6, Syn_Bid | Formation of complex I and dissociation via CYLD, formation of complex IIb and inhibition of procaspase 9 by XIAP in the intrinsic pathway |
| **35** | 2 Syn_BAX, Syn_SMAC, Syn_Cyt c, Syn_Apaf1, Syn_Procasp9, T65, deg3, T66, T67, Syn_TNFR1, Syn_TNF, Syn_TRADD, Syn_RIP1, T69, Syn_TRAF2, Syn_cIAP,T71, Syn_TAB, Syn_TAK1, 2 Syn_NEMO, T72, 2 Syn_IKK, Syn_LUBAC, Syn_FADD, 2 Syn_Procasp8, T75, Pc9_inhib, T1, T2, T3, T4, T5, T14, T70, T7, T15, T8, T16, T9, T17, T13, deg5, T31, T32, T10, T11, T12, T18, T19, Syn_CYLD, diss1, T21, T20, T43, T44, T45, T46, T49, deg1, Syn_XIAP, diss3, T6, Syn_Bid | Formation of complex I and dissociation via CYLD, formation of complex IIa and inhibition of procaspase 9 by XIAP in the intrinsic pathway |
| **36** | 2 Syn_BAX, Syn_SMAC, Syn_Cyt c, Syn_Apaf1, Syn_Procasp9, T65, deg3, T66, T67, Syn_TNFR1, Syn_TNF, Syn_TRADD, Syn_RIP1, T69, T71, T72, Syn_FADD, T76, 2 Syn_Procasp8, Apo_XIAP_inhib, T75, T1, T2, T3, T70, T31, T32, T48, diss2, T53, T54, T61, T62, T50, deg2, Syn_XIAP, diss5, Syn_Bid | Dissociation of TRADD and RIP1 and formation of complex IIb, inhibition of XIAP by SMAC and intrinsic apoptosis induction |
| **37** | 2 Syn_BAX, Syn_SMAC, Syn_Cyt c, Syn_Apaf1, Syn_Procasp9, T65, deg3, T66, T67, Syn_TNFR1, Syn_TNF, Syn_TRADD, T69, T71, T72, Syn_FADD, T76, 2 Syn_Procasp8, Apo_XIAP_inhib, T75, T1, T2, T70, T31, T32, diss2, T42, T43, T44, T45, T46, Syn_XIAP, diss3, Syn_Bid | Dissociation of TRADD and formation of complex IIa, inhibition of XIAP by SMAC and intrinsic apoptosis induction |
| **38** | 2 Syn_BAX, Syn_SMAC, Syn_Cyt c, Syn_Apaf1, Syn_Procasp9, T65, deg3, T66, T67, Syn_TNFR1, Syn_TNF, Syn_TRADD, Syn_RIP1, T69, T71, T72, Syn_FADD, T76, 2 Syn_Procasp8, Apo_XIAP_inhib, T75, T1, T2, T3, T70, T31, T32, T48, diss2, T43, T44, T45, T46, T49, deg1, Syn_XIAP, diss3, Syn_Bid | Dissociation of TRADD and RIP1 and formation of complex IIa, inhibition of XIAP by SMAC and intrinsic apoptosis induction |
| **39** | 2 Syn_BAX, Syn_SMAC, Syn_Cyt c, Syn_Apaf1, Syn_Procasp9, T65, deg3, T66, T67, Syn_TNFR1, Syn_TNF, Syn_TRADD, Syn_RIP1, T69, Syn_TRAF2, Syn_cIAP,T71, Syn_TAB, Syn_TAK1, 2 Syn_NEMO, T72, 2 Syn_IKK, Syn_LUBAC, Syn_FADD, T76, 2 Syn_Procasp8, Apo_XIAP_inhib, T75, T1, T2, T3, T4, T5, T14, T70, T7, T15, T8, T16, T9, T17, T13, T31, T32, T10, T11, T12, T18, T19, Syn_CYLD, diss1, T21, T20, T53, T54, T61, T62, T50, deg2, Syn_XIAP, diss5, T6, Syn_Bid | Formation of complex I and dissociation via CYLD, formation of complex IIb and inhibition of XIAP by SMAC and intrinsic apoptosis induction |
| **40** | 2 Syn_BAX, Syn_SMAC, Syn_Cyt c, Syn_Apaf1, Syn_Procasp9, T65, deg3, T66, T67, Syn_TNFR1, Syn_TNF, Syn_TRADD, Syn_RIP1, T69, Syn_TRAF2, Syn_cIAP,T71, Syn_TAB, Syn_TAK1, 2 Syn_NEMO, T72, 2 Syn_IKK, Syn_LUBAC, Syn_FADD, T76, 2∗Syn_Procasp8, Apo_XIAP_inhib, T75, T1, T2, T3, T4, T5, T14, T70, T7, T15, T8, T16, T9, T17, T13, T31, T32, T10, T11, T12, T18, T19, Syn_CYLD, diss1, T21, T20, T43, T44, T45, T46, T49, deg1, Syn_XIAP, diss3, T6, Syn_Bid | Formation of complex I and dissociation via CYLD, formation of complex IIa and inhibition of XIAP by SMAC and intrinsic apoptosis induction |
| **41** | Syn_BAX, T65, deg3, T66, T67, T68, Syn_TNFR1, Syn_TNF, BAX_inhib, Syn_TRADD, Syn_RIP1, Syn_FADD, 2 Syn_Procasp8, T1, T2, T3, T35, T36, T48, diss2, T53, T54, T61, T62, T50, deg2, Syn_BCL-2, diss5, Syn_Bid | Dissociation of TRADD and RIP1 and formation of complex IIb, inhibition of BAX by BCL-2 |
| **42** | Syn_BAX, T65, deg3, T66, T67, T68, Syn_TNFR1, Syn_TNF, BAX_inhib, Syn_TRADD, Syn_FADD, 2 Syn_Procasp8, T1, T2, T35, T36, diss2, T42, T43, T44, T45, T46, Syn_BCL-2, diss3, Syn_Bid | Dissociation of TRADD and formation of complex IIa, inhibition of BAX by BCL-2 |
| **43** | Syn_BAX, T65, deg3, T66, T67, T68, Syn_TNFR1, Syn_TNF, BAX_inhib, Syn_TRADD, Syn_RIP1, Syn_FADD, 2 Syn_Procasp8, T1, T2, T3, T35, T36, T48, diss2, T43, T44, T45, T46, T49, deg1, Syn_BCL-2, diss3, Syn_Bid | Dissociation of TRADD and RIP1, formation of complex IIa, inhibition of BAX by BCL-2 |
| **44** | Syn_BAX, T65, deg3, T66, T67, T68, Syn_TNFR1, Syn_TNF, BAX_inhib, Syn_TRADD, Syn_RIP1, Syn_TRAF2, Syn_cIAP, Syn_TAB, Syn_TAK1, 2 Syn_NEMO, 2 Syn_IKK, Syn_LUBAC, Syn_FADD, 2 Syn_Procasp8, T1, T2, T3, T4, T5, T14, T7, T15, T8, T16, T9, T17, T13, T35, T36, T10, T11, T12, T18, T19, Syn_CYLD, diss1, T21, T20, T53, T54, T61, T62, T50, deg2, Syn_BCL-2, diss5, T6, Syn_Bid | Formation of complex I and dissociation via CYLD, formation of complex IIb and inhibition of BAX by BCL-2 |
| **45** | Syn_BAX, T65, deg3, T66, T67, T68, Syn_TNFR1, Syn_TNF, BAX_inhib, Syn_TRADD, Syn_RIP1, Syn_TRAF2, Syn_cIAP, Syn_TAB, Syn_TAK1, 2 Syn_NEMO, 2 Syn_IKK, Syn_LUBAC, Syn_FADD, 2 Syn_Procasp8, T1, T2, T3, T4, T5, T14, T7, T15, T8, T16, T9, T17, T13, T35, T36, T10, T11, T12, T18, T19, Syn_CYLD, diss1, T21, T20, T43, T44, T45, T46, T49, deg1, Syn_BCL-2, diss3, T6, Syn_Bid | Formation of complex I and dissociation via CYLD, formation of complex IIa and inhibition of BAX by BCL-2 |
| 46 | 2 Syn_BAX, Syn_SMAC, Syn_Cyt c, Syn_Apaf1, 2 Syn_Procasp9, Apoptosis, T65, deg3, T66, T67, Syn_TNFR1, Syn_TNF, Syn_TRADD, Syn_RIP1, T69, T71, T72, T73, Syn_FADD, 2 Syn_Procasp8, T1, T2, T3, diss6, T70, deg5, deg6, T74, T48, diss2, T53, T54, T61, T62, T50, deg2, diss5, Syn_Procasp3, Syn_Bid | Dissociation of TRADD and RIP1 and induction of intrinsic apoptosis via complex IIb |
| 47 | 2 Syn_BAX, Syn_SMAC, Syn_Cyt c, Syn_Apaf1, 2 Syn_Procasp9, Apoptosis, T65, deg3, T66, T67, Syn_TNFR1, Syn_TNF, Syn_TRADD, T69, T71, T72, T73, Syn_FADD, 2 Syn_Procasp8, T1, T2, diss6, T70, deg5, deg6, T74, diss2, T42, T43, T44, T45, T46, diss3, Syn_Procasp3, Syn_Bid | Dissociation of TRADD and induction of intrinsic apoptosis via complex IIb |
| 48 | 2 Syn_BAX, Syn_SMAC, Syn_Cyt c, Syn_Apaf1, 2 Syn_Procasp9, Apoptosis, T65, deg3, T66, T67, Syn_TNFR1, Syn_TNF, Syn_TRADD, Syn_RIP1, T69, T71, T72, T73, Syn_FADD, 2 Syn_Procasp8, T1, T2, T3, diss6, T70, deg5, deg6, T74, T48, diss2, T43, T44, T45, T46, T49, deg1, diss3, Syn_Procasp3, Syn_Bid | Dissociation of TRADD and RIP1 and induction of intrinsic apoptosis via complex IIa |
