# Supplementary material for: Mathematical modeling of the molecular switch of TNFR1-mediated signaling pathways applying Petri net formalism and in silico knockout analysis
Source: PLoS Comput Biol. 2022 Aug 22;18(8):e1010383. doi: 10.1371/journal.pcbi.1010383 (PMC9467317; doi:10.1371/journal.pcbi.1010383)
Supplement: S6 Table — For each MI, its number, the number(s) of the transition invariants (TIs), and the names of transitions are given. For descriptions of transitions, we refer to S1 Table. For descriptions of TIs, see S5 Table. An MI combines several TIs to cover a complete pathway. We indicate whether an MI is pure. A pure MI induces a network that is free of place invariants, see text. (DOCX) [file pcbi.1010383.s007.docx]

**S6 Table:** List of 279 Manatee invariants (MIs). For each MI, its number, the number(s) of the transition invariants (TIs), and the names of transitions are given. For descriptions of transitions, we refer to S1 Table. For descriptions of TIs, see S5 Table. An MI combines several TIs to cover a complete pathway. We indicate whether an MI is pure. A pure MI induces a network that is free of place invariants, see text.

| **Manatee invariants** | **Covered**  **transition**  **invariants** | **Covered**  **transitions** |
| --- | --- | --- |
| 1. (pure) | 1 | Apoptosis, deg3, Syn_TNFR1, Syn_TNF, Syn_TRADD, Syn_RIP1, Syn_TRAF2, Syn_cIAP, Syn_TAB, Syn_TAK1, 2 Syn_NEMO, 2 Syn_IKK, Syn_LUBAC, Syn_FADD, 2 Syn_Procasp8, T1, T2, T3, T4, T5, T14, T7, T15, T8, T16, T9, T17, T13, T63, T10, T11, T12, T18, T19, Syn_CYLD, diss1, T21, T20, T43, T44, T45, T46, T49, deg1, diss3, T6, Syn_Procasp3 |
| 2. (pure) | 2 | Apoptosis, deg3, Syn_TNFR1, Syn_TNF, Syn_TRADD, Syn_RIP1, Syn_TRAF2, Syn_cIAP, Syn_TAB, Syn_TAK1, 2 Syn_NEMO, 2 Syn_IKK, Syn_LUBAC, Syn_FADD, 2 Syn_Procasp8, T1, T2, T3, T4, T5, T14, T7, T15, T8, T16, T9, T17, T13, T63, T10, T11, T12, T18, T19, Syn_CYLD, diss1, T21, T20, T53, T54, T61, T62, T50, deg2, diss5, T6, Syn_Procasp3 |
| 3. (pure) | 3 | Apoptosis, deg3, Syn_TNFR1, Syn_TNF, Syn_TRADD, Syn_RIP1, Syn_FADD, 2 Syn_Procasp8, T1, T2, T3, T63, T48, diss2, T43, T44, T45, T46, T49, deg1, diss3, Syn_Procasp3 |
| 4. | 15, 18, 19, 21 | 2 Syn_BAX, Syn_SMAC, Syn_Cyt_c, Syn_Apaf1, 2 Syn_Procasp9, Apoptosis, T65, deg3, T66, T67, 2 Syn_TNFR1, 2 Syn_TNF, 2 Syn_TRADD, 2 Syn_RIP1, T69, Syn_TRAF2, Syn_cIAP, T71, Syn_TAB, Syn_TAK1, 2 Syn_NEMO, T72, 2 Syn_IKK, Syn_LUBAC, T73, Syn_NF_kB, 2 Syn_FADD, 3 Syn_Procasp8, Syn_RIP3, 2 T1, 2 T2, 2 T3, T4, T5, diss6, T14, T60, RIP1_RIP3_inhib, T70, T7, T15, T8, T16, T9, T17, T13, deg5, T22, T23, T24, deg7, Syn_SCF, T25, T26, T27, T30, T33, T34, deg6, T74, T39, T40, T10, T11, T12, T18, T19, Syn_CYLD, diss1, T21, T20, deg4, T38, T48, diss2, T53, T43, T54, T59, Pc8_inhib, T44, T45, T46, T50, deg2, T49, deg1, Syn_IkB, Syn_cFLIPL, diss3, diss5, T6, Syn_Procasp3, Syn_Bid |
| 5. (pure) | 5 | Apoptosis, deg3, Syn_TNFR1, Syn_TNF, Syn_TRADD, Syn_FADD, 2 Syn_Procasp8, T1, T2, T63, diss2, T42, T43, T44, T45, T46, diss3, Syn_Procasp3 |
| 6. (pure) | 6 | Apoptosis, deg3, Syn_TNFR1, Syn_TNF, Syn_TRADD, Syn_RIP1, Syn_FADD, 2 Syn_Procasp8, T1, T2, T3, T63, T48, diss2, T53, T54, T61, T62, T50, deg2, diss5, Syn_Procasp3 |
| 7. | 9, 15, 18 | Syn_TNFR1, Syn_TNF, Syn_TRADD, Syn_RIP1, Syn_TRAF2, Syn_cIAP, Syn_TAB, Syn_TAK1, 2 Syn_NEMO, 2 Syn_IKK, Syn_LUBAC, Syn_NF_kB, T1, T2, T3, T4, T5, T14, T7, T15, T8, T16, T9, T17, T13, T22, T23, T24, deg7, Syn_SCF, T25, T26, T28, T29_, T27, T30, T39, T40, T41, CI_diss, T10, T11, T12, deg4, T38, Syn_IkB, Syn_A20, T6 |
| 8. | 15, 18, 19, 20 | 2 Syn_TNFR1, 2 Syn_TNF, 2 Syn_TRADD, 2 Syn_RIP1, Syn_TRAF2, Syn_cIAP, Syn_TAB, Syn_TAK1, 2 Syn_NEMO, 2 Syn_IKK, Syn_LUBAC, Syn_NF_kB, 2 Syn_FADD, 2 Syn_Procasp8, Syn_cFLIPs, 2 Syn_RIP3, Syn_MLKL, 2 T1, 2 T2, 2 T3, T4, T5, T14, T60, RIP1_RIP3_inhib, T58, T7, T15, T8, T16, T9, T17, T13, T22, T23, T24, deg7, Syn_SCF, T25, T26, T27, T30, T33, T34, T39, T40, T10, T11, T12, T18, T19, Syn_CYLD, diss1, T21, T20, deg4, T38, T48, diss2, 2 T53, 2 T54, T59, Pc8_inhib, T55, T56, T57, Necroptosis, 2 T50, 2 deg2, Syn_IkB, Syn_cFLIPL, 2 diss5, T6 |
| 9. | 15, 18, 19, 22 | 2 Syn_TNFR1, 2 Syn_TNF, 2 Syn_TRADD, 2 Syn_RIP1, Syn_TRAF2, Syn_cIAP, Syn_TAB, Syn_TAK1, 2 Syn_NEMO, 2 Syn_IKK, Syn_LUBAC, Syn_NF_kB, 2 Syn_FADD, 2 Syn_Procasp8, 2 Syn_RIP3, 2 T1, 2 T2, 2 T3, T4, T5, T14, 2 T60, 2 RIP1_RIP3_inhib, T7, T15, T8, T16, T9, T17, T13, T22, T23, T24, deg7, Syn_SCF, T25, T26, T27, T30, 2 T33, 2 T34, T39, T40, T10, T11, T12, T18, T19, Syn_CYLD, diss1, T21, T20, deg4, T38, T48, diss2, 2 T53, 2 T54, 2 T59, 2 Pc8_inhib, 2 T50, 2 deg2, Syn_IkB, 2 Syn_cFLIPL, 2 diss5, T6 |
| 10. (pure) | 10 | Syn_TNFR1, Syn_TNF, Syn_TRADD, Syn_RIP1, Syn_FADD, Syn_Procasp8, Syn_cFLIPs, Syn_RIP3, Syn_MLKL, T1, T2, T3, T58, T48, diss2, T53, T54, T55, T56, T57, Necroptosis, T50, deg2, diss5 |
| 11. | 15, 18, 19, 23 | 2 Syn_TNFR1, 2 Syn_TNF, 2 Syn_TRADD, 2 Syn_RIP1, Syn_TRAF2, Syn_cIAP, Syn_TAB, Syn_TAK1, 2 Syn_NEMO, 2 Syn_IKK, Syn_LUBAC, Syn_NF_kB, 2 Syn_FADD, 2 Syn_Procasp8, Syn_RIP3, 2 T1, 2 T2, 2 T3, T4, T5, T14, T60, RIP1_RIP3_inhib, T7, T15, T8, T16, T9, T17, T13, T22, T23, T24, deg7, Syn_SCF, T25, T26, T27, T30, 2 T33, 2 T34, T39, T40, T10, T11, T12, T18, T19, Syn_CYLD, diss1, T21, T20, deg4, T38, T48, diss2, T53, T43, T54, T59, Pc8_inhib, T44, T47, CIIa_inhib, T50, deg2, T49, deg1, Syn_IkB, 2 Syn_cFLIPL, diss3, diss5, T6 |
| 12. | 8, 15, 18 | deg3, Syn_TNFR1, Syn_TNF, Syn_TRADD, Syn_RIP1, Syn_TRAF2, Syn_cIAP, Syn_TAB, Syn_TAK1, 2 Syn_NEMO, 2 Syn_IKK, Syn_LUBAC, Syn_NF_kB, Syn_FADD, 2 Syn_Procasp8, T1, T2, T3, T4, T5, T14, T64, CASP3_inhib, T7, T15, T8, T16, T9, T17, T13, T22, T23, T24, deg7, Syn_SCF, T25, T26, T27, T30, T31, T32, T63, T39, T40, T10, T11, T12, T18, T19, Syn_CYLD, diss1, T21, T20, deg4, T38, T53, T54, T61, T62, T50, deg2, Syn_IkB, Syn_XIAP, diss5, T6, Syn_Procasp3 |
| 13. (pure) | 13 | Syn_TNFR1, Syn_TNF, Syn_TRADD, Syn_RIP1, Syn_RIP3, Syn_MLKL, T1, T2, T3, T58, T51, T52, diss4, T48, diss2, Necroptosis, T50, deg2 |
| 14. | 15, 18, 19, 24 | 2 Syn_BAX, Syn_SMAC, Syn_Cyt_c, Syn_Apaf1, 2 Syn_Procasp9, Apoptosis, T65, deg3, T66, T67, 2 Syn_TNFR1, 2 Syn_TNF, 2 Syn_TRADD, 2 Syn_RIP1, T69, Syn_TRAF2, Syn_cIAP, T71, Syn_TAB, Syn_TAK1, 2 Syn_NEMO, T72, 2 Syn_IKK, Syn_LUBAC, T73, Syn_NF_kB, 2 Syn_FADD, 3 Syn_Procasp8, Syn_RIP3, 2 T1, 2 T2, 2 T3, T4, T5, diss6, T14, T60, RIP1_RIP3_inhib, T70, T7, T15, T8, T16, T9, T17, T13, deg5, T22, T23, T24, deg7, Syn_SCF, T25, T26, T27, T30, T33, T34, deg6, T74, T39, T40, T10, T11, T12, T18, T19, Syn_CYLD, diss1, T21, T20, deg4, T38, T48, diss2, 2 T53, 2 T54, T61, T62, T59, Pc8_inhib, 2 T50, 2 deg2, Syn_IkB, Syn_cFLIPL, 2 diss5, T6, Syn_Procasp3, Syn_Bid |
| 15. | 15, 18, 19, 26 | 2 Syn_TNFR1, 2 Syn_TNF, 2 Syn_TRADD, 2 Syn_RIP1, Syn_TRAF2, Syn_cIAP, Syn_TAB, Syn_TAK1, 2 Syn_NEMO, 2 Syn_IKK, Syn_LUBAC, Syn_NF_kB, Syn_FADD, Syn_Procasp8, 2 Syn_RIP3, Syn_MLKL, 2 T1, 2 T2, 2 T3, T4, T5, T14, T60, RIP1_RIP3_inhib, T58, T51, T7, T15, T52, T8, T16, T9, T17, T13, T22, T23, T24, deg7, Syn_SCF, T25, T26, T27, T30, T33, T34, diss4, T39, T40, T10, T11, T12, T18, T19, Syn_CYLD, diss1, T21, T20, deg4, T38, T48, diss2, T53, T54, T59, Pc8_inhib, Necroptosis, 2 T50, 2 deg2, Syn_IkB, Syn_cFLIPL, diss5, T6 |
| 16. | 15, 18, 19, 30 | 2 Syn_BAX, Syn_SMAC, Syn_Cyt_c, Syn_Apaf1, 2 Syn_Procasp9, T65, deg3, T66, T67, 2 Syn_TNFR1, 2 Syn_TNF, 2 Syn_TRADD, 2 Syn_RIP1, T69, Syn_TRAF2, Syn_cIAP, T71, Syn_TAB, Syn_TAK1, 2 Syn_NEMO, T72, 2 Syn_IKK, Syn_LUBAC, T73, Syn_NF_kB, 2 Syn_FADD, 3 Syn_Procasp8, Syn_RIP3, 2 T1, 2 T2, 2 T3, T4, T5, diss6, T14, T60, RIP1_RIP3_inhib, T64, CASP3_inhib, T70, T7, T15, T8, T16, T9, T17, T13, deg5, T22, T23, T24, deg7, Syn_SCF, T25, T26, T27, T30, T31, T33, T32, T34, deg6, T74, T39, T40, T10, T11, T12, T18, T19, Syn_CYLD, diss1, T21, T20, deg4, T38, T48, diss2, 2 T53, 2 T54, T61, T62, T59, Pc8_inhib, 2 T50, 2 deg2, Syn_IkB, Syn_XIAP, Syn_cFLIPL, 2 diss5, T6, Syn_Procasp3, Syn_Bid |
| 17. | 15, 18, 19, 31 | 2 Syn_BAX, Syn_SMAC, Syn_Cyt_c, Syn_Apaf1, 2 Syn_Procasp9, T65, deg3, T66, T67, 2 Syn_TNFR1, 2 Syn_TNF, 2 Syn_TRADD, 2 Syn_RIP1, T69, Syn_TRAF2, Syn_cIAP, T71, Syn_TAB, Syn_TAK1, 2 Syn_NEMO, T72, 2 Syn_IKK, Syn_LUBAC, T73, Syn_NF_kB, 2 Syn_FADD, 3 Syn_Procasp8, Syn_RIP3, 2 T1, 2 T2, 2 T3, T4, T5, diss6, T14, T60, RIP1_RIP3_inhib, T64, CASP3_inhib, T70, T7, T15, T8, T16, T9, T17, T13, deg5, T22, T23, T24, deg7, Syn_SCF, T25, T26, T27, T30, T31, T33, T32, T34, deg6, T74, T39, T40, T10, T11, T12, T18, T19, Syn_CYLD, diss1, T21, T20, deg4, T38, T48, diss2, T53, T43, T54, T59, Pc8_inhib, T44, T45, T46, T50, deg2, T49, deg1, Syn_IkB, Syn_XIAP, Syn_cFLIPL, diss3, diss5, T6, Syn_Procasp3, Syn_Bid |
| 18. (pure) | 18 | Syn_NF_kB, deg4 |
| 19. | 13, 16, 19, 34 | 2 Syn_BAX, Syn_SMAC, Syn_Cyt_c, Syn_Apaf1, Syn_Procasp9, T65, deg3, T66, T67, 2 Syn_TNFR1, 2 Syn_TNF, 2 Syn_TRADD, 2 Syn_RIP1, T69, Syn_TRAF2, Syn_cIAP, T71, Syn_TAB, Syn_TAK1, 2 Syn_NEMO, T72, 2 Syn_IKK, Syn_LUBAC, Syn_NF_kB, 2 Syn_FADD, 3 Syn_Procasp8, Syn_RIP3, T75, Pc9_inhib, 2 T1, 2 T2, 2 T3, T4, T5, T14, T60, RIP1_RIP3_inhib, T70, T7, T15, T8, T16, T9, T17, T13, deg5, T22, T23, T24, deg7, Syn_SCF, T25, T26, T27, T30, T31, T33, T32, T34, T39, T40, T10, T11, T12, T18, T19, Syn_CYLD, diss1, T21, T20, deg4, T38, T48, diss2, 2 T53, 2 T54, T61, T62, T59, Pc8_inhib, 2 T50, 2 deg2, Syn_IkB, Syn_XIAP, Syn_cFLIPL, 2 diss5, T6, Syn_Bid |
| 20. (pure) | 20 | Syn_TNFR1, Syn_TNF, Syn_TRADD, Syn_RIP1, Syn_TRAF2, Syn_cIAP, Syn_TAB, Syn_TAK1, 2 Syn_NEMO, 2 Syn_IKK, Syn_LUBAC, Syn_FADD, Syn_Procasp8, Syn_cFLIPs, Syn_RIP3, Syn_MLKL, T1, T2, T3, T4, T5, T14, T58, T7, T15, T8, T16, T9, T17, T13, T10, T11, T12, T18, T19, Syn_CYLD, diss1, T21, T20, T53, T54, T55, T56, T57, Necroptosis, T50, deg2, diss5, T6 |
| 21. (pure) | 21 | 2 Syn_BAX, Syn_SMAC, Syn_Cyt_c, Syn_Apaf1, 2 Syn_Procasp9, Apoptosis, T65, deg3, T66, T67, Syn_TNFR1, Syn_TNF, Syn_TRADD, Syn_RIP1, T69, Syn_TRAF2, Syn_cIAP, T71, Syn_TAB, Syn_TAK1, 2 Syn_NEMO, T72, 2 Syn_IKK, Syn_LUBAC, T73, Syn_FADD, 2 Syn_Procasp8, T1, T2, T3, T4, T5, diss6, T14, T70, T7, T15, T8, T16, T9, T17, T13, deg5, deg6, T74, T10, T11, T12, T18, T19, Syn_CYLD, diss1, T21, T20, T43, T44, T45, T46, T49, deg1, diss3, T6, Syn_Procasp3, Syn_Bid |
| 22. | 15, 18, 19, 35 | 2 Syn_BAX, Syn_SMAC, Syn_Cyt_c, Syn_Apaf1, Syn_Procasp9, T65, deg3, T66, T67, 2 Syn_TNFR1, 2 Syn_TNF, 2 Syn_TRADD, 2 Syn_RIP1, T69, Syn_TRAF2, Syn_cIAP, T71, Syn_TAB, Syn_TAK1, 2 Syn_NEMO, T72, 2 Syn_IKK, Syn_LUBAC, Syn_NF_kB, 2 Syn_FADD, 3 Syn_Procasp8, Syn_RIP3, T75, Pc9_inhib, 2 T1, 2 T2, 2 T3, T4, T5, T14, T60, RIP1_RIP3_inhib, T70, T7, T15, T8, T16, T9, T17, T13, deg5, T22, T23, T24, deg7, Syn_SCF, T25, T26, T27, T30, T31, T33, T32, T34, T39, T40, T10, T11, T12, T18, T19, Syn_CYLD, diss1, T21, T20, deg4, T38, T48, diss2, T53, T43, T54, T59, Pc8_inhib, T44, T45, T46, T50, deg2, T49, deg1, Syn_IkB, Syn_XIAP, Syn_cFLIPL, diss3, diss5, T6, Syn_Bid |
| 23. | 14, 15, 18, 20 | deg3, 2 Syn_TNFR1, 2 Syn_TNF, 2 Syn_TRADD, 2 Syn_RIP1, Syn_TRAF2, Syn_cIAP, Syn_TAB, Syn_TAK1, 2 Syn_NEMO, 2 Syn_IKK, Syn_LUBAC, Syn_NF_kB, 2 Syn_FADD, 3 Syn_Procasp8, Syn_cFLIPs, Syn_RIP3, Syn_MLKL, 2 T1, 2 T2, 2 T3, T4, T5, T14, T58, T64, CASP3_inhib, T7, T15, T8, T16, T9, T17, T13, T22, T23, T24, deg7, Syn_SCF, T25, T26, T27, T30, T31, T32, T63, T39, T40, T10, T11, T12, T18, T19, Syn_CYLD, diss1, T21, T20, deg4, T38, T48, diss2, 2 T53, 2 T54, T61, T62, T55, T56, T57, Necroptosis, 2 T50, 2 deg2, Syn_IkB, Syn_XIAP, 2 diss5, T6, Syn_Procasp3 |
| 24. (pure) | 24 | 2 Syn_BAX, Syn_SMAC, Syn_Cyt_c, Syn_Apaf1, 2 Syn_Procasp9, Apoptosis, T65, deg3, T66, T67, Syn_TNFR1, Syn_TNF, Syn_TRADD, Syn_RIP1, T69, Syn_TRAF2, Syn_cIAP, T71, Syn_TAB, Syn_TAK1, 2 Syn_NEMO, T72, 2 Syn_IKK, Syn_LUBAC, T73, Syn_FADD, 2 Syn_Procasp8, T1, T2, T3, T4, T5, diss6, T14, T70, T7, T15, T8, T16, T9, T17, T13, deg5, deg6, T74, T10, T11, T12, T18, T19, Syn_CYLD, diss1, T21, T20, T53, T54, T61, T62, T50, deg2, diss5, T6, Syn_Procasp3, Syn_Bid |
| 25. (pure) | 25 | Syn_TNFR1, Syn_TNF, Syn_TRADD, Syn_RIP1, Syn_TRAF2, Syn_cIAP, Syn_TAB, Syn_TAK1, 2 Syn_NEMO, 2 Syn_IKK, Syn_LUBAC, Syn_RIP3, Syn_MLKL, T1, T2, T3, T4, T5, T14, T58, T51, T7, T15, T52, T8, T16, T9, T17, T13, diss4, T10, T11, T12, T18, T19, Syn_CYLD, diss1, T21, T20, Necroptosis, T50, deg2, T6 |
| 26. | 15, 18, 26, 45 | 3 Syn_BAX, Syn_SMAC, Syn_Cyt_c, Syn_Apaf1, 2 Syn_Procasp9, 2 T65, 2 deg3, 2 T66, 2 T67, T68, 2 Syn_TNFR1, 2 Syn_TNF, BAX_inhib, 2 Syn_TRADD, 2 Syn_RIP1, T69, Syn_TRAF2, Syn_cIAP, T71, Syn_TAB, Syn_TAK1, 2 Syn_NEMO, T72, 2 Syn_IKK, Syn_LUBAC, T73, Syn_NF_kB, 2 Syn_FADD, 4 Syn_Procasp8, 2 T1, 2 T2, 2 T3, T4, T5, diss6, T14, T64, CASP3_inhib, T70, T7, T15, T8, T16, T9, T17, T13, deg5, T22, T23, T24, deg7, Syn_SCF, T25, T26, T27, T30, T31, T35, T32, T36, deg6, T74, T39, T40, T10, T11, T12, T18, T19, Syn_CYLD, diss1, T21, T20, deg4, T38, T48, diss2, T53, T43, T54, T61, T62, T44, T45, T46, T50, deg2, T49, deg1, Syn_IkB, Syn_XIAP, Syn_BCL_2, diss3, diss5, T6, Syn_Procasp3, 2 Syn_Bid |
| 27. | 15, 18, 26, 44 | 3 Syn_BAX, Syn_SMAC, Syn_Cyt_c, Syn_Apaf1, 2 Syn_Procasp9, 2 T65, 2 deg3, 2 T66, 2 T67, T68, 2 Syn_TNFR1, 2 Syn_TNF, BAX_inhib, 2 Syn_TRADD, 2 Syn_RIP1, T69, Syn_TRAF2, Syn_cIAP, T71, Syn_TAB, Syn_TAK1, 2 Syn_NEMO, T72, 2 Syn_IKK, Syn_LUBAC, T73, Syn_NF_kB, 2 Syn_FADD, 4 Syn_Procasp8, 2 T1, 2 T2, 2 T3, T4, T5, diss6, T14, T64, CASP3_inhib, T70, T7, T15, T8, T16, T9, T17, T13, deg5, T22, T23, T24, deg7, Syn_SCF, T25, T26, T27, T30, T31, T35, T32, T36, deg6, T74, T39, T40, T10, T11, T12, T18, T19, Syn_CYLD, diss1, T21, T20, deg4, T38, T48, diss2, 2 T53, 2 T54, 2 T61, 2 T62, 2 T50, 2 deg2, Syn_IkB, Syn_XIAP, Syn_BCL_2, 2 diss5, T6, Syn_Procasp3, 2 Syn_Bid |
| 28. | 15, 18, 26, 40 | 4 Syn_BAX, 2 Syn_SMAC, 2 Syn_Cyt_c, 2 Syn_Apaf1, 3 Syn_Procasp9, 2 T65, 2 deg3, 2 T66, 2 T67, 2 Syn_TNFR1, 2 Syn_TNF, 2 Syn_TRADD, 2 Syn_RIP1, 2 T69, Syn_TRAF2, Syn_cIAP, 2 T71, Syn_TAB, Syn_TAK1, 2 Syn_NEMO, 2 T72, 2 Syn_IKK, Syn_LUBAC, T73, Syn_NF_kB, 2 Syn_FADD, T76, 4 Syn_Procasp8, Apo_XIAP_inhib, T75, 2 T1, 2 T2, 2 T3, T4, T5, diss6, T14, T64, CASP3_inhib, 2 T70, T7, T15, T8, T16, T9, T17, T13, deg5, T22, T23, T24, deg7, Syn_SCF, T25, T26, T27, T30, 2 T31, 2 T32, deg6, T74, T39, T40, T10, T11, T12, T18, T19, Syn_CYLD, diss1, T21, T20, deg4, T38, T48, diss2, T53, T43, T54, T61, T62, T44, T45, T46, T50, deg2, T49, deg1, Syn_IkB, 2 Syn_XIAP, diss3, diss5, T6, Syn_Procasp3, 2 Syn_Bid |
| 29. | 15, 18, 26, 39 | 4 Syn_BAX, 2 Syn_SMAC, 2 Syn_Cyt_c, 2 Syn_Apaf1, 3 Syn_Procasp9, 2 T65, 2 deg3, 2 T66, 2 T67, 2 Syn_TNFR1, 2 Syn_TNF, 2 Syn_TRADD, 2 Syn_RIP1, 2 T69, Syn_TRAF2, Syn_cIAP, 2 T71, Syn_TAB, Syn_TAK1, 2 Syn_NEMO, 2 T72, 2 Syn_IKK, Syn_LUBAC, T73, Syn_NF_kB, 2 Syn_FADD, T76, 4 Syn_Procasp8, Apo_XIAP_inhib, T75, 2 T1, 2 T2, 2 T3, T4, T5, diss6, T14, T64, CASP3_inhib, 2 T70, T7, T15, T8, T16, T9, T17, T13, deg5, T22, T23, T24, deg7, Syn_SCF, T25, T26, T27, T30, 2 T31, 2 T32, deg6, T74, T39, T40, T10, T11, T12, T18, T19, Syn_CYLD, diss1, T21, T20, deg4, T38, T48, diss2, 2 T53, 2 T54, 2 T61, 2 T62, 2 T50, 2 deg2, Syn_IkB, 2 Syn_XIAP, 2 diss5, T6, Syn_Procasp3, 2 Syn_Bid |
| 30. | 15, 18, 27, 29 | 4 Syn_BAX, 2 Syn_SMAC, 2 Syn_Cyt_c, 2 Syn_Apaf1, 4 Syn_Procasp9, 2 T65, 2 deg3, 2 T66, 2 T67, 2 Syn_TNFR1, 2 Syn_TNF, 2 Syn_TRADD, Syn_RIP1, 2 T69, Syn_TRAF2, Syn_cIAP, 2 T71, Syn_TAB, Syn_TAK1, 2 Syn_NEMO, 2 T72, 2 Syn_IKK, Syn_LUBAC, 2 T73, Syn_NF_kB, 2 Syn_FADD, 4 Syn_Procasp8, 2 T1, 2 T2, T3, T4, T5, 2 diss6, T14, 2 T64, 2 CASP3_inhib, 2 T70, T7, T15, T8, T16, T9, T17, T13, 2 deg5, T22, T23, T24, deg7, Syn_SCF, T25, T26, T27, T30, 2 T31, 2 T32, 2 deg6, 2 T74, T39, T40, T10, T11, T12, T18, T19, Syn_CYLD, diss1, T21, T20, deg4, T38, diss2, T53, T42, T43, T54, T61, T62, T44, T45, T46, T50, deg2, Syn_IkB, 2 Syn_XIAP, diss3, diss5, T6, 2 Syn_Procasp3, 2 Syn_Bid |
| 31. | 15, 18, 27, 30 | 4 Syn_BAX, 2 Syn_SMAC, 2 Syn_Cyt_c, 2 Syn_Apaf1, 4 Syn_Procasp9, 2 T65, 2 deg3, 2 T66, 2 T67, 2 Syn_TNFR1, 2 Syn_TNF, 2 Syn_TRADD, Syn_RIP1, 2 T69, Syn_TRAF2, Syn_cIAP, 2 T71, Syn_TAB, Syn_TAK1, 2 Syn_NEMO, 2 T72, 2 Syn_IKK, Syn_LUBAC, 2 T73, Syn_NF_kB, 2 Syn_FADD, 4 Syn_Procasp8, 2 T1, 2 T2, T3, T4, T5, 2 diss6, T14, 2 T64, 2 CASP3_inhib, 2 T70, T7, T15, T8, T16, T9, T17, T13, 2 deg5, T22, T23, T24, deg7, Syn_SCF, T25, T26, T27, T30, 2 T31, 2 T32, 2 deg6, 2 T74, T39, T40, T10, T11, T12, T18, T19, Syn_CYLD, diss1, T21, T20, deg4, T38, diss2, T42, 2 T43, 2 T44, 2 T45, 2 T46, T49, deg1, Syn_IkB, 2 Syn_XIAP, 2 diss3, T6, 2 Syn_Procasp3, 2 Syn_Bid |
| 32. | 15, 18, 27, 34 | 4 Syn_BAX, 2 Syn_SMAC, 2 Syn_Cyt_c, 2 Syn_Apaf1, 3 Syn_Procasp9, 2 T65, 2 deg3, 2 T66, 2 T67, 2 Syn_TNFR1, 2 Syn_TNF, 2 Syn_TRADD, Syn_RIP1, 2 T69, Syn_TRAF2, Syn_cIAP, 2 T71, Syn_TAB, Syn_TAK1, 2 Syn_NEMO, 2 T72, 2 Syn_IKK, Syn_LUBAC, T73, Syn_NF_kB, 2 Syn_FADD, 4 Syn_Procasp8, T75, Pc9_inhib, 2 T1, 2 T2, T3, T4, T5, diss6, T14, T64, CASP3_inhib, 2 T70, T7, T15, T8, T16, T9, T17, T13, 2 deg5, T22, T23, T24, deg7, Syn_SCF, T25, T26, T27, T30, 2 T31, 2 T32, deg6, T74, T39, T40, T10, T11, T12, T18, T19, Syn_CYLD, diss1, T21, T20, deg4, T38, diss2, T53, T42, T43, T54, T61, T62, T44, T45, T46, T50, deg2, Syn_IkB, 2 Syn_XIAP, diss3, diss5, T6, Syn_Procasp3, 2 Syn_Bid |
| 33. | 15, 18, 27, 35 | 4 Syn_BAX, 2 Syn_SMAC, 2 Syn_Cyt_c, 2 Syn_Apaf1, 3 Syn_Procasp9, 2 T65, 2 deg3, 2 T66, 2 T67, 2 Syn_TNFR1, 2 Syn_TNF, 2 Syn_TRADD, Syn_RIP1, 2 T69, Syn_TRAF2, Syn_cIAP, 2 T71, Syn_TAB, Syn_TAK1, 2 Syn_NEMO, 2 T72, 2 Syn_IKK, Syn_LUBAC, T73, Syn_NF_kB, 2 Syn_FADD, 4 Syn_Procasp8, T75, Pc9_inhib, 2 T1, 2 T2, T3, T4, T5, diss6, T14, T64, CASP3_inhib, 2 T70, T7, T15, T8, T16, T9, T17, T13, 2 deg5, T22, T23, T24, deg7, Syn_SCF, T25, T26, T27, T30, 2 T31, 2 T32, deg6, T74, T39, T40, T10, T11, T12, T18, T19, Syn_CYLD, diss1, T21, T20, deg4, T38, diss2, T42, 2 T43, 2 T44, 2 T45, 2 T46, T49, deg1, Syn_IkB, 2 Syn_XIAP, 2 diss3, T6, Syn_Procasp3, 2 Syn_Bid |
| 34. | 12, 15, 18, 39 | 2 Syn_BAX, Syn_SMAC, Syn_Cyt_c, Syn_Apaf1, Syn_Procasp9, T65, 2 deg3, T66, T67, 2 Syn_TNFR1, 2 Syn_TNF, 2 Syn_TRADD, Syn_RIP1, T69, Syn_TRAF2, Syn_cIAP, T71, Syn_TAB, Syn_TAK1, 2 Syn_NEMO, T72, 2 Syn_IKK, Syn_LUBAC, Syn_NF_kB, 2 Syn_FADD, T76, 4 Syn_Procasp8, Apo_XIAP_inhib, T75, 2 T1, 2 T2, T3, T4, T5, T14, T64, CASP3_inhib, T70, T7, T15, T8, T16, T9, T17, T13, T22, T23, T24, deg7, Syn_SCF, T25, T26, T27, T30, 2 T31, 2 T32, T63, T39, T40, T10, T11, T12, T18, T19, Syn_CYLD, diss1, T21, T20, deg4, T38, diss2, T53, T42, T43, T54, T61, T62, T44, T45, T46, T50, deg2, Syn_IkB, 2 Syn_XIAP, diss3, diss5, T6, Syn_Procasp3, Syn_Bid |
| 35. | 12, 15, 18,40 | 2 Syn_BAX, Syn_SMAC, Syn_Cyt_c, Syn_Apaf1, Syn_Procasp9, T65, 2 deg3, T66, T67, 2 Syn_TNFR1, 2 Syn_TNF, 2 Syn_TRADD, Syn_RIP1, T69, Syn_TRAF2, Syn_cIAP, T71, Syn_TAB, Syn_TAK1, 2 Syn_NEMO, T72, 2 Syn_IKK, Syn_LUBAC, Syn_NF_kB, 2 Syn_FADD, T76, 4 Syn_Procasp8, Apo_XIAP_inhib, T75, 2 T1, 2 T2, T3, T4, T5, T14, T64, CASP3_inhib, T70, T7, T15, T8, T16, T9, T17, T13, T22, T23, T24, deg7, Syn_SCF, T25, T26, T27, T30, 2 T31, 2 T32, T63, T39, T40, T10, T11, T12, T18, T19, Syn_CYLD, diss1, T21, T20, deg4, T38, diss2, T42, 2 T43, 2 T44, 2 T45, 2 T46, T49, deg1, Syn_IkB, 2 Syn_XIAP, 2 diss3, T6, Syn_Procasp3, Syn_Bid |
| 36. | 12, 15, 18, 44 | Syn_BAX, T65, 2 deg3, T66, T67, T68, 2 Syn_TNFR1, 2 Syn_TNF, BAX_inhib, 2 Syn_TRADD, Syn_RIP1, Syn_TRAF2, Syn_cIAP, Syn_TAB, Syn_TAK1, 2 Syn_NEMO, 2 Syn_IKK, Syn_LUBAC, Syn_NF_kB, 2 Syn_FADD, 4 Syn_Procasp8, 2 T1, 2 T2, T3, T4, T5, T14, T64, CASP3_inhib, T7, T15, T8, T16, T9, T17, T13, T22, T23, T24, deg7, Syn_SCF, T25, T26, T27, T30, T31, T35, T32, T36, T63, T39, T40, T10, T11, T12, T18, T19, Syn_CYLD, diss1, T21, T20, deg4, T38, diss2, T53, T42, T43, T54, T61, T62, T44, T45, T46, T50, deg2, Syn_IkB, Syn_XIAP, Syn_BCL_2, diss3, diss5, T6, Syn_Procasp3, Syn_Bid |
| 37. | 15, 18, 19, 45 | Syn_BAX, T65, deg3, T66, T67, T68, 2 Syn_TNFR1, 2 Syn_TNF, BAX_inhib, 2 Syn_TRADD, 2 Syn_RIP1, Syn_TRAF2, Syn_cIAP, Syn_TAB, Syn_TAK1, 2 Syn_NEMO, 2 Syn_IKK, Syn_LUBAC, Syn_NF_kB, 2 Syn_FADD, 3 Syn_Procasp8, Syn_RIP3, 2 T1, 2 T2, 2 T3, T4, T5, T14, T60, RIP1_RIP3_inhib, T7, T15, T8, T16, T9, T17, T13, T22, T23, T24, deg7, Syn_SCF, T25, T26, T27, T30, T33, T35, T34, T36, T39, T40, T10, T11, T12, T18, T19, Syn_CYLD, diss1, T21, T20, deg4, T38, T48, diss2, T53, T43, T54, T59, Pc8_inhib, T44, T45, T46, T50, deg2, T49, deg1, Syn_IkB, Syn_cFLIPL, Syn_BCL_2, diss3, diss5, T6, Syn_Bid |
| 38. | 15, 18, 19, 44 | Syn_BAX, T65, deg3, T66, T67, T68, 2 Syn_TNFR1, 2 Syn_TNF, BAX_inhib, 2 Syn_TRADD, 2 Syn_RIP1, Syn_TRAF2, Syn_cIAP, Syn_TAB, Syn_TAK1, 2 Syn_NEMO, 2 Syn_IKK, Syn_LUBAC, Syn_NF_kB, 2 Syn_FADD, 3 Syn_Procasp8, Syn_RIP3, 2 T1, 2 T2, 2 T3, T4, T5, T14, T60, RIP1_RIP3_inhib, T7, T15, T8, T16, T9, T17, T13, T22, T23, T24, deg7, Syn_SCF, T25, T26, T27, T30, T33, T35, T34, T36, T39, T40, T10, T11, T12, T18, T19, Syn_CYLD, diss1, T21, T20, deg4, T38, T48, diss2, 2 T53, 2 T54, T61, T62, T59, Pc8_inhib, 2 T50, 2 deg2, Syn_IkB, Syn_cFLIPL, Syn_BCL_2, 2 diss5, T6, Syn_Bid |
| 39. | 15, 18, 19, 40 | 2 Syn_BAX, Syn_SMAC, Syn_Cyt_c, Syn_Apaf1, Syn_Procasp9, T65, deg3, T66, T67, 2 Syn_TNFR1, 2 Syn_TNF, 2 Syn_TRADD, 2 Syn_RIP1, T69, Syn_TRAF2, Syn_cIAP, T71, Syn_TAB, Syn_TAK1, 2 Syn_NEMO, T72, 2 Syn_IKK, Syn_LUBAC, Syn_NF_kB, 2 Syn_FADD, T76, 3 Syn_Procasp8, Apo_XIAP_inhib, Syn_RIP3, T75, 2 T1, 2 T2, 2 T3, T4, T5, T14, T60, RIP1_RIP3_inhib, T70, T7, T15, T8, T16, T9, T17, T13, T22, T23, T24, deg7, Syn_SCF, T25, T26, T27, T30, T31, T33, T32, T34, T39, T40, T10, T11, T12, T18, T19, Syn_CYLD, diss1, T21, T20, deg4, T38, T48, diss2, T53, T43, T54, T59, Pc8_inhib, T44, T45, T46, T50, deg2, T49, deg1, Syn_IkB, Syn_XIAP, Syn_cFLIPL, diss3, diss5, T6, Syn_Bid |
| 40. | 15, 18, 19, 39 | 2 Syn_BAX, Syn_SMAC, Syn_Cyt_c, Syn_Apaf1, Syn_Procasp9, T65, deg3, T66, T67, 2 Syn_TNFR1, 2 Syn_TNF, 2 Syn_TRADD, 2 Syn_RIP1, T69, Syn_TRAF2, Syn_cIAP, T71, Syn_TAB, Syn_TAK1, 2 Syn_NEMO, T72, 2 Syn_IKK, Syn_LUBAC, Syn_NF_kB, 2 Syn_FADD, T76, 3 Syn_Procasp8, Apo_XIAP_inhib, Syn_RIP3, T75, 2 T1, 2 T2, 2 T3, T4, T5, T14, T60, RIP1_RIP3_inhib, T70, T7, T15, T8, T16, T9, T17, T13, T22, T23, T24, deg7, Syn_SCF, T25, T26, T27, T30, T31, T33, T32, T34, T39, T40, T10, T11, T12, T18, T19, Syn_CYLD, diss1, T21, T20, deg4, T38, T48, diss2, 2 T53, 2 T54, T61, T62, T59, Pc8_inhib, 2 T50, 2 deg2, Syn_IkB, Syn_XIAP, Syn_cFLIPL, 2 diss5, T6, Syn_Bid |
| 41. | 15, 18, 20, 43 | Syn_BAX, T65, deg3, T66, T67, T68, 2 Syn_TNFR1, 2 Syn_TNF, BAX_inhib, 2 Syn_TRADD, 2 Syn_RIP1, Syn_TRAF2, Syn_cIAP, Syn_TAB, Syn_TAK1, 2 Syn_NEMO, 2 Syn_IKK, Syn_LUBAC, Syn_NF_kB, 2 Syn_FADD, 3 Syn_Procasp8, Syn_cFLIPs, Syn_RIP3, Syn_MLKL, 2 T1, 2 T2, 2 T3, T4, T5, T14, T58, T7, T15, T8, T16, T9, T17, T13, T22, T23, T24, deg7, Syn_SCF, T25, T26, T27, T30, T35, T36, T39, T40, T10, T11, T12, T18, T19, Syn_CYLD, diss1, T21, T20, deg4, T38, T48, diss2, T53, T43, T54, T44, T45, T55, T56, T57, Necroptosis, T46, T50, deg2, T49, deg1, Syn_IkB, Syn_BCL_2, diss3, diss5, T6, Syn_Bid |
| 42. | 15, 18, 20 | Syn_TNFR1, Syn_TNF, Syn_TRADD, Syn_RIP1, Syn_TRAF2, Syn_cIAP, Syn_TAB, Syn_TAK1, 2 Syn_NEMO, 2 Syn_IKK, Syn_LUBAC, Syn_NF_kB, Syn_FADD, Syn_Procasp8, Syn_cFLIPs, Syn_RIP3, Syn_MLKL, T1, T2, T3, T4, T5, T14, T58, T7, T15, T8, T16, T9, T17, T13, T22, T23, T24, deg7, Syn_SCF, T25, T26, T27, T30, T39, T40, T10, T11, T12, T18, T19, Syn_CYLD, diss1, T21, T20, deg4, T38, T53, T54, T55, T56, T57, Necroptosis, T50, deg2, Syn_IkB, diss5, T6 |
| 43. | 15, 18, 21 | 2 Syn_BAX, Syn_SMAC, Syn_Cyt_c, Syn_Apaf1, 2 Syn_Procasp9, Apoptosis, T65, deg3, T66, T67, Syn_TNFR1, Syn_TNF, Syn_TRADD, Syn_RIP1, T69, Syn_TRAF2, Syn_cIAP, T71, Syn_TAB, Syn_TAK1, 2 Syn_NEMO, T72, 2 Syn_IKK, Syn_LUBAC, T73, Syn_NF_kB, Syn_FADD, 2 Syn_Procasp8, T1, T2, T3, T4, T5, diss6, T14, T70, T7, T15, T8, T16, T9, T17, T13, deg5, T22, T23, T24, deg7, Syn_SCF, T25, T26, T27, T30, deg6, T74, T39, T40, T10, T11, T12, T18, T19, Syn_CYLD, diss1, T21, T20, deg4, T38, T43, T44, T45, T46, T49, deg1, Syn_IkB, diss3, T6, Syn_Procasp3, Syn_Bid |
| 44. | 15, 18, 22 | Syn_TNFR1, Syn_TNF, Syn_TRADD, Syn_RIP1, Syn_TRAF2, Syn_cIAP, Syn_TAB, Syn_TAK1, 2 Syn_NEMO, 2 Syn_IKK, Syn_LUBAC, Syn_NF_kB, Syn_FADD, Syn_Procasp8, Syn_RIP3, T1, T2, T3, T4, T5, T14, T60, RIP1_RIP3_inhib, T7, T15, T8, T16, T9, T17, T13, T22, T23, T24, deg7, Syn_SCF, T25, T26, T27, T30, T33, T34, T39, T40, T10, T11, T12, T18, T19, Syn_CYLD, diss1, T21, T20, deg4, T38, T53, T54, T59, Pc8_inhib, T50, deg2, Syn_IkB, Syn_cFLIPL, diss5, T6 |
| 45. | 15, 18, 23 | Syn_TNFR1, Syn_TNF, Syn_TRADD, Syn_RIP1, Syn_TRAF2, Syn_cIAP, Syn_TAB, Syn_TAK1, 2 Syn_NEMO, 2 Syn_IKK, Syn_LUBAC, Syn_NF_kB, Syn_FADD, Syn_Procasp8, T1, T2, T3, T4, T5, T14, T7, T15, T8, T16, T9, T17, T13, T22, T23, T24, deg7, Syn_SCF, T25, T26, T27, T30, T33, T34, T39, T40, T10, T11, T12, T18, T19, Syn_CYLD, diss1, T21, T20, deg4, T38, T43, T44, T47, CIIa_inhib, T49, deg1, Syn_IkB, Syn_cFLIPL, diss3, T6 |
| 46. (pure) | 46 | 2 Syn_BAX, Syn_SMAC, Syn_Cyt_c, Syn_Apaf1, 2 Syn_Procasp9, Apoptosis, T65, deg3, T66, T67, Syn_TNFR1, Syn_TNF, Syn_TRADD, Syn_RIP1, T69, T71, T72, T73, Syn_FADD, 2 Syn_Procasp8, T1, T2, T3, diss6, T70, deg5, deg6, T74, T48, diss2, T53, T54, T61, T62, T50, deg2, diss5, Syn_Procasp3, Syn_Bid |
| 47. (pure) | 47 | 2 Syn_BAX, Syn_SMAC, Syn_Cyt_c, Syn_Apaf1, 2 Syn_Procasp9, Apoptosis, T65, deg3, T66, T67, Syn_TNFR1, Syn_TNF, Syn_TRADD, T69, T71, T72, T73, Syn_FADD, 2 Syn_Procasp8, T1, T2, diss6, T70, deg5, deg6, T74, diss2, T42, T43, T44, T45, T46, diss3, Syn_Procasp3, Syn_Bid |
| 48. (pure) | 48 | 2 Syn_BAX, Syn_SMAC, Syn_Cyt_c, Syn_Apaf1, 2 Syn_Procasp9, Apoptosis, T65, deg3, T66, T67, Syn_TNFR1, Syn_TNF, Syn_TRADD, Syn_RIP1, T69, T71, T72, T73, Syn_FADD, 2 Syn_Procasp8, T1, T2, T3, diss6, T70, deg5, deg6, T74, T48, diss2, T43, T44, T45, T46, T49, deg1, diss3, Syn_Procasp3, Syn_Bid |
| 49. | 15, 18, 24 | 2 Syn_BAX, Syn_SMAC, Syn_Cyt_c, Syn_Apaf1, 2 Syn_Procasp9, Apoptosis, T65, deg3, T66, T67, Syn_TNFR1, Syn_TNF, Syn_TRADD, Syn_RIP1, T69, Syn_TRAF2, Syn_cIAP, T71, Syn_TAB, Syn_TAK1, 2 Syn_NEMO, T72, 2 Syn_IKK, Syn_LUBAC, T73, Syn_NF_kB, Syn_FADD, 2 Syn_Procasp8, T1, T2, T3, T4, T5, diss6, T14, T70, T7, T15, T8, T16, T9, T17, T13, deg5, T22, T23, T24, deg7, Syn_SCF, T25, T26, T27, T30, deg6, T74, T39, T40, T10, T11, T12, T18, T19, Syn_CYLD, diss1, T21, T20, deg4, T38, T53, T54, T61, T62, T50, deg2, Syn_IkB, diss5, T6, Syn_Procasp3, Syn_Bid |
| 50. | 15, 18, 25 | Syn_TNFR1, Syn_TNF, Syn_TRADD, Syn_RIP1, Syn_TRAF2, Syn_cIAP, Syn_TAB, Syn_TAK1, 2 Syn_NEMO, 2 Syn_IKK, Syn_LUBAC, Syn_NF_kB, Syn_RIP3, Syn_MLKL, T1, T2, T3, T4, T5, T14, T58, T51, T7, T15, T52, T8, T16, T9, T17, T13, T22, T23, T24, deg7, Syn_SCF, T25, T26, T27, T30, diss4, T39, T40, T10, T11, T12, T18, T19, Syn_CYLD, diss1, T21, T20, deg4, T38, Necroptosis, T50, deg2, Syn_IkB, T6 |
| 51. | 15, 18, 29 | 2 Syn_BAX, Syn_SMAC, Syn_Cyt_c, Syn_Apaf1, 2 Syn_Procasp9, T65, deg3, T66, T67, Syn_TNFR1, Syn_TNF, Syn_TRADD, Syn_RIP1, T69, Syn_TRAF2, Syn_cIAP, T71, Syn_TAB, Syn_TAK1, 2 Syn_NEMO, T72, 2 Syn_IKK, Syn_LUBAC, T73, Syn_NF_kB, Syn_FADD, 2 Syn_Procasp8, T1, T2, T3, T4, T5, diss6, T14, T64, CASP3_inhib, T70, T7, T15, T8, T16, T9, T17, T13, deg5, T22, T23, T24, deg7, Syn_SCF, T25, T26, T27, T30, T31, T32, deg6, T74, T39, T40, T10, T11, T12, T18, T19, Syn_CYLD, diss1, T21, T20, deg4, T38, T53, T54, T61, T62, T50, deg2, Syn_IkB, Syn_XIAP, diss5, T6, Syn_Procasp3, Syn_Bid |
| 52. | 7, 15, 18 | deg3, Syn_TNFR1, Syn_TNF, Syn_TRADD, Syn_RIP1, Syn_TRAF2, Syn_cIAP, Syn_TAB, Syn_TAK1, 2 Syn_NEMO, 2 Syn_IKK, Syn_LUBAC, Syn_NF_kB, Syn_FADD, 2 Syn_Procasp8, T1, T2, T3, T4, T5, T14, T64, CASP3_inhib, T7, T15, T8, T16, T9, T17, T13, T22, T23, T24, deg7, Syn_SCF, T25, T26, T27, T30, T31, T32, T63, T39, T40, T10, T11, T12, T18, T19, Syn_CYLD, diss1, T21, T20, deg4, T38, T43, T44, T45, T46, T49, deg1, Syn_IkB, Syn_XIAP, diss3, T6, Syn_Procasp3 |
| 53. | 15, 18, 30 | 2 Syn_BAX, Syn_SMAC, Syn_Cyt_c, Syn_Apaf1, 2 Syn_Procasp9, T65, deg3, T66, T67, Syn_TNFR1, Syn_TNF, Syn_TRADD, Syn_RIP1, T69, Syn_TRAF2, Syn_cIAP, T71, Syn_TAB, Syn_TAK1, 2 Syn_NEMO, T72, 2 Syn_IKK, Syn_LUBAC, T73, Syn_NF_kB, Syn_FADD, 2 Syn_Procasp8, T1, T2, T3, T4, T5, diss6, T14, T64, CASP3_inhib, T70, T7, T15, T8, T16, T9, T17, T13, deg5, T22, T23, T24, deg7, Syn_SCF, T25, T26, T27, T30, T31, T32, deg6, T74, T39, T40, T10, T11, T12, T18, T19, Syn_CYLD, diss1, T21, T20, deg4, T38, T43, T44, T45, T46, T49, deg1, Syn_IkB, Syn_XIAP, diss3, T6, Syn_Procasp3, Syn_Bid |
| 54. | 15, 18, 34 | 2 Syn_BAX, Syn_SMAC, Syn_Cyt_c, Syn_Apaf1, Syn_Procasp9, T65, deg3, T66, T67, Syn_TNFR1, Syn_TNF, Syn_TRADD, Syn_RIP1, T69, Syn_TRAF2, Syn_cIAP, T71, Syn_TAB, Syn_TAK1, 2 Syn_NEMO, T72, 2 Syn_IKK, Syn_LUBAC, Syn_NF_kB, Syn_FADD, 2 Syn_Procasp8, T75, Pc9_inhib, T1, T2, T3, T4, T5, T14, T70, T7, T15, T8, T16, T9, T17, T13, deg5, T22, T23, T24, deg7, Syn_SCF, T25, T26, T27, T30, T31, T32, T39, T40, T10, T11, T12, T18, T19, Syn_CYLD, diss1, T21, T20, deg4, T38, T53, T54, T61, T62, T50, deg2, Syn_IkB, Syn_XIAP, diss5, T6, Syn_Bid |
| 55. | 15, 18, 35 | 2 Syn_BAX, Syn_SMAC, Syn_Cyt_c, Syn_Apaf1, Syn_Procasp9, T65, deg3, T66, T67, Syn_TNFR1, Syn_TNF, Syn_TRADD, Syn_RIP1, T69, Syn_TRAF2, Syn_cIAP, T71, Syn_TAB, Syn_TAK1, 2 Syn_NEMO, T72, 2 Syn_IKK, Syn_LUBAC, Syn_NF_kB, Syn_FADD, 2 Syn_Procasp8, T75, Pc9_inhib, T1, T2, T3, T4, T5, T14, T70, T7, T15, T8, T16, T9, T17, T13, deg5, T22, T23, T24, deg7, Syn_SCF, T25, T26, T27, T30, T31, T32, T39, T40, T10, T11, T12, T18, T19, Syn_CYLD, diss1, T21, T20, deg4, T38, T43, T44, T45, T46, T49, deg1, Syn_IkB, Syn_XIAP, diss3, T6, Syn_Bid |
| 56. | 1, 15, 18 | Apoptosis, deg3, Syn_TNFR1, Syn_TNF, Syn_TRADD, Syn_RIP1, Syn_TRAF2, Syn_cIAP, Syn_TAB, Syn_TAK1, 2 Syn_NEMO, 2 Syn_IKK, Syn_LUBAC, Syn_NF_kB, Syn_FADD, 2 Syn_Procasp8, T1, T2, T3, T4, T5, T14, T7, T15, T8, T16, T9, T17, T13, T22, T23, T24, deg7, Syn_SCF, T25, T26, T27, T30, T63, T39, T40, T10, T11, T12, T18, T19, Syn_CYLD, diss1, T21, T20, deg4, T38, T43, T44, T45, T46, T49, deg1, Syn_IkB, diss3, T6, Syn_Procasp3 |
| 57. | 15, 18, 20, 42 | Syn_BAX, T65, deg3, T66, T67, T68, 2 Syn_TNFR1, 2 Syn_TNF, BAX_inhib, 2 Syn_TRADD, Syn_RIP1, Syn_TRAF2, Syn_cIAP, Syn_TAB, Syn_TAK1, 2 Syn_NEMO, 2 Syn_IKK, Syn_LUBAC, Syn_NF_kB, 2 Syn_FADD, 3 Syn_Procasp8, Syn_cFLIPs, Syn_RIP3, Syn_MLKL, 2 T1, 2 T2, T3, T4, T5, T14, T58, T7, T15, T8, T16, T9, T17, T13, T22, T23, T24, deg7, Syn_SCF, T25, T26, T27, T30, T35, T36, T39, T40, T10, T11, T12, T18, T19, Syn_CYLD, diss1, T21, T20, deg4, T38, diss2, T53, T42, T43, T54, T44, T45, T55, T56, T57, Necroptosis, T46, T50, deg2, Syn_IkB, Syn_BCL_2, diss3, diss5, T6, Syn_Bid |
| 58. | 2, 15, 18 | Apoptosis, deg3, Syn_TNFR1, Syn_TNF, Syn_TRADD, Syn_RIP1, Syn_TRAF2, Syn_cIAP, Syn_TAB, Syn_TAK1, 2 Syn_NEMO, 2 Syn_IKK, Syn_LUBAC, Syn_NF_kB, Syn_FADD, 2 Syn_Procasp8, T1, T2, T3, T4, T5, T14, T7, T15, T8, T16, T9, T17, T13, T22, T23, T24, deg7, Syn_SCF, T25, T26, T27, T30, T63, T39, T40, T10, T11, T12, T18, T19, Syn_CYLD, diss1, T21, T20, deg4, T38, T53, T54, T61, T62, T50, deg2, Syn_IkB, diss5, T6, Syn_Procasp3 |
| 59. | 15, 18, 20, 41 | Syn_BAX, T65, deg3, T66, T67, T68, 2 Syn_TNFR1, 2 Syn_TNF, BAX_inhib, 2 Syn_TRADD, 2 Syn_RIP1, Syn_TRAF2, Syn_cIAP, Syn_TAB, Syn_TAK1, 2 Syn_NEMO, 2 Syn_IKK, Syn_LUBAC, Syn_NF_kB, 2 Syn_FADD, 3 Syn_Procasp8, Syn_cFLIPs, Syn_RIP3, Syn_MLKL, 2 T1, 2 T2, 2 T3, T4, T5, T14, T58, T7, T15, T8, T16, T9, T17, T13, T22, T23, T24, deg7, Syn_SCF, T25, T26, T27, T30, T35, T36, T39, T40, T10, T11, T12, T18, T19, Syn_CYLD, diss1, T21, T20, deg4, T38, T48, diss2, 2 T53, 2 T54, T61, T62, T55, T56, T57, Necroptosis, 2 T50, 2 deg2, Syn_IkB, Syn_BCL_2, 2 diss5, T6, Syn_Bid |
| 60. | 15, 18, 20, 38 | 2 Syn_BAX, Syn_SMAC, Syn_Cyt_c, Syn_Apaf1, Syn_Procasp9, T65, deg3, T66, T67, 2 Syn_TNFR1, 2 Syn_TNF, 2 Syn_TRADD, 2 Syn_RIP1, T69, Syn_TRAF2, Syn_cIAP, T71, Syn_TAB, Syn_TAK1, 2 Syn_NEMO, T72, 2 Syn_IKK, Syn_LUBAC, Syn_NF_kB, 2 Syn_FADD, T76, 3 Syn_Procasp8, Syn_cFLIPs, Apo_XIAP_inhib, Syn_RIP3, T75, Syn_MLKL, 2 T1, 2 T2, 2 T3, T4, T5, T14, T58, T70, T7, T15, T8, T16, T9, T17, T13, T22, T23, T24, deg7, Syn_SCF, T25, T26, T27, T30, T31, T32, T39, T40, T10, T11, T12, T18, T19, Syn_CYLD, diss1, T21, T20, deg4, T38, T48, diss2, T53, T43, T54, T44, T45, T55, T56, T57, Necroptosis, T46, T50, deg2, T49, deg1, Syn_IkB, Syn_XIAP, diss3, diss5, T6, Syn_Bid |
| 61. | 15, 18, 20, 37 | 2 Syn_BAX, Syn_SMAC, Syn_Cyt_c, Syn_Apaf1, Syn_Procasp9, T65, deg3, T66, T67, 2 Syn_TNFR1, 2 Syn_TNF, 2 Syn_TRADD, Syn_RIP1, T69, Syn_TRAF2, Syn_cIAP, T71, Syn_TAB, Syn_TAK1, 2 Syn_NEMO, T72, 2 Syn_IKK, Syn_LUBAC, Syn_NF_kB, 2 Syn_FADD, T76, 3 Syn_Procasp8, Syn_cFLIPs, Apo_XIAP_inhib, Syn_RIP3, T75, Syn_MLKL, 2 T1, 2 T2, T3, T4, T5, T14, T58, T70, T7, T15, T8, T16, T9, T17, T13, T22, T23, T24, deg7, Syn_SCF, T25, T26, T27, T30, T31, T32, T39, T40, T10, T11, T12, T18, T19, Syn_CYLD, diss1, T21, T20, deg4, T38, diss2, T53, T42, T43, T54, T44, T45, T55, T56, T57, Necroptosis, T46, T50, deg2, Syn_IkB, Syn_XIAP, diss3, diss5, T6, Syn_Bid |
| 62. | 15, 18, 20, 36 | 2 Syn_BAX, Syn_SMAC, Syn_Cyt_c, Syn_Apaf1, Syn_Procasp9, T65, deg3, T66, T67, 2 Syn_TNFR1, 2 Syn_TNF, 2 Syn_TRADD, 2 Syn_RIP1, T69, Syn_TRAF2, Syn_cIAP, T71, Syn_TAB, Syn_TAK1, 2 Syn_NEMO, T72, 2 Syn_IKK, Syn_LUBAC, Syn_NF_kB, 2 Syn_FADD, T76, 3 Syn_Procasp8, Syn_cFLIPs, Apo_XIAP_inhib, Syn_RIP3, T75, Syn_MLKL, 2 T1, 2 T2, 2 T3, T4, T5, T14, T58, T70, T7, T15, T8, T16, T9, T17, T13, T22, T23, T24, deg7, Syn_SCF, T25, T26, T27, T30, T31, T32, T39, T40, T10, T11, T12, T18, T19, Syn_CYLD, diss1, T21, T20, deg4, T38, T48, diss2, 2 T53, 2 T54, T61, T62, T55, T56, T57, Necroptosis, 2 T50, 2 deg2, Syn_IkB, Syn_XIAP, 2 diss5, T6, Syn_Bid |
| 63. | 15, 18, 20, 33 | 2 Syn_BAX, Syn_SMAC, Syn_Cyt_c, Syn_Apaf1, Syn_Procasp9, T65, deg3, T66, T67, 2 Syn_TNFR1, 2 Syn_TNF, 2 Syn_TRADD, 2 Syn_RIP1, T69, Syn_TRAF2, Syn_cIAP, T71, Syn_TAB, Syn_TAK1, 2 Syn_NEMO, T72, 2 Syn_IKK, Syn_LUBAC, Syn_NF_kB, 2 Syn_FADD, 3 Syn_Procasp8, Syn_cFLIPs, Syn_RIP3, T75, Syn_MLKL, Pc9_inhib, 2 T1, 2 T2, 2 T3, T4, T5, T14, T58, T70, T7, T15, T8, T16, T9, T17, T13, deg5, T22, T23, T24, deg7, Syn_SCF, T25, T26, T27, T30, T31, T32, T39, T40, T10, T11, T12, T18, T19, Syn_CYLD, diss1, T21, T20, deg4, T38, T48, diss2, T53, T43, T54, T44, T45, T55, T56, T57, Necroptosis, T46, T50, deg2, T49, deg1, Syn_IkB, Syn_XIAP, diss3, diss5, T6, Syn_Bid |
| 64. | 15, 18, 20, 32 | 2 Syn_BAX, Syn_SMAC, Syn_Cyt_c, Syn_Apaf1, Syn_Procasp9, T65, deg3, T66, T67, 2 Syn_TNFR1, 2 Syn_TNF, 2 Syn_TRADD, Syn_RIP1, T69, Syn_TRAF2, Syn_cIAP, T71, Syn_TAB, Syn_TAK1, 2 Syn_NEMO, T72, 2 Syn_IKK, Syn_LUBAC, Syn_NF_kB, 2 Syn_FADD, 3 Syn_Procasp8, Syn_cFLIPs, Syn_RIP3, T75, Syn_MLKL, Pc9_inhib, 2 T1, 2 T2, T3, T4, T5, T14, T58, T70, T7, T15, T8, T16, T9, T17, T13, deg5, T22, T23, T24, deg7, Syn_SCF, T25, T26, T27, T30, T31, T32, T39, T40, T10, T11, T12, T18, T19, Syn_CYLD, diss1, T21, T20, deg4, T38, diss2, T53, T42, T43, T54, T44, T45, T55, T56, T57, Necroptosis, T46, T50, deg2, Syn_IkB, Syn_XIAP, diss3, diss5, T6, Syn_Bid |
| 65. | 15, 18, 20, 31 | 2 Syn_BAX, Syn_SMAC, Syn_Cyt_c, Syn_Apaf1, Syn_Procasp9, T65, deg3, T66, T67, 2 Syn_TNFR1, 2 Syn_TNF, 2 Syn_TRADD, 2 Syn_RIP1, T69, Syn_TRAF2, Syn_cIAP, T71, Syn_TAB, Syn_TAK1, 2 Syn_NEMO, T72, 2 Syn_IKK, Syn_LUBAC, Syn_NF_kB, 2 Syn_FADD, 3 Syn_Procasp8, Syn_cFLIPs, Syn_RIP3, T75, Syn_MLKL, Pc9_inhib, 2 T1, 2 T2, 2 T3, T4, T5, T14, T58, T70, T7, T15, T8, T16, T9, T17, T13, deg5, T22, T23, T24, deg7, Syn_SCF, T25, T26, T27, T30, T31, T32, T39, T40, T10, T11, T12, T18, T19, Syn_CYLD, diss1, T21, T20, deg4, T38, T48, diss2, 2 T53, 2 T54, T61, T62, T55, T56, T57, Necroptosis, 2 T50, 2 deg2, Syn_IkB, Syn_XIAP, 2 diss5, T6, Syn_Bid |
| 66. | 15, 18, 20, 28 | 2 Syn_BAX, Syn_SMAC, Syn_Cyt_c, Syn_Apaf1, 2 Syn_Procasp9, T65, deg3, T66, T67, 2 Syn_TNFR1, 2 Syn_TNF, 2 Syn_TRADD, 2 Syn_RIP1, T69, Syn_TRAF2, Syn_cIAP, T71, Syn_TAB, Syn_TAK1, 2 Syn_NEMO, T72, 2 Syn_IKK, Syn_LUBAC, T73, Syn_NF_kB, 2 Syn_FADD, 3 Syn_Procasp8, Syn_cFLIPs, Syn_RIP3, Syn_MLKL, 2 T1, 2 T2, 2 T3, T4, T5, diss6, T14, T58, T64, CASP3_inhib, T70, T7, T15, T8, T16, T9, T17, T13, deg5, T22, T23, T24, deg7, Syn_SCF, T25, T26, T27, T30, T31, T32, deg6, T74, T39, T40, T10, T11, T12, T18, T19, Syn_CYLD, diss1, T21, T20, deg4, T38, T48, diss2, T53, T43, T54, T44, T45, T55, T56, T57, Necroptosis, T46, T50, deg2, T49, deg1, Syn_IkB, Syn_XIAP, diss3, diss5, T6, Syn_Procasp3, Syn_Bid |
| 67. | 15, 18, 20, 27 | 2 Syn_BAX, Syn_SMAC, Syn_Cyt_c, Syn_Apaf1, 2 Syn_Procasp9, T65, deg3, T66, T67, 2 Syn_TNFR1, 2 Syn_TNF, 2 Syn_TRADD, Syn_RIP1, T69, Syn_TRAF2, Syn_cIAP, T71, Syn_TAB, Syn_TAK1, 2 Syn_NEMO, T72, 2 Syn_IKK, Syn_LUBAC, T73, Syn_NF_kB, 2 Syn_FADD, 3 Syn_Procasp8, Syn_cFLIPs, Syn_RIP3, Syn_MLKL, 2 T1, 2 T2, T3, T4, T5, diss6, T14, T58, T64, CASP3_inhib, T70, T7, T15, T8, T16, T9, T17, T13, deg5, T22, T23, T24, deg7, Syn_SCF, T25, T26, T27, T30, T31, T32, deg6, T74, T39, T40, T10, T11, T12, T18, T19, Syn_CYLD, diss1, T21, T20, deg4, T38, diss2, T53, T42, T43, T54, T44, T45, T55, T56, T57, Necroptosis, T46, T50, deg2, Syn_IkB, Syn_XIAP, diss3, diss5, T6, Syn_Procasp3, Syn_Bid |
| 68. | 15, 18, 20, 26 | 2 Syn_BAX, Syn_SMAC, Syn_Cyt_c, Syn_Apaf1, 2 Syn_Procasp9, T65, deg3, T66, T67, 2 Syn_TNFR1, 2 Syn_TNF, 2 Syn_TRADD, 2 Syn_RIP1, T69, Syn_TRAF2, Syn_cIAP, T71, Syn_TAB, Syn_TAK1, 2 Syn_NEMO, T72, 2 Syn_IKK, Syn_LUBAC, T73, Syn_NF_kB, 2 Syn_FADD, 3 Syn_Procasp8, Syn_cFLIPs, Syn_RIP3, Syn_MLKL, 2 T1, 2 T2, 2 T3, T4, T5, diss6, T14, T58, T64, CASP3_inhib, T70, T7, T15, T8, T16, T9, T17, T13, deg5, T22, T23, T24, deg7, Syn_SCF, T25, T26, T27, T30, T31, T32, deg6, T74, T39, T40, T10, T11, T12, T18, T19, Syn_CYLD, diss1, T21, T20, deg4, T38, T48, diss2, 2 T53, 2 T54, T61, T62, T55, T56, T57, Necroptosis, 2 T50, 2 deg2, Syn_IkB, Syn_XIAP, 2 diss5, T6, Syn_Procasp3, Syn_Bid |
| 69. | 15, 18, 21, 43 | 3 Syn_BAX, Syn_SMAC, Syn_Cyt_c, Syn_Apaf1, 2 Syn_Procasp9, Apoptosis, 2 T65, 2 deg3, 2 T66, 2 T67, T68, 2 Syn_TNFR1, 2 Syn_TNF, BAX_inhib, 2 Syn_TRADD, 2 Syn_RIP1, T69, Syn_TRAF2, Syn_cIAP, T71, Syn_TAB, Syn_TAK1, 2 Syn_NEMO, T72, 2 Syn_IKK, Syn_LUBAC, T73, Syn_NF_kB, 2 Syn_FADD, 4 Syn_Procasp8, 2 T1, 2 T2, 2 T3, T4, T5, diss6, T14, T70, T7, T15, T8, T16, T9, T17, T13, deg5, T22, T23, T24, deg7, Syn_SCF, T25, T26, T27, T30, T35, T36, deg6, T74, T39, T40, T10, T11, T12, T18, T19, Syn_CYLD, diss1, T21, T20, deg4, T38, T48, diss2, 2 T43, 2 T44, 2 T45, 2 T46, 2 T49, 2 deg1, Syn_IkB, Syn_BCL_2, 2 diss3, T6, Syn_Procasp3, 2 Syn_Bid |
| 70. | 15, 18, 21, 42 | 3 Syn_BAX, Syn_SMAC, Syn_Cyt_c, Syn_Apaf1, 2 Syn_Procasp9, Apoptosis, 2 T65, 2 deg3, 2 T66, 2 T67, T68, 2 Syn_TNFR1, 2 Syn_TNF, BAX_inhib, 2 Syn_TRADD, Syn_RIP1, T69, Syn_TRAF2, Syn_cIAP, T71, Syn_TAB, Syn_TAK1, 2 Syn_NEMO, T72, 2 Syn_IKK, Syn_LUBAC, T73, Syn_NF_kB, 2 Syn_FADD, 4 Syn_Procasp8, 2 T1, 2 T2, T3, T4, T5, diss6, T14, T70, T7, T15, T8, T16, T9, T17, T13, deg5, T22, T23, T24, deg7, Syn_SCF, T25, T26, T27, T30, T35, T36, deg6, T74, T39, T40, T10, T11, T12, T18, T19, Syn_CYLD, diss1, T21, T20, deg4, T38, diss2, T42, 2 T43, 2 T44, 2 T45, 2 T46, T49, deg1, Syn_IkB, Syn_BCL_2, 2 diss3, T6, Syn_Procasp3, 2 Syn_Bid |
| 71. | 15, 18, 21, 41 | 3 Syn_BAX, Syn_SMAC, Syn_Cyt_c, Syn_Apaf1, 2 Syn_Procasp9, Apoptosis, 2 T65, 2 deg3, 2 T66, 2 T67, T68, 2 Syn_TNFR1, 2 Syn_TNF, BAX_inhib, 2 Syn_TRADD, 2 Syn_RIP1, T69, Syn_TRAF2, Syn_cIAP, T71, Syn_TAB, Syn_TAK1, 2 Syn_NEMO, T72, 2 Syn_IKK, Syn_LUBAC, T73, Syn_NF_kB, 2 Syn_FADD, 4 Syn_Procasp8, 2 T1, 2 T2, 2 T3, T4, T5, diss6, T14, T70, T7, T15, T8, T16, T9, T17, T13, deg5, T22, T23, T24, deg7, Syn_SCF, T25, T26, T27, T30, T35, T36, deg6, T74, T39, T40, T10, T11, T12, T18, T19, Syn_CYLD, diss1, T21, T20, deg4, T38, T48, diss2, T53, T43, T54, T61, T62, T44, T45, T46, T50, deg2, T49, deg1, Syn_IkB, Syn_BCL_2, diss3, diss5, T6, Syn_Procasp3, 2 Syn_Bid |
| 72. | 15, 18, 21, 38 | 4 Syn_BAX, 2 Syn_SMAC, 2 Syn_Cyt_c, 2 Syn_Apaf1, 3 Syn_Procasp9, Apoptosis, 2 T65, 2 deg3, 2 T66, 2 T67, 2 Syn_TNFR1, 2 Syn_TNF, 2 Syn_TRADD, 2 Syn_RIP1, 2 T69, Syn_TRAF2, Syn_cIAP, 2 T71, Syn_TAB, Syn_TAK1, 2 Syn_NEMO, 2 T72, 2 Syn_IKK, Syn_LUBAC, T73, Syn_NF_kB, 2 Syn_FADD, T76, 4 Syn_Procasp8, Apo_XIAP_inhib, T75, 2 T1, 2 T2, 2 T3, T4, T5, diss6, T14, 2 T70, T7, T15, T8, T16, T9, T17, T13, deg5, T22, T23, T24, deg7, Syn_SCF, T25, T26, T27, T30, T31, T32, deg6, T74, T39, T40, T10, T11, T12, T18, T19, Syn_CYLD, diss1, T21, T20, deg4, T38, T48, diss2, 2 T43, 2 T44, 2 T45, 2 T46, 2 T49, 2 deg1, Syn_IkB, Syn_XIAP, 2 diss3, T6, Syn_Procasp3, 2 Syn_Bid |
| 73. | 15, 18, 21, 37 | 4 Syn_BAX, 2 Syn_SMAC, 2 Syn_Cyt_c, 2 Syn_Apaf1, 3 Syn_Procasp9, Apoptosis, 2 T65, 2 deg3, 2 T66, 2 T67, 2 Syn_TNFR1, 2 Syn_TNF, 2 Syn_TRADD, Syn_RIP1, 2 T69, Syn_TRAF2, Syn_cIAP, 2 T71, Syn_TAB, Syn_TAK1, 2 Syn_NEMO, 2 T72, 2 Syn_IKK, Syn_LUBAC, T73, Syn_NF_kB, 2 Syn_FADD, T76, 4 Syn_Procasp8, Apo_XIAP_inhib, T75, 2 T1, 2 T2, T3, T4, T5, diss6, T14, 2 T70, T7, T15, T8, T16, T9, T17, T13, deg5, T22, T23, T24, deg7, Syn_SCF, T25, T26, T27, T30, T31, T32, deg6, T74, T39, T40, T10, T11, T12, T18, T19, Syn_CYLD, diss1, T21, T20, deg4, T38, diss2, T42, 2 T43, 2 T44, 2 T45, 2 T46, T49, deg1, Syn_IkB, Syn_XIAP, 2 diss3, T6, Syn_Procasp3, 2 Syn_Bid |
| 74. | 15, 18, 21, 36 | 4 Syn_BAX, 2 Syn_SMAC, 2 Syn_Cyt_c, 2 Syn_Apaf1, 3 Syn_Procasp9, Apoptosis, 2 T65, 2 deg3, 2 T66, 2 T67, 2 Syn_TNFR1, 2 Syn_TNF, 2 Syn_TRADD, 2 Syn_RIP1, 2 T69, Syn_TRAF2, Syn_cIAP, 2 T71, Syn_TAB, Syn_TAK1, 2 Syn_NEMO, 2 T72, 2 Syn_IKK, Syn_LUBAC, T73, Syn_NF_kB, 2 Syn_FADD, T76, 4 Syn_Procasp8, Apo_XIAP_inhib, T75, 2 T1, 2 T2, 2 T3, T4, T5, diss6, T14, 2 T70, T7, T15, T8, T16, T9, T17, T13, deg5, T22, T23, T24, deg7, Syn_SCF, T25, T26, T27, T30, T31, T32, deg6, T74, T39, T40, T10, T11, T12, T18, T19, Syn_CYLD, diss1, T21, T20, deg4, T38, T48, diss2, T53, T43, T54, T61, T62, T44, T45, T46, T50, deg2, T49, deg1, Syn_IkB, Syn_XIAP, diss3, diss5, T6, Syn_Procasp3, 2 Syn_Bid |
| 75. | 15, 18, 21, 33 | 4 Syn_BAX, 2 Syn_SMAC, 2 Syn_Cyt_c, 2 Syn_Apaf1, 3 Syn_Procasp9, Apoptosis, 2 T65, 2 deg3, 2 T66, 2 T67, 2 Syn_TNFR1, 2 Syn_TNF, 2 Syn_TRADD, 2 Syn_RIP1, 2 T69, Syn_TRAF2, Syn_cIAP, 2 T71, Syn_TAB, Syn_TAK1, 2 Syn_NEMO, 2 T72, 2 Syn_IKK, Syn_LUBAC, T73, Syn_NF_kB, 2 Syn_FADD, 4 Syn_Procasp8, T75, Pc9_inhib, 2 T1, 2 T2, 2 T3, T4, T5, diss6, T14, 2 T70, T7, T15, T8, T16, T9, T17, T13, 2 deg5, T22, T23, T24, deg7, Syn_SCF, T25, T26, T27, T30, T31, T32, deg6, T74, T39, T40, T10, T11, T12, T18, T19, Syn_CYLD, diss1, T21, T20, deg4, T38, T48, diss2, 2 T43, 2 T44, 2 T45, 2 T46, 2 T49, 2 deg1, Syn_IkB, Syn_XIAP, 2 diss3, T6, Syn_Procasp3, 2 Syn_Bid |
| 76. | 15, 18, 21, 32 | 4 Syn_BAX, 2 Syn_SMAC, 2 Syn_Cyt_c, 2 Syn_Apaf1, 3 Syn_Procasp9, Apoptosis, 2 T65, 2 deg3, 2 T66, 2 T67, 2 Syn_TNFR1, 2 Syn_TNF, 2 Syn_TRADD, Syn_RIP1, 2 T69, Syn_TRAF2, Syn_cIAP, 2 T71, Syn_TAB, Syn_TAK1, 2 Syn_NEMO, 2 T72, 2 Syn_IKK, Syn_LUBAC, T73, Syn_NF_kB, 2 Syn_FADD, 4 Syn_Procasp8, T75, Pc9_inhib, 2 T1, 2 T2, T3, T4, T5, diss6, T14, 2 T70, T7, T15, T8, T16, T9, T17, T13, 2 deg5, T22, T23, T24, deg7, Syn_SCF, T25, T26, T27, T30, T31, T32, deg6, T74, T39, T40, T10, T11, T12, T18, T19, Syn_CYLD, diss1, T21, T20, deg4, T38, diss2, T42, 2 T43, 2 T44, 2 T45, 2 T46, T49, deg1, Syn_IkB, Syn_XIAP, 2 diss3, T6, Syn_Procasp3, 2 Syn_Bid |
| 77. | 15, 18, 21, 31 | 4 Syn_BAX, 2 Syn_SMAC, 2 Syn_Cyt_c, 2 Syn_Apaf1, 3 Syn_Procasp9, Apoptosis, 2 T65, 2 deg3, 2 T66, 2 T67, 2 Syn_TNFR1, 2 Syn_TNF, 2 Syn_TRADD, 2 Syn_RIP1, 2 T69, Syn_TRAF2, Syn_cIAP, 2 T71, Syn_TAB, Syn_TAK1, 2 Syn_NEMO, 2 T72, 2 Syn_IKK, Syn_LUBAC, T73, Syn_NF_kB, 2 Syn_FADD, 4 Syn_Procasp8, T75, Pc9_inhib, 2 T1, 2 T2, 2 T3, T4, T5, diss6, T14, 2 T70, T7, T15, T8, T16, T9, T17, T13, 2 deg5, T22, T23, T24, deg7, Syn_SCF, T25, T26, T27, T30, T31, T32, deg6, T74, T39, T40, T10, T11, T12, T18, T19, Syn_CYLD, diss1, T21, T20, deg4, T38, T48, diss2, T53, T43, T54, T61, T62, T44, T45, T46, T50, deg2, T49, deg1, Syn_IkB, Syn_XIAP, diss3, diss5, T6, Syn_Procasp3, 2 Syn_Bid |
| 78. | 15, 18, 21, 28 | 4 Syn_BAX, 2 Syn_SMAC, 2 Syn_Cyt_c, 2 Syn_Apaf1, 4 Syn_Procasp9, Apoptosis, 2 T65, 2 deg3, 2 T66, 2 T67, 2 Syn_TNFR1, 2 Syn_TNF, 2 Syn_TRADD, 2 Syn_RIP1, 2 T69, Syn_TRAF2, Syn_cIAP, 2 T71, Syn_TAB, Syn_TAK1, 2 Syn_NEMO, 2 T72, 2 Syn_IKK, Syn_LUBAC, 2 T73, Syn_NF_kB, 2 Syn_FADD, 4 Syn_Procasp8, 2 T1, 2 T2, 2 T3, T4, T5, 2 diss6, T14, T64, CASP3_inhib, 2 T70, T7, T15, T8, T16, T9, T17, T13, 2 deg5, T22, T23, T24, deg7, Syn_SCF, T25, T26, T27, T30, T31, T32, 2 deg6, 2 T74, T39, T40, T10, T11, T12, T18, T19, Syn_CYLD, diss1, T21, T20, deg4, T38, T48, diss2, 2 T43, 2 T44, 2 T45, 2 T46, 2 T49, 2 deg1, Syn_IkB, Syn_XIAP, 2 diss3, T6, 2 Syn_Procasp3, 2 Syn_Bid |
| 79. | 15, 18, 21, 27 | 4 Syn_BAX, 2 Syn_SMAC, 2 Syn_Cyt_c, 2 Syn_Apaf1, 4 Syn_Procasp9, Apoptosis, 2 T65, 2 deg3, 2 T66, 2 T67, 2 Syn_TNFR1, 2 Syn_TNF, 2 Syn_TRADD, Syn_RIP1, 2 T69, Syn_TRAF2, Syn_cIAP, 2 T71, Syn_TAB, Syn_TAK1, 2 Syn_NEMO, 2 T72, 2 Syn_IKK, Syn_LUBAC, 2 T73, Syn_NF_kB, 2 Syn_FADD, 4 Syn_Procasp8, 2 T1, 2 T2, T3, T4, T5, 2 diss6, T14, T64, CASP3_inhib, 2 T70, T7, T15, T8, T16, T9, T17, T13, 2 deg5, T22, T23, T24, deg7, Syn_SCF, T25, T26, T27, T30, T31, T32, 2 deg6, 2 T74, T39, T40, T10, T11, T12, T18, T19, Syn_CYLD, diss1, T21, T20, deg4, T38, diss2, T42, 2 T43, 2 T44, 2 T45, 2 T46, T49, deg1, Syn_IkB, Syn_XIAP, 2 diss3, T6, 2 Syn_Procasp3, 2 Syn_Bid |
| 80. | 15, 17, 18, 39 | 2 Syn_BAX, Syn_SMAC, Syn_Cyt_c, Syn_Apaf1, Syn_Procasp9, T65, deg3, T66, T67, 2 Syn_TNFR1, 2 Syn_TNF, 2 Syn_TRADD, 2 Syn_RIP1, T69, Syn_TRAF2, Syn_cIAP, T71, Syn_TAB, Syn_TAK1, 2 Syn_NEMO, T72, 2 Syn_IKK, Syn_LUBAC, Syn_NF_kB, 2 Syn_FADD, T76, 3 Syn_Procasp8, Apo_XIAP_inhib, T75, 2 T1, 2 T2, 2 T3, T4, T5, T14, T70, T7, T15, T8, T16, T9, T17, T13, T22, T23, T24, deg7, Syn_SCF, T25, T26, T27, T30, T31, T33, T32, T34, T39, T40, T10, T11, T12, T18, T19, Syn_CYLD, diss1, T21, T20, deg4, T38, T48, diss2, T53, T43, T54, T61, T62, T44, T47, CIIa_inhib, T50, deg2, T49, deg1, Syn_IkB, Syn_XIAP, Syn_cFLIPL, diss3, diss5, T6, Syn_Bid |
| 81. | 15, 17, 18, 40 | 2 Syn_BAX, Syn_SMAC, Syn_Cyt_c, Syn_Apaf1, Syn_Procasp9, T65, deg3, T66, T67, 2 Syn_TNFR1, 2 Syn_TNF, 2 Syn_TRADD, 2 Syn_RIP1, T69, Syn_TRAF2, Syn_cIAP, T71, Syn_TAB, Syn_TAK1, 2 Syn_NEMO, T72, 2 Syn_IKK, Syn_LUBAC, Syn_NF_kB, 2 Syn_FADD, T76, 3 Syn_Procasp8, Apo_XIAP_inhib, T75, 2 T1, 2 T2, 2 T3, T4, T5, T14, T70, T7, T15, T8, T16, T9, T17, T13, T22, T23, T24, deg7, Syn_SCF, T25, T26, T27, T30, T31, T33, T32, T34, T39, T40, T10, T11, T12, T18, T19, Syn_CYLD, diss1, T21, T20, deg4, T38, T48, diss2, 2 T43, 2 T44, T45, T46, T47, CIIa_inhib, 2 T49, 2 deg1, Syn_IkB, Syn_XIAP, Syn_cFLIPL, 2 diss3, T6, Syn_Bid |
| 82. | 15, 17, 18, 44 | Syn_BAX, T65, deg3, T66, T67, T68, 2 Syn_TNFR1, 2 Syn_TNF, BAX_inhib, 2 Syn_TRADD, 2 Syn_RIP1, Syn_TRAF2, Syn_cIAP, Syn_TAB, Syn_TAK1, 2 Syn_NEMO, 2 Syn_IKK, Syn_LUBAC, Syn_NF_kB, 2 Syn_FADD, 3 Syn_Procasp8, 2 T1, 2 T2, 2 T3, T4, T5, T14, T7, T15, T8, T16, T9, T17, T13, T22, T23, T24, deg7, Syn_SCF, T25, T26, T27, T30, T33, T35, T34, T36, T39, T40, T10, T11, T12, T18, T19, Syn_CYLD, diss1, T21, T20, deg4, T38, T48, diss2, T53, T43, T54, T61, T62, T44, T47, CIIa_inhib, T50, deg2, T49, deg1, Syn_IkB, Syn_cFLIPL, Syn_BCL_2, diss3, diss5, T6, Syn_Bid |
| 83. | 15, 17, 18, 45 | Syn_BAX, T65, deg3, T66, T67, T68, 2 Syn_TNFR1, 2 Syn_TNF, BAX_inhib, 2 Syn_TRADD, 2 Syn_RIP1, Syn_TRAF2, Syn_cIAP, Syn_TAB, Syn_TAK1, 2 Syn_NEMO, 2 Syn_IKK, Syn_LUBAC, Syn_NF_kB, 2 Syn_FADD, 3 Syn_Procasp8, 2 T1, 2 T2, 2 T3, T4, T5, T14, T7, T15, T8, T16, T9, T17, T13, T22, T23, T24, deg7, Syn_SCF, T25, T26, T27, T30, T33, T35, T34, T36, T39, T40, T10, T11, T12, T18, T19, Syn_CYLD, diss1, T21, T20, deg4, T38, T48, diss2, 2 T43, 2 T44, T45, T46, T47, CIIa_inhib, 2 T49, 2 deg1, Syn_IkB, Syn_cFLIPL, Syn_BCL_2, 2 diss3, T6, Syn_Bid |
| 84. | 15, 18, 21, 26 | 4 Syn_BAX, 2 Syn_SMAC, 2 Syn_Cyt_c, 2 Syn_Apaf1, 4 Syn_Procasp9, Apoptosis, 2 T65, 2 deg3, 2 T66, 2 T67, 2 Syn_TNFR1, 2 Syn_TNF, 2 Syn_TRADD, 2 Syn_RIP1, 2 T69, Syn_TRAF2, Syn_cIAP, 2 T71, Syn_TAB, Syn_TAK1, 2 Syn_NEMO, 2 T72, 2 Syn_IKK, Syn_LUBAC, 2 T73, Syn_NF_kB, 2 Syn_FADD, 4 Syn_Procasp8, 2 T1, 2 T2, 2 T3, T4, T5, 2 diss6, T14, T64, CASP3_inhib, 2 T70, T7, T15, T8, T16, T9, T17, T13, 2 deg5, T22, T23, T24, deg7, Syn_SCF, T25, T26, T27, T30, T31, T32, 2 deg6, 2 T74, T39, T40, T10, T11, T12, T18, T19, Syn_CYLD, diss1, T21, T20, deg4, T38, T48, diss2, T53, T43, T54, T61, T62, T44, T45, T46, T50, deg2, T49, deg1, Syn_IkB, Syn_XIAP, diss3, diss5, T6, 2 Syn_Procasp3, 2 Syn_Bid |
| 85. | 12, 15, 18, 45 | Syn_BAX, T65, 2 deg3, T66, T67, T68, 2 Syn_TNFR1, 2 Syn_TNF, BAX_inhib, 2 Syn_TRADD, Syn_RIP1, Syn_TRAF2, Syn_cIAP, Syn_TAB, Syn_TAK1, 2 Syn_NEMO, 2 Syn_IKK, Syn_LUBAC, Syn_NF_kB, 2 Syn_FADD, 4 Syn_Procasp8, 2 T1, 2 T2, T3, T4, T5, T14, T64, CASP3_inhib, T7, T15, T8, T16, T9, T17, T13, T22, T23, T24, deg7, Syn_SCF, T25, T26, T27, T30, T31, T35, T32, T36, T63, T39, T40, T10, T11, T12, T18, T19, Syn_CYLD, diss1, T21, T20, deg4, T38, diss2, T42, 2 T43, 2 T44, 2 T45, 2 T46, T49, deg1, Syn_IkB, Syn_XIAP, Syn_BCL_2, 2 diss3, T6, Syn_Procasp3, Syn_Bid |
| 86. | 15, 18, 27, 45 | 3 Syn_BAX, Syn_SMAC, Syn_Cyt_c, Syn_Apaf1, 2 Syn_Procasp9, 2 T65, 2 deg3, 2 T66, 2 T67, T68, 2 Syn_TNFR1, 2 Syn_TNF, BAX_inhib, 2 Syn_TRADD, Syn_RIP1, T69, Syn_TRAF2, Syn_cIAP, T71, Syn_TAB, Syn_TAK1, 2 Syn_NEMO, T72, 2 Syn_IKK, Syn_LUBAC, T73, Syn_NF_kB, 2 Syn_FADD, 4 Syn_Procasp8, 2 T1, 2 T2, T3, T4, T5, diss6, T14, T64, CASP3_inhib, T70, T7, T15, T8, T16, T9, T17, T13, deg5, T22, T23, T24, deg7, Syn_SCF, T25, T26, T27, T30, T31, T35, T32, T36, deg6, T74, T39, T40, T10, T11, T12, T18, T19, Syn_CYLD, diss1, T21, T20, deg4, T38, diss2, T42, 2 T43, 2 T44, 2 T45, 2 T46, T49, deg1, Syn_IkB, Syn_XIAP, Syn_BCL_2, 2 diss3, T6, Syn_Procasp3, 2 Syn_Bid |
| 87. | 15, 18, 27, 44 | 3 Syn_BAX, Syn_SMAC, Syn_Cyt_c, Syn_Apaf1, 2 Syn_Procasp9, 2 T65, 2 deg3, 2 T66, 2 T67, T68, 2 Syn_TNFR1, 2 Syn_TNF, BAX_inhib, 2 Syn_TRADD, Syn_RIP1, T69, Syn_TRAF2, Syn_cIAP, T71, Syn_TAB, Syn_TAK1, 2 Syn_NEMO, T72, 2 Syn_IKK, Syn_LUBAC, T73, Syn_NF_kB, 2 Syn_FADD, 4 Syn_Procasp8, 2 T1, 2 T2, T3, T4, T5, diss6, T14, T64, CASP3_inhib, T70, T7, T15, T8, T16, T9, T17, T13, deg5, T22, T23, T24, deg7, Syn_SCF, T25, T26, T27, T30, T31, T35, T32, T36, deg6, T74, T39, T40, T10, T11, T12, T18, T19, Syn_CYLD, diss1, T21, T20, deg4, T38, diss2, T53, T42, T43, T54, T61, T62, T44, T45, T46, T50, deg2, Syn_IkB, Syn_XIAP, Syn_BCL_2, diss3, diss5, T6, Syn_Procasp3, 2 Syn_Bid |
| 88. | 15, 18, 45 | Syn_BAX, T65, deg3, T66, T67, T68, Syn_TNFR1, Syn_TNF, BAX_inhib, Syn_TRADD, Syn_RIP1, Syn_TRAF2, Syn_cIAP, Syn_TAB, Syn_TAK1, 2 Syn_NEMO, 2 Syn_IKK, Syn_LUBAC, Syn_NF_kB, Syn_FADD, 2 Syn_Procasp8, T1, T2, T3, T4, T5, T14, T7, T15, T8, T16, T9, T17, T13, T22, T23, T24, deg7, Syn_SCF, T25, T26, T27, T30, T35, T36, T39, T40, T10, T11, T12, T18, T19, Syn_CYLD, diss1, T21, T20, deg4, T38, T43, T44, T45, T46, T49, deg1, Syn_IkB, Syn_BCL_2, diss3, T6, Syn_Bid |
| 89. | 15, 18, 44 | Syn_BAX, T65, deg3, T66, T67, T68, Syn_TNFR1, Syn_TNF, BAX_inhib, Syn_TRADD, Syn_RIP1, Syn_TRAF2, Syn_cIAP, Syn_TAB, Syn_TAK1, 2 Syn_NEMO, 2 Syn_IKK, Syn_LUBAC, Syn_NF_kB, Syn_FADD, 2 Syn_Procasp8, T1, T2, T3, T4, T5, T14, T7, T15, T8, T16, T9, T17, T13, T22, T23, T24, deg7, Syn_SCF, T25, T26, T27, T30, T35, T36, T39, T40, T10, T11, T12, T18, T19, Syn_CYLD, diss1, T21, T20, deg4, T38, T53, T54, T61, T62, T50, deg2, Syn_IkB, Syn_BCL_2, diss5, T6, Syn_Bid |
| 90. | 15, 18, 40 | 2 Syn_BAX, Syn_SMAC, Syn_Cyt_c, Syn_Apaf1, Syn_Procasp9, T65, deg3, T66, T67, Syn_TNFR1, Syn_TNF, Syn_TRADD, Syn_RIP1, T69, Syn_TRAF2, Syn_cIAP, T71, Syn_TAB, Syn_TAK1, 2 Syn_NEMO, T72, 2 Syn_IKK, Syn_LUBAC, Syn_NF_kB, Syn_FADD, T76, 2 Syn_Procasp8, Apo_XIAP_inhib, T75, T1, T2, T3, T4, T5, T14, T70, T7, T15, T8, T16, T9, T17, T13, T22, T23, T24, deg7, Syn_SCF, T25, T26, T27, T30, T31, T32, T39, T40, T10, T11, T12, T18, T19, Syn_CYLD, diss1, T21, T20, deg4, T38, T43, T44, T45, T46, T49, deg1, Syn_IkB, Syn_XIAP, diss3, T6, Syn_Bid |
| 91. | 15, 18, 39 | 2 Syn_BAX, Syn_SMAC, Syn_Cyt_c, Syn_Apaf1, Syn_Procasp9, T65, deg3, T66, T67, Syn_TNFR1, Syn_TNF, Syn_TRADD, Syn_RIP1, T69, Syn_TRAF2, Syn_cIAP, T71, Syn_TAB, Syn_TAK1, 2 Syn_NEMO, T72, 2 Syn_IKK, Syn_LUBAC, Syn_NF_kB, Syn_FADD, T76, 2 Syn_Procasp8, Apo_XIAP_inhib, T75, T1, T2, T3, T4, T5, T14, T70, T7, T15, T8, T16, T9, T17, T13, T22, T23, T24, deg7, Syn_SCF, T25, T26, T27, T30, T31, T32, T39, T40, T10, T11, T12, T18, T19, Syn_CYLD, diss1, T21, T20, deg4, T38, T53, T54, T61, T62, T50, deg2, Syn_IkB, Syn_XIAP, diss5, T6, Syn_Bid |
| 92. | 1, 11, 15, 18 | Apoptosis, 2 deg3, 2 Syn_TNFR1, 2 Syn_TNF, 2 Syn_TRADD, 2 Syn_RIP1, Syn_TRAF2, Syn_cIAP, Syn_TAB, Syn_TAK1, 2 Syn_NEMO, 2 Syn_IKK, Syn_LUBAC, Syn_NF_kB, 2 Syn_FADD, 4 Syn_Procasp8, 2 T1, 2 T2, 2 T3, T4, T5, T14, T64, CASP3_inhib, T7, T15, T8, T16, T9, T17, T13, T22, T23, T24, deg7, Syn_SCF, T25, T26, T27, T30, T31, T32, 2 T63, T39, T40, T10, T11, T12, T18, T19, Syn_CYLD, diss1, T21, T20, deg4, T38, T48, diss2, 2 T43, 2 T44, 2 T45, 2 T46, 2 T49, 2 deg1, Syn_IkB, Syn_XIAP, 2 diss3, T6, 2 Syn_Procasp3 |
| 93. | 1, 12, 15, 18 | Apoptosis, 2 deg3, 2 Syn_TNFR1, 2 Syn_TNF, 2 Syn_TRADD, Syn_RIP1, Syn_TRAF2, Syn_cIAP, Syn_TAB, Syn_TAK1, 2 Syn_NEMO, 2 Syn_IKK, Syn_LUBAC, Syn_NF_kB, 2 Syn_FADD, 4 Syn_Procasp8, 2 T1, 2 T2, T3, T4, T5, T14, T64, CASP3_inhib, T7, T15, T8, T16, T9, T17, T13, T22, T23, T24, deg7, Syn_SCF, T25, T26, T27, T30, T31, T32, 2 T63, T39, T40, T10, T11, T12, T18, T19, Syn_CYLD, diss1, T21, T20, deg4, T38, diss2, T42, 2 T43, 2 T44, 2 T45, 2 T46, T49, deg1, Syn_IkB, Syn_XIAP, 2 diss3, T6, 2 Syn_Procasp3 |
| 94. | 1, 14, 15, 18 | Apoptosis, 2 deg3, 2 Syn_TNFR1, 2 Syn_TNF, 2 Syn_TRADD, 2 Syn_RIP1, Syn_TRAF2, Syn_cIAP, Syn_TAB, Syn_TAK1, 2 Syn_NEMO, 2 Syn_IKK, Syn_LUBAC, Syn_NF_kB, 2 Syn_FADD, 4 Syn_Procasp8, 2 T1, 2 T2, 2 T3, T4, T5, T14, T64, CASP3_inhib, T7, T15, T8, T16, T9, T17, T13, T22, T23, T24, deg7, Syn_SCF, T25, T26, T27, T30, T31, T32, 2 T63, T39, T40, T10, T11, T12, T18, T19, Syn_CYLD, diss1, T21, T20, deg4, T38, T48, diss2, T53, T43, T54, T61, T62, T44, T45, T46, T50, deg2, T49, deg1, Syn_IkB, Syn_XIAP, diss3, diss5, T6, 2 Syn_Procasp3 |
| 95. | 1, 15, 16, 18 | Apoptosis, deg3, Syn_TNFR1, Syn_TNF, Syn_TRADD, Syn_RIP1, Syn_TRAF2, Syn_cIAP, Syn_TAB, Syn_TAK1, 2 Syn_NEMO, 2 Syn_IKK, Syn_LUBAC, Syn_NF_kB, Syn_FADD, 2 Syn_Procasp8, T1, T2, T3, T4, T5, T14, T7, T15, T8, T16, T9, T17, T13, 2 T22, T37, 2 T23, 2 T24, 2 deg7, 2 Syn_SCF, T25, 2 T26, 2 T27, T30, T63, T39, T40, T10, T11, T12, T18, T19, Syn_CYLD, diss1, T21, T20, deg4, T38, T43, T44, T45, T46, T49, deg1, 2 Syn_IkB, diss3, T6, Syn_Procasp3 |
| 96. | 1, 15, 17, 18 | Apoptosis, deg3, 2 Syn_TNFR1, 2 Syn_TNF, 2 Syn_TRADD, 2 Syn_RIP1, Syn_TRAF2, Syn_cIAP, Syn_TAB, Syn_TAK1, 2 Syn_NEMO, 2 Syn_IKK, Syn_LUBAC, Syn_NF_kB, 2 Syn_FADD, 3 Syn_Procasp8, 2 T1, 2 T2, 2 T3, T4, T5, T14, T7, T15, T8, T16, T9, T17, T13, T22, T23, T24, deg7, Syn_SCF, T25, T26, T27, T30, T33, T34, T63, T39, T40, T10, T11, T12, T18, T19, Syn_CYLD, diss1, T21, T20, deg4, T38, T48, diss2, 2 T43, 2 T44, T45, T46, T47, CIIa_inhib, 2 T49, 2 deg1, Syn_IkB, Syn_cFLIPL, 2 diss3, T6, Syn_Procasp3 |
| 97. | 1, 15, 18, 43 | Syn_BAX, Apoptosis, T65, 2 deg3, T66, T67, T68, 2 Syn_TNFR1, 2 Syn_TNF, BAX_inhib, 2 Syn_TRADD, 2 Syn_RIP1, Syn_TRAF2, Syn_cIAP, Syn_TAB, Syn_TAK1, 2 Syn_NEMO, 2 Syn_IKK, Syn_LUBAC, Syn_NF_kB, 2 Syn_FADD, 4 Syn_Procasp8, 2 T1, 2 T2, 2 T3, T4, T5, T14, T7, T15, T8, T16, T9, T17, T13, T22, T23, T24, deg7, Syn_SCF, T25, T26, T27, T30, T35, T36, T63, T39, T40, T10, T11, T12, T18, T19, Syn_CYLD, diss1, T21, T20, deg4, T38, T48, diss2, 2 T43, 2 T44, 2 T45, 2 T46, 2 T49, 2 deg1, Syn_IkB, Syn_BCL_2, 2 diss3, T6, Syn_Procasp3, Syn_Bid |
| 98. | 1, 15, 18, 42 | Syn_BAX, Apoptosis, T65, 2 deg3, T66, T67, T68, 2 Syn_TNFR1, 2 Syn_TNF, BAX_inhib, 2 Syn_TRADD, Syn_RIP1, Syn_TRAF2, Syn_cIAP, Syn_TAB, Syn_TAK1, 2 Syn_NEMO, 2 Syn_IKK, Syn_LUBAC, Syn_NF_kB, 2 Syn_FADD, 4 Syn_Procasp8, 2 T1, 2 T2, T3, T4, T5, T14, T7, T15, T8, T16, T9, T17, T13, T22, T23, T24, deg7, Syn_SCF, T25, T26, T27, T30, T35, T36, T63, T39, T40, T10, T11, T12, T18, T19, Syn_CYLD, diss1, T21, T20, deg4, T38, diss2, T42, 2 T43, 2 T44, 2 T45, 2 T46, T49, deg1, Syn_IkB, Syn_BCL_2, 2 diss3, T6, Syn_Procasp3, Syn_Bid |
| 99. | 1, 15, 18, 41 | Syn_BAX, Apoptosis, T65, 2 deg3, T66, T67, T68, 2 Syn_TNFR1, 2 Syn_TNF, BAX_inhib, 2 Syn_TRADD, 2 Syn_RIP1, Syn_TRAF2, Syn_cIAP, Syn_TAB, Syn_TAK1, 2 Syn_NEMO, 2 Syn_IKK, Syn_LUBAC, Syn_NF_kB, 2 Syn_FADD, 4 Syn_Procasp8, 2 T1, 2 T2, 2 T3, T4, T5, T14, T7, T15, T8, T16, T9, T17, T13, T22, T23, T24, deg7, Syn_SCF, T25, T26, T27, T30, T35, T36, T63, T39, T40, T10, T11, T12, T18, T19, Syn_CYLD, diss1, T21, T20, deg4, T38, T48, diss2, T53, T43, T54, T61, T62, T44, T45, T46, T50, deg2, T49, deg1, Syn_IkB, Syn_BCL_2, diss3, diss5, T6, Syn_Procasp3, Syn_Bid |
| 100. | 1, 15, 18, 38 | 2 Syn_BAX, Syn_SMAC, Syn_Cyt_c, Syn_Apaf1, Syn_Procasp9, Apoptosis, T65, 2 deg3, T66, T67, 2 Syn_TNFR1, 2 Syn_TNF, 2 Syn_TRADD, 2 Syn_RIP1, T69, Syn_TRAF2, Syn_cIAP, T71, Syn_TAB, Syn_TAK1, 2 Syn_NEMO, T72, 2 Syn_IKK, Syn_LUBAC, Syn_NF_kB, 2 Syn_FADD, T76, 4 Syn_Procasp8, Apo_XIAP_inhib, T75, 2 T1, 2 T2, 2 T3, T4, T5, T14, T70, T7, T15, T8, T16, T9, T17, T13, T22, T23, T24, deg7, Syn_SCF, T25, T26, T27, T30, T31, T32, T63, T39, T40, T10, T11, T12, T18, T19, Syn_CYLD, diss1, T21, T20, deg4, T38, T48, diss2, 2 T43, 2 T44, 2 T45, 2 T46, 2 T49, 2 deg1, Syn_IkB, Syn_XIAP, 2 diss3, T6, Syn_Procasp3, Syn_Bid |
| 101. | 1, 15, 18, 37 | 2 Syn_BAX, Syn_SMAC, Syn_Cyt_c, Syn_Apaf1, Syn_Procasp9, Apoptosis, T65, 2 deg3, T66, T67, 2 Syn_TNFR1, 2 Syn_TNF, 2 Syn_TRADD, Syn_RIP1, T69, Syn_TRAF2, Syn_cIAP, T71, Syn_TAB, Syn_TAK1, 2 Syn_NEMO, T72, 2 Syn_IKK, Syn_LUBAC, Syn_NF_kB, 2 Syn_FADD, T76, 4 Syn_Procasp8, Apo_XIAP_inhib, T75, 2 T1, 2 T2, T3, T4, T5, T14, T70, T7, T15, T8, T16, T9, T17, T13, T22, T23, T24, deg7, Syn_SCF, T25, T26, T27, T30, T31, T32, T63, T39, T40, T10, T11, T12, T18, T19, Syn_CYLD, diss1, T21, T20, deg4, T38, diss2, T42, 2 T43, 2 T44, 2 T45, 2 T46, T49, deg1, Syn_IkB, Syn_XIAP, 2 diss3, T6, Syn_Procasp3, Syn_Bid |
| 102. | 1, 4, 15, 18 | Apoptosis, deg3, 2 Syn_TNFR1, 2 Syn_TNF, 2 Syn_TRADD, Syn_RIP1, Syn_TRAF2, Syn_cIAP, Syn_TAB, Syn_TAK1, 2 Syn_NEMO, 2 Syn_IKK, Syn_LUBAC, Syn_NF_kB, 2 Syn_FADD, 3 Syn_Procasp8, 2 T1, 2 T2, T3, T4, T5, T14, T7, T15, T8, T16, T9, T17, T13, T22, T23, T24, deg7, Syn_SCF, T25, T26, T27, T30, T33, T34, T63, T39, T40, T10, T11, T12, T18, T19, Syn_CYLD, diss1, T21, T20, deg4, T38, diss2, T42, 2 T43, 2 T44, T45, T46, T47, CIIa_inhib, T49, deg1, Syn_IkB, Syn_cFLIPL, 2 diss3, T6, Syn_Procasp3 |
| 103. | 1, 15, 18, 36 | 2 Syn_BAX, Syn_SMAC, Syn_Cyt_c, Syn_Apaf1, Syn_Procasp9, Apoptosis, T65, 2 deg3, T66, T67, 2 Syn_TNFR1, 2 Syn_TNF, 2 Syn_TRADD, 2 Syn_RIP1, T69, Syn_TRAF2, Syn_cIAP, T71, Syn_TAB, Syn_TAK1, 2 Syn_NEMO, T72, 2 Syn_IKK, Syn_LUBAC, Syn_NF_kB, 2 Syn_FADD, T76, 4 Syn_Procasp8, Apo_XIAP_inhib, T75, 2 T1, 2 T2, 2 T3, T4, T5, T14, T70, T7, T15, T8, T16, T9, T17, T13, T22, T23, T24, deg7, Syn_SCF, T25, T26, T27, T30, T31, T32, T63, T39, T40, T10, T11, T12, T18, T19, Syn_CYLD, diss1, T21, T20, deg4, T38, T48, diss2, T53, T43, T54, T61, T62, T44, T45, T46, T50, deg2, T49, deg1, Syn_IkB, Syn_XIAP, diss3, diss5, T6, Syn_Procasp3, Syn_Bid |
| 104. | 1, 15, 18, 33 | 2 Syn_BAX, Syn_SMAC, Syn_Cyt_c, Syn_Apaf1, Syn_Procasp9, Apoptosis, T65, 2 deg3, T66, T67, 2 Syn_TNFR1, 2 Syn_TNF, 2 Syn_TRADD, 2 Syn_RIP1, T69, Syn_TRAF2, Syn_cIAP, T71, Syn_TAB, Syn_TAK1, 2 Syn_NEMO, T72, 2 Syn_IKK, Syn_LUBAC, Syn_NF_kB, 2 Syn_FADD, 4 Syn_Procasp8, T75, Pc9_inhib, 2 T1, 2 T2, 2 T3, T4, T5, T14, T70, T7, T15, T8, T16, T9, T17, T13, deg5, T22, T23, T24, deg7, Syn_SCF, T25, T26, T27, T30, T31, T32, T63, T39, T40, T10, T11, T12, T18, T19, Syn_CYLD, diss1, T21, T20, deg4, T38, T48, diss2, 2 T43, 2 T44, 2 T45, 2 T46, 2 T49, 2 deg1, Syn_IkB, Syn_XIAP, 2 diss3, T6, Syn_Procasp3, Syn_Bid |
| 105. | 1, 15, 18, 32 | 2 Syn_BAX, Syn_SMAC, Syn_Cyt_c, Syn_Apaf1, Syn_Procasp9, Apoptosis, T65, 2 deg3, T66, T67, 2 Syn_TNFR1, 2 Syn_TNF, 2 Syn_TRADD, Syn_RIP1, T69, Syn_TRAF2, Syn_cIAP, T71, Syn_TAB, Syn_TAK1, 2 Syn_NEMO, T72, 2 Syn_IKK, Syn_LUBAC, Syn_NF_kB, 2 Syn_FADD, 4 Syn_Procasp8, T75, Pc9_inhib, 2 T1, 2 T2, T3, T4, T5, T14, T70, T7, T15, T8, T16, T9, T17, T13, deg5, T22, T23, T24, deg7, Syn_SCF, T25, T26, T27, T30, T31, T32, T63, T39, T40, T10, T11, T12, T18, T19, Syn_CYLD, diss1, T21, T20, deg4, T38, diss2, T42, 2 T43, 2 T44, 2 T45, 2 T46, T49, deg1, Syn_IkB, Syn_XIAP, 2 diss3, T6, Syn_Procasp3, Syn_Bid |
| 106. | 1, 15, 18, 31 | 2 Syn_BAX, Syn_SMAC, Syn_Cyt_c, Syn_Apaf1, Syn_Procasp9, Apoptosis, T65, 2 deg3, T66, T67, 2 Syn_TNFR1, 2 Syn_TNF, 2 Syn_TRADD, 2 Syn_RIP1, T69, Syn_TRAF2, Syn_cIAP, T71, Syn_TAB, Syn_TAK1, 2 Syn_NEMO, T72, 2 Syn_IKK, Syn_LUBAC, Syn_NF_kB, 2 Syn_FADD, 4 Syn_Procasp8, T75, Pc9_inhib, 2 T1, 2 T2, 2 T3, T4, T5, T14, T70, T7, T15, T8, T16, T9, T17, T13, deg5, T22, T23, T24, deg7, Syn_SCF, T25, T26, T27, T30, T31, T32, T63, T39, T40, T10, T11, T12, T18, T19, Syn_CYLD, diss1, T21, T20, deg4, T38, T48, diss2, T53, T43, T54, T61, T62, T44, T45, T46, T50, deg2, T49, deg1, Syn_IkB, Syn_XIAP, diss3, diss5, T6, Syn_Procasp3, Syn_Bid |
| 107. | 1, 15, 18, 28 | 2 Syn_BAX, Syn_SMAC, Syn_Cyt_c, Syn_Apaf1, 2 Syn_Procasp9, Apoptosis, T65, 2 deg3, T66, T67, 2 Syn_TNFR1, 2 Syn_TNF, 2 Syn_TRADD, 2 Syn_RIP1, T69, Syn_TRAF2, Syn_cIAP, T71, Syn_TAB, Syn_TAK1, 2 Syn_NEMO, T72, 2 Syn_IKK, Syn_LUBAC, T73, Syn_NF_kB, 2 Syn_FADD, 4 Syn_Procasp8, 2 T1, 2 T2, 2 T3, T4, T5, diss6, T14, T64, CASP3_inhib, T70, T7, T15, T8, T16, T9, T17, T13, deg5, T22, T23, T24, deg7, Syn_SCF, T25, T26, T27, T30, T31, T32, deg6, T63, T74, T39, T40, T10, T11, T12, T18, T19, Syn_CYLD, diss1, T21, T20, deg4, T38, T48, diss2, 2 T43, 2 T44, 2 T45, 2 T46, 2 T49, 2 deg1, Syn_IkB, Syn_XIAP, 2 diss3, T6, 2 Syn_Procasp3, Syn_Bid |
| 108. | 1, 15, 18, 27 | 2 Syn_BAX, Syn_SMAC, Syn_Cyt_c, Syn_Apaf1, 2 Syn_Procasp9, Apoptosis, T65, 2 deg3, T66, T67, 2 Syn_TNFR1, 2 Syn_TNF, 2 Syn_TRADD, Syn_RIP1, T69, Syn_TRAF2, Syn_cIAP, T71, Syn_TAB, Syn_TAK1, 2 Syn_NEMO, T72, 2 Syn_IKK, Syn_LUBAC, T73, Syn_NF_kB, 2 Syn_FADD, 4 Syn_Procasp8, 2 T1, 2 T2, T3, T4, T5, diss6, T14, T64, CASP3_inhib, T70, T7, T15, T8, T16, T9, T17, T13, deg5, T22, T23, T24, deg7, Syn_SCF, T25, T26, T27, T30, T31, T32, deg6, T63, T74, T39, T40, T10, T11, T12, T18, T19, Syn_CYLD, diss1, T21, T20, deg4, T38, diss2, T42, 2 T43, 2 T44, 2 T45, 2 T46, T49, deg1, Syn_IkB, Syn_XIAP, 2 diss3, T6, 2 Syn_Procasp3, Syn_Bid |
| 109. | 1, 15, 18, 26 | 2 Syn_BAX, Syn_SMAC, Syn_Cyt_c, Syn_Apaf1, 2 Syn_Procasp9, Apoptosis, T65, 2 deg3, T66, T67, 2 Syn_TNFR1, 2 Syn_TNF, 2 Syn_TRADD, 2 Syn_RIP1, T69, Syn_TRAF2, Syn_cIAP, T71, Syn_TAB, Syn_TAK1, 2 Syn_NEMO, T72, 2 Syn_IKK, Syn_LUBAC, T73, Syn_NF_kB, 2 Syn_FADD, 4 Syn_Procasp8, 2 T1, 2 T2, 2 T3, T4, T5, diss6, T14, T64, CASP3_inhib, T70, T7, T15, T8, T16, T9, T17, T13, deg5, T22, T23, T24, deg7, Syn_SCF, T25, T26, T27, T30, T31, T32, deg6, T63, T74, T39, T40, T10, T11, T12, T18, T19, Syn_CYLD, diss1, T21, T20, deg4, T38, T48, diss2, T53, T43, T54, T61, T62, T44, T45, T46, T50, deg2, T49, deg1, Syn_IkB, Syn_XIAP, diss3, diss5, T6, 2 Syn_Procasp3, Syn_Bid |
| 110. | 1, 15, 18, 19 | Apoptosis, deg3, 2 Syn_TNFR1, 2 Syn_TNF, 2 Syn_TRADD, 2 Syn_RIP1, Syn_TRAF2, Syn_cIAP, Syn_TAB, Syn_TAK1, 2 Syn_NEMO, 2 Syn_IKK, Syn_LUBAC, Syn_NF_kB, 2 Syn_FADD, 3 Syn_Procasp8, Syn_RIP3, 2 T1, 2 T2, 2 T3, T4, T5, T14, T60, RIP1_RIP3_inhib, T7, T15, T8, T16, T9, T17, T13, T22, T23, T24, deg7, Syn_SCF, T25, T26, T27, T30, T33, T34, T63, T39, T40, T10, T11, T12, T18, T19, Syn_CYLD, diss1, T21, T20, deg4, T38, T48, diss2, T53, T43, T54, T59, Pc8_inhib, T44, T45, T46, T50, deg2, T49, deg1, Syn_IkB, Syn_cFLIPL, diss3, diss5, T6, Syn_Procasp |
| 111. | 15, 18, 25, 37 | 2 Syn_BAX, Syn_SMAC, Syn_Cyt_c, Syn_Apaf1, Syn_Procasp9, T65, deg3, T66, T67, 2 Syn_TNFR1, 2 Syn_TNF, 2 Syn_TRADD, Syn_RIP1, T69, Syn_TRAF2, Syn_cIAP, T71, Syn_TAB, Syn_TAK1, 2 Syn_NEMO, T72, 2 Syn_IKK, Syn_LUBAC, Syn_NF_kB, Syn_FADD, T76, 2 Syn_Procasp8, Apo_XIAP_inhib, Syn_RIP3, T75, Syn_MLKL, 2 T1, 2 T2, T3, T4, T5, T14, T58, T70, T51, T7, T15, T52, T8, T16, T9, T17, T13, T22, T23, T24, deg7, Syn_SCF, T25, T26, T27, T30, T31, T32, diss4, T39, T40, T10, T11, T12, T18, T19, Syn_CYLD, diss1, T21, T20, deg4, T38, diss2, T42, T43, T44, T45, Necroptosis, T46, T50, deg2, Syn_IkB, Syn_XIAP, diss3, T6, Syn_Bid |
| 112. | 2, 12, 15, 18 | Apoptosis, 2 deg3, 2 Syn_TNFR1, 2 Syn_TNF, 2 Syn_TRADD, Syn_RIP1, Syn_TRAF2, Syn_cIAP, Syn_TAB, Syn_TAK1, 2 Syn_NEMO, 2 Syn_IKK, Syn_LUBAC, Syn_NF_kB, 2 Syn_FADD, 4 Syn_Procasp8, 2 T1, 2 T2, T3, T4, T5, T14, T64, CASP3_inhib, T7, T15, T8, T16, T9, T17, T13, T22, T23, T24, deg7, Syn_SCF, T25, T26, T27, T30, T31, T32, 2 T63, T39, T40, T10, T11, T12, T18, T19, Syn_CYLD, diss1, T21, T20, deg4, T38, diss2, T53, T42, T43, T54, T61, T62, T44, T45, T46, T50, deg2, Syn_IkB, Syn_XIAP, diss3, diss5, T6, 2 Syn_Procasp3 |
| 113. | 2, 14, 15, 18 | Apoptosis, 2 deg3, 2 Syn_TNFR1, 2 Syn_TNF, 2 Syn_TRADD, 2 Syn_RIP1, Syn_TRAF2, Syn_cIAP, Syn_TAB, Syn_TAK1, 2 Syn_NEMO, 2 Syn_IKK, Syn_LUBAC, Syn_NF_kB, 2 Syn_FADD, 4 Syn_Procasp8, 2 T1, 2 T2, 2 T3, T4, T5, T14, T64, CASP3_inhib, T7, T15, T8, T16, T9, T17, T13, T22, T23, T24, deg7, Syn_SCF, T25, T26, T27, T30, T31, T32, 2 T63, T39, T40, T10, T11, T12, T18, T19, Syn_CYLD, diss1, T21, T20, deg4, T38, T48, diss2, 2 T53, 2 T54, 2 T61, 2 T62, 2 T50, 2 deg2, Syn_IkB, Syn_XIAP, 2 diss5, T6, 2 Syn_Procasp3 |
| 114. | 2, 15, 16, 18 | Apoptosis, deg3, Syn_TNFR1, Syn_TNF, Syn_TRADD, Syn_RIP1, Syn_TRAF2, Syn_cIAP, Syn_TAB, Syn_TAK1, 2 Syn_NEMO, 2 Syn_IKK, Syn_LUBAC, Syn_NF_kB, Syn_FADD, 2 Syn_Procasp8, T1, T2, T3, T4, T5, T14, T7, T15, T8, T16, T9, T17, T13, 2 T22, T37, 2 T23, 2 T24, 2 deg7, 2 Syn_SCF, T25, 2 T26, 2 T27, T30, T63, T39, T40, T10, T11, T12, T18, T19, Syn_CYLD, diss1, T21, T20, deg4, T38, T53, T54, T61, T62, T50, deg2, 2 Syn_IkB, diss5, T6, Syn_Procasp3 |
| 115. | 2, 15, 17, 18 | Apoptosis, deg3, 2 Syn_TNFR1, 2 Syn_TNF, 2 Syn_TRADD, 2 Syn_RIP1, Syn_TRAF2, Syn_cIAP, Syn_TAB, Syn_TAK1, 2 Syn_NEMO, 2 Syn_IKK, Syn_LUBAC, Syn_NF_kB, 2 Syn_FADD, 3 Syn_Procasp8, 2 T1, 2 T2, 2 T3, T4, T5, T14, T7, T15, T8, T16, T9, T17, T13, T22, T23, T24, deg7, Syn_SCF, T25, T26, T27, T30, T33, T34, T63, T39, T40, T10, T11, T12, T18, T19, Syn_CYLD, diss1, T21, T20, deg4, T38, T48, diss2, T53, T43, T54, T61, T62, T44, T47, CIIa_inhib, T50, deg2, T49, deg1, Syn_IkB, Syn_cFLIPL, diss3, diss5, T6, Syn_Procasp3 |
| 116. | 15, 18, 25, 38 | 2 Syn_BAX, Syn_SMAC, Syn_Cyt_c, Syn_Apaf1, Syn_Procasp9, T65, deg3, T66, T67, 2 Syn_TNFR1, 2 Syn_TNF, 2 Syn_TRADD, 2 Syn_RIP1, T69, Syn_TRAF2, Syn_cIAP, T71, Syn_TAB, Syn_TAK1, 2 Syn_NEMO, T72, 2 Syn_IKK, Syn_LUBAC, Syn_NF_kB, Syn_FADD, T76, 2 Syn_Procasp8, Apo_XIAP_inhib, Syn_RIP3, T75, Syn_MLKL, 2 T1, 2 T2, 2 T3, T4, T5, T14, T58, T70, T51, T7, T15, T52, T8, T16, T9, T17, T13, T22, T23, T24, deg7, Syn_SCF, T25, T26, T27, T30, T31, T32, diss4, T39, T40, T10, T11, T12, T18, T19, Syn_CYLD, diss1, T21, T20, deg4, T38, T48, diss2, T43, T44, T45, Necroptosis, T46, T50, deg2, T49, deg1, Syn_IkB, Syn_XIAP, diss3, T6, Syn_Bid |
| 117. | 2, 15, 18, 42 | Syn_BAX, Apoptosis, T65, 2 deg3, T66, T67, T68, 2 Syn_TNFR1, 2 Syn_TNF, BAX_inhib, 2 Syn_TRADD, Syn_RIP1, Syn_TRAF2, Syn_cIAP, Syn_TAB, Syn_TAK1, 2 Syn_NEMO, 2 Syn_IKK, Syn_LUBAC, Syn_NF_kB, 2 Syn_FADD, 4 Syn_Procasp8, 2 T1, 2 T2, T3, T4, T5, T14, T7, T15, T8, T16, T9, T17, T13, T22, T23, T24, deg7, Syn_SCF, T25, T26, T27, T30, T35, T36, T63, T39, T40, T10, T11, T12, T18, T19, Syn_CYLD, diss1, T21, T20, deg4, T38, diss2, T53, T42, T43, T54, T61, T62, T44, T45, T46, T50, deg2, Syn_IkB, Syn_BCL_2, diss3, diss5, T6, Syn_Procasp3, Syn_Bid |
| 118. | 2, 15, 18, 41 | Syn_BAX, Apoptosis, T65, 2 deg3, T66, T67, T68, 2 Syn_TNFR1, 2 Syn_TNF, BAX_inhib, 2 Syn_TRADD, 2 Syn_RIP1, Syn_TRAF2, Syn_cIAP, Syn_TAB, Syn_TAK1, 2 Syn_NEMO, 2 Syn_IKK, Syn_LUBAC, Syn_NF_kB, 2 Syn_FADD, 4 Syn_Procasp8, 2 T1, 2 T2, 2 T3, T4, T5, T14, T7, T15, T8, T16, T9, T17, T13, T22, T23, T24, deg7, Syn_SCF, T25, T26, T27, T30, T35, T36, T63, T39, T40, T10, T11, T12, T18, T19, Syn_CYLD, diss1, T21, T20, deg4, T38, T48, diss2, 2 T53, 2 T54, 2 T61, 2 T62, 2 T50, 2 deg2, Syn_IkB, Syn_BCL_2, 2 diss5, T6, Syn_Procasp3, Syn_Bid |
| 119. | 14, 15, 18, 45 | Syn_BAX, T65, 2 deg3, T66, T67, T68, 2 Syn_TNFR1, 2 Syn_TNF, BAX_inhib, 2 Syn_TRADD, 2 Syn_RIP1, Syn_TRAF2, Syn_cIAP, Syn_TAB, Syn_TAK1, 2 Syn_NEMO, 2 Syn_IKK, Syn_LUBAC, Syn_NF_kB, 2 Syn_FADD, 4 Syn_Procasp8, 2 T1, 2 T2, 2 T3, T4, T5, T14, T64, CASP3_inhib, T7, T15, T8, T16, T9, T17, T13, T22, T23, T24, deg7, Syn_SCF, T25, T26, T27, T30, T31, T35, T32, T36, T63, T39, T40, T10, T11, T12, T18, T19, Syn_CYLD, diss1, T21, T20, deg4, T38, T48, diss2, T53, T43, T54, T61, T62, T44, T45, T46, T50, deg2, T49, deg1, Syn_IkB, Syn_XIAP, Syn_BCL_2, diss3, diss5, T6, Syn_Procasp3, Syn_Bid |
| 120. | 2, 15, 18, 37 | 2 Syn_BAX, Syn_SMAC, Syn_Cyt_c, Syn_Apaf1, Syn_Procasp9, Apoptosis, T65, 2 deg3, T66, T67, 2 Syn_TNFR1, 2 Syn_TNF, 2 Syn_TRADD, Syn_RIP1, T69, Syn_TRAF2, Syn_cIAP, T71, Syn_TAB, Syn_TAK1, 2 Syn_NEMO, T72, 2 Syn_IKK, Syn_LUBAC, Syn_NF_kB, 2 Syn_FADD, T76, 4 Syn_Procasp8, Apo_XIAP_inhib, T75, 2 T1, 2 T2, T3, T4, T5, T14, T70, T7, T15, T8, T16, T9, T17, T13, T22, T23, T24, deg7, Syn_SCF, T25, T26, T27, T30, T31, T32, T63, T39, T40, T10, T11, T12, T18, T19, Syn_CYLD, diss1, T21, T20, deg4, T38, diss2, T53, T42, T43, T54, T61, T62, T44, T45, T46, T50, deg2, Syn_IkB, Syn_XIAP, diss3, diss5, T6, Syn_Procasp3, Syn_Bid |
| 121. | 2, 4, 15, 18 | Apoptosis, deg3, 2 Syn_TNFR1, 2 Syn_TNF, 2 Syn_TRADD, Syn_RIP1, Syn_TRAF2, Syn_cIAP, Syn_TAB, Syn_TAK1, 2 Syn_NEMO, 2 Syn_IKK, Syn_LUBAC, Syn_NF_kB, 2 Syn_FADD, 3 Syn_Procasp8, 2 T1, 2 T2, T3, T4, T5, T14, T7, T15, T8, T16, T9, T17, T13, T22, T23, T24, deg7, Syn_SCF, T25, T26, T27, T30, T33, T34, T63, T39, T40, T10, T11, T12, T18, T19, Syn_CYLD, diss1, T21, T20, deg4, T38, diss2, T53, T42, T43, T54, T61, T62, T44, T47, CIIa_inhib, T50, deg2, Syn_IkB, Syn_cFLIPL, diss3, diss5, T6, Syn_Procasp3 |
| 122. | 2, 15, 18, 36 | 2 Syn_BAX, Syn_SMAC, Syn_Cyt_c, Syn_Apaf1, Syn_Procasp9, Apoptosis, T65, 2 deg3, T66, T67, 2 Syn_TNFR1, 2 Syn_TNF, 2 Syn_TRADD, 2 Syn_RIP1, T69, Syn_TRAF2, Syn_cIAP, T71, Syn_TAB, Syn_TAK1, 2 Syn_NEMO, T72, 2 Syn_IKK, Syn_LUBAC, Syn_NF_kB, 2 Syn_FADD, T76, 4 Syn_Procasp8, Apo_XIAP_inhib, T75, 2 T1, 2 T2, 2 T3, T4, T5, T14, T70, T7, T15, T8, T16, T9, T17, T13, T22, T23, T24, deg7, Syn_SCF, T25, T26, T27, T30, T31, T32, T63, T39, T40, T10, T11, T12, T18, T19, Syn_CYLD, diss1, T21, T20, deg4, T38, T48, diss2, 2 T53, 2 T54, 2 T61, 2 T62, 2 T50, 2 deg2, Syn_IkB, Syn_XIAP, 2 diss5, T6, Syn_Procasp3, Syn_Bid |
| 123. | 14, 15, 18, 44 | Syn_BAX, T65, 2 deg3, T66, T67, T68, 2 Syn_TNFR1, 2 Syn_TNF, BAX_inhib, 2 Syn_TRADD, 2 Syn_RIP1, Syn_TRAF2, Syn_cIAP, Syn_TAB, Syn_TAK1, 2 Syn_NEMO, 2 Syn_IKK, Syn_LUBAC, Syn_NF_kB, 2 Syn_FADD, 4 Syn_Procasp8, 2 T1, 2 T2, 2 T3, T4, T5, T14, T64, CASP3_inhib, T7, T15, T8, T16, T9, T17, T13, T22, T23, T24, deg7, Syn_SCF, T25, T26, T27, T30, T31, T35, T32, T36, T63, T39, T40, T10, T11, T12, T18, T19, Syn_CYLD, diss1, T21, T20, deg4, T38, T48, diss2, 2 T53, 2 T54, 2 T61, 2 T62, 2 T50, 2 deg2, Syn_IkB, Syn_XIAP, Syn_BCL_2, 2 diss5, T6, Syn_Procasp3, Syn_Bid |
| 124. | 2, 15, 18, 32 | 2 Syn_BAX, Syn_SMAC, Syn_Cyt_c, Syn_Apaf1, Syn_Procasp9, Apoptosis, T65, 2 deg3, T66, T67, 2 Syn_TNFR1, 2 Syn_TNF, 2 Syn_TRADD, Syn_RIP1, T69, Syn_TRAF2, Syn_cIAP, T71, Syn_TAB, Syn_TAK1, 2 Syn_NEMO, T72, 2 Syn_IKK, Syn_LUBAC, Syn_NF_kB, 2 Syn_FADD, 4 Syn_Procasp8, T75, Pc9_inhib, 2 T1, 2 T2, T3, T4, T5, T14, T70, T7, T15, T8, T16, T9, T17, T13, deg5, T22, T23, T24, deg7, Syn_SCF, T25, T26, T27, T30, T31, T32, T63, T39, T40, T10, T11, T12, T18, T19, Syn_CYLD, diss1, T21, T20, deg4, T38, diss2, T53, T42, T43, T54, T61, T62, T44, T45, T46, T50, deg2, Syn_IkB, Syn_XIAP, diss3, diss5, T6, Syn_Procasp3, Syn_Bid |
| 125. | 2, 15, 18, 31 | 2 Syn_BAX, Syn_SMAC, Syn_Cyt_c, Syn_Apaf1, Syn_Procasp9, Apoptosis, T65, 2 deg3, T66, T67, 2 Syn_TNFR1, 2 Syn_TNF, 2 Syn_TRADD, 2 Syn_RIP1, T69, Syn_TRAF2, Syn_cIAP, T71, Syn_TAB, Syn_TAK1, 2 Syn_NEMO, T72, 2 Syn_IKK, Syn_LUBAC, Syn_NF_kB, 2 Syn_FADD, 4 Syn_Procasp8, T75, Pc9_inhib, 2 T1, 2 T2, 2 T3, T4, T5, T14, T70, T7, T15, T8, T16, T9, T17, T13, deg5, T22, T23, T24, deg7, Syn_SCF, T25, T26, T27, T30, T31, T32, T63, T39, T40, T10, T11, T12, T18, T19, Syn_CYLD, diss1, T21, T20, deg4, T38, T48, diss2, 2 T53, 2 T54, 2 T61, 2 T62, 2 T50, 2 deg2, Syn_IkB, Syn_XIAP, 2 diss5, T6, Syn_Procasp3, Syn_Bid |
| 126. | 15, 18, 25, 41 | Syn_BAX, T65, deg3, T66, T67, T68, 2 Syn_TNFR1, 2 Syn_TNF, BAX_inhib, 2 Syn_TRADD, 2 Syn_RIP1, Syn_TRAF2, Syn_cIAP, Syn_TAB, Syn_TAK1, 2 Syn_NEMO, 2 Syn_IKK, Syn_LUBAC, Syn_NF_kB, Syn_FADD, 2 Syn_Procasp8, Syn_RIP3, Syn_MLKL, 2 T1, 2 T2, 2 T3, T4, T5, T14, T58, T51, T7, T15, T52, T8, T16, T9, T17, T13, T22, T23, T24, deg7, Syn_SCF, T25, T26, T27, T30, T35, T36, diss4, T39, T40, T10, T11, T12, T18, T19, Syn_CYLD, diss1, T21, T20, deg4, T38, T48, diss2, T53, T54, T61, T62, Necroptosis, 2 T50, 2 deg2, Syn_IkB, Syn_BCL_2, diss5, T6, Syn_Bid |
| 127. | 2, 15, 18, 27 | 2 Syn_BAX, Syn_SMAC, Syn_Cyt_c, Syn_Apaf1, 2 Syn_Procasp9, Apoptosis, T65, 2 deg3, T66, T67, 2 Syn_TNFR1, 2 Syn_TNF, 2 Syn_TRADD, Syn_RIP1, T69, Syn_TRAF2, Syn_cIAP, T71, Syn_TAB, Syn_TAK1, 2 Syn_NEMO, T72, 2 Syn_IKK, Syn_LUBAC, T73, Syn_NF_kB, 2 Syn_FADD, 4 Syn_Procasp8, 2 T1, 2 T2, T3, T4, T5, diss6, T14, T64, CASP3_inhib, T70, T7, T15, T8, T16, T9, T17, T13, deg5, T22, T23, T24, deg7, Syn_SCF, T25, T26, T27, T30, T31, T32, deg6, T63, T74, T39, T40, T10, T11, T12, T18, T19, Syn_CYLD, diss1, T21, T20, deg4, T38, diss2, T53, T42, T43, T54, T61, T62, T44, T45, T46, T50, deg2, Syn_IkB, Syn_XIAP, diss3, diss5, T6, 2 Syn_Procasp3, Syn_Bid |
| 128. | 2, 15, 18, 26 | 2 Syn_BAX, Syn_SMAC, Syn_Cyt_c, Syn_Apaf1, 2 Syn_Procasp9, Apoptosis, T65, 2 deg3, T66, T67, 2 Syn_TNFR1, 2 Syn_TNF, 2 Syn_TRADD, 2 Syn_RIP1, T69, Syn_TRAF2, Syn_cIAP, T71, Syn_TAB, Syn_TAK1, 2 Syn_NEMO, T72, 2 Syn_IKK, Syn_LUBAC, T73, Syn_NF_kB, 2 Syn_FADD, 4 Syn_Procasp8, 2 T1, 2 T2, 2 T3, T4, T5, diss6, T14, T64, CASP3_inhib, T70, T7, T15, T8, T16, T9, T17, T13, deg5, T22, T23, T24, deg7, Syn_SCF, T25, T26, T27, T30, T31, T32, deg6, T63, T74, T39, T40, T10, T11, T12, T18, T19, Syn_CYLD, diss1, T21, T20, deg4, T38, T48, diss2, 2 T53, 2 T54, 2 T61, 2 T62, 2 T50, 2 deg2, Syn_IkB, Syn_XIAP, 2 diss5, T6, 2 Syn_Procasp3, Syn_Bid |
| 129. | 2, 15, 18, 19 | Apoptosis, deg3, 2 Syn_TNFR1, 2 Syn_TNF, 2 Syn_TRADD, 2 Syn_RIP1, Syn_TRAF2, Syn_cIAP, Syn_TAB, Syn_TAK1, 2 Syn_NEMO, 2 Syn_IKK, Syn_LUBAC, Syn_NF_kB, 2 Syn_FADD, 3 Syn_Procasp8, Syn_RIP3, 2 T1, 2 T2, 2 T3, T4, T5, T14, T60, RIP1_RIP3_inhib, T7, T15, T8, T16, T9, T17, T13, T22, T23, T24, deg7, Syn_SCF, T25, T26, T27, T30, T33, T34, T63, T39, T40, T10, T11, T12, T18, T19, Syn_CYLD, diss1, T21, T20, deg4, T38, T48, diss2, 2 T53, 2 T54, T61, T62, T59, Pc8_inhib, 2 T50, 2 deg2, Syn_IkB, Syn_cFLIPL, 2 diss5, T6, Syn_Procasp3 |
| 130. | 15, 18, 27, 40 | 4 Syn_BAX, 2 Syn_SMAC, 2 Syn_Cyt_c, 2 Syn_Apaf1, 3 Syn_Procasp9, 2 T65, 2 deg3, 2 T66, 2 T67, 2 Syn_TNFR1, 2 Syn_TNF, 2 Syn_TRADD, Syn_RIP1, 2 T69, Syn_TRAF2, Syn_cIAP, 2 T71, Syn_TAB, Syn_TAK1, 2 Syn_NEMO, 2 T72, 2 Syn_IKK, Syn_LUBAC, T73, Syn_NF_kB, 2 Syn_FADD, T76, 4 Syn_Procasp8, Apo_XIAP_inhib, T75, 2 T1, 2 T2, T3, T4, T5, diss6, T14, T64, CASP3_inhib, 2 T70, T7, T15, T8, T16, T9, T17, T13, deg5, T22, T23, T24, deg7, Syn_SCF, T25, T26, T27, T30, 2 T31, 2 T32, deg6, T74, T39, T40, T10, T11, T12, T18, T19, Syn_CYLD, diss1, T21, T20, deg4, T38, diss2, T42, 2 T43, 2 T44, 2 T45, 2 T46, T49, deg1, Syn_IkB, 2 Syn_XIAP, 2 diss3, T6, Syn_Procasp3, 2 Syn_Bid |
| 131. | 4, 9, 15, 18 | 2 Syn_TNFR1, 2 Syn_TNF, 2 Syn_TRADD, Syn_RIP1, Syn_TRAF2, Syn_cIAP, Syn_TAB, Syn_TAK1, 2 Syn_NEMO, 2 Syn_IKK, Syn_LUBAC, Syn_NF_kB, Syn_FADD, Syn_Procasp8, 2 T1, 2 T2, T3, T4, T5, T14, T7, T15, T8, T16, T9, T17, T13, T22, T23, T24, deg7, Syn_SCF, T25, T26, T28, T29_, T27, T30, T33, T34, T39, T40, T41, CI_diss, T10, T11, T12, deg4, T38, diss2, T42, T43, T44, T47, CIIa_inhib, Syn_IkB, Syn_A20, Syn_cFLIPL, diss3, T6 |
| 132. | 15, 18, 27, 39 | 4 Syn_BAX, 2 Syn_SMAC, 2 Syn_Cyt_c, 2 Syn_Apaf1, 3 Syn_Procasp9, 2 T65, 2 deg3, 2 T66, 2 T67, 2 Syn_TNFR1, 2 Syn_TNF, 2 Syn_TRADD, Syn_RIP1, 2 T69, Syn_TRAF2, Syn_cIAP, 2 T71, Syn_TAB, Syn_TAK1, 2 Syn_NEMO, 2 T72, 2 Syn_IKK, Syn_LUBAC, T73, Syn_NF_kB, 2 Syn_FADD, T76, 4 Syn_Procasp8, Apo_XIAP_inhib, T75, 2 T1, 2 T2, T3, T4, T5, diss6, T14, T64, CASP3_inhib, 2 T70, T7, T15, T8, T16, T9, T17, T13, deg5, T22, T23, T24, deg7, Syn_SCF, T25, T26, T27, T30, 2 T31, 2 T32, deg6, T74, T39, T40, T10, T11, T12, T18, T19, Syn_CYLD, diss1, T21, T20, deg4, T38, diss2, T53, T42, T43, T54, T61, T62, T44, T45, T46, T50, deg2, Syn_IkB, 2 Syn_XIAP, diss3, diss5, T6, Syn_Procasp3, 2 Syn_Bid |
| 133. | 15, 18, 28, 29 | 4 Syn_BAX, 2 Syn_SMAC, 2 Syn_Cyt_c, 2 Syn_Apaf1, 4 Syn_Procasp9, 2 T65, 2 deg3, 2 T66, 2 T67, 2 Syn_TNFR1, 2 Syn_TNF, 2 Syn_TRADD, 2 Syn_RIP1, 2 T69, Syn_TRAF2, Syn_cIAP, 2 T71, Syn_TAB, Syn_TAK1, 2 Syn_NEMO, 2 T72, 2 Syn_IKK, Syn_LUBAC, 2 T73, Syn_NF_kB, 2 Syn_FADD, 4 Syn_Procasp8, 2 T1, 2 T2, 2 T3, T4, T5, 2 diss6, T14, 2 T64, 2 CASP3_inhib, 2 T70, T7, T15, T8, T16, T9, T17, T13, 2 deg5, T22, T23, T24, deg7, Syn_SCF, T25, T26, T27, T30, 2 T31, 2 T32, 2 deg6, 2 T74, T39, T40, T10, T11, T12, T18, T19, Syn_CYLD, diss1, T21, T20, deg4, T38, T48, diss2, T53, T43, T54, T61, T62, T44, T45, T46, T50, deg2, T49, deg1, Syn_IkB, 2 Syn_XIAP, diss3, diss5, T6, 2 Syn_Procasp3, 2 Syn_Bid |
| 134. | 4, 8, 15, 18 | deg3, 2 Syn_TNFR1, 2 Syn_TNF, 2 Syn_TRADD, Syn_RIP1, Syn_TRAF2, Syn_cIAP, Syn_TAB, Syn_TAK1, 2 Syn_NEMO, 2 Syn_IKK, Syn_LUBAC, Syn_NF_kB, 2 Syn_FADD, 3 Syn_Procasp8, 2 T1, 2 T2, T3, T4, T5, T14, T64, CASP3_inhib, T7, T15, T8, T16, T9, T17, T13, T22, T23, T24, deg7, Syn_SCF, T25, T26, T27, T30, T31, T33, T32, T34, T63, T39, T40, T10, T11, T12, T18, T19, Syn_CYLD, diss1, T21, T20, deg4, T38, diss2, T53, T42, T43, T54, T61, T62, T44, T47, CIIa_inhib, T50, deg2, Syn_IkB, Syn_XIAP, Syn_cFLIPL, diss3, diss5, T6, Syn_Procasp3 |
| 135. | 15, 18, 28, 30 | 4 Syn_BAX, 2 Syn_SMAC, 2 Syn_Cyt_c, 2 Syn_Apaf1, 4 Syn_Procasp9, 2 T65, 2 deg3, 2 T66, 2 T67, 2 Syn_TNFR1, 2 Syn_TNF, 2 Syn_TRADD, 2 Syn_RIP1, 2 T69, Syn_TRAF2, Syn_cIAP, 2 T71, Syn_TAB, Syn_TAK1, 2 Syn_NEMO, 2 T72, 2 Syn_IKK, Syn_LUBAC, 2 T73, Syn_NF_kB, 2 Syn_FADD, 4 Syn_Procasp8, 2 T1, 2 T2, 2 T3, T4, T5, 2 diss6, T14, 2 T64, 2 CASP3_inhib, 2 T70, T7, T15, T8, T16, T9, T17, T13, 2 deg5, T22, T23, T24, deg7, Syn_SCF, T25, T26, T27, T30, 2 T31, 2 T32, 2 deg6, 2 T74, T39, T40, T10, T11, T12, T18, T19, Syn_CYLD, diss1, T21, T20, deg4, T38, T48, diss2, 2 T43, 2 T44, 2 T45, 2 T46, 2 T49, 2 deg1, Syn_IkB, 2 Syn_XIAP, 2 diss3, T6, 2 Syn_Procasp3, 2 Syn_Bid |
| 136. | 15, 18, 28, 34 | 4 Syn_BAX, 2 Syn_SMAC, 2 Syn_Cyt_c, 2 Syn_Apaf1, 3 Syn_Procasp9, 2 T65, 2 deg3, 2 T66, 2 T67, 2 Syn_TNFR1, 2 Syn_TNF, 2 Syn_TRADD, 2 Syn_RIP1, 2 T69, Syn_TRAF2, Syn_cIAP, 2 T71, Syn_TAB, Syn_TAK1, 2 Syn_NEMO, 2 T72, 2 Syn_IKK, Syn_LUBAC, T73, Syn_NF_kB, 2 Syn_FADD, 4 Syn_Procasp8, T75, Pc9_inhib, 2 T1, 2 T2, 2 T3, T4, T5, diss6, T14, T64, CASP3_inhib, 2 T70, T7, T15, T8, T16, T9, T17, T13, 2 deg5, T22, T23, T24, deg7, Syn_SCF, T25, T26, T27, T30, 2 T31, 2 T32, deg6, T74, T39, T40, T10, T11, T12, T18, T19, Syn_CYLD, diss1, T21, T20, deg4, T38, T48, diss2, T53, T43, T54, T61, T62, T44, T45, T46, T50, deg2, T49, deg1, Syn_IkB, 2 Syn_XIAP, diss3, diss5, T6, Syn_Procasp3, 2 Syn_Bid |
| 137. | 15, 18, 28, 35 | 4 Syn_BAX, 2 Syn_SMAC, 2 Syn_Cyt_c, 2 Syn_Apaf1, 3 Syn_Procasp9, 2 T65, 2 deg3, 2 T66, 2 T67, 2 Syn_TNFR1, 2 Syn_TNF, 2 Syn_TRADD, 2 Syn_RIP1, 2 T69, Syn_TRAF2, Syn_cIAP, 2 T71, Syn_TAB, Syn_TAK1, 2 Syn_NEMO, 2 T72, 2 Syn_IKK, Syn_LUBAC, T73, Syn_NF_kB, 2 Syn_FADD, 4 Syn_Procasp8, T75, Pc9_inhib, 2 T1, 2 T2, 2 T3, T4, T5, diss6, T14, T64, CASP3_inhib, 2 T70, T7, T15, T8, T16, T9, T17, T13, 2 deg5, T22, T23, T24, deg7, Syn_SCF, T25, T26, T27, T30, 2 T31, 2 T32, deg6, T74, T39, T40, T10, T11, T12, T18, T19, Syn_CYLD, diss1, T21, T20, deg4, T38, T48, diss2, 2 T43, 2 T44, 2 T45, 2 T46, 2 T49, 2 deg1, Syn_IkB, 2 Syn_XIAP, 2 diss3, T6, Syn_Procasp3, 2 Syn_Bid |
| 138. | 12, 15, 18, 35 | 2 Syn_BAX, Syn_SMAC, Syn_Cyt_c, Syn_Apaf1, Syn_Procasp9, T65, 2 deg3, T66, T67, 2 Syn_TNFR1, 2 Syn_TNF, 2 Syn_TRADD, Syn_RIP1, T69, Syn_TRAF2, Syn_cIAP, T71, Syn_TAB, Syn_TAK1, 2 Syn_NEMO, T72, 2 Syn_IKK, Syn_LUBAC, Syn_NF_kB, 2 Syn_FADD, 4 Syn_Procasp8, T75, Pc9_inhib, 2 T1, 2 T2, T3, T4, T5, T14, T64, CASP3_inhib, T70, T7, T15, T8, T16, T9, T17, T13, deg5, T22, T23, T24, deg7, Syn_SCF, T25, T26, T27, T30, 2 T31, 2 T32, T63, T39, T40, T10, T11, T12, T18, T19, Syn_CYLD, diss1, T21, T20, deg4, T38, diss2, T42, 2 T43, 2 T44, 2 T45, 2 T46, T49, deg1, Syn_IkB, 2 Syn_XIAP, 2 diss3, T6, Syn_Procasp3, Syn_Bid |
| 139. | 12, 15, 18, 34 | 2 Syn_BAX, Syn_SMAC, Syn_Cyt_c, Syn_Apaf1, Syn_Procasp9, T65, 2 deg3, T66, T67, 2 Syn_TNFR1, 2 Syn_TNF, 2 Syn_TRADD, Syn_RIP1, T69, Syn_TRAF2, Syn_cIAP, T71, Syn_TAB, Syn_TAK1, 2 Syn_NEMO, T72, 2 Syn_IKK, Syn_LUBAC, Syn_NF_kB, 2 Syn_FADD, 4 Syn_Procasp8, T75, Pc9_inhib, 2 T1, 2 T2, T3, T4, T5, T14, T64, CASP3_inhib, T70, T7, T15, T8, T16, T9, T17, T13, deg5, T22, T23, T24, deg7, Syn_SCF, T25, T26, T27, T30, 2 T31, 2 T32, T63, T39, T40, T10, T11, T12, T18, T19, Syn_CYLD, diss1, T21, T20, deg4, T38, diss2, T53, T42, T43, T54, T61, T62, T44, T45, T46, T50, deg2, Syn_IkB, 2 Syn_XIAP, diss3, diss5, T6, Syn_Procasp3, Syn_Bid |
| 140. | 12, 15, 18, 30 | 2 Syn_BAX, Syn_SMAC, Syn_Cyt_c, Syn_Apaf1, 2 Syn_Procasp9, T65, 2 deg3, T66, T67, 2 Syn_TNFR1, 2 Syn_TNF, 2 Syn_TRADD, Syn_RIP1, T69, Syn_TRAF2, Syn_cIAP, T71, Syn_TAB, Syn_TAK1, 2 Syn_NEMO, T72, 2 Syn_IKK, Syn_LUBAC, T73, Syn_NF_kB, 2 Syn_FADD, 4 Syn_Procasp8, 2 T1, 2 T2, T3, T4, T5, diss6, T14, 2 T64, 2 CASP3_inhib, T70, T7, T15, T8, T16, T9, T17, T13, deg5, T22, T23, T24, deg7, Syn_SCF, T25, T26, T27, T30, 2 T31, 2 T32, deg6, T63, T74, T39, T40, T10, T11, T12, T18, T19, Syn_CYLD, diss1, T21, T20, deg4, T38, diss2, T42, 2 T43, 2 T44, 2 T45, 2 T46, T49, deg1, Syn_IkB, 2 Syn_XIAP, 2 diss3, T6, 2 Syn_Procasp3, Syn_Bid |
| 141. | 12, 15, 18, 29 | 2 Syn_BAX, Syn_SMAC, Syn_Cyt_c, Syn_Apaf1, 2 Syn_Procasp9, T65, 2 deg3, T66, T67, 2 Syn_TNFR1, 2 Syn_TNF, 2 Syn_TRADD, Syn_RIP1, T69, Syn_TRAF2, Syn_cIAP, T71, Syn_TAB, Syn_TAK1, 2 Syn_NEMO, T72, 2 Syn_IKK, Syn_LUBAC, T73, Syn_NF_kB, 2 Syn_FADD, 4 Syn_Procasp8, 2 T1, 2 T2, T3, T4, T5, diss6, T14, 2 T64, 2 CASP3_inhib, T70, T7, T15, T8, T16, T9, T17, T13, deg5, T22, T23, T24, deg7, Syn_SCF, T25, T26, T27, T30, 2 T31, 2 T32, deg6, T63, T74, T39, T40, T10, T11, T12, T18, T19, Syn_CYLD, diss1, T21, T20, deg4, T38, diss2, T53, T42, T43, T54, T61, T62, T44, T45, T46, T50, deg2, Syn_IkB, 2 Syn_XIAP, diss3, diss5, T6, 2 Syn_Procasp3, Syn_Bid |
| 142. | 12, 15, 18, 25 | deg3, 2 Syn_TNFR1, 2 Syn_TNF, 2 Syn_TRADD, Syn_RIP1, Syn_TRAF2, Syn_cIAP, Syn_TAB, Syn_TAK1, 2 Syn_NEMO, 2 Syn_IKK, Syn_LUBAC, Syn_NF_kB, Syn_FADD, 2 Syn_Procasp8, Syn_RIP3, Syn_MLKL, 2 T1, 2 T2, T3, T4, T5, T14, T58, T64, CASP3_inhib, T51, T7, T15, T52, T8, T16, T9, T17, T13, T22, T23, T24, deg7, Syn_SCF, T25, T26, T27, T30, T31, T32, diss4, T63, T39, T40, T10, T11, T12, T18, T19, Syn_CYLD, diss1, T21, T20, deg4, T38, diss2, T42, T43, T44, T45, Necroptosis, T46, T50, deg2, Syn_IkB, Syn_XIAP, diss3, T6, Syn_Procasp3 |
| 143. | 9, 15, 18, 19 | 2 Syn_TNFR1, 2 Syn_TNF, 2 Syn_TRADD, 2 Syn_RIP1, Syn_TRAF2, Syn_cIAP, Syn_TAB, Syn_TAK1, 2 Syn_NEMO, 2 Syn_IKK, Syn_LUBAC, Syn_NF_kB, Syn_FADD, Syn_Procasp8, Syn_RIP3, 2 T1, 2 T2, 2 T3, T4, T5, T14, T60, RIP1_RIP3_inhib, T7, T15, T8, T16, T9, T17, T13, T22, T23, T24, deg7, Syn_SCF, T25, T26, T28, T29_, T27, T30, T33, T34, T39, T40, T41, CI_diss, T10, T11, T12, deg4, T38, T48, diss2, T53, T54, T59, Pc8_inhib, T50, deg2, Syn_IkB, Syn_A20, Syn_cFLIPL, diss5, T6 |
| 144. | 12, 15, 18, 23 | deg3, 2 Syn_TNFR1, 2 Syn_TNF, 2 Syn_TRADD, Syn_RIP1, Syn_TRAF2, Syn_cIAP, Syn_TAB, Syn_TAK1, 2 Syn_NEMO, 2 Syn_IKK, Syn_LUBAC, Syn_NF_kB, 2 Syn_FADD, 3 Syn_Procasp8, 2 T1, 2 T2, T3, T4, T5, T14, T64, CASP3_inhib, T7, T15, T8, T16, T9, T17, T13, T22, T23, T24, deg7, Syn_SCF, T25, T26, T27, T30, T31, T33, T32, T34, T63, T39, T40, T10, T11, T12, T18, T19, Syn_CYLD, diss1, T21, T20, deg4, T38, diss2, T42, 2 T43, 2 T44, T45, T46, T47, CIIa_inhib, T49, deg1, Syn_IkB, Syn_XIAP, Syn_cFLIPL, 2 diss3, T6, Syn_Procasp3 |
| 145. | 12, 15, 18, 22 | deg3, 2 Syn_TNFR1, 2 Syn_TNF, 2 Syn_TRADD, Syn_RIP1, Syn_TRAF2, Syn_cIAP, Syn_TAB, Syn_TAK1, 2 Syn_NEMO, 2 Syn_IKK, Syn_LUBAC, Syn_NF_kB, 2 Syn_FADD, 3 Syn_Procasp8, Syn_RIP3, 2 T1, 2 T2, T3, T4, T5, T14, T60, RIP1_RIP3_inhib, T64, CASP3_inhib, T7, T15, T8, T16, T9, T17, T13, T22, T23, T24, deg7, Syn_SCF, T25, T26, T27, T30, T31, T33, T32, T34, T63, T39, T40, T10, T11, T12, T18, T19, Syn_CYLD, diss1, T21, T20, deg4, T38, diss2, T53, T42, T43, T54, T59, Pc8_inhib, T44, T45, T46, T50, deg2, Syn_IkB, Syn_XIAP, Syn_cFLIPL, diss3, diss5, T6, Syn_Procasp3 |
| 146. | 9, 15, 18, 36 | 2 Syn_BAX, Syn_SMAC, Syn_Cyt_c, Syn_Apaf1, Syn_Procasp9, T65, deg3, T66, T67, 2 Syn_TNFR1, 2 Syn_TNF, 2 Syn_TRADD, 2 Syn_RIP1, T69, Syn_TRAF2, Syn_cIAP, T71, Syn_TAB, Syn_TAK1, 2 Syn_NEMO, T72, 2 Syn_IKK, Syn_LUBAC, Syn_NF_kB, Syn_FADD, T76, 2 Syn_Procasp8, Apo_XIAP_inhib, T75, 2 T1, 2 T2, 2 T3, T4, T5, T14, T70, T7, T15, T8, T16, T9, T17, T13, T22, T23, T24, deg7, Syn_SCF, T25, T26, T28, T29_, T27, T30, T31, T32, T39, T40, T41, CI_diss, T10, T11, T12, deg4, T38, T48, diss2, T53, T54, T61, T62, T50, deg2, Syn_IkB, Syn_A20, Syn_XIAP, diss5, T6, Syn_Bid |
| 147. | 15, 17, 18, 35 | 2 Syn_BAX, Syn_SMAC, Syn_Cyt_c, Syn_Apaf1, Syn_Procasp9, T65, deg3, T66, T67, 2 Syn_TNFR1, 2 Syn_TNF, 2 Syn_TRADD, 2 Syn_RIP1, T69, Syn_TRAF2, Syn_cIAP, T71, Syn_TAB, Syn_TAK1, 2 Syn_NEMO, T72, 2 Syn_IKK, Syn_LUBAC, Syn_NF_kB, 2 Syn_FADD, 3 Syn_Procasp8, T75, Pc9_inhib, 2 T1, 2 T2, 2 T3, T4, T5, T14, T70, T7, T15, T8, T16, T9, T17, T13, deg5, T22, T23, T24, deg7, Syn_SCF, T25, T26, T27, T30, T31, T33, T32, T34, T39, T40, T10, T11, T12, T18, T19, Syn_CYLD, diss1, T21, T20, deg4, T38, T48, diss2, 2 T43, 2 T44, T45, T46, T47, CIIa_inhib, 2 T49, 2 deg1, Syn_IkB, Syn_XIAP, Syn_cFLIPL, 2 diss3, T6, Syn_Bid |
| 148. | 15, 17, 18, 34 | 2 Syn_BAX, Syn_SMAC, Syn_Cyt_c, Syn_Apaf1, Syn_Procasp9, T65, deg3, T66, T67, 2 Syn_TNFR1, 2 Syn_TNF, 2 Syn_TRADD, 2 Syn_RIP1, T69, Syn_TRAF2, Syn_cIAP, T71, Syn_TAB, Syn_TAK1, 2 Syn_NEMO, T72, 2 Syn_IKK, Syn_LUBAC, Syn_NF_kB, 2 Syn_FADD, 3 Syn_Procasp8, T75, Pc9_inhib, 2 T1, 2 T2, 2 T3, T4, T5, T14, T70, T7, T15, T8, T16, T9, T17, T13, deg5, T22, T23, T24, deg7, Syn_SCF, T25, T26, T27, T30, T31, T33, T32, T34, T39, T40, T10, T11, T12, T18, T19, Syn_CYLD, diss1, T21, T20, deg4, T38, T48, diss2, T53, T43, T54, T61, T62, T44, T47, CIIa_inhib, T50, deg2, T49, deg1, Syn_IkB, Syn_XIAP, Syn_cFLIPL, diss3, diss5, T6, Syn_Bid |
| 149. | 15, 18, 43, 44 | 2 Syn_BAX, 2 T65, 2 deg3, 2 T66, 2 T67, 2 T68, 2 Syn_TNFR1, 2 Syn_TNF, 2 BAX_inhib, 2 Syn_TRADD, 2 Syn_RIP1, Syn_TRAF2, Syn_cIAP, Syn_TAB, Syn_TAK1, 2 Syn_NEMO, 2 Syn_IKK, Syn_LUBAC, Syn_NF_kB, 2 Syn_FADD, 4 Syn_Procasp8, 2 T1, 2 T2, 2 T3, T4, T5, T14, T7, T15, T8, T16, T9, T17, T13, T22, T23, T24, deg7, Syn_SCF, T25, T26, T27, T30, 2 T35, 2 T36, T39, T40, T10, T11, T12, T18, T19, Syn_CYLD, diss1, T21, T20, deg4, T38, T48, diss2, T53, T43, T54, T61, T62, T44, T45, T46, T50, deg2, T49, deg1, Syn_IkB, 2 Syn_BCL_2, diss3, diss5, T6, 2 Syn_Bid |
| 150. | 15, 18, 43, 45 | 2 Syn_BAX, 2 T65, 2 deg3, 2 T66, 2 T67, 2 T68, 2 Syn_TNFR1, 2 Syn_TNF, 2 BAX_inhib, 2 Syn_TRADD, 2 Syn_RIP1, Syn_TRAF2, Syn_cIAP, Syn_TAB, Syn_TAK1, 2 Syn_NEMO, 2 Syn_IKK, Syn_LUBAC, Syn_NF_kB, 2 Syn_FADD, 4 Syn_Procasp8, 2 T1, 2 T2, 2 T3, T4, T5, T14, T7, T15, T8, T16, T9, T17, T13, T22, T23, T24, deg7, Syn_SCF, T25, T26, T27, T30, 2 T35, 2 T36, T39, T40, T10, T11, T12, T18, T19, Syn_CYLD, diss1, T21, T20, deg4, T38, T48, diss2, 2 T43, 2 T44, 2 T45, 2 T46, 2 T49, 2 deg1, Syn_IkB, 2 Syn_BCL_2, 2 diss3, T6, 2 Syn_Bid |
| 151. | 15, 18, 42, 44 | 2 Syn_BAX, 2 T65, 2 deg3, 2 T66, 2 T67, 2 T68, 2 Syn_TNFR1, 2 Syn_TNF, 2 BAX_inhib, 2 Syn_TRADD, Syn_RIP1, Syn_TRAF2, Syn_cIAP, Syn_TAB, Syn_TAK1, 2 Syn_NEMO, 2 Syn_IKK, Syn_LUBAC, Syn_NF_kB, 2 Syn_FADD, 4 Syn_Procasp8, 2 T1, 2 T2, T3, T4, T5, T14, T7, T15, T8, T16, T9, T17, T13, T22, T23, T24, deg7, Syn_SCF, T25, T26, T27, T30, 2 T35, 2 T36, T39, T40, T10, T11, T12, T18, T19, Syn_CYLD, diss1, T21, T20, deg4, T38, diss2, T53, T42, T43, T54, T61, T62, T44, T45, T46, T50, deg2, Syn_IkB, 2 Syn_BCL_2, diss3, diss5, T6, 2 Syn_Bid |
| 152. | 15, 18, 42, 45 | 2 Syn_BAX, 2 T65, 2 deg3, 2 T66, 2 T67, 2 T68, 2 Syn_TNFR1, 2 Syn_TNF, 2 BAX_inhib, 2 Syn_TRADD, Syn_RIP1, Syn_TRAF2, Syn_cIAP, Syn_TAB, Syn_TAK1, 2 Syn_NEMO, 2 Syn_IKK, Syn_LUBAC, Syn_NF_kB, 2 Syn_FADD, 4 Syn_Procasp8, 2 T1, 2 T2, T3, T4, T5, T14, T7, T15, T8, T16, T9, T17, T13, T22, T23, T24, deg7, Syn_SCF, T25, T26, T27, T30, 2 T35, 2 T36, T39, T40, T10, T11, T12, T18, T19, Syn_CYLD, diss1, T21, T20, deg4, T38, diss2, T42, 2 T43, 2 T44, 2 T45, 2 T46, T49, deg1, Syn_IkB, 2 Syn_BCL_2, 2 diss3, T6, 2 Syn_Bid |
| 153. | 14, 15, 18, 39 | 2 Syn_BAX, Syn_SMAC, Syn_Cyt_c, Syn_Apaf1, Syn_Procasp9, T65, 2 deg3, T66, T67, 2 Syn_TNFR1, 2 Syn_TNF, 2 Syn_TRADD, 2 Syn_RIP1, T69, Syn_TRAF2, Syn_cIAP, T71, Syn_TAB, Syn_TAK1, 2 Syn_NEMO, T72, 2 Syn_IKK, Syn_LUBAC, Syn_NF_kB, 2 Syn_FADD, T76, 4 Syn_Procasp8, Apo_XIAP_inhib, T75, 2 T1, 2 T2, 2 T3, T4, T5, T14, T64, CASP3_inhib, T70, T7, T15, T8, T16, T9, T17, T13, T22, T23, T24, deg7, Syn_SCF, T25, T26, T27, T30, 2 T31, 2 T32, T63, T39, T40, T10, T11, T12, T18, T19, Syn_CYLD, diss1, T21, T20, deg4, T38, T48, diss2, 2 T53, 2 T54, 2 T61, 2 T62, 2 T50, 2 deg2, Syn_IkB, 2 Syn_XIAP, 2 diss5, T6, Syn_Procasp3, Syn_Bid |
| 154. | 15, 18, 41, 44 | 2 Syn_BAX, 2 T65, 2 deg3, 2 T66, 2 T67, 2 T68, 2 Syn_TNFR1, 2 Syn_TNF, 2 BAX_inhib, 2 Syn_TRADD, 2 Syn_RIP1, Syn_TRAF2, Syn_cIAP, Syn_TAB, Syn_TAK1, 2 Syn_NEMO, 2 Syn_IKK, Syn_LUBAC, Syn_NF_kB, 2 Syn_FADD, 4 Syn_Procasp8, 2 T1, 2 T2, 2 T3, T4, T5, T14, T7, T15, T8, T16, T9, T17, T13, T22, T23, T24, deg7, Syn_SCF, T25, T26, T27, T30, 2 T35, 2 T36, T39, T40, T10, T11, T12, T18, T19, Syn_CYLD, diss1, T21, T20, deg4, T38, T48, diss2, 2 T53, 2 T54, 2 T61, 2 T62, 2 T50, 2 deg2, Syn_IkB, 2 Syn_BCL_2, 2 diss5, T6, 2 Syn_Bid |
| 155. | 15, 16, 18, 45 | Syn_BAX, T65, deg3, T66, T67, T68, Syn_TNFR1, Syn_TNF, BAX_inhib, Syn_TRADD, Syn_RIP1, Syn_TRAF2, Syn_cIAP, Syn_TAB, Syn_TAK1, 2 Syn_NEMO, 2 Syn_IKK, Syn_LUBAC, Syn_NF_kB, Syn_FADD, 2 Syn_Procasp8, T1, T2, T3, T4, T5, T14, T7, T15, T8, T16, T9, T17, T13, 2 T22, T37, 2 T23, 2 T24, 2 deg7, 2 Syn_SCF, T25, 2 T26, 2 T27, T30, T35, T36, T39, T40, T10, T11, T12, T18, T19, Syn_CYLD, diss1, T21, T20, deg4, T38, T43, T44, T45, T46, T49, deg1, 2 Syn_IkB, Syn_BCL_2, diss3, T6, Syn_Bid |
| 156. | 12, 15, 18, 20 | deg3, 2 Syn_TNFR1, 2 Syn_TNF, 2 Syn_TRADD, Syn_RIP1, Syn_TRAF2, Syn_cIAP, Syn_TAB, Syn_TAK1, 2 Syn_NEMO, 2 Syn_IKK, Syn_LUBAC, Syn_NF_kB, 2 Syn_FADD, 3 Syn_Procasp8, Syn_cFLIPs, Syn_RIP3, Syn_MLKL, 2 T1, 2 T2, T3, T4, T5, T14, T58, T64, CASP3_inhib, T7, T15, T8, T16, T9, T17, T13, T22, T23, T24, deg7, Syn_SCF, T25, T26, T27, T30, T31, T32, T63, T39, T40, T10, T11, T12, T18, T19, Syn_CYLD, diss1, T21, T20, deg4, T38, diss2, T53, T42, T43, T54, T44, T45, T55, T56, T57, Necroptosis, T46, T50, deg2, Syn_IkB, Syn_XIAP, diss3, diss5, T6, Syn_Procasp3 |
| 157. | 15, 18, 28, 45 | 3 Syn_BAX, Syn_SMAC, Syn_Cyt_c, Syn_Apaf1, 2 Syn_Procasp9, 2 T65, 2 deg3, 2 T66, 2 T67, T68, 2 Syn_TNFR1, 2 Syn_TNF, BAX_inhib, 2 Syn_TRADD, 2 Syn_RIP1, T69, Syn_TRAF2, Syn_cIAP, T71, Syn_TAB, Syn_TAK1, 2 Syn_NEMO, T72, 2 Syn_IKK, Syn_LUBAC, T73, Syn_NF_kB, 2 Syn_FADD, 4 Syn_Procasp8, 2 T1, 2 T2, 2 T3, T4, T5, diss6, T14, T64, CASP3_inhib, T70, T7, T15, T8, T16, T9, T17, T13, deg5, T22, T23, T24, deg7, Syn_SCF, T25, T26, T27, T30, T31, T35, T32, T36, deg6, T74, T39, T40, T10, T11, T12, T18, T19, Syn_CYLD, diss1, T21, T20, deg4, T38, T48, diss2, 2 T43, 2 T44, 2 T45, 2 T46, 2 T49, 2 deg1, Syn_IkB, Syn_XIAP, Syn_BCL_2, 2 diss3, T6, Syn_Procasp3, 2 Syn_Bid |
| 158. | 4, 15, 18, 20 | 2 Syn_TNFR1, 2 Syn_TNF, 2 Syn_TRADD, Syn_RIP1, Syn_TRAF2, Syn_cIAP, Syn_TAB, Syn_TAK1, 2 Syn_NEMO, 2 Syn_IKK, Syn_LUBAC, Syn_NF_kB, 2 Syn_FADD, 2 Syn_Procasp8, Syn_cFLIPs, Syn_RIP3, Syn_MLKL, 2 T1, 2 T2, T3, T4, T5, T14, T58, T7, T15, T8, T16, T9, T17, T13, T22, T23, T24, deg7, Syn_SCF, T25, T26, T27, T30, T33, T34, T39, T40, T10, T11, T12, T18, T19, Syn_CYLD, diss1, T21, T20, deg4, T38, diss2, T53, T42, T43, T54, T44, T55, T56, T57, Necroptosis, T47, CIIa_inhib, T50, deg2, Syn_IkB, Syn_cFLIPL, diss3, diss5, T6 |
| 159. | 4, 15, 18, 21 | 2 Syn_BAX, Syn_SMAC, Syn_Cyt_c, Syn_Apaf1, 2 Syn_Procasp9, Apoptosis, T65, deg3, T66, T67, 2 Syn_TNFR1, 2 Syn_TNF, 2 Syn_TRADD, Syn_RIP1, T69, Syn_TRAF2, Syn_cIAP, T71, Syn_TAB, Syn_TAK1, 2 Syn_NEMO, T72, 2 Syn_IKK, Syn_LUBAC, T73, Syn_NF_kB, 2 Syn_FADD, 3 Syn_Procasp8, 2 T1, 2 T2, T3, T4, T5, diss6, T14, T70, T7, T15, T8, T16, T9, T17, T13, deg5, T22, T23, T24, deg7, Syn_SCF, T25, T26, T27, T30, T33, T34, deg6, T74, T39, T40, T10, T11, T12, T18, T19, Syn_CYLD, diss1, T21, T20, deg4, T38, diss2, T42, 2 T43, 2 T44, T45, T46, T47, CIIa_inhib, T49, deg1, Syn_IkB, Syn_cFLIPL, 2 diss3, T6, Syn_Procasp3, Syn_Bid |
| 160. | 4, 15, 18, 22 | 2 Syn_TNFR1, 2 Syn_TNF, 2 Syn_TRADD, Syn_RIP1, Syn_TRAF2, Syn_cIAP, Syn_TAB, Syn_TAK1, 2 Syn_NEMO, 2 Syn_IKK, Syn_LUBAC, Syn_NF_kB, 2 Syn_FADD, 2 Syn_Procasp8, Syn_RIP3, 2 T1, 2 T2, T3, T4, T5, T14, T60, RIP1_RIP3_inhib, T7, T15, T8, T16, T9, T17, T13, T22, T23, T24, deg7, Syn_SCF, T25, T26, T27, T30, 2 T33, 2 T34, T39, T40, T10, T11, T12, T18, T19, Syn_CYLD, diss1, T21, T20, deg4, T38, diss2, T53, T42, T43, T54, T59, Pc8_inhib, T44, T47, CIIa_inhib, T50, deg2, Syn_IkB, 2 Syn_cFLIPL, diss3, diss5, T6 |
| 161. | 4, 15, 18, 23 | 2 Syn_TNFR1, 2 Syn_TNF, 2 Syn_TRADD, Syn_RIP1, Syn_TRAF2, Syn_cIAP, Syn_TAB, Syn_TAK1, 2 Syn_NEMO, 2 Syn_IKK, Syn_LUBAC, Syn_NF_kB, 2 Syn_FADD, 2 Syn_Procasp8, 2 T1, 2 T2, T3, T4, T5, T14, T7, T15, T8, T16, T9, T17, T13, T22, T23, T24, deg7, Syn_SCF, T25, T26, T27, T30, 2 T33, 2 T34, T39, T40, T10, T11, T12, T18, T19, Syn_CYLD, diss1, T21, T20, deg4, T38, diss2, T42, 2 T43, 2 T44, 2 T47, 2 CIIa_inhib, T49, deg1, Syn_IkB, 2 Syn_cFLIPL, 2 diss3, T6 |
| 162. | 4, 15, 18, 24 | 2 Syn_BAX, Syn_SMAC, Syn_Cyt_c, Syn_Apaf1, 2 Syn_Procasp9, Apoptosis, T65, deg3, T66, T67, 2 Syn_TNFR1, 2 Syn_TNF, 2 Syn_TRADD, Syn_RIP1, T69, Syn_TRAF2, Syn_cIAP, T71, Syn_TAB, Syn_TAK1, 2 Syn_NEMO, T72, 2 Syn_IKK, Syn_LUBAC, T73, Syn_NF_kB, 2 Syn_FADD, 3 Syn_Procasp8, 2 T1, 2 T2, T3, T4, T5, diss6, T14, T70, T7, T15, T8, T16, T9, T17, T13, deg5, T22, T23, T24, deg7, Syn_SCF, T25, T26, T27, T30, T33, T34, deg6, T74, T39, T40, T10, T11, T12, T18, T19, Syn_CYLD, diss1, T21, T20, deg4, T38, diss2, T53, T42, T43, T54, T61, T62, T44, T47, CIIa_inhib, T50, deg2, Syn_IkB, Syn_cFLIPL, diss3, diss5, T6, Syn_Procasp3, Syn_Bid |
| 163. | 4, 15, 18, 25 | 2 Syn_TNFR1, 2 Syn_TNF, 2 Syn_TRADD, Syn_RIP1, Syn_TRAF2, Syn_cIAP, Syn_TAB, Syn_TAK1, 2 Syn_NEMO, 2 Syn_IKK, Syn_LUBAC, Syn_NF_kB, Syn_FADD, Syn_Procasp8, Syn_RIP3, Syn_MLKL, 2 T1, 2 T2, T3, T4, T5, T14, T58, T51, T7, T15, T52, T8, T16, T9, T17, T13, T22, T23, T24, deg7, Syn_SCF, T25, T26, T27, T30, T33, T34, diss4, T39, T40, T10, T11, T12, T18, T19, Syn_CYLD, diss1, T21, T20, deg4, T38, diss2, T42, T43, T44, Necroptosis, T47, CIIa_inhib, T50, deg2, Syn_IkB, Syn_cFLIPL, diss3, T6 |
| 164. | 4, 15, 18, 29 | 2 Syn_BAX, Syn_SMAC, Syn_Cyt_c, Syn_Apaf1, 2 Syn_Procasp9, T65, deg3, T66, T67, 2 Syn_TNFR1, 2 Syn_TNF, 2 Syn_TRADD, Syn_RIP1, T69, Syn_TRAF2, Syn_cIAP, T71, Syn_TAB, Syn_TAK1, 2 Syn_NEMO, T72, 2 Syn_IKK, Syn_LUBAC, T73, Syn_NF_kB, 2 Syn_FADD, 3 Syn_Procasp8, 2 T1, 2 T2, T3, T4, T5, diss6, T14, T64, CASP3_inhib, T70, T7, T15, T8, T16, T9, T17, T13, deg5, T22, T23, T24, deg7, Syn_SCF, T25, T26, T27, T30, T31, T33, T32, T34, deg6, T74, T39, T40, T10, T11, T12, T18, T19, Syn_CYLD, diss1, T21, T20, deg4, T38, diss2, T53, T42, T43, T54, T61, T62, T44, T47, CIIa_inhib, T50, deg2, Syn_IkB, Syn_XIAP, Syn_cFLIPL, diss3, diss5, T6, Syn_Procasp3, Syn_Bid |
| 165. | 15, 16, 18, 40 | 2 Syn_BAX, Syn_SMAC, Syn_Cyt_c, Syn_Apaf1, Syn_Procasp9, T65, deg3, T66, T67, Syn_TNFR1, Syn_TNF, Syn_TRADD, Syn_RIP1, T69, Syn_TRAF2, Syn_cIAP, T71, Syn_TAB, Syn_TAK1, 2 Syn_NEMO, T72, 2 Syn_IKK, Syn_LUBAC, Syn_NF_kB, Syn_FADD, T76, 2 Syn_Procasp8, Apo_XIAP_inhib, T75, T1, T2, T3, T4, T5, T14, T70, T7, T15, T8, T16, T9, T17, T13, 2 T22, T37, 2 T23, 2 T24, 2 deg7, 2 Syn_SCF, T25, 2 T26, 2 T27, T30, T31, T32, T39, T40, T10, T11, T12, T18, T19, Syn_CYLD, diss1, T21, T20, deg4, T38, T43, T44, T45, T46, T49, deg1, 2 Syn_IkB, Syn_XIAP, diss3, T6, Syn_Bid |
| 166. | 4, 15, 18, 30 | 2 Syn_BAX, Syn_SMAC, Syn_Cyt_c, Syn_Apaf1, 2 Syn_Procasp9, T65, deg3, T66, T67, 2 Syn_TNFR1, 2 Syn_TNF, 2 Syn_TRADD, Syn_RIP1, T69, Syn_TRAF2, Syn_cIAP, T71, Syn_TAB, Syn_TAK1, 2 Syn_NEMO, T72, 2 Syn_IKK, Syn_LUBAC, T73, Syn_NF_kB, 2 Syn_FADD, 3 Syn_Procasp8, 2 T1, 2 T2, T3, T4, T5, diss6, T14, T64, CASP3_inhib, T70, T7, T15, T8, T16, T9, T17, T13, deg5, T22, T23, T24, deg7, Syn_SCF, T25, T26, T27, T30, T31, T33, T32, T34, deg6, T74, T39, T40, T10, T11, T12, T18, T19, Syn_CYLD, diss1, T21, T20, deg4, T38, diss2, T42, 2 T43, 2 T44, T45, T46, T47, CIIa_inhib, T49, deg1, Syn_IkB, Syn_XIAP, Syn_cFLIPL, 2 diss3, T6, Syn_Procasp3, Syn_Bid |
| 167. | 4, 15, 18, 34 | 2 Syn_BAX, Syn_SMAC, Syn_Cyt_c, Syn_Apaf1, Syn_Procasp9, T65, deg3, T66, T67, 2 Syn_TNFR1, 2 Syn_TNF, 2 Syn_TRADD, Syn_RIP1, T69, Syn_TRAF2, Syn_cIAP, T71, Syn_TAB, Syn_TAK1, 2 Syn_NEMO, T72, 2 Syn_IKK, Syn_LUBAC, Syn_NF_kB, 2 Syn_FADD, 3 Syn_Procasp8, T75, Pc9_inhib, 2 T1, 2 T2, T3, T4, T5, T14, T70, T7, T15, T8, T16, T9, T17, T13, deg5, T22, T23, T24, deg7, Syn_SCF, T25, T26, T27, T30, T31, T33, T32, T34, T39, T40, T10, T11, T12, T18, T19, Syn_CYLD, diss1, T21, T20, deg4, T38, diss2, T53, T42, T43, T54, T61, T62, T44, T47, CIIa_inhib, T50, deg2, Syn_IkB, Syn_XIAP, Syn_cFLIPL, diss3, diss5, T6, Syn_Bid |
| 168. | 4, 15, 18, 35 | 2 Syn_BAX, Syn_SMAC, Syn_Cyt_c, Syn_Apaf1, Syn_Procasp9, T65, deg3, T66, T67, 2 Syn_TNFR1, 2 Syn_TNF, 2 Syn_TRADD, Syn_RIP1, T69, Syn_TRAF2, Syn_cIAP, T71, Syn_TAB, Syn_TAK1, 2 Syn_NEMO, T72, 2 Syn_IKK, Syn_LUBAC, Syn_NF_kB, 2 Syn_FADD, 3 Syn_Procasp8, T75, Pc9_inhib, 2 T1, 2 T2, T3, T4, T5, T14, T70, T7, T15, T8, T16, T9, T17, T13, deg5, T22, T23, T24, deg7, Syn_SCF, T25, T26, T27, T30, T31, T33, T32, T34, T39, T40, T10, T11, T12, T18, T19, Syn_CYLD, diss1, T21, T20, deg4, T38, diss2, T42, 2 T43, 2 T44, T45, T46, T47, CIIa_inhib, T49, deg1, Syn_IkB, Syn_XIAP, Syn_cFLIPL, 2 diss3, T6, Syn_Bid |
| 169. | 15, 18, 31, 45 | 3 Syn_BAX, Syn_SMAC, Syn_Cyt_c, Syn_Apaf1, Syn_Procasp9, 2 T65, 2 deg3, 2 T66, 2 T67, T68, 2 Syn_TNFR1, 2 Syn_TNF, BAX_inhib, 2 Syn_TRADD, 2 Syn_RIP1, T69, Syn_TRAF2, Syn_cIAP, T71, Syn_TAB, Syn_TAK1, 2 Syn_NEMO, T72, 2 Syn_IKK, Syn_LUBAC, Syn_NF_kB, 2 Syn_FADD, 4 Syn_Procasp8, T75, Pc9_inhib, 2 T1, 2 T2, 2 T3, T4, T5, T14, T70, T7, T15, T8, T16, T9, T17, T13, deg5, T22, T23, T24, deg7, Syn_SCF, T25, T26, T27, T30, T31, T35, T32, T36, T39, T40, T10, T11, T12, T18, T19, Syn_CYLD, diss1, T21, T20, deg4, T38, T48, diss2, T53, T43, T54, T61, T62, T44, T45, T46, T50, deg2, T49, deg1, Syn_IkB, Syn_XIAP, Syn_BCL_2, diss3, diss5, T6, 2 Syn_Bid |
| 170. | 15, 17, 18, 30 | 2 Syn_BAX, Syn_SMAC, Syn_Cyt_c, Syn_Apaf1, 2 Syn_Procasp9, T65, deg3, T66, T67, 2 Syn_TNFR1, 2 Syn_TNF, 2 Syn_TRADD, 2 Syn_RIP1, T69, Syn_TRAF2, Syn_cIAP, T71, Syn_TAB, Syn_TAK1, 2 Syn_NEMO, T72, 2 Syn_IKK, Syn_LUBAC, T73, Syn_NF_kB, 2 Syn_FADD, 3 Syn_Procasp8, 2 T1, 2 T2, 2 T3, T4, T5, diss6, T14, T64, CASP3_inhib, T70, T7, T15, T8, T16, T9, T17, T13, deg5, T22, T23, T24, deg7, Syn_SCF, T25, T26, T27, T30, T31, T33, T32, T34, deg6, T74, T39, T40, T10, T11, T12, T18, T19, Syn_CYLD, diss1, T21, T20, deg4, T38, T48, diss2, 2 T43, 2 T44, T45, T46, T47, CIIa_inhib, 2 T49, 2 deg1, Syn_IkB, Syn_XIAP, Syn_cFLIPL, 2 diss3, T6, Syn_Procasp3, Syn_Bid |
| 171. | 15, 18, 28, 40 | 4 Syn_BAX, 2 Syn_SMAC, 2 Syn_Cyt_c, 2 Syn_Apaf1, 3 Syn_Procasp9, 2 T65, 2 deg3, 2 T66, 2 T67, 2 Syn_TNFR1, 2 Syn_TNF, 2 Syn_TRADD, 2 Syn_RIP1, 2 T69, Syn_TRAF2, Syn_cIAP, 2 T71, Syn_TAB, Syn_TAK1, 2 Syn_NEMO, 2 T72, 2 Syn_IKK, Syn_LUBAC, T73, Syn_NF_kB, 2 Syn_FADD, T76, 4 Syn_Procasp8, Apo_XIAP_inhib, T75, 2 T1, 2 T2, 2 T3, T4, T5, diss6, T14, T64, CASP3_inhib, 2 T70, T7, T15, T8, T16, T9, T17, T13, deg5, T22, T23, T24, deg7, Syn_SCF, T25, T26, T27, T30, 2 T31, 2 T32, deg6, T74, T39, T40, T10, T11, T12, T18, T19, Syn_CYLD, diss1, T21, T20, deg4, T38, T48, diss2, 2 T43, 2 T44, 2 T45, 2 T46, 2 T49, 2 deg1, Syn_IkB, 2 Syn_XIAP, 2 diss3, T6, Syn_Procasp3, 2 Syn_Bid |
| 172. | 15, 18, 31, 35 | 4 Syn_BAX, 2 Syn_SMAC, 2 Syn_Cyt_c, 2 Syn_Apaf1, 2 Syn_Procasp9, 2 T65, 2 deg3, 2 T66, 2 T67, 2 Syn_TNFR1, 2 Syn_TNF, 2 Syn_TRADD, 2 Syn_RIP1, 2 T69, Syn_TRAF2, Syn_cIAP, 2 T71, Syn_TAB, Syn_TAK1, 2 Syn_NEMO, 2 T72, 2 Syn_IKK, Syn_LUBAC, Syn_NF_kB, 2 Syn_FADD, 4 Syn_Procasp8, 2 T75, 2 Pc9_inhib, 2 T1, 2 T2, 2 T3, T4, T5, T14, 2 T70, T7, T15, T8, T16, T9, T17, T13, 2 deg5, T22, T23, T24, deg7, Syn_SCF, T25, T26, T27, T30, 2 T31, 2 T32, T39, T40, T10, T11, T12, T18, T19, Syn_CYLD, diss1, T21, T20, deg4, T38, T48, diss2, T53, T43, T54, T61, T62, T44, T45, T46, T50, deg2, T49, deg1, Syn_IkB, 2 Syn_XIAP, diss3, diss5, T6, 2 Syn_Bid |
| 173. | 9, 15, 17, 18 | 2 Syn_TNFR1, 2 Syn_TNF, 2 Syn_TRADD, 2 Syn_RIP1, Syn_TRAF2, Syn_cIAP, Syn_TAB, Syn_TAK1, 2 Syn_NEMO, 2 Syn_IKK, Syn_LUBAC, Syn_NF_kB, Syn_FADD, Syn_Procasp8, 2 T1, 2 T2, 2 T3, T4, T5, T14, T7, T15, T8, T16, T9, T17, T13, T22, T23, T24, deg7, Syn_SCF, T25, T26, T28, T29_, T27, T30, T33, T34, T39, T40, T41, CI_diss, T10, T11, T12, deg4, T38, T48, diss2, T43, T44, T47, CIIa_inhib, T49, deg1, Syn_IkB, Syn_A20, Syn_cFLIPL, diss3, T6 |
| 174. | 14, 15, 18, 22 | deg3, 2 Syn_TNFR1, 2 Syn_TNF, 2 Syn_TRADD, 2 Syn_RIP1, Syn_TRAF2, Syn_cIAP, Syn_TAB, Syn_TAK1, 2 Syn_NEMO, 2 Syn_IKK, Syn_LUBAC, Syn_NF_kB, 2 Syn_FADD, 3 Syn_Procasp8, Syn_RIP3, 2 T1, 2 T2, 2 T3, T4, T5, T14, T60, RIP1_RIP3_inhib, T64, CASP3_inhib, T7, T15, T8, T16, T9, T17, T13, T22, T23, T24, deg7, Syn_SCF, T25, T26, T27, T30, T31, T33, T32, T34, T63, T39, T40, T10, T11, T12, T18, T19, Syn_CYLD, diss1, T21, T20, deg4, T38, T48, diss2, 2 T53, 2 T54, T61, T62, T59, Pc8_inhib, 2 T50, 2 deg2, Syn_IkB, Syn_XIAP, Syn_cFLIPL, 2 diss5, T6, Syn_Procasp3 |
| 175. | 14, 15, 18, 23 | deg3, 2 Syn_TNFR1, 2 Syn_TNF, 2 Syn_TRADD, 2 Syn_RIP1, Syn_TRAF2, Syn_cIAP, Syn_TAB, Syn_TAK1, 2 Syn_NEMO, 2 Syn_IKK, Syn_LUBAC, Syn_NF_kB, 2 Syn_FADD, 3 Syn_Procasp8, 2 T1, 2 T2, 2 T3, T4, T5, T14, T64, CASP3_inhib, T7, T15, T8, T16, T9, T17, T13, T22, T23, T24, deg7, Syn_SCF, T25, T26, T27, T30, T31, T33, T32, T34, T63, T39, T40, T10, T11, T12, T18, T19, Syn_CYLD, diss1, T21, T20, deg4, T38, T48, diss2, T53, T43, T54, T61, T62, T44, T47, CIIa_inhib, T50, deg2, T49, deg1, Syn_IkB, Syn_XIAP, Syn_cFLIPL, diss3, diss5, T6, Syn_Procasp3 |
| 176. | 15, 18, 34, 41 | 3 Syn_BAX, Syn_SMAC, Syn_Cyt_c, Syn_Apaf1, Syn_Procasp9, 2 T65, 2 deg3, 2 T66, 2 T67, T68, 2 Syn_TNFR1, 2 Syn_TNF, BAX_inhib, 2 Syn_TRADD, 2 Syn_RIP1, T69, Syn_TRAF2, Syn_cIAP, T71, Syn_TAB, Syn_TAK1, 2 Syn_NEMO, T72, 2 Syn_IKK, Syn_LUBAC, Syn_NF_kB, 2 Syn_FADD, 4 Syn_Procasp8, T75, Pc9_inhib, 2 T1, 2 T2, 2 T3, T4, T5, T14, T70, T7, T15, T8, T16, T9, T17, T13, deg5, T22, T23, T24, deg7, Syn_SCF, T25, T26, T27, T30, T31, T35, T32, T36, T39, T40, T10, T11, T12, T18, T19, Syn_CYLD, diss1, T21, T20, deg4, T38, T48, diss2, 2 T53, 2 T54, 2 T61, 2 T62, 2 T50, 2 deg2, Syn_IkB, Syn_XIAP, Syn_BCL_2, 2 diss5, T6, 2 Syn_Bid |
| 177. | 14, 15, 18, 25 | deg3, 2 Syn_TNFR1, 2 Syn_TNF, 2 Syn_TRADD, 2 Syn_RIP1, Syn_TRAF2, Syn_cIAP, Syn_TAB, Syn_TAK1, 2 Syn_NEMO, 2 Syn_IKK, Syn_LUBAC, Syn_NF_kB, Syn_FADD, 2 Syn_Procasp8, Syn_RIP3, Syn_MLKL, 2 T1, 2 T2, 2 T3, T4, T5, T14, T58, T64, CASP3_inhib, T51, T7, T15, T52, T8, T16, T9, T17, T13, T22, T23, T24, deg7, Syn_SCF, T25, T26, T27, T30, T31, T32, diss4, T63, T39, T40, T10, T11, T12, T18, T19, Syn_CYLD, diss1, T21, T20, deg4, T38, T48, diss2, T53, T54, T61, T62, Necroptosis, 2 T50, 2 deg2, Syn_IkB, Syn_XIAP, diss5, T6, Syn_Procasp3 |
| 178. | 14, 15, 18, 29 | 2 Syn_BAX, Syn_SMAC, Syn_Cyt_c, Syn_Apaf1, 2 Syn_Procasp9, T65, 2 deg3, T66, T67, 2 Syn_TNFR1, 2 Syn_TNF, 2 Syn_TRADD, 2 Syn_RIP1, T69, Syn_TRAF2, Syn_cIAP, T71, Syn_TAB, Syn_TAK1, 2 Syn_NEMO, T72, 2 Syn_IKK, Syn_LUBAC, T73, Syn_NF_kB, 2 Syn_FADD, 4 Syn_Procasp8, 2 T1, 2 T2, 2 T3, T4, T5, diss6, T14, 2 T64, 2 CASP3_inhib, T70, T7, T15, T8, T16, T9, T17, T13, deg5, T22, T23, T24, deg7, Syn_SCF, T25, T26, T27, T30, 2 T31, 2 T32, deg6, T63, T74, T39, T40, T10, T11, T12, T18, T19, Syn_CYLD, diss1, T21, T20, deg4, T38, T48, diss2, 2 T53, 2 T54, 2 T61, 2 T62, 2 T50, 2 deg2, Syn_IkB, 2 Syn_XIAP, 2 diss5, T6, 2 Syn_Procasp3, Syn_Bid |
| 179. | 14, 15, 18, 30 | 2 Syn_BAX, Syn_SMAC, Syn_Cyt_c, Syn_Apaf1, 2 Syn_Procasp9, T65, 2 deg3, T66, T67, 2 Syn_TNFR1, 2 Syn_TNF, 2 Syn_TRADD, 2 Syn_RIP1, T69, Syn_TRAF2, Syn_cIAP, T71, Syn_TAB, Syn_TAK1, 2 Syn_NEMO, T72, 2 Syn_IKK, Syn_LUBAC, T73, Syn_NF_kB, 2 Syn_FADD, 4 Syn_Procasp8, 2 T1, 2 T2, 2 T3, T4, T5, diss6, T14, 2 T64, 2 CASP3_inhib, T70, T7, T15, T8, T16, T9, T17, T13, deg5, T22, T23, T24, deg7, Syn_SCF, T25, T26, T27, T30, 2 T31, 2 T32, deg6, T63, T74, T39, T40, T10, T11, T12, T18, T19, Syn_CYLD, diss1, T21, T20, deg4, T38, T48, diss2, T53, T43, T54, T61, T62, T44, T45, T46, T50, deg2, T49, deg1, Syn_IkB, 2 Syn_XIAP, diss3, diss5, T6, 2 Syn_Procasp3, Syn_Bid |
| 180. | 14, 15, 18, 34 | 2 Syn_BAX, Syn_SMAC, Syn_Cyt_c, Syn_Apaf1, Syn_Procasp9, T65, 2 deg3, T66, T67, 2 Syn_TNFR1, 2 Syn_TNF, 2 Syn_TRADD, 2 Syn_RIP1, T69, Syn_TRAF2, Syn_cIAP, T71, Syn_TAB, Syn_TAK1, 2 Syn_NEMO, T72, 2 Syn_IKK, Syn_LUBAC, Syn_NF_kB, 2 Syn_FADD, 4 Syn_Procasp8, T75, Pc9_inhib, 2 T1, 2 T2, 2 T3, T4, T5, T14, T64, CASP3_inhib, T70, T7, T15, T8, T16, T9, T17, T13, deg5, T22, T23, T24, deg7, Syn_SCF, T25, T26, T27, T30, 2 T31, 2 T32, T63, T39, T40, T10, T11, T12, T18, T19, Syn_CYLD, diss1, T21, T20, deg4, T38, T48, diss2, 2 T53, 2 T54, 2 T61, 2 T62, 2 T50, 2 deg2, Syn_IkB, 2 Syn_XIAP, 2 diss5, T6, Syn_Procasp3, Syn_Bid |
| 181. | 15, 18, 25, 27 | 2 Syn_BAX, Syn_SMAC, Syn_Cyt_c, Syn_Apaf1, 2 Syn_Procasp9, T65, deg3, T66, T67, 2 Syn_TNFR1, 2 Syn_TNF, 2 Syn_TRADD, Syn_RIP1, T69, Syn_TRAF2, Syn_cIAP, T71, Syn_TAB, Syn_TAK1, 2 Syn_NEMO, T72, 2 Syn_IKK, Syn_LUBAC, T73, Syn_NF_kB, Syn_FADD, 2 Syn_Procasp8, Syn_RIP3, Syn_MLKL, 2 T1, 2 T2, T3, T4, T5, diss6, T14, T58, T64, CASP3_inhib, T70, T51, T7, T15, T52, T8, T16, T9, T17, T13, deg5, T22, T23, T24, deg7, Syn_SCF, T25, T26, T27, T30, T31, T32, diss4, deg6, T74, T39, T40, T10, T11, T12, T18, T19, Syn_CYLD, diss1, T21, T20, deg4, T38, diss2, T42, T43, T44, T45, Necroptosis, T46, T50, deg2, Syn_IkB, Syn_XIAP, diss3, T6, Syn_Procasp3, Syn_Bid |
| 182. | 15, 18, 32, 40 | 4 Syn_BAX, 2 Syn_SMAC, 2 Syn_Cyt_c, 2 Syn_Apaf1, 2 Syn_Procasp9, 2 T65, 2 deg3, 2 T66, 2 T67, 2 Syn_TNFR1, 2 Syn_TNF, 2 Syn_TRADD, Syn_RIP1, 2 T69, Syn_TRAF2, Syn_cIAP, 2 T71, Syn_TAB, Syn_TAK1, 2 Syn_NEMO, 2 T72, 2 Syn_IKK, Syn_LUBAC, Syn_NF_kB, 2 Syn_FADD, T76, 4 Syn_Procasp8, Apo_XIAP_inhib, 2 T75, Pc9_inhib, 2 T1, 2 T2, T3, T4, T5, T14, 2 T70, T7, T15, T8, T16, T9, T17, T13, deg5, T22, T23, T24, deg7, Syn_SCF, T25, T26, T27, T30, 2 T31, 2 T32, T39, T40, T10, T11, T12, T18, T19, Syn_CYLD, diss1, T21, T20, deg4, T38, diss2, T42, 2 T43, 2 T44, 2 T45, 2 T46, T49, deg1, Syn_IkB, 2 Syn_XIAP, 2 diss3, T6, 2 Syn_Bid |
| 183. | 15, 18, 26, 29 | 4 Syn_BAX, 2 Syn_SMAC, 2 Syn_Cyt_c, 2 Syn_Apaf1, 4 Syn_Procasp9, 2 T65, 2 deg3, 2 T66, 2 T67, 2 Syn_TNFR1, 2 Syn_TNF, 2 Syn_TRADD, 2 Syn_RIP1, 2 T69, Syn_TRAF2, Syn_cIAP, 2 T71, Syn_TAB, Syn_TAK1, 2 Syn_NEMO, 2 T72, 2 Syn_IKK, Syn_LUBAC, 2 T73, Syn_NF_kB, 2 Syn_FADD, 4 Syn_Procasp8, 2 T1, 2 T2, 2 T3, T4, T5, 2 diss6, T14, 2 T64, 2 CASP3_inhib, 2 T70, T7, T15, T8, T16, T9, T17, T13, 2 deg5, T22, T23, T24, deg7, Syn_SCF, T25, T26, T27, T30, 2 T31, 2 T32, 2 deg6, 2 T74, T39, T40, T10, T11, T12, T18, T19, Syn_CYLD, diss1, T21, T20, deg4, T38, T48, diss2, 2 T53, 2 T54, 2 T61, 2 T62, 2 T50, 2 deg2, Syn_IkB, 2 Syn_XIAP, 2 diss5, T6, 2 Syn_Procasp3, 2 Syn_Bid |
| 184. | 15, 18, 22, 42 15, 18, 40, 41 | Syn_BAX, T65, deg3, T66, T67, T68, 2 Syn_TNFR1, 2 Syn_TNF, BAX_inhib, 2 Syn_TRADD, Syn_RIP1, Syn_TRAF2, Syn_cIAP, Syn_TAB, Syn_TAK1, 2 Syn_NEMO, 2 Syn_IKK, Syn_LUBAC, Syn_NF_kB, 2 Syn_FADD, 3 Syn_Procasp8, Syn_RIP3, 2 T1, 2 T2, T3, T4, T5, T14, T60, RIP1_RIP3_inhib, T7, T15, T8, T16, T9, T17, T13, T22, T23, T24, deg7, Syn_SCF, T25, T26, T27, T30, T33, T35, T34, T36, T39, T40, T10, T11, T12, T18, T19, Syn_CYLD, diss1, T21, T20, deg4, T38, diss2, T53, T42, T43, T54, T59, Pc8_inhib, T44, T45, T46, T50, deg2, Syn_IkB, Syn_cFLIPL, Syn_BCL_2, diss3, diss5, T6, Syn_Bid |
| 185. | 15, 18, 40, 41 | 3 Syn_BAX, Syn_SMAC, Syn_Cyt_c, Syn_Apaf1, Syn_Procasp9, 2 T65, 2 deg3, 2 T66, 2 T67, T68, 2 Syn_TNFR1, 2 Syn_TNF, BAX_inhib, 2 Syn_TRADD, 2 Syn_RIP1, T69, Syn_TRAF2, Syn_cIAP, T71, Syn_TAB, Syn_TAK1, 2 Syn_NEMO, T72, 2 Syn_IKK, Syn_LUBAC, Syn_NF_kB, 2 Syn_FADD, T76, 4 Syn_Procasp8, Apo_XIAP_inhib, T75, 2 T1, 2 T2, 2 T3, T4, T5, T14, T70, T7, T15, T8, T16, T9, T17, T13, T22, T23, T24, deg7, Syn_SCF, T25, T26, T27, T30, T31, T35, T32, T36, T39, T40, T10, T11, T12, T18, T19, Syn_CYLD, diss1, T21, T20, deg4, T38, T48, diss2, T53, T43, T54, T61, T62, T44, T45, T46, T50, deg2, T49, deg1, Syn_IkB, Syn_XIAP, Syn_BCL_2, diss3, diss5, T6, 2 Syn_Bid |
| 186. | 15, 18, 40, 42 15, 18, 40, 43 | 3 Syn_BAX, Syn_SMAC, Syn_Cyt_c, Syn_Apaf1, Syn_Procasp9, 2 T65, 2 deg3, 2 T66, 2 T67, T68, 2 Syn_TNFR1, 2 Syn_TNF, BAX_inhib, 2 Syn_TRADD, Syn_RIP1, T69, Syn_TRAF2, Syn_cIAP, T71, Syn_TAB, Syn_TAK1, 2 Syn_NEMO, T72, 2 Syn_IKK, Syn_LUBAC, Syn_NF_kB, 2 Syn_FADD, T76, 4 Syn_Procasp8, Apo_XIAP_inhib, T75, 2 T1, 2 T2, T3, T4, T5, T14, T70, T7, T15, T8, T16, T9, T17, T13, T22, T23, T24, deg7, Syn_SCF, T25, T26, T27, T30, T31, T35, T32, T36, T39, T40, T10, T11, T12, T18, T19, Syn_CYLD, diss1, T21, T20, deg4, T38, diss2, T42, 2 T43, 2 T44, 2 T45, 2 T46, T49, deg1, Syn_IkB, Syn_XIAP, Syn_BCL_2, 2 diss3, T6, 2 Syn_Bid |
| 187. | 15, 18, 40, 43 | 3 Syn_BAX, Syn_SMAC, Syn_Cyt_c, Syn_Apaf1, Syn_Procasp9, 2 T65, 2 deg3, 2 T66, 2 T67, T68, 2 Syn_TNFR1, 2 Syn_TNF, BAX_inhib, 2 Syn_TRADD, 2 Syn_RIP1, T69, Syn_TRAF2, Syn_cIAP, T71, Syn_TAB, Syn_TAK1, 2 Syn_NEMO, T72, 2 Syn_IKK, Syn_LUBAC, Syn_NF_kB, 2 Syn_FADD, T76, 4 Syn_Procasp8, Apo_XIAP_inhib, T75, 2 T1, 2 T2, 2 T3, T4, T5, T14, T70, T7, T15, T8, T16, T9, T17, T13, T22, T23, T24, deg7, Syn_SCF, T25, T26, T27, T30, T31, T35, T32, T36, T39, T40, T10, T11, T12, T18, T19, Syn_CYLD, diss1, T21, T20, deg4, T38, T48, diss2, 2 T43, 2 T44, 2 T45, 2 T46, 2 T49, 2 deg1, Syn_IkB, Syn_XIAP, Syn_BCL_2, 2 diss3, T6, 2 Syn_Bid |
| 188. | 15, 17, 18, 29 | 2 Syn_BAX, Syn_SMAC, Syn_Cyt_c, Syn_Apaf1, 2 Syn_Procasp9, T65, deg3, T66, T67, 2 Syn_TNFR1, 2 Syn_TNF, 2 Syn_TRADD, 2 Syn_RIP1, T69, Syn_TRAF2, Syn_cIAP, T71, Syn_TAB, Syn_TAK1, 2 Syn_NEMO, T72, 2 Syn_IKK, Syn_LUBAC, T73, Syn_NF_kB, 2 Syn_FADD, 3 Syn_Procasp8, 2 T1, 2 T2, 2 T3, T4, T5, diss6, T14, T64, CASP3_inhib, T70, T7, T15, T8, T16, T9, T17, T13, deg5, T22, T23, T24, deg7, Syn_SCF, T25, T26, T27, T30, T31, T33, T32, T34, deg6, T74, T39, T40, T10, T11, T12, T18, T19, Syn_CYLD, diss1, T21, T20, deg4, T38, T48, diss2, T53, T43, T54, T61, T62, T44, T47, CIIa_inhib, T50, deg2, T49, deg1, Syn_IkB, Syn_XIAP, Syn_cFLIPL, diss3, diss5, T6, Syn_Procasp3, Syn_Bid |
| 189. | 15, 17, 18, 25 | 2 Syn_TNFR1, 2 Syn_TNF, 2 Syn_TRADD, 2 Syn_RIP1, Syn_TRAF2, Syn_cIAP, Syn_TAB, Syn_TAK1, 2 Syn_NEMO, 2 Syn_IKK, Syn_LUBAC, Syn_NF_kB, Syn_FADD, Syn_Procasp8, Syn_RIP3, Syn_MLKL, 2 T1, 2 T2, 2 T3, T4, T5, T14, T58, T51, T7, T15, T52, T8, T16, T9, T17, T13, T22, T23, T24, deg7, Syn_SCF, T25, T26, T27, T30, T33, T34, diss4, T39, T40, T10, T11, T12, T18, T19, Syn_CYLD, diss1, T21, T20, deg4, T38, T48, diss2, T43, T44, Necroptosis, T47, CIIa_inhib, T50, deg2, T49, deg1, Syn_IkB, Syn_cFLIPL, diss3, T6 |
| 190. | 15, 17, 18, 24 | 2 Syn_BAX, Syn_SMAC, Syn_Cyt_c, Syn_Apaf1, 2 Syn_Procasp9, Apoptosis, T65, deg3, T66, T67, 2 Syn_TNFR1, 2 Syn_TNF, 2 Syn_TRADD, 2 Syn_RIP1, T69, Syn_TRAF2, Syn_cIAP, T71, Syn_TAB, Syn_TAK1, 2 Syn_NEMO, T72, 2 Syn_IKK, Syn_LUBAC, T73, Syn_NF_kB, 2 Syn_FADD, 3 Syn_Procasp8, 2 T1, 2 T2, 2 T3, T4, T5, diss6, T14, T70, T7, T15, T8, T16, T9, T17, T13, deg5, T22, T23, T24, deg7, Syn_SCF, T25, T26, T27, T30, T33, T34, deg6, T74, T39, T40, T10, T11, T12, T18, T19, Syn_CYLD, diss1, T21, T20, deg4, T38, T48, diss2, T53, T43, T54, T61, T62, T44, T47, CIIa_inhib, T50, deg2, T49, deg1, Syn_IkB, Syn_cFLIPL, diss3, diss5, T6, Syn_Procasp3, Syn_Bid |
| 191. | 15, 17, 18, 23 | 2 Syn_TNFR1, 2 Syn_TNF, 2 Syn_TRADD, 2 Syn_RIP1, Syn_TRAF2, Syn_cIAP, Syn_TAB, Syn_TAK1, 2 Syn_NEMO, 2 Syn_IKK, Syn_LUBAC, Syn_NF_kB, 2 Syn_FADD, 2 Syn_Procasp8, 2 T1, 2 T2, 2 T3, T4, T5, T14, T7, T15, T8, T16, T9, T17, T13, T22, T23, T24, deg7, Syn_SCF, T25, T26, T27, T30, 2 T33, 2 T34, T39, T40, T10, T11, T12, T18, T19, Syn_CYLD, diss1, T21, T20, deg4, T38, T48, diss2, 2 T43, 2 T44, 2 T47, 2 CIIa_inhib, 2 T49, 2 deg1, Syn_IkB, 2 Syn_cFLIPL, 2 diss3, T6 |
| 192. | 15, 16, 18, 29 | 2 Syn_BAX, Syn_SMAC, Syn_Cyt_c, Syn_Apaf1, 2 Syn_Procasp9, T65, deg3, T66, T67, Syn_TNFR1, Syn_TNF, Syn_TRADD, Syn_RIP1, T69, Syn_TRAF2, Syn_cIAP, T71, Syn_TAB, Syn_TAK1, 2 Syn_NEMO, T72, 2 Syn_IKK, Syn_LUBAC, T73, Syn_NF_kB, Syn_FADD, 2 Syn_Procasp8, T1, T2, T3, T4, T5, diss6, T14, T64, CASP3_inhib, T70, T7, T15, T8, T16, T9, T17, T13, deg5, 2 T22, T37, 2 T23, 2 T24, 2 deg7, 2 Syn_SCF, T25, 2 T26, 2 T27, T30, T31, T32, deg6, T74, T39, T40, T10, T11, T12, T18, T19, Syn_CYLD, diss1, T21, T20, deg4, T38, T53, T54, T61, T62, T50, deg2, 2 Syn_IkB, Syn_XIAP, diss5, T6, Syn_Procasp3, Syn_Bid |
| 193. | 15, 17, 18, 21 | 2 Syn_BAX, Syn_SMAC, Syn_Cyt_c, Syn_Apaf1, 2 Syn_Procasp9, Apoptosis, T65, deg3, T66, T67, 2 Syn_TNFR1, 2 Syn_TNF, 2 Syn_TRADD, 2 Syn_RIP1, T69, Syn_TRAF2, Syn_cIAP, T71, Syn_TAB, Syn_TAK1, 2 Syn_NEMO, T72, 2 Syn_IKK, Syn_LUBAC, T73, Syn_NF_kB, 2 Syn_FADD, 3 Syn_Procasp8, 2 T1, 2 T2, 2 T3, T4, T5, diss6, T14, T70, T7, T15, T8, T16, T9, T17, T13, deg5, T22, T23, T24, deg7, Syn_SCF, T25, T26, T27, T30, T33, T34, deg6, T74, T39, T40, T10, T11, T12, T18, T19, Syn_CYLD, diss1, T21, T20, deg4, T38, T48, diss2, 2 T43, 2 T44, T45, T46, T47, CIIa_inhib, 2 T49, 2 deg1, Syn_IkB, Syn_cFLIPL, 2 diss3, T6, Syn_Procasp3, Syn_Bid |
| 194. | 4, 15, 18, 45 | Syn_BAX, T65, deg3, T66, T67, T68, 2 Syn_TNFR1, 2 Syn_TNF, BAX_inhib, 2 Syn_TRADD, Syn_RIP1, Syn_TRAF2, Syn_cIAP, Syn_TAB, Syn_TAK1, 2 Syn_NEMO, 2 Syn_IKK, Syn_LUBAC, Syn_NF_kB, 2 Syn_FADD, 3 Syn_Procasp8, 2 T1, 2 T2, T3, T4, T5, T14, T7, T15, T8, T16, T9, T17, T13, T22, T23, T24, deg7, Syn_SCF, T25, T26, T27, T30, T33, T35, T34, T36, T39, T40, T10, T11, T12, T18, T19, Syn_CYLD, diss1, T21, T20, deg4, T38, diss2, T42, 2 T43, 2 T44, T45, T46, T47, CIIa_inhib, T49, deg1, Syn_IkB, Syn_cFLIPL, Syn_BCL_2, 2 diss3, T6, Syn_Bid |
| 195. | 4, 15, 18, 44 | Syn_BAX, T65, deg3, T66, T67, T68, 2 Syn_TNFR1, 2 Syn_TNF, BAX_inhib, 2 Syn_TRADD, Syn_RIP1, Syn_TRAF2, Syn_cIAP, Syn_TAB, Syn_TAK1, 2 Syn_NEMO, 2 Syn_IKK, Syn_LUBAC, Syn_NF_kB, 2 Syn_FADD, 3 Syn_Procasp8, 2 T1, 2 T2, T3, T4, T5, T14, T7, T15, T8, T16, T9, T17, T13, T22, T23, T24, deg7, Syn_SCF, T25, T26, T27, T30, T33, T35, T34, T36, T39, T40, T10, T11, T12, T18, T19, Syn_CYLD, diss1, T21, T20, deg4, T38, diss2, T53, T42, T43, T54, T61, T62, T44, T47, CIIa_inhib, T50, deg2, Syn_IkB, Syn_cFLIPL, Syn_BCL_2, diss3, diss5, T6, Syn_Bid |
| 196. | 4, 15, 18, 40 | 2 Syn_BAX, Syn_SMAC, Syn_Cyt_c, Syn_Apaf1, Syn_Procasp9, T65, deg3, T66, T67, 2 Syn_TNFR1, 2 Syn_TNF, 2 Syn_TRADD, Syn_RIP1, T69, Syn_TRAF2, Syn_cIAP, T71, Syn_TAB, Syn_TAK1, 2 Syn_NEMO, T72, 2 Syn_IKK, Syn_LUBAC, Syn_NF_kB, 2 Syn_FADD, T76, 3 Syn_Procasp8, Apo_XIAP_inhib, T75, 2 T1, 2 T2, T3, T4, T5, T14, T70, T7, T15, T8, T16, T9, T17, T13, T22, T23, T24, deg7, Syn_SCF, T25, T26, T27, T30, T31, T33, T32, T34, T39, T40, T10, T11, T12, T18, T19, Syn_CYLD, diss1, T21, T20, deg4, T38, diss2, T42, 2 T43, 2 T44, T45, T46, T47, CIIa_inhib, T49, deg1, Syn_IkB, Syn_XIAP, Syn_cFLIPL, 2 diss3, T6, Syn_Bid |
| 197. | 4, 15, 18, 39 | 2 Syn_BAX, Syn_SMAC, Syn_Cyt_c, Syn_Apaf1, Syn_Procasp9, T65, deg3, T66, T67, 2 Syn_TNFR1, 2 Syn_TNF, 2 Syn_TRADD, Syn_RIP1, T69, Syn_TRAF2, Syn_cIAP, T71, Syn_TAB, Syn_TAK1, 2 Syn_NEMO, T72, 2 Syn_IKK, Syn_LUBAC, Syn_NF_kB, 2 Syn_FADD, T76, 3 Syn_Procasp8, Apo_XIAP_inhib, T75, 2 T1, 2 T2, T3, T4, T5, T14, T70, T7, T15, T8, T16, T9, T17, T13, T22, T23, T24, deg7, Syn_SCF, T25, T26, T27, T30, T31, T33, T32, T34, T39, T40, T10, T11, T12, T18, T19, Syn_CYLD, diss1, T21, T20, deg4, T38, diss2, T53, T42, T43, T54, T61, T62, T44, T47, CIIa_inhib, T50, deg2, Syn_IkB, Syn_XIAP, Syn_cFLIPL, diss3, diss5, T6, Syn_Bid |
| 198. | 15, 17, 18, 20 | 2 Syn_TNFR1, 2 Syn_TNF, 2 Syn_TRADD, 2 Syn_RIP1, Syn_TRAF2, Syn_cIAP, Syn_TAB, Syn_TAK1, 2 Syn_NEMO, 2 Syn_IKK, Syn_LUBAC, Syn_NF_kB, 2 Syn_FADD, 2 Syn_Procasp8, Syn_cFLIPs, Syn_RIP3, Syn_MLKL, 2 T1, 2 T2, 2 T3, T4, T5, T14, T58, T7, T15, T8, T16, T9, T17, T13, T22, T23, T24, deg7, Syn_SCF, T25, T26, T27, T30, T33, T34, T39, T40, T10, T11, T12, T18, T19, Syn_CYLD, diss1, T21, T20, deg4, T38, T48, diss2, T53, T43, T54, T44, T55, T56, T57, Necroptosis, T47, CIIa_inhib, T50, deg2, T49, deg1, Syn_IkB, Syn_cFLIPL, diss3, diss5, T6 |
| 199. | 15, 16, 18, 35 | 2 Syn_BAX, Syn_SMAC, Syn_Cyt_c, Syn_Apaf1, Syn_Procasp9, T65, deg3, T66, T67, Syn_TNFR1, Syn_TNF, Syn_TRADD, Syn_RIP1, T69, Syn_TRAF2, Syn_cIAP, T71, Syn_TAB, Syn_TAK1, 2 Syn_NEMO, T72, 2 Syn_IKK, Syn_LUBAC, Syn_NF_kB, Syn_FADD, 2 Syn_Procasp8, T75, Pc9_inhib, T1, T2, T3, T4, T5, T14, T70, T7, T15, T8, T16, T9, T17, T13, deg5, 2 T22, T37, 2 T23, 2 T24, 2 deg7, 2 Syn_SCF, T25, 2 T26, 2 T27, T30, T31, T32, T39, T40, T10, T11, T12, T18, T19, Syn_CYLD, diss1, T21, T20, deg4, T38, T43, T44, T45, T46, T49, deg1, 2 Syn_IkB, Syn_XIAP, diss3, T6, Syn_Bid |
| 200. | 7, 11, 15, 18 | 2 deg3, 2 Syn_TNFR1, 2 Syn_TNF, 2 Syn_TRADD, 2 Syn_RIP1, Syn_TRAF2, Syn_cIAP, Syn_TAB, Syn_TAK1, 2 Syn_NEMO, 2 Syn_IKK, Syn_LUBAC, Syn_NF_kB, 2 Syn_FADD, 4 Syn_Procasp8, 2 T1, 2 T2, 2 T3, T4, T5, T14, 2 T64, 2 CASP3_inhib, T7, T15, T8, T16, T9, T17, T13, T22, T23, T24, deg7, Syn_SCF, T25, T26, T27, T30, 2 T31, 2 T32, 2 T63, T39, T40, T10, T11, T12, T18, T19, Syn_CYLD, diss1, T21, T20, deg4, T38, T48, diss2, 2 T43, 2 T44, 2 T45, 2 T46, 2 T49, 2 deg1, Syn_IkB, 2 Syn_XIAP, 2 diss3, T6, 2 Syn_Procasp3 |
| 201. | 7, 12, 15, 18 | 2 deg3, 2 Syn_TNFR1, 2 Syn_TNF, 2 Syn_TRADD, Syn_RIP1, Syn_TRAF2, Syn_cIAP, Syn_TAB, Syn_TAK1, 2 Syn_NEMO, 2 Syn_IKK, Syn_LUBAC, Syn_NF_kB, 2 Syn_FADD, 4 Syn_Procasp8, 2 T1, 2 T2, T3, T4, T5, T14, 2 T64, 2 CASP3_inhib, T7, T15, T8, T16, T9, T17, T13, T22, T23, T24, deg7, Syn_SCF, T25, T26, T27, T30, 2 T31, 2 T32, 2 T63, T39, T40, T10, T11, T12, T18, T19, Syn_CYLD, diss1, T21, T20, deg4, T38, diss2, T42, 2 T43, 2 T44, 2 T45, 2 T46, T49, deg1, Syn_IkB, 2 Syn_XIAP, 2 diss3, T6, 2 Syn_Procasp3 |
| 202. | 9, 15, 18, 37 | 2 Syn_BAX, Syn_SMAC, Syn_Cyt_c, Syn_Apaf1, Syn_Procasp9, T65, deg3, T66, T67, 2 Syn_TNFR1, 2 Syn_TNF, 2 Syn_TRADD, Syn_RIP1, T69, Syn_TRAF2, Syn_cIAP, T71, Syn_TAB, Syn_TAK1, 2 Syn_NEMO, T72, 2 Syn_IKK, Syn_LUBAC, Syn_NF_kB, Syn_FADD, T76, 2 Syn_Procasp8, Apo_XIAP_inhib, T75, 2 T1, 2 T2, T3, T4, T5, T14, T70, T7, T15, T8, T16, T9, T17, T13, T22, T23, T24, deg7, Syn_SCF, T25, T26, T28, T29_, T27, T30, T31, T32, T39, T40, T41, CI_diss, T10, T11, T12, deg4, T38, diss2, T42, T43, T44, T45, T46, Syn_IkB, Syn_A20, Syn_XIAP, diss3, T6, Syn_Bid |
| 203. | 7, 14, 15, 18 | 2 deg3, 2 Syn_TNFR1, 2 Syn_TNF, 2 Syn_TRADD, 2 Syn_RIP1, Syn_TRAF2, Syn_cIAP, Syn_TAB, Syn_TAK1, 2 Syn_NEMO, 2 Syn_IKK, Syn_LUBAC, Syn_NF_kB, 2 Syn_FADD, 4 Syn_Procasp8, 2 T1, 2 T2, 2 T3, T4, T5, T14, 2 T64, 2 CASP3_inhib, T7, T15, T8, T16, T9, T17, T13, T22, T23, T24, deg7, Syn_SCF, T25, T26, T27, T30, 2 T31, 2 T32, 2 T63, T39, T40, T10, T11, T12, T18, T19, Syn_CYLD, diss1, T21, T20, deg4, T38, T48, diss2, T53, T43, T54, T61, T62, T44, T45, T46, T50, deg2, T49, deg1, Syn_IkB, 2 Syn_XIAP, diss3, diss5, T6, 2 Syn_Procasp3 |
| 204. | 15, 18, 22, 37 | 2 Syn_BAX, Syn_SMAC, Syn_Cyt_c, Syn_Apaf1, Syn_Procasp9, T65, deg3, T66, T67, 2 Syn_TNFR1, 2 Syn_TNF, 2 Syn_TRADD, Syn_RIP1, T69, Syn_TRAF2, Syn_cIAP, T71, Syn_TAB, Syn_TAK1, 2 Syn_NEMO, T72, 2 Syn_IKK, Syn_LUBAC, Syn_NF_kB, 2 Syn_FADD, T76, 3 Syn_Procasp8, Apo_XIAP_inhib, Syn_RIP3, T75, 2 T1, 2 T2, T3, T4, T5, T14, T60, RIP1_RIP3_inhib, T70, T7, T15, T8, T16, T9, T17, T13, T22, T23, T24, deg7, Syn_SCF, T25, T26, T27, T30, T31, T33, T32, T34, T39, T40, T10, T11, T12, T18, T19, Syn_CYLD, diss1, T21, T20, deg4, T38, diss2, T53, T42, T43, T54, T59, Pc8_inhib, T44, T45, T46, T50, deg2, Syn_IkB, Syn_XIAP, Syn_cFLIPL, diss3, diss5, T6, Syn_Bid |
| 205. | 9, 15, 18, 38 | 2 Syn_BAX, Syn_SMAC, Syn_Cyt_c, Syn_Apaf1, Syn_Procasp9, T65, deg3, T66, T67, 2 Syn_TNFR1, 2 Syn_TNF, 2 Syn_TRADD, 2 Syn_RIP1, T69, Syn_TRAF2, Syn_cIAP, T71, Syn_TAB, Syn_TAK1, 2 Syn_NEMO, T72, 2 Syn_IKK, Syn_LUBAC, Syn_NF_kB, Syn_FADD, T76, 2 Syn_Procasp8, Apo_XIAP_inhib, T75, 2 T1, 2 T2, 2 T3, T4, T5, T14, T70, T7, T15, T8, T16, T9, T17, T13, T22, T23, T24, deg7, Syn_SCF, T25, T26, T28, T29_, T27, T30, T31, T32, T39, T40, T41, CI_diss, T10, T11, T12, deg4, T38, T48, diss2, T43, T44, T45, T46, T49, deg1, Syn_IkB, Syn_A20, Syn_XIAP, diss3, T6, Syn_Bid |
| 206. | 7, 15, 16, 18 | deg3, Syn_TNFR1, Syn_TNF, Syn_TRADD, Syn_RIP1, Syn_TRAF2, Syn_cIAP, Syn_TAB, Syn_TAK1, 2 Syn_NEMO, 2 Syn_IKK, Syn_LUBAC, Syn_NF_kB, Syn_FADD, 2 Syn_Procasp8, T1, T2, T3, T4, T5, T14, T64, CASP3_inhib, T7, T15, T8, T16, T9, T17, T13, 2 T22, T37, 2 T23, 2 T24, 2 deg7, 2 Syn_SCF, T25, 2 T26, 2 T27, T30, T31, T32, T63, T39, T40, T10, T11, T12, T18, T19, Syn_CYLD, diss1, T21, T20, deg4, T38, T43, T44, T45, T46, T49, deg1, 2 Syn_IkB, Syn_XIAP, diss3, T6, Syn_Procasp3 |
| 207. | 15, 18, 39, 41 | 3 Syn_BAX, Syn_SMAC, Syn_Cyt_c, Syn_Apaf1, Syn_Procasp9, 2 T65, 2 deg3, 2 T66, 2 T67, T68, 2 Syn_TNFR1, 2 Syn_TNF, BAX_inhib, 2 Syn_TRADD, 2 Syn_RIP1, T69, Syn_TRAF2, Syn_cIAP, T71, Syn_TAB, Syn_TAK1, 2 Syn_NEMO, T72, 2 Syn_IKK, Syn_LUBAC, Syn_NF_kB, 2 Syn_FADD, T76, 4 Syn_Procasp8, Apo_XIAP_inhib, T75, 2 T1, 2 T2, 2 T3, T4, T5, T14, T70, T7, T15, T8, T16, T9, T17, T13, T22, T23, T24, deg7, Syn_SCF, T25, T26, T27, T30, T31, T35, T32, T36, T39, T40, T10, T11, T12, T18, T19, Syn_CYLD, diss1, T21, T20, deg4, T38, T48, diss2, 2 T53, 2 T54, 2 T61, 2 T62, 2 T50, 2 deg2, Syn_IkB, Syn_XIAP, Syn_BCL_2, 2 diss5, T6, 2 Syn_Bid |
| 208. | 15, 18, 39, 42 | 3 Syn_BAX, Syn_SMAC, Syn_Cyt_c, Syn_Apaf1, Syn_Procasp9, 2 T65, 2 deg3, 2 T66, 2 T67, T68, 2 Syn_TNFR1, 2 Syn_TNF, BAX_inhib, 2 Syn_TRADD, Syn_RIP1, T69, Syn_TRAF2, Syn_cIAP, T71, Syn_TAB, Syn_TAK1, 2 Syn_NEMO, T72, 2 Syn_IKK, Syn_LUBAC, Syn_NF_kB, 2 Syn_FADD, T76, 4 Syn_Procasp8, Apo_XIAP_inhib, T75, 2 T1, 2 T2, T3, T4, T5, T14, T70, T7, T15, T8, T16, T9, T17, T13, T22, T23, T24, deg7, Syn_SCF, T25, T26, T27, T30, T31, T35, T32, T36, T39, T40, T10, T11, T12, T18, T19, Syn_CYLD, diss1, T21, T20, deg4, T38, diss2, T53, T42, T43, T54, T61, T62, T44, T45, T46, T50, deg2, Syn_IkB, Syn_XIAP, Syn_BCL_2, diss3, diss5, T6, 2 Syn_Bid |
| 209. | 9, 15, 16, 18 | Syn_TNFR1, Syn_TNF, Syn_TRADD, Syn_RIP1, Syn_TRAF2, Syn_cIAP, Syn_TAB, Syn_TAK1, 2 Syn_NEMO, 2 Syn_IKK, Syn_LUBAC, Syn_NF_kB, T1, T2, T3, T4, T5, T14, T7, T15, T8, T16, T9, T17, T13, 2 T22, T37, 2 T23, 2 T24, 2 deg7, 2 Syn_SCF, T25, 2 T26, T28, T29_, 2 T27, T30, T39, T40, T41, CI_diss, T10, T11, T12, deg4, T38, 2 Syn_IkB, Syn_A20, T6 |
| 210. | 15, 18, 32, 35 | 4 Syn_BAX, 2 Syn_SMAC, 2 Syn_Cyt_c, 2 Syn_Apaf1, 2 Syn_Procasp9, 2 T65, 2 deg3, 2 T66, 2 T67, 2 Syn_TNFR1, 2 Syn_TNF, 2 Syn_TRADD, Syn_RIP1, 2 T69, Syn_TRAF2, Syn_cIAP, 2 T71, Syn_TAB, Syn_TAK1, 2 Syn_NEMO, 2 T72, 2 Syn_IKK, Syn_LUBAC, Syn_NF_kB, 2 Syn_FADD, 4 Syn_Procasp8, 2 T75, 2 Pc9_inhib, 2 T1, 2 T2, T3, T4, T5, T14, 2 T70, T7, T15, T8, T16, T9, T17, T13, 2 deg5, T22, T23, T24, deg7, Syn_SCF, T25, T26, T27, T30, 2 T31, 2 T32, T39, T40, T10, T11, T12, T18, T19, Syn_CYLD, diss1, T21, T20, deg4, T38, diss2, T42, 2 T43, 2 T44, 2 T45, 2 T46, T49, deg1, Syn_IkB, 2 Syn_XIAP, 2 diss3, T6, 2 Syn_Bid |
| 211. | 15, 18, 22, 32 | 2 Syn_BAX, Syn_SMAC, Syn_Cyt_c, Syn_Apaf1, Syn_Procasp9, T65, deg3, T66, T67, 2 Syn_TNFR1, 2 Syn_TNF, 2 Syn_TRADD, Syn_RIP1, T69, Syn_TRAF2, Syn_cIAP, T71, Syn_TAB, Syn_TAK1, 2 Syn_NEMO, T72, 2 Syn_IKK, Syn_LUBAC, Syn_NF_kB, 2 Syn_FADD, 3 Syn_Procasp8, Syn_RIP3, T75, Pc9_inhib, 2 T1, 2 T2, T3, T4, T5, T14, T60, RIP1_RIP3_inhib, T70, T7, T15, T8, T16, T9, T17, T13, deg5, T22, T23, T24, deg7, Syn_SCF, T25, T26, T27, T30, T31, T33, T32, T34, T39, T40, T10, T11, T12, T18, T19, Syn_CYLD, diss1, T21, T20, deg4, T38, diss2, T53, T42, T43, T54, T59, Pc8_inhib, T44, T45, T46, T50, deg2, Syn_IkB, Syn_XIAP, Syn_cFLIPL, diss3, diss5, T6, Syn_Bid |
| 212. | 15, 18, 32, 34 | 4 Syn_BAX, 2 Syn_SMAC, 2 Syn_Cyt_c, 2 Syn_Apaf1, 2 Syn_Procasp9, 2 T65, 2 deg3, 2 T66, 2 T67, 2 Syn_TNFR1, 2 Syn_TNF, 2 Syn_TRADD, Syn_RIP1, 2 T69, Syn_TRAF2, Syn_cIAP, 2 T71, Syn_TAB, Syn_TAK1, 2 Syn_NEMO, 2 T72, 2 Syn_IKK, Syn_LUBAC, Syn_NF_kB, 2 Syn_FADD, 4 Syn_Procasp8, 2 T75, 2 Pc9_inhib, 2 T1, 2 T2, T3, T4, T5, T14, 2 T70, T7, T15, T8, T16, T9, T17, T13, 2 deg5, T22, T23, T24, deg7, Syn_SCF, T25, T26, T27, T30, 2 T31, 2 T32, T39, T40, T10, T11, T12, T18, T19, Syn_CYLD, diss1, T21, T20, deg4, T38, diss2, T53, T42, T43, T54, T61, T62, T44, T45, T46, T50, deg2, Syn_IkB, 2 Syn_XIAP, diss3, diss5, T6, 2 Syn_Bid |
| 213. | 15, 18, 31, 39 | 4 Syn_BAX, 2 Syn_SMAC, 2 Syn_Cyt_c, 2 Syn_Apaf1, 2 Syn_Procasp9, 2 T65, 2 deg3, 2 T66, 2 T67, 2 Syn_TNFR1, 2 Syn_TNF, 2 Syn_TRADD, 2 Syn_RIP1, 2 T69, Syn_TRAF2, Syn_cIAP, 2 T71, Syn_TAB, Syn_TAK1, 2 Syn_NEMO, 2 T72, 2 Syn_IKK, Syn_LUBAC, Syn_NF_kB, 2 Syn_FADD, T76, 4 Syn_Procasp8, Apo_XIAP_inhib, 2 T75, Pc9_inhib, 2 T1, 2 T2, 2 T3, T4, T5, T14, 2 T70, T7, T15, T8, T16, T9, T17, T13, deg5, T22, T23, T24, deg7, Syn_SCF, T25, T26, T27, T30, 2 T31, 2 T32, T39, T40, T10, T11, T12, T18, T19, Syn_CYLD, diss1, T21, T20, deg4, T38, T48, diss2, 2 T53, 2 T54, 2 T61, 2 T62, 2 T50, 2 deg2, Syn_IkB, 2 Syn_XIAP, 2 diss5, T6, 2 Syn_Bid |
| 214. | 15, 18, 22, 27 | 2 Syn_BAX, Syn_SMAC, Syn_Cyt_c, Syn_Apaf1, 2 Syn_Procasp9, T65, deg3, T66, T67, 2 Syn_TNFR1, 2 Syn_TNF, 2 Syn_TRADD, Syn_RIP1, T69, Syn_TRAF2, Syn_cIAP, T71, Syn_TAB, Syn_TAK1, 2 Syn_NEMO, T72, 2 Syn_IKK, Syn_LUBAC, T73, Syn_NF_kB, 2 Syn_FADD, 3 Syn_Procasp8, Syn_RIP3, 2 T1, 2 T2, T3, T4, T5, diss6, T14, T60, RIP1_RIP3_inhib, T64, CASP3_inhib, T70, T7, T15, T8, T16, T9, T17, T13, deg5, T22, T23, T24, deg7, Syn_SCF, T25, T26, T27, T30, T31, T33, T32, T34, deg6, T74, T39, T40, T10, T11, T12, T18, T19, Syn_CYLD, diss1, T21, T20, deg4, T38, diss2, T53, T42, T43, T54, T59, Pc8_inhib, T44, T45, T46, T50, deg2, Syn_IkB, Syn_XIAP, Syn_cFLIPL, diss3, diss5, T6, Syn_Procasp3, Syn_Bid |
| 215. | 15, 18, 31, 40 | 4 Syn_BAX, 2 Syn_SMAC, 2 Syn_Cyt_c, 2 Syn_Apaf1, 2 Syn_Procasp9, 2 T65, 2 deg3, 2 T66, 2 T67, 2 Syn_TNFR1, 2 Syn_TNF, 2 Syn_TRADD, 2 Syn_RIP1, 2 T69, Syn_TRAF2, Syn_cIAP, 2 T71, Syn_TAB, Syn_TAK1, 2 Syn_NEMO, 2 T72, 2 Syn_IKK, Syn_LUBAC, Syn_NF_kB, 2 Syn_FADD, T76, 4 Syn_Procasp8, Apo_XIAP_inhib, 2 T75, Pc9_inhib, 2 T1, 2 T2, 2 T3, T4, T5, T14, 2 T70, T7, T15, T8, T16, T9, T17, T13, deg5, T22, T23, T24, deg7, Syn_SCF, T25, T26, T27, T30, 2 T31, 2 T32, T39, T40, T10, T11, T12, T18, T19, Syn_CYLD, diss1, T21, T20, deg4, T38, T48, diss2, T53, T43, T54, T61, T62, T44, T45, T46, T50, deg2, T49, deg1, Syn_IkB, 2 Syn_XIAP, diss3, diss5, T6, 2 Syn_Bid |
| 216. | 15, 18, 38, 39 | 4 Syn_BAX, 2 Syn_SMAC, 2 Syn_Cyt_c, 2 Syn_Apaf1, 2 Syn_Procasp9, 2 T65, 2 deg3, 2 T66, 2 T67, 2 Syn_TNFR1, 2 Syn_TNF, 2 Syn_TRADD, 2 Syn_RIP1, 2 T69, Syn_TRAF2, Syn_cIAP, 2 T71, Syn_TAB, Syn_TAK1, 2 Syn_NEMO, 2 T72, 2 Syn_IKK, Syn_LUBAC, Syn_NF_kB, 2 Syn_FADD, 2 T76, 4 Syn_Procasp8, 2 Apo_XIAP_inhib, 2 T75, 2 T1, 2 T2, 2 T3, T4, T5, T14, 2 T70, T7, T15, T8, T16, T9, T17, T13, T22, T23, T24, deg7, Syn_SCF, T25, T26, T27, T30, 2 T31, 2 T32, T39, T40, T10, T11, T12, T18, T19, Syn_CYLD, diss1, T21, T20, deg4, T38, T48, diss2, T53, T43, T54, T61, T62, T44, T45, T46, T50, deg2, T49, deg1, Syn_IkB, 2 Syn_XIAP, diss3, diss5, T6, 2 Syn_Bid |
| 217. | 15, 18, 38, 40 | 4 Syn_BAX, 2 Syn_SMAC, 2 Syn_Cyt_c, 2 Syn_Apaf1, 2 Syn_Procasp9, 2 T65, 2 deg3, 2 T66, 2 T67, 2 Syn_TNFR1, 2 Syn_TNF, 2 Syn_TRADD, 2 Syn_RIP1, 2 T69, Syn_TRAF2, Syn_cIAP, 2 T71, Syn_TAB, Syn_TAK1, 2 Syn_NEMO, 2 T72, 2 Syn_IKK, Syn_LUBAC, Syn_NF_kB, 2 Syn_FADD, 2 T76, 4 Syn_Procasp8, 2 Apo_XIAP_inhib, 2 T75, 2 T1, 2 T2, 2 T3, T4, T5, T14, 2 T70, T7, T15, T8, T16, T9, T17, T13, T22, T23, T24, deg7, Syn_SCF, T25, T26, T27, T30, 2 T31, 2 T32, T39, T40, T10, T11, T12, T18, T19, Syn_CYLD, diss1, T21, T20, deg4, T38, T48, diss2, 2 T43, 2 T44, 2 T45, 2 T46, 2 T49, 2 deg1, Syn_IkB, 2 Syn_XIAP, 2 diss3, T6, 2 Syn_Bid |
| 218. | 15, 16, 18, 22 | Syn_TNFR1, Syn_TNF, Syn_TRADD, Syn_RIP1, Syn_TRAF2, Syn_cIAP, Syn_TAB, Syn_TAK1, 2 Syn_NEMO, 2 Syn_IKK, Syn_LUBAC, Syn_NF_kB, Syn_FADD, Syn_Procasp8, Syn_RIP3, T1, T2, T3, T4, T5, T14, T60, RIP1_RIP3_inhib, T7, T15, T8, T16, T9, T17, T13, 2 T22, T37, 2 T23, 2 T24, 2 deg7, 2 Syn_SCF, T25, 2 T26, 2 T27, T30, T33, T34, T39, T40, T10, T11, T12, T18, T19, Syn_CYLD, diss1, T21, T20, deg4, T38, T53, T54, T59, Pc8_inhib, T50, deg2, 2 Syn_IkB, Syn_cFLIPL, diss5, T6 |
| 219. | 8, 15, 18, 33 | 2 Syn_BAX, Syn_SMAC, Syn_Cyt_c, Syn_Apaf1, Syn_Procasp9, T65, 2 deg3, T66, T67, 2 Syn_TNFR1, 2 Syn_TNF, 2 Syn_TRADD, 2 Syn_RIP1, T69, Syn_TRAF2, Syn_cIAP, T71, Syn_TAB, Syn_TAK1, 2 Syn_NEMO, T72, 2 Syn_IKK, Syn_LUBAC, Syn_NF_kB, 2 Syn_FADD, 4 Syn_Procasp8, T75, Pc9_inhib, 2 T1, 2 T2, 2 T3, T4, T5, T14, T64, CASP3_inhib, T70, T7, T15, T8, T16, T9, T17, T13, deg5, T22, T23, T24, deg7, Syn_SCF, T25, T26, T27, T30, 2 T31, 2 T32, T63, T39, T40, T10, T11, T12, T18, T19, Syn_CYLD, diss1, T21, T20, deg4, T38, T48, diss2, T53, T43, T54, T61, T62, T44, T45, T46, T50, deg2, T49, deg1, Syn_IkB, 2 Syn_XIAP, diss3, diss5, T6, Syn_Procasp3, Syn_Bid |
| 220. | 15, 18, 31, 34 | 4 Syn_BAX, 2 Syn_SMAC, 2 Syn_Cyt_c, 2 Syn_Apaf1, 2 Syn_Procasp9, 2 T65, 2 deg3, 2 T66, 2 T67, 2 Syn_TNFR1, 2 Syn_TNF, 2 Syn_TRADD, 2 Syn_RIP1, 2 T69, Syn_TRAF2, Syn_cIAP, 2 T71, Syn_TAB, Syn_TAK1, 2 Syn_NEMO, 2 T72, 2 Syn_IKK, Syn_LUBAC, Syn_NF_kB, 2 Syn_FADD, 4 Syn_Procasp8, 2 T75, 2 Pc9_inhib, 2 T1, 2 T2, 2 T3, T4, T5, T14, 2 T70, T7, T15, T8, T16, T9, T17, T13, 2 deg5, T22, T23, T24, deg7, Syn_SCF, T25, T26, T27, T30, 2 T31, 2 T32, T39, T40, T10, T11, T12, T18, T19, Syn_CYLD, diss1, T21, T20, deg4, T38, T48, diss2, 2 T53, 2 T54, 2 T61, 2 T62, 2 T50, 2 deg2, Syn_IkB, 2 Syn_XIAP, 2 diss5, T6, 2 Syn_Bid |
| 221. | 15, 18, 25, 26 | 2 Syn_BAX, Syn_SMAC, Syn_Cyt_c, Syn_Apaf1, 2 Syn_Procasp9, T65, deg3, T66, T67, 2 Syn_TNFR1, 2 Syn_TNF, 2 Syn_TRADD, 2 Syn_RIP1, T69, Syn_TRAF2, Syn_cIAP, T71, Syn_TAB, Syn_TAK1, 2 Syn_NEMO, T72, 2 Syn_IKK, Syn_LUBAC, T73, Syn_NF_kB, Syn_FADD, 2 Syn_Procasp8, Syn_RIP3, Syn_MLKL, 2 T1, 2 T2, 2 T3, T4, T5, diss6, T14, T58, T64, CASP3_inhib, T70, T51, T7, T15, T52, T8, T16, T9, T17, T13, deg5, T22, T23, T24, deg7, Syn_SCF, T25, T26, T27, T30, T31, T32, diss4, deg6, T74, T39, T40, T10, T11, T12, T18, T19, Syn_CYLD, diss1, T21, T20, deg4, T38, T48, diss2, T53, T54, T61, T62, Necroptosis, 2 T50, 2 deg2, Syn_IkB, Syn_XIAP, diss5, T6, Syn_Procasp3, Syn_Bid |
| 222. | 11, 15, 18, 39 | 2 Syn_BAX, Syn_SMAC, Syn_Cyt_c, Syn_Apaf1, Syn_Procasp9, T65, 2 deg3, T66, T67, 2 Syn_TNFR1, 2 Syn_TNF, 2 Syn_TRADD, 2 Syn_RIP1, T69, Syn_TRAF2, Syn_cIAP, T71, Syn_TAB, Syn_TAK1, 2 Syn_NEMO, T72, 2 Syn_IKK, Syn_LUBAC, Syn_NF_kB, 2 Syn_FADD, T76, 4 Syn_Procasp8, Apo_XIAP_inhib, T75, 2 T1, 2 T2, 2 T3, T4, T5, T14, T64, CASP3_inhib, T70, T7, T15, T8, T16, T9, T17, T13, T22, T23, T24, deg7, Syn_SCF, T25, T26, T27, T30, 2 T31, 2 T32, T63, T39, T40, T10, T11, T12, T18, T19, Syn_CYLD, diss1, T21, T20, deg4, T38, T48, diss2, T53, T43, T54, T61, T62, T44, T45, T46, T50, deg2, T49, deg1, Syn_IkB, 2 Syn_XIAP, diss3, diss5, T6, Syn_Procasp3, Syn_Bid |
| 223. | 7, 15, 17, 18 | deg3, 2 Syn_TNFR1, 2 Syn_TNF, 2 Syn_TRADD, 2 Syn_RIP1, Syn_TRAF2, Syn_cIAP, Syn_TAB, Syn_TAK1, 2 Syn_NEMO, 2 Syn_IKK, Syn_LUBAC, Syn_NF_kB, 2 Syn_FADD, 3 Syn_Procasp8, 2 T1, 2 T2, 2 T3, T4, T5, T14, T64, CASP3_inhib, T7, T15, T8, T16, T9, T17, T13, T22, T23, T24, deg7, Syn_SCF, T25, T26, T27, T30, T31, T33, T32, T34, T63, T39, T40, T10, T11, T12, T18, T19, Syn_CYLD, diss1, T21, T20, deg4, T38, T48, diss2, 2 T43, 2 T44, T45, T46, T47, CIIa_inhib, 2 T49, 2 deg1, Syn_IkB, Syn_XIAP, Syn_cFLIPL, 2 diss3, T6, Syn_Procasp3 |
| 224. | 15, 18, 25, 31 | 2 Syn_BAX, Syn_SMAC, Syn_Cyt_c, Syn_Apaf1, Syn_Procasp9, T65, deg3, T66, T67, 2 Syn_TNFR1, 2 Syn_TNF, 2 Syn_TRADD, 2 Syn_RIP1, T69, Syn_TRAF2, Syn_cIAP, T71, Syn_TAB, Syn_TAK1, 2 Syn_NEMO, T72, 2 Syn_IKK, Syn_LUBAC, Syn_NF_kB, Syn_FADD, 2 Syn_Procasp8, Syn_RIP3, T75, Syn_MLKL, Pc9_inhib, 2 T1, 2 T2, 2 T3, T4, T5, T14, T58, T70, T51, T7, T15, T52, T8, T16, T9, T17, T13, deg5, T22, T23, T24, deg7, Syn_SCF, T25, T26, T27, T30, T31, T32, diss4, T39, T40, T10, T11, T12, T18, T19, Syn_CYLD, diss1, T21, T20, deg4, T38, T48, diss2, T53, T54, T61, T62, Necroptosis, 2 T50, 2 deg2, Syn_IkB, Syn_XIAP, diss5, T6, Syn_Bid |
| 225. | 15, 16, 18, 21 | 2 Syn_BAX, Syn_SMAC, Syn_Cyt_c, Syn_Apaf1, 2 Syn_Procasp9, Apoptosis, T65, deg3, T66, T67, Syn_TNFR1, Syn_TNF, Syn_TRADD, Syn_RIP1, T69, Syn_TRAF2, Syn_cIAP, T71, Syn_TAB, Syn_TAK1, 2 Syn_NEMO, T72, 2 Syn_IKK, Syn_LUBAC, T73, Syn_NF_kB, Syn_FADD, 2 Syn_Procasp8, T1, T2, T3, T4, T5, diss6, T14, T70, T7, T15, T8, T16, T9, T17, T13, deg5, 2 T22, T37, 2 T23, 2 T24, 2 deg7, 2 Syn_SCF, T25, 2 T26, 2 T27, T30, deg6, T74, T39, T40, T10, T11, T12, T18, T19, Syn_CYLD, diss1, T21, T20, deg4, T38, T43, T44, T45, T46, T49, deg1, 2 Syn_IkB, diss3, T6, Syn_Procasp3, Syn_Bid |
| 226. | 15, 18, 32, 44 | 3 Syn_BAX, Syn_SMAC, Syn_Cyt_c, Syn_Apaf1, Syn_Procasp9, 2 T65, 2 deg3, 2 T66, 2 T67, T68, 2 Syn_TNFR1, 2 Syn_TNF, BAX_inhib, 2 Syn_TRADD, Syn_RIP1, T69, Syn_TRAF2, Syn_cIAP, T71, Syn_TAB, Syn_TAK1, 2 Syn_NEMO, T72, 2 Syn_IKK, Syn_LUBAC, Syn_NF_kB, 2 Syn_FADD, 4 Syn_Procasp8, T75, Pc9_inhib, 2 T1, 2 T2, T3, T4, T5, T14, T70, T7, T15, T8, T16, T9, T17, T13, deg5, T22, T23, T24, deg7, Syn_SCF, T25, T26, T27, T30, T31, T35, T32, T36, T39, T40, T10, T11, T12, T18, T19, Syn_CYLD, diss1, T21, T20, deg4, T38, diss2, T53, T42, T43, T54, T61, T62, T44, T45, T46, T50, deg2, Syn_IkB, Syn_XIAP, Syn_BCL_2, diss3, diss5, T6, 2 Syn_Bid |
| 227. | 15, 18, 25, 28 | 2 Syn_BAX, Syn_SMAC, Syn_Cyt_c, Syn_Apaf1, 2 Syn_Procasp9, T65, deg3, T66, T67, 2 Syn_TNFR1, 2 Syn_TNF, 2 Syn_TRADD, 2 Syn_RIP1, T69, Syn_TRAF2, Syn_cIAP, T71, Syn_TAB, Syn_TAK1, 2 Syn_NEMO, T72, 2 Syn_IKK, Syn_LUBAC, T73, Syn_NF_kB, Syn_FADD, 2 Syn_Procasp8, Syn_RIP3, Syn_MLKL, 2 T1, 2 T2, 2 T3, T4, T5, diss6, T14, T58, T64, CASP3_inhib, T70, T51, T7, T15, T52, T8, T16, T9, T17, T13, deg5, T22, T23, T24, deg7, Syn_SCF, T25, T26, T27, T30, T31, T32, diss4, deg6, T74, T39, T40, T10, T11, T12, T18, T19, Syn_CYLD, diss1, T21, T20, deg4, T38, T48, diss2, T43, T44, T45, Necroptosis, T46, T50, deg2, T49, deg1, Syn_IkB, Syn_XIAP, diss3, T6, Syn_Procasp3, Syn_Bid |
| 228. | 9, 15, 18, 31 | 2 Syn_BAX, Syn_SMAC, Syn_Cyt_c, Syn_Apaf1, Syn_Procasp9, T65, deg3, T66, T67, 2 Syn_TNFR1, 2 Syn_TNF, 2 Syn_TRADD, 2 Syn_RIP1, T69, Syn_TRAF2, Syn_cIAP, T71, Syn_TAB, Syn_TAK1, 2 Syn_NEMO, T72, 2 Syn_IKK, Syn_LUBAC, Syn_NF_kB, Syn_FADD, 2 Syn_Procasp8, T75, Pc9_inhib, 2 T1, 2 T2, 2 T3, T4, T5, T14, T70, T7, T15, T8, T16, T9, T17, T13, deg5, T22, T23, T24, deg7, Syn_SCF, T25, T26, T28, T29_, T27, T30, T31, T32, T39, T40, T41, CI_diss, T10, T11, T12, deg4, T38, T48, diss2, T53, T54, T61, T62, T50, deg2, Syn_IkB, Syn_A20, Syn_XIAP, diss5, T6, Syn_Bid |
| 229. | 15, 18, 32, 45 | 3 Syn_BAX, Syn_SMAC, Syn_Cyt_c, Syn_Apaf1, Syn_Procasp9, 2 T65, 2 deg3, 2 T66, 2 T67, T68, 2 Syn_TNFR1, 2 Syn_TNF, BAX_inhib, 2 Syn_TRADD, Syn_RIP1, T69, Syn_TRAF2, Syn_cIAP, T71, Syn_TAB, Syn_TAK1, 2 Syn_NEMO, T72, 2 Syn_IKK, Syn_LUBAC, Syn_NF_kB, 2 Syn_FADD, 4 Syn_Procasp8, T75, Pc9_inhib, 2 T1, 2 T2, T3, T4, T5, T14, T70, T7, T15, T8, T16, T9, T17, T13, deg5, T22, T23, T24, deg7, Syn_SCF, T25, T26, T27, T30, T31, T35, T32, T36, T39, T40, T10, T11, T12, T18, T19, Syn_CYLD, diss1, T21, T20, deg4, T38, diss2, T42, 2 T43, 2 T44, 2 T45, 2 T46, T49, deg1, Syn_IkB, Syn_XIAP, Syn_BCL_2, 2 diss3, T6, 2 Syn_Bid |
| 230. | 15, 16, 18, 44 | Syn_BAX, T65, deg3, T66, T67, T68, Syn_TNFR1, Syn_TNF, BAX_inhib, Syn_TRADD, Syn_RIP1, Syn_TRAF2, Syn_cIAP, Syn_TAB, Syn_TAK1, 2 Syn_NEMO, 2 Syn_IKK, Syn_LUBAC, Syn_NF_kB, Syn_FADD, 2 Syn_Procasp8, T1, T2, T3, T4, T5, T14, T7, T15, T8, T16, T9, T17, T13, 2 T22, T37, 2 T23, 2 T24, 2 deg7, 2 Syn_SCF, T25, 2 T26, 2 T27, T30, T35, T36, T39, T40, T10, T11, T12, T18, T19, Syn_CYLD, diss1, T21, T20, deg4, T38, T53, T54, T61, T62, T50, deg2, 2 Syn_IkB, Syn_BCL_2, diss5, T6, Syn_Bid |
| 231. | 11, 15, 18, 40 | 2 Syn_BAX, Syn_SMAC, Syn_Cyt_c, Syn_Apaf1, Syn_Procasp9, T65, 2 deg3, T66, T67, 2 Syn_TNFR1, 2 Syn_TNF, 2 Syn_TRADD, 2 Syn_RIP1, T69, Syn_TRAF2, Syn_cIAP, T71, Syn_TAB, Syn_TAK1, 2 Syn_NEMO, T72, 2 Syn_IKK, Syn_LUBAC, Syn_NF_kB, 2 Syn_FADD, T76, 4 Syn_Procasp8, Apo_XIAP_inhib, T75, 2 T1, 2 T2, 2 T3, T4, T5, T14, T64, CASP3_inhib, T70, T7, T15, T8, T16, T9, T17, T13, T22, T23, T24, deg7, Syn_SCF, T25, T26, T27, T30, 2 T31, 2 T32, T63, T39, T40, T10, T11, T12, T18, T19, Syn_CYLD, diss1, T21, T20, deg4, T38, T48, diss2, 2 T43, 2 T44, 2 T45, 2 T46, 2 T49, 2 deg1, Syn_IkB, 2 Syn_XIAP, 2 diss3, T6, Syn_Procasp3, Syn_Bid |
| 232. | 15, 16, 18, 20 | Syn_TNFR1, Syn_TNF, Syn_TRADD, Syn_RIP1, Syn_TRAF2, Syn_cIAP, Syn_TAB, Syn_TAK1, 2 Syn_NEMO, 2 Syn_IKK, Syn_LUBAC, Syn_NF_kB, Syn_FADD, Syn_Procasp8, Syn_cFLIPs, Syn_RIP3, Syn_MLKL, T1, T2, T3, T4, T5, T14, T58, T7, T15, T8, T16, T9, T17, T13, 2 T22, T37, 2 T23, 2 T24, 2 deg7, 2 Syn_SCF, T25, 2 T26, 2 T27, T30, T39, T40, T10, T11, T12, T18, T19, Syn_CYLD, diss1, T21, T20, deg4, T38, T53, T54, T55, T56, T57, Necroptosis, T50, deg2, 2 Syn_IkB, diss5, T6 |
| 233. | 11, 15, 18, 45 | Syn_BAX, T65, 2 deg3, T66, T67, T68, 2 Syn_TNFR1, 2 Syn_TNF, BAX_inhib, 2 Syn_TRADD, 2 Syn_RIP1, Syn_TRAF2, Syn_cIAP, Syn_TAB, Syn_TAK1, 2 Syn_NEMO, 2 Syn_IKK, Syn_LUBAC, Syn_NF_kB, 2 Syn_FADD, 4 Syn_Procasp8, 2 T1, 2 T2, 2 T3, T4, T5, T14, T64, CASP3_inhib, T7, T15, T8, T16, T9, T17, T13, T22, T23, T24, deg7, Syn_SCF, T25, T26, T27, T30, T31, T35, T32, T36, T63, T39, T40, T10, T11, T12, T18, T19, Syn_CYLD, diss1, T21, T20, deg4, T38, T48, diss2, 2 T43, 2 T44, 2 T45, 2 T46, 2 T49, 2 deg1, Syn_IkB, Syn_XIAP, Syn_BCL_2, 2 diss3, T6, Syn_Procasp3, Syn_Bid |
| 234. | 9, 15, 18, 26 | 2 Syn_BAX, Syn_SMAC, Syn_Cyt_c, Syn_Apaf1, 2 Syn_Procasp9, T65, deg3, T66, T67, 2 Syn_TNFR1, 2 Syn_TNF, 2 Syn_TRADD, 2 Syn_RIP1, T69, Syn_TRAF2, Syn_cIAP, T71, Syn_TAB, Syn_TAK1, 2 Syn_NEMO, T72, 2 Syn_IKK, Syn_LUBAC, T73, Syn_NF_kB, Syn_FADD, 2 Syn_Procasp8, 2 T1, 2 T2, 2 T3, T4, T5, diss6, T14, T64, CASP3_inhib, T70, T7, T15, T8, T16, T9, T17, T13, deg5, T22, T23, T24, deg7, Syn_SCF, T25, T26, T28, T29_, T27, T30, T31, T32, deg6, T74, T39, T40, T41, CI_diss, T10, T11, T12, deg4, T38, T48, diss2, T53, T54, T61, T62, T50, deg2, Syn_IkB, Syn_A20, Syn_XIAP, diss5, T6, Syn_Procasp3, Syn_Bid |
| 235. | 15, 18, 33, 40 | 4 Syn_BAX, 2 Syn_SMAC, 2 Syn_Cyt_c, 2 Syn_Apaf1, 2 Syn_Procasp9, 2 T65, 2 deg3, 2 T66, 2 T67, 2 Syn_TNFR1, 2 Syn_TNF, 2 Syn_TRADD, 2 Syn_RIP1, 2 T69, Syn_TRAF2, Syn_cIAP, 2 T71, Syn_TAB, Syn_TAK1, 2 Syn_NEMO, 2 T72, 2 Syn_IKK, Syn_LUBAC, Syn_NF_kB, 2 Syn_FADD, T76, 4 Syn_Procasp8, Apo_XIAP_inhib, 2 T75, Pc9_inhib, 2 T1, 2 T2, 2 T3, T4, T5, T14, 2 T70, T7, T15, T8, T16, T9, T17, T13, deg5, T22, T23, T24, deg7, Syn_SCF, T25, T26, T27, T30, 2 T31, 2 T32, T39, T40, T10, T11, T12, T18, T19, Syn_CYLD, diss1, T21, T20, deg4, T38, T48, diss2, 2 T43, 2 T44, 2 T45, 2 T46, 2 T49, 2 deg1, Syn_IkB, 2 Syn_XIAP, 2 diss3, T6, 2 Syn_Bid |
| 236. | 15, 16, 18, 30 | 2 Syn_BAX, Syn_SMAC, Syn_Cyt_c, Syn_Apaf1, 2 Syn_Procasp9, T65, deg3, T66, T67, Syn_TNFR1, Syn_TNF, Syn_TRADD, Syn_RIP1, T69, Syn_TRAF2, Syn_cIAP, T71, Syn_TAB, Syn_TAK1, 2 Syn_NEMO, T72, 2 Syn_IKK, Syn_LUBAC, T73, Syn_NF_kB, Syn_FADD, 2 Syn_Procasp8, T1, T2, T3, T4, T5, diss6, T14, T64, CASP3_inhib, T70, T7, T15, T8, T16, T9, T17, T13, deg5, 2 T22, T37, 2 T23, 2 T24, 2 deg7, 2 Syn_SCF, T25, 2 T26, 2 T27, T30, T31, T32, deg6, T74, T39, T40, T10, T11, T12, T18, T19, Syn_CYLD, diss1, T21, T20, deg4, T38, T43, T44, T45, T46, T49, deg1, 2 Syn_IkB, Syn_XIAP, diss3, T6, Syn_Procasp3, Syn_Bid |
| 237. | 15, 18, 25, 33 | 2 Syn_BAX, Syn_SMAC, Syn_Cyt_c, Syn_Apaf1, Syn_Procasp9, T65, deg3, T66, T67, 2 Syn_TNFR1, 2 Syn_TNF, 2 Syn_TRADD, 2 Syn_RIP1, T69, Syn_TRAF2, Syn_cIAP, T71, Syn_TAB, Syn_TAK1, 2 Syn_NEMO, T72, 2 Syn_IKK, Syn_LUBAC, Syn_NF_kB, Syn_FADD, 2 Syn_Procasp8, Syn_RIP3, T75, Syn_MLKL, Pc9_inhib, 2 T1, 2 T2, 2 T3, T4, T5, T14, T58, T70, T51, T7, T15, T52, T8, T16, T9, T17, T13, deg5, T22, T23, T24, deg7, Syn_SCF, T25, T26, T27, T30, T31, T32, diss4, T39, T40, T10, T11, T12, T18, T19, Syn_CYLD, diss1, T21, T20, deg4, T38, T48, diss2, T43, T44, T45, Necroptosis, T46, T50, deg2, T49, deg1, Syn_IkB, Syn_XIAP, diss3, T6, Syn_Bid |
| 238. | 15, 18, 33, 35 | 4 Syn_BAX, 2 Syn_SMAC, 2 Syn_Cyt_c, 2 Syn_Apaf1, 2 Syn_Procasp9, 2 T65, 2 deg3, 2 T66, 2 T67, 2 Syn_TNFR1, 2 Syn_TNF, 2 Syn_TRADD, 2 Syn_RIP1, 2 T69, Syn_TRAF2, Syn_cIAP, 2 T71, Syn_TAB, Syn_TAK1, 2 Syn_NEMO, 2 T72, 2 Syn_IKK, Syn_LUBAC, Syn_NF_kB, 2 Syn_FADD, 4 Syn_Procasp8, 2 T75, 2 Pc9_inhib, 2 T1, 2 T2, 2 T3, T4, T5, T14, 2 T70, T7, T15, T8, T16, T9, T17, T13, 2 deg5, T22, T23, T24, deg7, Syn_SCF, T25, T26, T27, T30, 2 T31, 2 T32, T39, T40, T10, T11, T12, T18, T19, Syn_CYLD, diss1, T21, T20, deg4, T38, T48, diss2, 2 T43, 2 T44, 2 T45, 2 T46, 2 T49, 2 deg1, Syn_IkB, 2 Syn_XIAP, 2 diss3, T6, 2 Syn_Bid |
| 239. | 9, 15, 18, 28 | 2 Syn_BAX, Syn_SMAC, Syn_Cyt_c, Syn_Apaf1, 2 Syn_Procasp9, T65, deg3, T66, T67, 2 Syn_TNFR1, 2 Syn_TNF, 2 Syn_TRADD, 2 Syn_RIP1, T69, Syn_TRAF2, Syn_cIAP, T71, Syn_TAB, Syn_TAK1, 2 Syn_NEMO, T72, 2 Syn_IKK, Syn_LUBAC, T73, Syn_NF_kB, Syn_FADD, 2 Syn_Procasp8, 2 T1, 2 T2, 2 T3, T4, T5, diss6, T14, T64, CASP3_inhib, T70, T7, T15, T8, T16, T9, T17, T13, deg5, T22, T23, T24, deg7, Syn_SCF, T25, T26, T28, T29_, T27, T30, T31, T32, deg6, T74, T39, T40, T41, CI_diss, T10, T11, T12, deg4, T38, T48, diss2, T43, T44, T45, T46, T49, deg1, Syn_IkB, Syn_A20, Syn_XIAP, diss3, T6, Syn_Procasp3, Syn_Bid |
| 240. | 9, 15, 18, 32 | 2 Syn_BAX, Syn_SMAC, Syn_Cyt_c, Syn_Apaf1, Syn_Procasp9, T65, deg3, T66, T67, 2 Syn_TNFR1, 2 Syn_TNF, 2 Syn_TRADD, Syn_RIP1, T69, Syn_TRAF2, Syn_cIAP, T71, Syn_TAB, Syn_TAK1, 2 Syn_NEMO, T72, 2 Syn_IKK, Syn_LUBAC, Syn_NF_kB, Syn_FADD, 2 Syn_Procasp8, T75, Pc9_inhib, 2 T1, 2 T2, T3, T4, T5, T14, T70, T7, T15, T8, T16, T9, T17, T13, deg5, T22, T23, T24, deg7, Syn_SCF, T25, T26, T28, T29_, T27, T30, T31, T32, T39, T40, T41, CI_diss, T10, T11, T12, deg4, T38, diss2, T42, T43, T44, T45, T46, Syn_IkB, Syn_A20, Syn_XIAP, diss3, T6, Syn_Bid |
| 241. | 15, 18, 37, 39 | 4 Syn_BAX, 2 Syn_SMAC, 2 Syn_Cyt_c, 2 Syn_Apaf1, 2 Syn_Procasp9, 2 T65, 2 deg3, 2 T66, 2 T67, 2 Syn_TNFR1, 2 Syn_TNF, 2 Syn_TRADD, Syn_RIP1, 2 T69, Syn_TRAF2, Syn_cIAP, 2 T71, Syn_TAB, Syn_TAK1, 2 Syn_NEMO, 2 T72, 2 Syn_IKK, Syn_LUBAC, Syn_NF_kB, 2 Syn_FADD, 2 T76, 4 Syn_Procasp8, 2 Apo_XIAP_inhib, 2 T75, 2 T1, 2 T2, T3, T4, T5, T14, 2 T70, T7, T15, T8, T16, T9, T17, T13, T22, T23, T24, deg7, Syn_SCF, T25, T26, T27, T30, 2 T31, 2 T32, T39, T40, T10, T11, T12, T18, T19, Syn_CYLD, diss1, T21, T20, deg4, T38, diss2, T53, T42, T43, T54, T61, T62, T44, T45, T46, T50, deg2, Syn_IkB, 2 Syn_XIAP, diss3, diss5, T6, 2 Syn_Bid |
| 242. | 15, 18, 24, 37 | 4 Syn_BAX, 2 Syn_SMAC, 2 Syn_Cyt_c, 2 Syn_Apaf1, 3 Syn_Procasp9, Apoptosis, 2 T65, 2 deg3, 2 T66, 2 T67, 2 Syn_TNFR1, 2 Syn_TNF, 2 Syn_TRADD, Syn_RIP1, 2 T69, Syn_TRAF2, Syn_cIAP, 2 T71, Syn_TAB, Syn_TAK1, 2 Syn_NEMO, 2 T72, 2 Syn_IKK, Syn_LUBAC, T73, Syn_NF_kB, 2 Syn_FADD, T76, 4 Syn_Procasp8, Apo_XIAP_inhib, T75, 2 T1, 2 T2, T3, T4, T5, diss6, T14, 2 T70, T7, T15, T8, T16, T9, T17, T13, deg5, T22, T23, T24, deg7, Syn_SCF, T25, T26, T27, T30, T31, T32, deg6, T74, T39, T40, T10, T11, T12, T18, T19, Syn_CYLD, diss1, T21, T20, deg4, T38, diss2, T53, T42, T43, T54, T61, T62, T44, T45, T46, T50, deg2, Syn_IkB, Syn_XIAP, diss3, diss5, T6, Syn_Procasp3, 2 Syn_Bid |
| 243. | 7, 15, 18, 33 | 2 Syn_BAX, Syn_SMAC, Syn_Cyt_c, Syn_Apaf1, Syn_Procasp9, T65, 2 deg3, T66, T67, 2 Syn_TNFR1, 2 Syn_TNF, 2 Syn_TRADD, 2 Syn_RIP1, T69, Syn_TRAF2, Syn_cIAP, T71, Syn_TAB, Syn_TAK1, 2 Syn_NEMO, T72, 2 Syn_IKK, Syn_LUBAC, Syn_NF_kB, 2 Syn_FADD, 4 Syn_Procasp8, T75, Pc9_inhib, 2 T1, 2 T2, 2 T3, T4, T5, T14, T64, CASP3_inhib, T70, T7, T15, T8, T16, T9, T17, T13, deg5, T22, T23, T24, deg7, Syn_SCF, T25, T26, T27, T30, 2 T31, 2 T32, T63, T39, T40, T10, T11, T12, T18, T19, Syn_CYLD, diss1, T21, T20, deg4, T38, T48, diss2, 2 T43, 2 T44, 2 T45, 2 T46, 2 T49, 2 deg1, Syn_IkB, 2 Syn_XIAP, 2 diss3, T6, Syn_Procasp3, Syn_Bid |
| 244. | 15, 18, 25, 43 | Syn_BAX, T65, deg3, T66, T67, T68, 2 Syn_TNFR1, 2 Syn_TNF, BAX_inhib, 2 Syn_TRADD, 2 Syn_RIP1, Syn_TRAF2, Syn_cIAP, Syn_TAB, Syn_TAK1, 2 Syn_NEMO, 2 Syn_IKK, Syn_LUBAC, Syn_NF_kB, Syn_FADD, 2 Syn_Procasp8, Syn_RIP3, Syn_MLKL, 2 T1, 2 T2, 2 T3, T4, T5, T14, T58, T51, T7, T15, T52, T8, T16, T9, T17, T13, T22, T23, T24, deg7, Syn_SCF, T25, T26, T27, T30, T35, T36, diss4, T39, T40, T10, T11, T12, T18, T19, Syn_CYLD, diss1, T21, T20, deg4, T38, T48, diss2, T43, T44, T45, Necroptosis, T46, T50, deg2, T49, deg1, Syn_IkB, Syn_BCL_2, diss3, T6, Syn_Bid |
| 245. | 9, 15, 18, 43 | Syn_BAX, T65, deg3, T66, T67, T68, 2 Syn_TNFR1, 2 Syn_TNF, BAX_inhib, 2 Syn_TRADD, 2 Syn_RIP1, Syn_TRAF2, Syn_cIAP, Syn_TAB, Syn_TAK1, 2 Syn_NEMO, 2 Syn_IKK, Syn_LUBAC, Syn_NF_kB, Syn_FADD, 2 Syn_Procasp8, 2 T1, 2 T2, 2 T3, T4, T5, T14, T7, T15, T8, T16, T9, T17, T13, T22, T23, T24, deg7, Syn_SCF, T25, T26, T28, T29_, T27, T30, T35, T36, T39, T40, T41, CI_diss, T10, T11, T12, deg4, T38, T48, diss2, T43, T44, T45, T46, T49, deg1, Syn_IkB, Syn_A20, Syn_BCL_2, diss3, T6, Syn_Bid |
| 246. | 7, 15, 18, 28 | 2 Syn_BAX, Syn_SMAC, Syn_Cyt_c, Syn_Apaf1, 2 Syn_Procasp9, T65, 2 deg3, T66, T67, 2 Syn_TNFR1, 2 Syn_TNF, 2 Syn_TRADD, 2 Syn_RIP1, T69, Syn_TRAF2, Syn_cIAP, T71, Syn_TAB, Syn_TAK1, 2 Syn_NEMO, T72, 2 Syn_IKK, Syn_LUBAC, T73, Syn_NF_kB, 2 Syn_FADD, 4 Syn_Procasp8, 2 T1, 2 T2, 2 T3, T4, T5, diss6, T14, 2 T64, 2 CASP3_inhib, T70, T7, T15, T8, T16, T9, T17, T13, deg5, T22, T23, T24, deg7, Syn_SCF, T25, T26, T27, T30, 2 T31, 2 T32, deg6, T63, T74, T39, T40, T10, T11, T12, T18, T19, Syn_CYLD, diss1, T21, T20, deg4, T38, T48, diss2, 2 T43, 2 T44, 2 T45, 2 T46, 2 T49, 2 deg1, Syn_IkB, 2 Syn_XIAP, 2 diss3, T6, 2 Syn_Procasp3, Syn_Bid |
| 247. | 15, 18, 24, 27 | 4 Syn_BAX, 2 Syn_SMAC, 2 Syn_Cyt_c, 2 Syn_Apaf1, 4 Syn_Procasp9, Apoptosis, 2 T65, 2 deg3, 2 T66, 2 T67, 2 Syn_TNFR1, 2 Syn_TNF, 2 Syn_TRADD, Syn_RIP1, 2 T69, Syn_TRAF2, Syn_cIAP, 2 T71, Syn_TAB, Syn_TAK1, 2 Syn_NEMO, 2 T72, 2 Syn_IKK, Syn_LUBAC, 2 T73, Syn_NF_kB, 2 Syn_FADD, 4 Syn_Procasp8, 2 T1, 2 T2, T3, T4, T5, 2 diss6, T14, T64, CASP3_inhib, 2 T70, T7, T15, T8, T16, T9, T17, T13, 2 deg5, T22, T23, T24, deg7, Syn_SCF, T25, T26, T27, T30, T31, T32, 2 deg6, 2 T74, T39, T40, T10, T11, T12, T18, T19, Syn_CYLD, diss1, T21, T20, deg4, T38, diss2, T53, T42, T43, T54, T61, T62, T44, T45, T46, T50, deg2, Syn_IkB, Syn_XIAP, diss3, diss5, T6, 2 Syn_Procasp3, 2 Syn_Bid |
| 248. | 15, 16, 18, 24 | 2 Syn_BAX, Syn_SMAC, Syn_Cyt_c, Syn_Apaf1, 2 Syn_Procasp9, Apoptosis, T65, deg3, T66, T67, Syn_TNFR1, Syn_TNF, Syn_TRADD, Syn_RIP1, T69, Syn_TRAF2, Syn_cIAP, T71, Syn_TAB, Syn_TAK1, 2 Syn_NEMO, T72, 2 Syn_IKK, Syn_LUBAC, T73, Syn_NF_kB, Syn_FADD, 2 Syn_Procasp8, T1, T2, T3, T4, T5, diss6, T14, T70, T7, T15, T8, T16, T9, T17, T13, deg5, 2 T22, T37, 2 T23, 2 T24, 2 deg7, 2 Syn_SCF, T25, 2 T26, 2 T27, T30, deg6, T74, T39, T40, T10, T11, T12, T18, T19, Syn_CYLD, diss1, T21, T20, deg4, T38, T53, T54, T61, T62, T50, deg2, 2 Syn_IkB, diss5, T6, Syn_Procasp3, Syn_Bid |
| 249. | 7, 15, 18, 19 | deg3, 2 Syn_TNFR1, 2 Syn_TNF, 2 Syn_TRADD, 2 Syn_RIP1, Syn_TRAF2, Syn_cIAP, Syn_TAB, Syn_TAK1, 2 Syn_NEMO, 2 Syn_IKK, Syn_LUBAC, Syn_NF_kB, 2 Syn_FADD, 3 Syn_Procasp8, Syn_RIP3, 2 T1, 2 T2, 2 T3, T4, T5, T14, T60, RIP1_RIP3_inhib, T64, CASP3_inhib, T7, T15, T8, T16, T9, T17, T13, T22, T23, T24, deg7, Syn_SCF, T25, T26, T27, T30, T31, T33, T32, T34, T63, T39, T40, T10, T11, T12, T18, T19, Syn_CYLD, diss1, T21, T20, deg4, T38, T48, diss2, T53, T43, T54, T59, Pc8_inhib, T44, T45, T46, T50, deg2, T49, deg1, Syn_IkB, Syn_XIAP, Syn_cFLIPL, diss3, diss5, T6, Syn_Procasp3 |
| 250. | 15, 18, 37, 40 | 4 Syn_BAX, 2 Syn_SMAC, 2 Syn_Cyt_c, 2 Syn_Apaf1, 2 Syn_Procasp9, 2 T65, 2 deg3, 2 T66, 2 T67, 2 Syn_TNFR1, 2 Syn_TNF, 2 Syn_TRADD, Syn_RIP1, 2 T69, Syn_TRAF2, Syn_cIAP, 2 T71, Syn_TAB, Syn_TAK1, 2 Syn_NEMO, 2 T72, 2 Syn_IKK, Syn_LUBAC, Syn_NF_kB, 2 Syn_FADD, 2 T76, 4 Syn_Procasp8, 2 Apo_XIAP_inhib, 2 T75, 2 T1, 2 T2, T3, T4, T5, T14, 2 T70, T7, T15, T8, T16, T9, T17, T13, T22, T23, T24, deg7, Syn_SCF, T25, T26, T27, T30, 2 T31, 2 T32, T39, T40, T10, T11, T12, T18, T19, Syn_CYLD, diss1, T21, T20, deg4, T38, diss2, T42, 2 T43, 2 T44, 2 T45, 2 T46, T49, deg1, Syn_IkB, 2 Syn_XIAP, 2 diss3, T6, 2 Syn_Bid |
| 251. | 9, 15, 18, 33 | 2 Syn_BAX, Syn_SMAC, Syn_Cyt_c, Syn_Apaf1, Syn_Procasp9, T65, deg3, T66, T67, 2 Syn_TNFR1, 2 Syn_TNF, 2 Syn_TRADD, 2 Syn_RIP1, T69, Syn_TRAF2, Syn_cIAP, T71, Syn_TAB, Syn_TAK1, 2 Syn_NEMO, T72, 2 Syn_IKK, Syn_LUBAC, Syn_NF_kB, Syn_FADD, 2 Syn_Procasp8, T75, Pc9_inhib, 2 T1, 2 T2, 2 T3, T4, T5, T14, T70, T7, T15, T8, T16, T9, T17, T13, deg5, T22, T23, T24, deg7, Syn_SCF, T25, T26, T28, T29_, T27, T30, T31, T32, T39, T40, T41, CI_diss, T10, T11, T12, deg4, T38, T48, diss2, T43, T44, T45, T46, T49, deg1, Syn_IkB, Syn_A20, Syn_XIAP, diss3, T6, Syn_Bid |
| 252. | 15, 18, 24, 26 | 4 Syn_BAX, 2 Syn_SMAC, 2 Syn_Cyt_c, 2 Syn_Apaf1, 4 Syn_Procasp9, Apoptosis, 2 T65, 2 deg3, 2 T66, 2 T67, 2 Syn_TNFR1, 2 Syn_TNF, 2 Syn_TRADD, 2 Syn_RIP1, 2 T69, Syn_TRAF2, Syn_cIAP, 2 T71, Syn_TAB, Syn_TAK1, 2 Syn_NEMO, 2 T72, 2 Syn_IKK, Syn_LUBAC, 2 T73, Syn_NF_kB, 2 Syn_FADD, 4 Syn_Procasp8, 2 T1, 2 T2, 2 T3, T4, T5, 2 diss6, T14, T64, CASP3_inhib, 2 T70, T7, T15, T8, T16, T9, T17, T13, 2 deg5, T22, T23, T24, deg7, Syn_SCF, T25, T26, T27, T30, T31, T32, 2 deg6, 2 T74, T39, T40, T10, T11, T12, T18, T19, Syn_CYLD, diss1, T21, T20, deg4, T38, T48, diss2, 2 T53, 2 T54, 2 T61, 2 T62, 2 T50, 2 deg2, Syn_IkB, Syn_XIAP, 2 diss5, T6, 2 Syn_Procasp3, 2 Syn_Bid |
| 253. | 9, 15, 18, 27 | 2 Syn_BAX, Syn_SMAC, Syn_Cyt_c, Syn_Apaf1, 2 Syn_Procasp9, T65, deg3, T66, T67, 2 Syn_TNFR1, 2 Syn_TNF, 2 Syn_TRADD, Syn_RIP1, T69, Syn_TRAF2, Syn_cIAP, T71, Syn_TAB, Syn_TAK1, 2 Syn_NEMO, T72, 2 Syn_IKK, Syn_LUBAC, T73, Syn_NF_kB, Syn_FADD, 2 Syn_Procasp8, 2 T1, 2 T2, T3, T4, T5, diss6, T14, T64, CASP3_inhib, T70, T7, T15, T8, T16, T9, T17, T13, deg5, T22, T23, T24, deg7, Syn_SCF, T25, T26, T28, T29_, T27, T30, T31, T32, deg6, T74, T39, T40, T41, CI_diss, T10, T11, T12, deg4, T38, diss2, T42, T43, T44, T45, T46, Syn_IkB, Syn_A20, Syn_XIAP, diss3, T6, Syn_Procasp3, Syn_Bid |
| 254. | 15, 18, 29, 31 | 4 Syn_BAX, 2 Syn_SMAC, 2 Syn_Cyt_c, 2 Syn_Apaf1, 3 Syn_Procasp9, 2 T65, 2 deg3, 2 T66, 2 T67, 2 Syn_TNFR1, 2 Syn_TNF, 2 Syn_TRADD, 2 Syn_RIP1, 2 T69, Syn_TRAF2, Syn_cIAP, 2 T71, Syn_TAB, Syn_TAK1, 2 Syn_NEMO, 2 T72, 2 Syn_IKK, Syn_LUBAC, T73, Syn_NF_kB, 2 Syn_FADD, 4 Syn_Procasp8, T75, Pc9_inhib, 2 T1, 2 T2, 2 T3, T4, T5, diss6, T14, T64, CASP3_inhib, 2 T70, T7, T15, T8, T16, T9, T17, T13, 2 deg5, T22, T23, T24, deg7, Syn_SCF, T25, T26, T27, T30, 2 T31, 2 T32, deg6, T74, T39, T40, T10, T11, T12, T18, T19, Syn_CYLD, diss1, T21, T20, deg4, T38, T48, diss2, 2 T53, 2 T54, 2 T61, 2 T62, 2 T50, 2 deg2, Syn_IkB, 2 Syn_XIAP, 2 diss5, T6, Syn_Procasp3, 2 Syn_Bid |
| 255. | 15, 18, 24, 36 | 4 Syn_BAX, 2 Syn_SMAC, 2 Syn_Cyt_c, 2 Syn_Apaf1, 3 Syn_Procasp9, Apoptosis, 2 T65, 2 deg3, 2 T66, 2 T67, 2 Syn_TNFR1, 2 Syn_TNF, 2 Syn_TRADD, 2 Syn_RIP1, 2 T69, Syn_TRAF2, Syn_cIAP, 2 T71, Syn_TAB, Syn_TAK1, 2 Syn_NEMO, 2 T72, 2 Syn_IKK, Syn_LUBAC, T73, Syn_NF_kB, 2 Syn_FADD, T76, 4 Syn_Procasp8, Apo_XIAP_inhib, T75, 2 T1, 2 T2, 2 T3, T4, T5, diss6, T14, 2 T70, T7, T15, T8, T16, T9, T17, T13, deg5, T22, T23, T24, deg7, Syn_SCF, T25, T26, T27, T30, T31, T32, deg6, T74, T39, T40, T10, T11, T12, T18, T19, Syn_CYLD, diss1, T21, T20, deg4, T38, T48, diss2, 2 T53, 2 T54, 2 T61, 2 T62, 2 T50, 2 deg2, Syn_IkB, Syn_XIAP, 2 diss5, T6, Syn_Procasp3, 2 Syn_Bid |
| 256. | 15, 18, 25, 32 | 2 Syn_BAX, Syn_SMAC, Syn_Cyt_c, Syn_Apaf1, Syn_Procasp9, T65, deg3, T66, T67, 2 Syn_TNFR1, 2 Syn_TNF, 2 Syn_TRADD, Syn_RIP1, T69, Syn_TRAF2, Syn_cIAP, T71, Syn_TAB, Syn_TAK1, 2 Syn_NEMO, T72, 2 Syn_IKK, Syn_LUBAC, Syn_NF_kB, Syn_FADD, 2 Syn_Procasp8, Syn_RIP3, T75, Syn_MLKL, Pc9_inhib, 2 T1, 2 T2, T3, T4, T5, T14, T58, T70, T51, T7, T15, T52, T8, T16, T9, T17, T13, deg5, T22, T23, T24, deg7, Syn_SCF, T25, T26, T27, T30, T31, T32, diss4, T39, T40, T10, T11, T12, T18, T19, Syn_CYLD, diss1, T21, T20, deg4, T38, diss2, T42, T43, T44, T45, Necroptosis, T46, T50, deg2, Syn_IkB, Syn_XIAP, diss3, T6, Syn_Bid |
| 257. | 15, 18, 36, 39 | 4 Syn_BAX, 2 Syn_SMAC, 2 Syn_Cyt_c, 2 Syn_Apaf1, 2 Syn_Procasp9, 2 T65, 2 deg3, 2 T66, 2 T67, 2 Syn_TNFR1, 2 Syn_TNF, 2 Syn_TRADD, 2 Syn_RIP1, 2 T69, Syn_TRAF2, Syn_cIAP, 2 T71, Syn_TAB, Syn_TAK1, 2 Syn_NEMO, 2 T72, 2 Syn_IKK, Syn_LUBAC, Syn_NF_kB, 2 Syn_FADD, 2 T76, 4 Syn_Procasp8, 2 Apo_XIAP_inhib, 2 T75, 2 T1, 2 T2, 2 T3, T4, T5, T14, 2 T70, T7, T15, T8, T16, T9, T17, T13, T22, T23, T24, deg7, Syn_SCF, T25, T26, T27, T30, 2 T31, 2 T32, T39, T40, T10, T11, T12, T18, T19, Syn_CYLD, diss1, T21, T20, deg4, T38, T48, diss2, 2 T53, 2 T54, 2 T61, 2 T62, 2 T50, 2 deg2, Syn_IkB, 2 Syn_XIAP, 2 diss5, T6, 2 Syn_Bid |
| 258. | 15, 18, 24, 32 | 4 Syn_BAX, 2 Syn_SMAC, 2 Syn_Cyt_c, 2 Syn_Apaf1, 3 Syn_Procasp9, Apoptosis, 2 T65, 2 deg3, 2 T66, 2 T67, 2 Syn_TNFR1, 2 Syn_TNF, 2 Syn_TRADD, Syn_RIP1, 2 T69, Syn_TRAF2, Syn_cIAP, 2 T71, Syn_TAB, Syn_TAK1, 2 Syn_NEMO, 2 T72, 2 Syn_IKK, Syn_LUBAC, T73, Syn_NF_kB, 2 Syn_FADD, 4 Syn_Procasp8, T75, Pc9_inhib, 2 T1, 2 T2, T3, T4, T5, diss6, T14, 2 T70, T7, T15, T8, T16, T9, T17, T13, 2 deg5, T22, T23, T24, deg7, Syn_SCF, T25, T26, T27, T30, T31, T32, deg6, T74, T39, T40, T10, T11, T12, T18, T19, Syn_CYLD, diss1, T21, T20, deg4, T38, diss2, T53, T42, T43, T54, T61, T62, T44, T45, T46, T50, deg2, Syn_IkB, Syn_XIAP, diss3, diss5, T6, Syn_Procasp3, 2 Syn_Bid |
| 259. | 15, 18, 25, 42 | Syn_BAX, T65, deg3, T66, T67, T68, 2 Syn_TNFR1, 2 Syn_TNF, BAX_inhib, 2 Syn_TRADD, Syn_RIP1, Syn_TRAF2, Syn_cIAP, Syn_TAB, Syn_TAK1, 2 Syn_NEMO, 2 Syn_IKK, Syn_LUBAC, Syn_NF_kB, Syn_FADD, 2 Syn_Procasp8, Syn_RIP3, Syn_MLKL, 2 T1, 2 T2, T3, T4, T5, T14, T58, T51, T7, T15, T52, T8, T16, T9, T17, T13, T22, T23, T24, deg7, Syn_SCF, T25, T26, T27, T30, T35, T36, diss4, T39, T40, T10, T11, T12, T18, T19, Syn_CYLD, diss1, T21, T20, deg4, T38, diss2, T42, T43, T44, T45, Necroptosis, T46, T50, deg2, Syn_IkB, Syn_BCL_2, diss3, T6, Syn_Bid |
| 260. | 15, 16, 18, 34 | 2 Syn_BAX, Syn_SMAC, Syn_Cyt_c, Syn_Apaf1, Syn_Procasp9, T65, deg3, T66, T67, Syn_TNFR1, Syn_TNF, Syn_TRADD, Syn_RIP1, T69, Syn_TRAF2, Syn_cIAP, T71, Syn_TAB, Syn_TAK1, 2 Syn_NEMO, T72, 2 Syn_IKK, Syn_LUBAC, Syn_NF_kB, Syn_FADD, 2 Syn_Procasp8, T75, Pc9_inhib, T1, T2, T3, T4, T5, T14, T70, T7, T15, T8, T16, T9, T17, T13, deg5, 2 T22, T37, 2 T23, 2 T24, 2 deg7, 2 Syn_SCF, T25, 2 T26, 2 T27, T30, T31, T32, T39, T40, T10, T11, T12, T18, T19, Syn_CYLD, diss1, T21, T20, deg4, T38, T53, T54, T61, T62, T50, deg2, 2 Syn_IkB, Syn_XIAP, diss5, T6, Syn_Bid |
| 261. | 15, 18, 33, 45 | 3 Syn_BAX, Syn_SMAC, Syn_Cyt_c, Syn_Apaf1, Syn_Procasp9, 2 T65, 2 deg3, 2 T66, 2 T67, T68, 2 Syn_TNFR1, 2 Syn_TNF, BAX_inhib, 2 Syn_TRADD, 2 Syn_RIP1, T69, Syn_TRAF2, Syn_cIAP, T71, Syn_TAB, Syn_TAK1, 2 Syn_NEMO, T72, 2 Syn_IKK, Syn_LUBAC, Syn_NF_kB, 2 Syn_FADD, 4 Syn_Procasp8, T75, Pc9_inhib, 2 T1, 2 T2, 2 T3, T4, T5, T14, T70, T7, T15, T8, T16, T9, T17, T13, deg5, T22, T23, T24, deg7, Syn_SCF, T25, T26, T27, T30, T31, T35, T32, T36, T39, T40, T10, T11, T12, T18, T19, Syn_CYLD, diss1, T21, T20, deg4, T38, T48, diss2, 2 T43, 2 T44, 2 T45, 2 T46, 2 T49, 2 deg1, Syn_IkB, Syn_XIAP, Syn_BCL_2, 2 diss3, T6, 2 Syn_Bid |
| 262. | 9, 14, 15, 18 | deg3, 2 Syn_TNFR1, 2 Syn_TNF, 2 Syn_TRADD, 2 Syn_RIP1, Syn_TRAF2, Syn_cIAP, Syn_TAB, Syn_TAK1, 2 Syn_NEMO, 2 Syn_IKK, Syn_LUBAC, Syn_NF_kB, Syn_FADD, 2 Syn_Procasp8, 2 T1, 2 T2, 2 T3, T4, T5, T14, T64, CASP3_inhib, T7, T15, T8, T16, T9, T17, T13, T22, T23, T24, deg7, Syn_SCF, T25, T26, T28, T29_, T27, T30, T31, T32, T63, T39, T40, T41, CI_diss, T10, T11, T12, deg4, T38, T48, diss2, T53, T54, T61, T62, T50, deg2, Syn_IkB, Syn_A20, Syn_XIAP, diss5, T6, Syn_Procasp3 |
| 263. | 15, 16, 18, 39 | 2 Syn_BAX, Syn_SMAC, Syn_Cyt_c, Syn_Apaf1, Syn_Procasp9, T65, deg3, T66, T67, Syn_TNFR1, Syn_TNF, Syn_TRADD, Syn_RIP1, T69, Syn_TRAF2, Syn_cIAP, T71, Syn_TAB, Syn_TAK1, 2 Syn_NEMO, T72, 2 Syn_IKK, Syn_LUBAC, Syn_NF_kB, Syn_FADD, T76, 2 Syn_Procasp8, Apo_XIAP_inhib, T75, T1, T2, T3, T4, T5, T14, T70, T7, T15, T8, T16, T9, T17, T13, 2 T22, T37, 2 T23, 2 T24, 2 deg7, 2 Syn_SCF, T25, 2 T26, 2 T27, T30, T31, T32, T39, T40, T10, T11, T12, T18, T19, Syn_CYLD, diss1, T21, T20, deg4, T38, T53, T54, T61, T62, T50, deg2, 2 Syn_IkB, Syn_XIAP, diss5, T6, Syn_Bid |
| 264. | 15, 18, 25, 36 | 2 Syn_BAX, Syn_SMAC, Syn_Cyt_c, Syn_Apaf1, Syn_Procasp9, T65, deg3, T66, T67, 2 Syn_TNFR1, 2 Syn_TNF, 2 Syn_TRADD, 2 Syn_RIP1, T69, Syn_TRAF2, Syn_cIAP, T71, Syn_TAB, Syn_TAK1, 2 Syn_NEMO, T72, 2 Syn_IKK, Syn_LUBAC, Syn_NF_kB, Syn_FADD, T76, 2 Syn_Procasp8, Apo_XIAP_inhib, Syn_RIP3, T75, Syn_MLKL, 2 T1, 2 T2, 2 T3, T4, T5, T14, T58, T70, T51, T7, T15, T52, T8, T16, T9, T17, T13, T22, T23, T24, deg7, Syn_SCF, T25, T26, T27, T30, T31, T32, diss4, T39, T40, T10, T11, T12, T18, T19, Syn_CYLD, diss1, T21, T20, deg4, T38, T48, diss2, T53, T54, T61, T62, Necroptosis, 2 T50, 2 deg2, Syn_IkB, Syn_XIAP, diss5, T6, Syn_Bid |
| 265. | 9, 15, 18, 42 | Syn_BAX, T65, deg3, T66, T67, T68, 2 Syn_TNFR1, 2 Syn_TNF, BAX_inhib, 2 Syn_TRADD, Syn_RIP1, Syn_TRAF2, Syn_cIAP, Syn_TAB, Syn_TAK1, 2 Syn_NEMO, 2 Syn_IKK, Syn_LUBAC, Syn_NF_kB, Syn_FADD, 2 Syn_Procasp8, 2 T1, 2 T2, T3, T4, T5, T14, T7, T15, T8, T16, T9, T17, T13, T22, T23, T24, deg7, Syn_SCF, T25, T26, T28, T29_, T27, T30, T35, T36, T39, T40, T41, CI_diss, T10, T11, T12, deg4, T38, diss2, T42, T43, T44, T45, T46, Syn_IkB, Syn_A20, Syn_BCL_2, diss3, T6, Syn_Bid |
| 266. | 8, 12, 15, 18 | 2 deg3, 2 Syn_TNFR1, 2 Syn_TNF, 2 Syn_TRADD, Syn_RIP1, Syn_TRAF2, Syn_cIAP, Syn_TAB, Syn_TAK1, 2 Syn_NEMO, 2 Syn_IKK, Syn_LUBAC, Syn_NF_kB, 2 Syn_FADD, 4 Syn_Procasp8, 2 T1, 2 T2, T3, T4, T5, T14, 2 T64, 2 CASP3_inhib, T7, T15, T8, T16, T9, T17, T13, T22, T23, T24, deg7, Syn_SCF, T25, T26, T27, T30, 2 T31, 2 T32, 2 T63, T39, T40, T10, T11, T12, T18, T19, Syn_CYLD, diss1, T21, T20, deg4, T38, diss2, T53, T42, T43, T54, T61, T62, T44, T45, T46, T50, deg2, Syn_IkB, 2 Syn_XIAP, diss3, diss5, T6, 2 Syn_Procasp3 |
| 267. | 8, 14, 15, 18 | 2 deg3, 2 Syn_TNFR1, 2 Syn_TNF, 2 Syn_TRADD, 2 Syn_RIP1, Syn_TRAF2, Syn_cIAP, Syn_TAB, Syn_TAK1, 2 Syn_NEMO, 2 Syn_IKK, Syn_LUBAC, Syn_NF_kB, 2 Syn_FADD, 4 Syn_Procasp8, 2 T1, 2 T2, 2 T3, T4, T5, T14, 2 T64, 2 CASP3_inhib, T7, T15, T8, T16, T9, T17, T13, T22, T23, T24, deg7, Syn_SCF, T25, T26, T27, T30, 2 T31, 2 T32, 2 T63, T39, T40, T10, T11, T12, T18, T19, Syn_CYLD, diss1, T21, T20, deg4, T38, T48, diss2, 2 T53, 2 T54, 2 T61, 2 T62, 2 T50, 2 deg2, Syn_IkB, 2 Syn_XIAP, 2 diss5, T6, 2 Syn_Procasp3 |
| 268. | 15, 18, 34, 37 | 4 Syn_BAX, 2 Syn_SMAC, 2 Syn_Cyt_c, 2 Syn_Apaf1, 2 Syn_Procasp9, 2 T65, 2 deg3, 2 T66, 2 T67, 2 Syn_TNFR1, 2 Syn_TNF, 2 Syn_TRADD, Syn_RIP1, 2 T69, Syn_TRAF2, Syn_cIAP, 2 T71, Syn_TAB, Syn_TAK1, 2 Syn_NEMO, 2 T72, 2 Syn_IKK, Syn_LUBAC, Syn_NF_kB, 2 Syn_FADD, T76, 4 Syn_Procasp8, Apo_XIAP_inhib, 2 T75, Pc9_inhib, 2 T1, 2 T2, T3, T4, T5, T14, 2 T70, T7, T15, T8, T16, T9, T17, T13, deg5, T22, T23, T24, deg7, Syn_SCF, T25, T26, T27, T30, 2 T31, 2 T32, T39, T40, T10, T11, T12, T18, T19, Syn_CYLD, diss1, T21, T20, deg4, T38, diss2, T53, T42, T43, T54, T61, T62, T44, T45, T46, T50, deg2, Syn_IkB, 2 Syn_XIAP, diss3, diss5, T6, 2 Syn_Bid |
| 269. | 9, 15, 18, 41 | Syn_BAX, T65, deg3, T66, T67, T68, 2 Syn_TNFR1, 2 Syn_TNF, BAX_inhib, 2 Syn_TRADD, 2 Syn_RIP1, Syn_TRAF2, Syn_cIAP, Syn_TAB, Syn_TAK1, 2 Syn_NEMO, 2 Syn_IKK, Syn_LUBAC, Syn_NF_kB, Syn_FADD, 2 Syn_Procasp8, 2 T1, 2 T2, 2 T3, T4, T5, T14, T7, T15, T8, T16, T9, T17, T13, T22, T23, T24, deg7, Syn_SCF, T25, T26, T28, T29_, T27, T30, T35, T36, T39, T40, T41, CI_diss, T10, T11, T12, deg4, T38, T48, diss2, T53, T54, T61, T62, T50, deg2, Syn_IkB, Syn_A20, Syn_BCL_2, diss5, T6, Syn_Bid |
| 270. | 8, 15, 16, 18 | deg3, Syn_TNFR1, Syn_TNF, Syn_TRADD, Syn_RIP1, Syn_TRAF2, Syn_cIAP, Syn_TAB, Syn_TAK1, 2 Syn_NEMO, 2 Syn_IKK, Syn_LUBAC, Syn_NF_kB, Syn_FADD, 2 Syn_Procasp8, T1, T2, T3, T4, T5, T14, T64, CASP3_inhib, T7, T15, T8, T16, T9, T17, T13, 2 T22, T37, 2 T23, 2 T24, 2 deg7, 2 Syn_SCF, T25, 2 T26, 2 T27, T30, T31, T32, T63, T39, T40, T10, T11, T12, T18, T19, Syn_CYLD, diss1, T21, T20, deg4, T38, T53, T54, T61, T62, T50, deg2, 2 Syn_IkB, Syn_XIAP, diss5, T6, Syn_Procasp3 |
| 271. | 15, 16, 18, 23 | Syn_TNFR1, Syn_TNF, Syn_TRADD, Syn_RIP1, Syn_TRAF2, Syn_cIAP, Syn_TAB, Syn_TAK1, 2 Syn_NEMO, 2 Syn_IKK, Syn_LUBAC, Syn_NF_kB, Syn_FADD, Syn_Procasp8, T1, T2, T3, T4, T5, T14, T7, T15, T8, T16, T9, T17, T13, 2 T22, T37, 2 T23, 2 T24, 2 deg7, 2 Syn_SCF, T25, 2 T26, 2 T27, T30, T33, T34, T39, T40, T10, T11, T12, T18, T19, Syn_CYLD, diss1, T21, T20, deg4, T38, T43, T44, T47, CIIa_inhib, T49, deg1, 2 Syn_IkB, Syn_cFLIPL, diss3, T6 |
| 272. | 11, 15, 18, 25 | deg3, 2 Syn_TNFR1, 2 Syn_TNF, 2 Syn_TRADD, 2 Syn_RIP1, Syn_TRAF2, Syn_cIAP, Syn_TAB, Syn_TAK1, 2 Syn_NEMO, 2 Syn_IKK, Syn_LUBAC, Syn_NF_kB, Syn_FADD, 2 Syn_Procasp8, Syn_RIP3, Syn_MLKL, 2 T1, 2 T2, 2 T3, T4, T5, T14, T58, T64, CASP3_inhib, T51, T7, T15, T52, T8, T16, T9, T17, T13, T22, T23, T24, deg7, Syn_SCF, T25, T26, T27, T30, T31, T32, diss4, T63, T39, T40, T10, T11, T12, T18, T19, Syn_CYLD, diss1, T21, T20, deg4, T38, T48, diss2, T43, T44, T45, Necroptosis, T46, T50, deg2, T49, deg1, Syn_IkB, Syn_XIAP, diss3, T6, Syn_Procasp3 |
| 273. | 15, 16, 18, 25 | Syn_TNFR1, Syn_TNF, Syn_TRADD, Syn_RIP1, Syn_TRAF2, Syn_cIAP, Syn_TAB, Syn_TAK1, 2 Syn_NEMO, 2 Syn_IKK, Syn_LUBAC, Syn_NF_kB, Syn_RIP3, Syn_MLKL, T1, T2, T3, T4, T5, T14, T58, T51, T7, T15, T52, T8, T16, T9, T17, T13, 2 T22, T37, 2 T23, 2 T24, 2 deg7, 2 Syn_SCF, T25, 2 T26, 2 T27, T30, diss4, T39, T40, T10, T11, T12, T18, T19, Syn_CYLD, diss1, T21, T20, deg4, T38, Necroptosis, T50, deg2, 2 Syn_IkB, T6 |
| 274. | 15, 18, 24, 42 | 3 Syn_BAX, Syn_SMAC, Syn_Cyt_c, Syn_Apaf1, 2 Syn_Procasp9, Apoptosis, 2 T65, 2 deg3, 2 T66, 2 T67, T68, 2 Syn_TNFR1, 2 Syn_TNF, BAX_inhib, 2 Syn_TRADD, Syn_RIP1, T69, Syn_TRAF2, Syn_cIAP, T71, Syn_TAB, Syn_TAK1, 2 Syn_NEMO, T72, 2 Syn_IKK, Syn_LUBAC, T73, Syn_NF_kB, 2 Syn_FADD, 4 Syn_Procasp8, 2 T1, 2 T2, T3, T4, T5, diss6, T14, T70, T7, T15, T8, T16, T9, T17, T13, deg5, T22, T23, T24, deg7, Syn_SCF, T25, T26, T27, T30, T35, T36, deg6, T74, T39, T40, T10, T11, T12, T18, T19, Syn_CYLD, diss1, T21, T20, deg4, T38, diss2, T53, T42, T43, T54, T61, T62, T44, T45, T46, T50, deg2, Syn_IkB, Syn_BCL_2, diss3, diss5, T6, Syn_Procasp3, 2 Syn_Bid |
| 275. | 9, 12, 15, 18 | deg3, 2 Syn_TNFR1, 2 Syn_TNF, 2 Syn_TRADD, Syn_RIP1, Syn_TRAF2, Syn_cIAP, Syn_TAB, Syn_TAK1, 2 Syn_NEMO, 2 Syn_IKK, Syn_LUBAC, Syn_NF_kB, Syn_FADD, 2 Syn_Procasp8, 2 T1, 2 T2, T3, T4, T5, T14, T64, CASP3_inhib, T7, T15, T8, T16, T9, T17, T13, T22, T23, T24, deg7, Syn_SCF, T25, T26, T28, T29_, T27, T30, T31, T32, T63, T39, T40, T41, CI_diss, T10, T11, T12, deg4, T38, diss2, T42, T43, T44, T45, T46, Syn_IkB, Syn_A20, Syn_XIAP, diss3, T6, Syn_Procasp3 |
| 276. | 15, 18, 24, 41 | 3 Syn_BAX, Syn_SMAC, Syn_Cyt_c, Syn_Apaf1, 2 Syn_Procasp9, Apoptosis, 2 T65, 2 deg3, 2 T66, 2 T67, T68, 2 Syn_TNFR1, 2 Syn_TNF, BAX_inhib, 2 Syn_TRADD, 2 Syn_RIP1, T69, Syn_TRAF2, Syn_cIAP, T71, Syn_TAB, Syn_TAK1, 2 Syn_NEMO, T72, 2 Syn_IKK, Syn_LUBAC, T73, Syn_NF_kB, 2 Syn_FADD, 4 Syn_Procasp8, 2 T1, 2 T2, 2 T3, T4, T5, diss6, T14, T70, T7, T15, T8, T16, T9, T17, T13, deg5, T22, T23, T24, deg7, Syn_SCF, T25, T26, T27, T30, T35, T36, deg6, T74, T39, T40, T10, T11, T12, T18, T19, Syn_CYLD, diss1, T21, T20, deg4, T38, T48, diss2, 2 T53, 2 T54, 2 T61, 2 T62, 2 T50, 2 deg2, Syn_IkB, Syn_BCL_2, 2 diss5, T6, Syn_Procasp3, 2 Syn_Bid |
| 277. | 11, 15, 18, 20 | deg3, 2 Syn_TNFR1, 2 Syn_TNF, 2 Syn_TRADD, 2 Syn_RIP1, Syn_TRAF2, Syn_cIAP, Syn_TAB, Syn_TAK1, 2 Syn_NEMO, 2 Syn_IKK, Syn_LUBAC, Syn_NF_kB, 2 Syn_FADD, 3 Syn_Procasp8, Syn_cFLIPs, Syn_RIP3, Syn_MLKL, 2 T1, 2 T2, 2 T3, T4, T5, T14, T58, T64, CASP3_inhib, T7, T15, T8, T16, T9, T17, T13, T22, T23, T24, deg7, Syn_SCF, T25, T26, T27, T30, T31, T32, T63, T39, T40, T10, T11, T12, T18, T19, Syn_CYLD, diss1, T21, T20, deg4, T38, T48, diss2, T53, T43, T54, T44, T45, T55, T56, T57, Necroptosis, T46, T50, deg2, T49, deg1, Syn_IkB, Syn_XIAP, diss3, diss5, T6, Syn_Procasp3 |
| 278. | 9, 11, 15, 18 | deg3, 2 Syn_TNFR1, 2 Syn_TNF, 2 Syn_TRADD, 2 Syn_RIP1, Syn_TRAF2, Syn_cIAP, Syn_TAB, Syn_TAK1, 2 Syn_NEMO, 2 Syn_IKK, Syn_LUBAC, Syn_NF_kB, Syn_FADD, 2 Syn_Procasp8, 2 T1, 2 T2, 2 T3, T4, T5, T14, T64, CASP3_inhib, T7, T15, T8, T16, T9, T17, T13, T22, T23, T24, deg7, Syn_SCF, T25, T26, T28, T29_, T27, T30, T31, T32, T63, T39, T40, T41, CI_diss, T10, T11, T12, deg4, T38, T48, diss2, T43, T44, T45, T46, T49, deg1, Syn_IkB, Syn_A20, Syn_XIAP, diss3, T6, Syn_Procasp3 |
| 279. | 15, 18, 24, 31 | 4 Syn_BAX, 2 Syn_SMAC, 2 Syn_Cyt_c, 2 Syn_Apaf1, 3 Syn_Procasp9, Apoptosis, 2 T65, 2 deg3, 2 T66, 2 T67, 2 Syn_TNFR1, 2 Syn_TNF, 2 Syn_TRADD, 2 Syn_RIP1, 2 T69, Syn_TRAF2, Syn_cIAP, 2 T71, Syn_TAB, Syn_TAK1, 2 Syn_NEMO, 2 T72, 2 Syn_IKK, Syn_LUBAC, T73, Syn_NF_kB, 2 Syn_FADD, 4 Syn_Procasp8, T75, Pc9_inhib, 2 T1, 2 T2, 2 T3, T4, T5, diss6, T14, 2 T70, T7, T15, T8, T16, T9, T17, T13, 2 deg5, T22, T23, T24, deg7, Syn_SCF, T25, T26, T27, T30, T31, T32, deg6, T74, T39, T40, T10, T11, T12, T18, T19, Syn_CYLD, diss1, T21, T20, deg4, T38, T48, diss2, 2 T53, 2 T54, 2 T61, 2 T62, 2 T50, 2 deg2, Syn_IkB, Syn_XIAP, 2 diss5, T6, Syn_Procasp3, 2 Syn_Bid |
