# Supplementary material for: Mathematical modeling of the molecular switch of TNFR1-mediated signaling pathways applying Petri net formalism and in silico knockout analysis
Source: PLoS Comput Biol. 2022 Aug 22;18(8):e1010383. doi: 10.1371/journal.pcbi.1010383 (PMC9467317; doi:10.1371/journal.pcbi.1010383)
Supplement: S7 Table — The list interprets the in silico knockout matrix of Fig 10. For each row of the matrix, the table lists the knocked out protein(s), the number of affected proteins or complexes, and descriptions of affected processes and of related processes. Knockouts with identical patterns of red circles are merged. (DOCX) [file pcbi.1010383.s008.docx]

**S7 Table:** Effects of *in silico* knockouts of specific proteins. The list interprets the *in silico* knockout matrix of Fig 10. For each row of the matrix, the table lists the knocked out protein(s), the number of affected proteins or complexes, and descriptions of affected processes and of related processes. Knockouts with identical patterns of red circles are merged.

| Knocked out entity  [row no.; #affected(red) entries] | Affected processes | Related functional aspects |
| --- | --- | --- |
| BAX  [1; 6] | complex formation of BCL-2 and BAX (BCL-2:BAX); activation of CASP9 in the apoptosome (CASP9, Apoptosome) | Activation of CASP3 and CASP8 are not directly dependent on BAX (CASP3, CASP8). |
| cFLIPS  [2; 1] | complex Formation of cFLIPS bound to complex IIb (CIIb:cFLIPs) | cFLIPS can promote necroptosis induction in complex IIb, but as other pathways exist that can also induce necroptosis, the knockout of cFLIPS has no direct effect on necroptosis induction. |
| cIAP1/2 and TRAF2  [3, 20; 10] | formation of complex I and the NF-κB-dependent gene expression as well as the feedback and crosstalk regulation of the target genes | This emphasizes the direct regulation of both proteins since cIAP1/2 requires TRAF2 for recruitment. |
| CYLD  [4; 1] | complex formation of CYLD bound to the complex I (CI:CYLD) | CYLD promotes the dissociation of complex I and the formation of complex II. As several pathways also cover the processes, no other effects are observed. |
| FADD, procaspase 8  [5, 12; 14] | all places associated to apoptosis processes | Only the survival pathways and necroptosis induction is still functional. This emphasizes the direct regulation of both proteins since procaspase 8 requires FADD for recruitment. |
| IKK, NEMO, TAK1, LUBAC  [6, 7, 9, 16; 9] | the downstream activation of NF-κB and the regulation of the target genes | Complex II formation and cell death induction remain functional. This indicates the strong relation of the proteins in the Ub-dependent regulation in complex I. |
| MLKL  [8; 1] | activated MLKL located at the plasma membrane prior necroptosis induction (MLKL_PM) | As activated MLKL refers to the last step in the necroptosis pathway, necroptosis induction is hampered. |
| NF-κB  [10; 8] | NF-κB regulation via IκB and the regulation of NF-κB-dependent genes |  |
| procaspase 3  [11; 2] | CASP3 activation and CASP3 inhibition by XIAP (CASP3, XIAP:CASP3) |  |
| procaspase 9  [13; 4] | processes of the regulation of procaspase 9 in the apoptosome via XIAP and SMAC |  |
| RIP1  [14; 14] | formation of complex I and the induction of necroptosis | Only apoptosis processes are still functioning since RIP1 is a major player in the TNFR1 signal transduction pathway. |
| RIP3  [15; 2] | formation of the necrosome and the activation of MLKL (RIP1:RIP3, MLKL_PM) |  |
| TNF, TNFR1, TRADD  [17, 18, 19; 20] | all places except for the nuclear NF-κB (NF-kB_n). Since the three proteins initialize the pathway, all downstream pathway components are affected by the knockouts. | This is due to the modeling of the turnover of NF-κB, which remains unaffected by the knockout. |
| cIAP1/2 and XIAP (Smac mimetic)  [20; 10] | formation of complex I, NF-κB-dependent gene expression, and XIAP regulation | Only apoptosis and necroptosis induction remain functional. |
| IκB, A20, XIAP, cFLIP_L_, and BCL-2 (cycloheximide)  [21; 7] | the translation of upregulated genes | Only the cell death pathways remain unaffected. |
