# Supplementary figures and images for: Mathematical modeling of the molecular switch of TNFR1-mediated signaling pathways applying Petri net formalism and in silico knockout analysis
Source: PLoS Comput Biol. 2022 Aug 22;18(8):e1010383. doi: 10.1371/journal.pcbi.1010383 (PMC9467317; doi:10.1371/journal.pcbi.1010383)

**
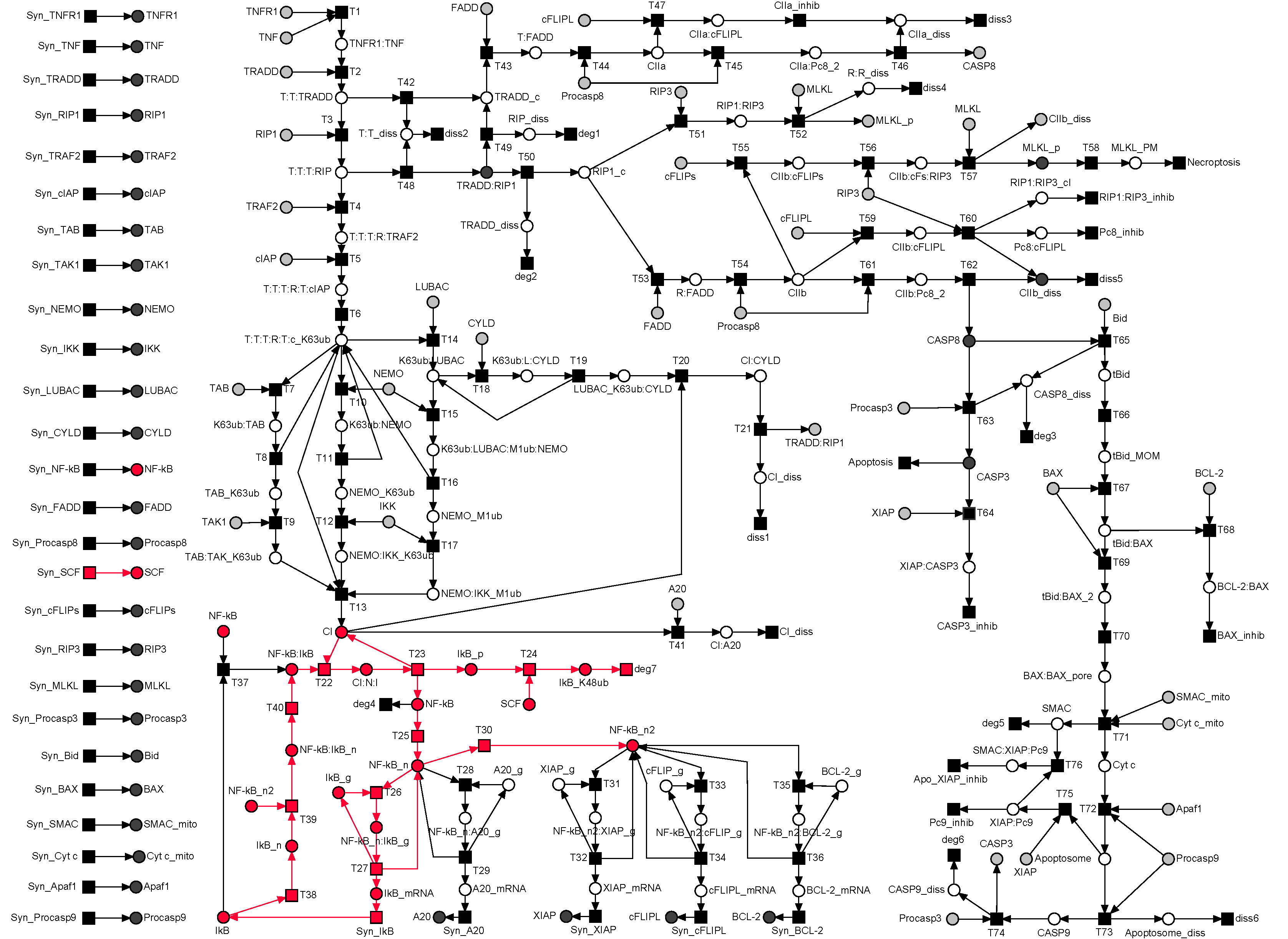
**

**S1 Fig:** Exemplary transition invariant, TI15.

Supplement: S1 Fig — TI15 highlighted in red describes the activation of NF-κB, the degradation of IκB, the gene expression of IκB, and the interaction of complex I with the inhibitory complex of NF-κB and I κB. The pathway relies on a former production of complex I, place CI. The assembly process of complex I is not part of TI15 and hence, TI15 represents an incomplete pathway. (DOCX) [file pcbi.1010383.s009.docx]

**
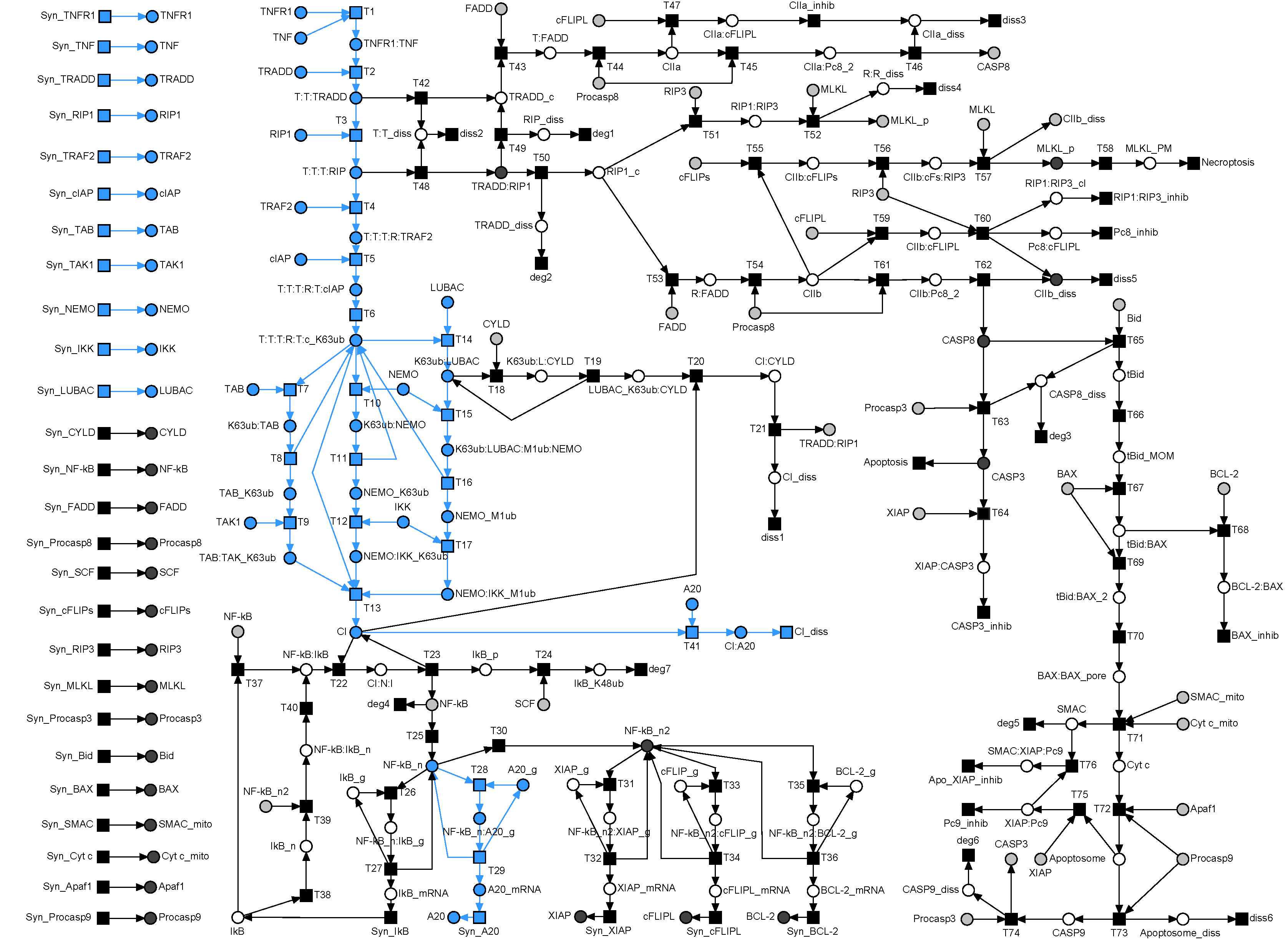
**

**S2 Fig:** Exemplary transition invariant, TI9.

Supplement: S2 Fig — TI9 highlighted in blue describes the assembly of complex I and the dissociation of complex I via A20. The transcription of A20 relies on a former translocation of NF-κB into the nucleus. The translocation of NF-κB into the nucleus is not part of TI9 and hence, TI9 represents an incomplete pathway. (DOCX) [file pcbi.1010383.s010.docx]

**
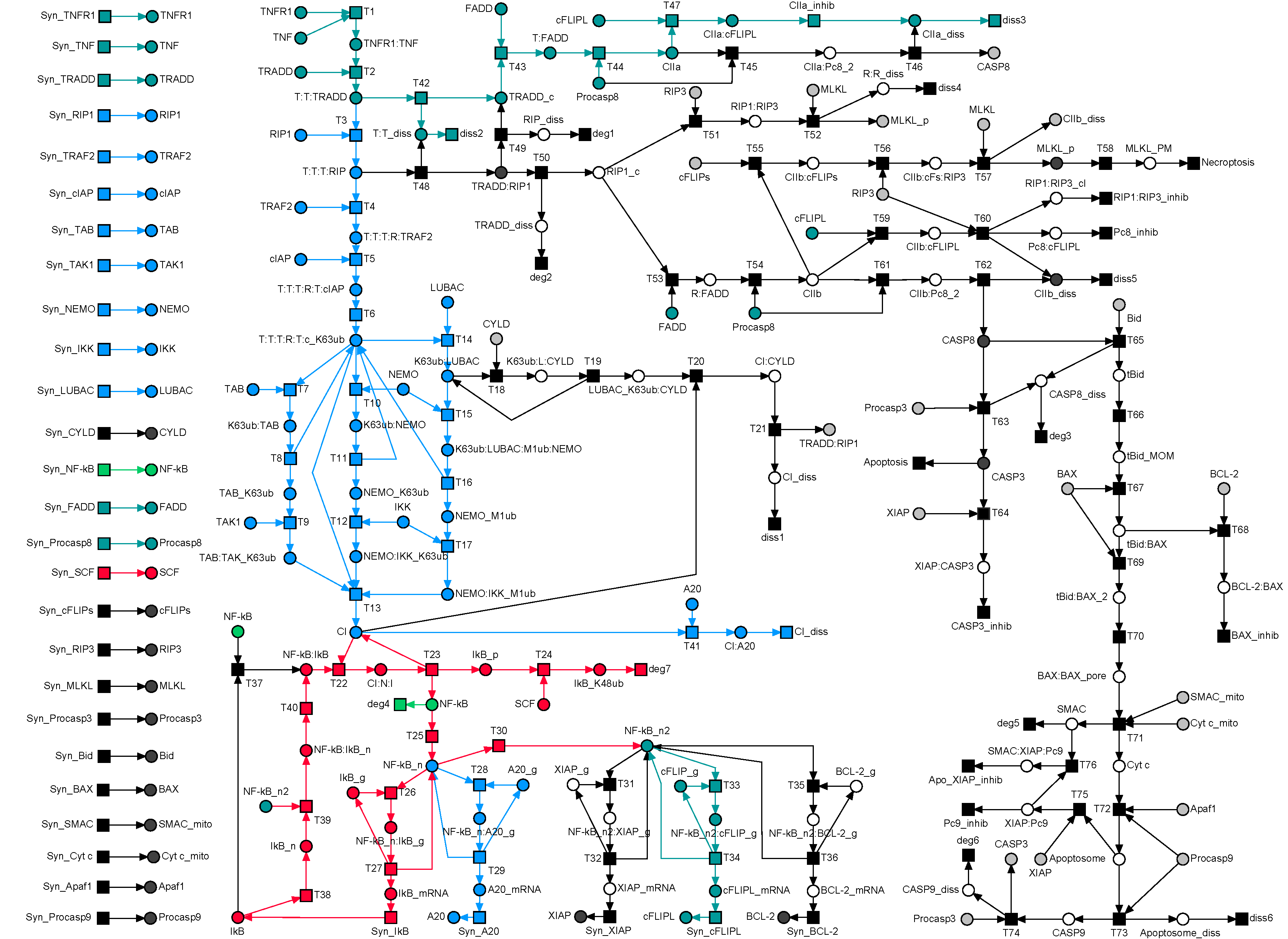
**

**S3 Fig:** Exemplary Manatee invariant, MI131.

Supplement: S3 Fig — MI131 is the linear combination of four transitions invariants (TIs), TI4 highlighted in dark green, TI9 highlighted in blue, TI15 highlighted in red, and TI18 highlighted in green. Note that, the TIs, i.e., their color code in the figure, may overlap. For detailed information on the TIs, we refer to S5 Table. MI131 represents a possible signal flow that is induced by the binding of TNF to the receptor TNFR, see TI9 and TI4, highlighted in blue and in dark green, respectively. The assembly of complex I, place CI, is part of TI9. MI131 combines the assembly of complex I with the degradation of complex I, see TI15, highlighted in red, and the synthesis of NF-κB, see TI18, highlighted in green. MI131 includes the inhibition of apoptosis by cFLIP, see TI4, highlighted in dark green. MI131 represents a complete pathway. MI131 resolves all relevant preconditions and interrelation of processes, as, e.g., the A20 feedback loop is accompanied by a preceding activation of NF-κB. (DOCX) [file pcbi.1010383.s011.docx]

**
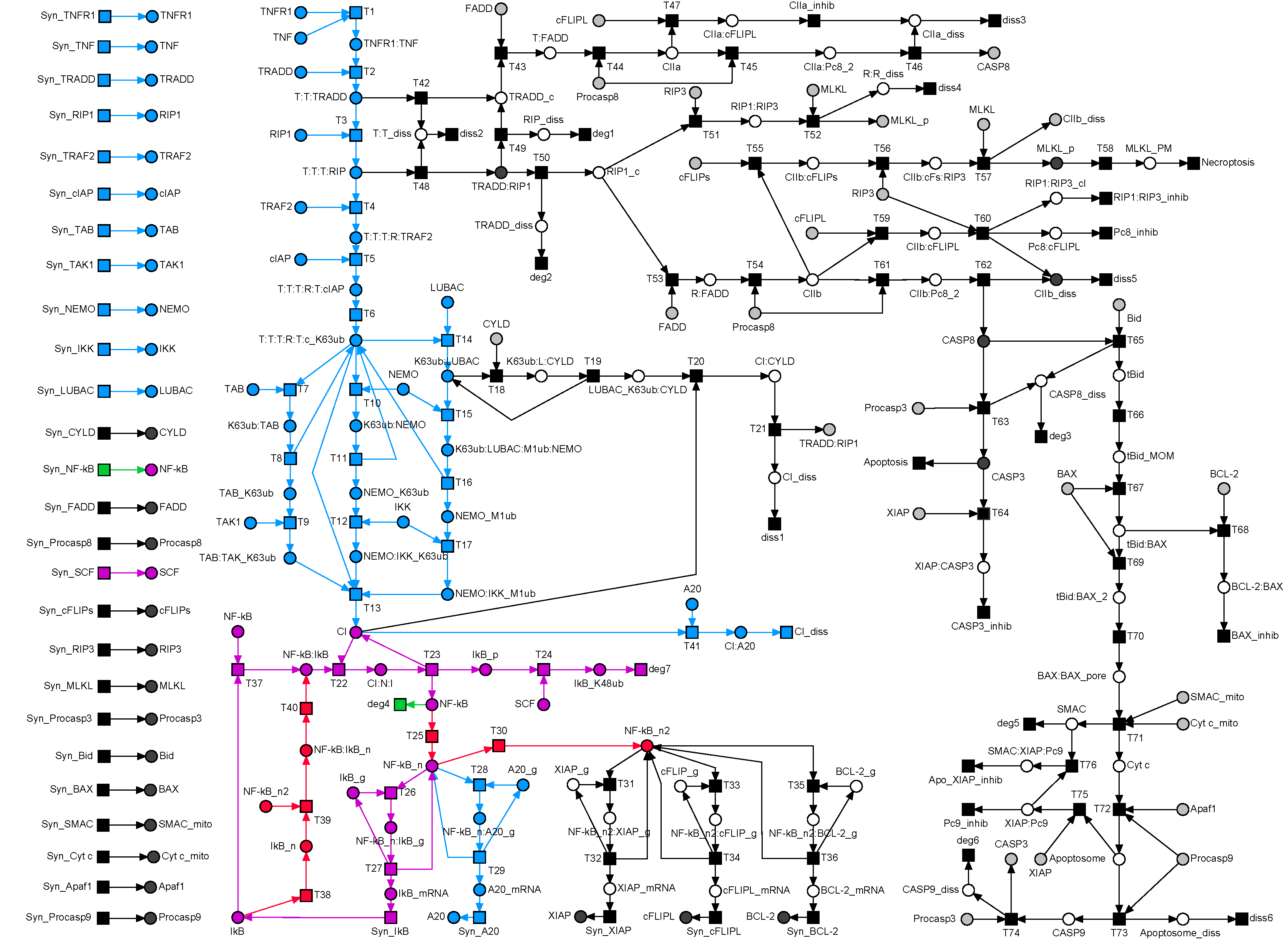
**

**S4 Fig:** Exemplary Manatee invariant, MI209.

Supplement: S4 Fig — MI209 is the linear combination of four transition invariants (TI), TI9, highlighted in blue, TI15, highlighted in red, TI16, highlighted in purple, and TI18, highlighted in green. Note that, the transition invariants, i.e., their color code in the figure, may overlap. For detailed information on the transition invariants, we refer to S5 Table. MI209 represents a possible signal flow that is induced by the binding of TNF to the receptor TNFR, see TI9, highlighted in blue. The assembly of complex I, place CI, is part of TI9. MI209 combines the assembly of complex I with the degradation of complex I, see TI16, highlighted in purple, the synthesis of NF-κB, see TI18, highlighted in green, and the translocation of NF-κB into the nucleus followed by the induction of transcription of IκB, see TI15, highlighted in red. MI209 represents a complete pathway. MI209 resolves all relevant preconditions and interrelation of processes, as, e.g., the A20 feedback loop is accompanied by a preceding activation of NF-κB. (DOCX) [file pcbi.1010383.s012.docx]
